# Transition-metal free C–N bond formation from alkyl iodides and diazonium salts via halogen-atom transfer

**DOI:** 10.1038/s41467-022-35613-7

**Published:** 2022-12-27

**Authors:** Jing Zhang, Min Jiang, Chang-Sheng Wang, Kai Guo, Quan-Xin Li, Cheng Ma, Shao-Fei Ni, Gen-Qiang Chen, Yan Zong, Hua Lu, Li-Wen Xu, Xinxin Shao

**Affiliations:** 1 College of Material, Chemistry and Chemical Engineering, Key Laboratory of Organosilicon Chemistry and Material Technology of Ministry of Education, Hangzhou Normal University, Hangzhou, 311121 Zhejiang China; 2 College of Biotechnology and Pharmaceutical Engineering, Nanjing Tech University, 30 Puzhu Rd S, Nanjing, 211816 China; 3 Department of Chemistry and Key Laboratory for Preparation and Application of Ordered Structural Materials of Guang-dong Province, Shantou University, Shantou, 515063 Guangdong China; 4 Academy for Advanced Interdisciplinary Studies and Department of Chemistry, Southern University of Science and Technology, 1088 Xueyuan Road, Shenzhen, 518055 China

**Keywords:** Synthetic chemistry methodology, Organic chemistry

## Abstract

Construction of C-N bond continues to be one part of the most significant goals in organic chemistry because of the universal applications of amines in pharmaceuticals, materials and agrochemicals. However, E2 elimination through classic S_N_2 substitution of alkyl halides lead to generation of alkenes as major side-products. Thus, formation of a challenging C(sp^3^)-N bond especially on tertiary carbon center remains highly desirable. Herein, we present a practical alternative to prepare primary, secondary and tertiary alkyl amines with high efficiency between alkyl iodides and easily accessible diazonium salts. This robust transformation only employs Cs_2_CO_3_ promoting halogen-atom transfer (XAT) process under transition-metal-free reaction conditions, thus providing a rapid method to assemble diverse C(sp^3^)-N bonds. Moreover, diazonium salts served as alkyl radical initiator and amination reagent in the reaction. Mechanism studies suggest this reaction undergo through halogen-atom transfer process to generate active alkyl radical which couples with diazonium cations to furnish final products.

## Abstract (web summary)

The pursuit of efficient C–N bond formation is a prime focus of synthetic organic chemistry. Here, the authors documented a base promoted amination between alkyl iodides and diazonium salts via halogen-atom transfer (XAT) process.

## Introduction

**Fig. 1:**
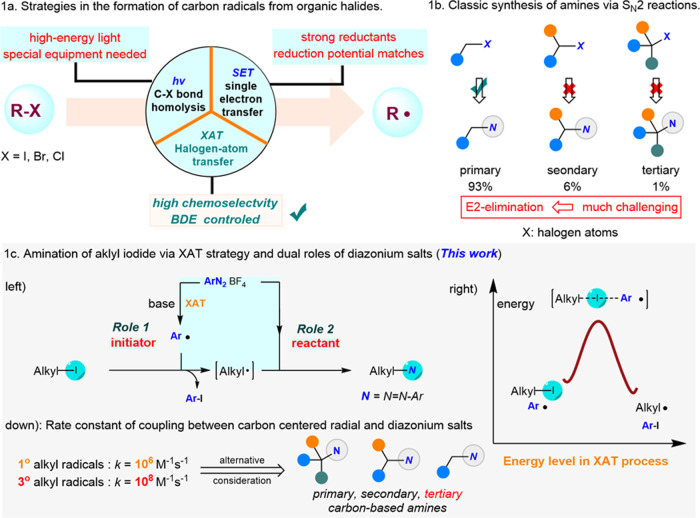
Pathways in the generation of alkyl radical and synthesis of challenging amines. **a** Comparation of homolysis of C-halogen bond, SET (single electron transfer), and XAT (halogen-atom transfer) for the formation of alkyl carbon radical. **b** Classic method for the construction of C–N bonds via S_N_2 substitutions. **c** Our reaction design: base-induced XAT (halogen-atom transfer) process of primary, secondary, and tertiary alkyl iodides and diazonium salts act as two critical roles in the amination of corresponding substrates.

Carbon radical has been well recognized as an easily-generated, reactive, and variable species in organic synthesis^1^. As a result, the design of reaction via radical process provides numerous opportunities to address the shortcomings in ionic chemistry^1^. For example, homolytic cleavage of C-halogen bonds of alkyl halide makes it possible for the generation of alkyl radical, which can be further functionalized to access highly valuable alkyl substituted products (Fig. 1a)^2^. However, this strategy usually requires specialized lights with high-energy and the reaction is always found to be with poor functional group tolerance^2–4^. More recently, two main approaches including excited metal catalysis (amido–Cu(I) complex)^5–10^ or metallaphotoredox catlysis^11–13^ combined with UV or visible light which were mainly developed by MacMillan, Fu, and Peters groups have been well-established with high efficiency (Fig. 1a, up). In particular, high-energy UV-light irradiation (hν = 254 nm) is utilized to promote the reaction largely depend on the matching of reduction potentials from different substrates (*E*_red_ < −2 V vs saturated calomel electrode for unactivated alkyl iodides)^2,3,14^.

Halogen-atom transfer (XAT), which much differs from the above two strategies, provides promising avenues for diverse functionalizations based on the bond dissociation energy (BDE) and the polarizability of the C-halogen bond (Fig. 1a, down)^1,15,16^. To trigger the homolytic activation of organic halides through XAT process, various abstractors such as α-aminoalkyl radicals, catalytic Mn specie, organosiliane, and Tin have been developed recently due to the weak C-halogen energy (50–70 kcal/mol for C–I bond)^1,17^. Leonori^17–21^ and Doyle groups^22,23^ independently disclosed α-aminoalkyl radicals could abstract the iodide atom under very mild reaction conditions to generate the active alkyl radical which would couple efficiently with broad nucleophiles to construct C–C, C–N, and C–S bonds. Manganese also exhibited powerful reactivity in converting alkyl iodides to alkyl radicals, producing a series of valuable compounds in high yields^24,25^. Very recently, Liu successfully achieved the copper-catalyzed difluoromethylation of alkyl iodides with high efficiency via halogen abstraction of C–I bonds by aryl radical from aryl diazonium salts, demonstrating the synthetic applicability of this powerful strategy^26^. Aliphatic amines have been recognized as one of the most privileged scaffolds in pharmaceuticals, agrochemicals, and organic chemistry due to their unique physical, chemical, and biological properties^27,28^. Consequently, it will be of great value to synthesize structure-varied mines, especially bearing a tertiary carbon atom and their derivatives which has become a long-standing goal for the chemists^29–38^. Classical S_N_2 amination of corresponding alkyl halides with nitrogen nucleophiles across the chemical industries represents a straightforward method to construct alkyl amines (Fig. 1b). However, this process is only limited to the primary substrate due to the less steric hindrance (~93% successful cases). In contrast, secondary (only ~6% successful cases) and tertiary (only ~1% successful cases) substrates are seriously difficult to process the displacement, and alkenes are obtained as the major side products through competitive E2-elimination^20,39^. Azo-compounds have been found as a kind of critical compounds which could be applied in numerous areas such as dyes^40^, photoswitches^41^, and pharmaceuticals^42^. Consequently, precise and efficient methods to access C–N bonds, especially on secondary or tertiary carbon atom centers through suitable pathways such as the radical process are much more problematic and challenging.

Dual role synthetic system of one compound has been widely applied in organic reactions by avoiding using of additional reagents, simplifying the operation, and improving the efficiency of the transformation^43–46^. Readily available diazonium salts have been recognized as versatile building blocks in organic synthesis because they are conveniently synthesized from the corresponding anilines and can undergo radical or electrophilic reaction pathways^47–56^. Moreover, the generation of aryl radical from diazonium salts requires additional equipment or technology such as thermal, photochemical, and electrochemical systems^56–60^. Kinetically favorable XAT occurs to deliver the aryl iodide (rate constant *k* = 10^9^M^−1^ s^−1^), resulting in the formation of more stable alkyl radicals (Fig. 1c, right)^24^. Inspired by the pioneering work on the iodine atom abstraction from the silyl methyl iodide moiety by Gevorgyan^61^, we envisioned whether diazonium salts could serve as both “radical abstractor” from alkyl halides for the formation of alkyl radical via XAT process and nitrogen source which can undergo coupling with the “in-situ” alkyl radical to assemble diverse C(sp^3^)-N bonds (Fig. 1c, left).

In this work, we developed an efficient method via XAT process in presence of base to construct a series of unsymmetric arylalkyldiazenes from alkyl iodides and diazonium salts. To achieve the proposed transformation, a suitable base is essential to promote the formation of aryl radical from diazonium salts, while additional reductants were required to promote the SET process from diazonium salts to generate the aryl radical in the previous reports^47,62–64^. Compared to primary alkyl radical, the tertiary carbon-centered radical was considered as more reactive toward the coupling with diazonium salts due to the higher nucleophilic character (Fig. 1c, down)^65^. Thus, unsymmetric arylalkyldiazenes which were also interesting are generated after the combination of alkyl radical and diazonium salts.

## Results and discussion

### Reaction optimizations

We initially select 4-methoxybenzenediazonium salt **1a** as the dual-role reagent and commercially available iodocyclopentane **2a** as the radical precursor (Table 1). It was found that common inorganic bases such as Cs_2_CO_3_, K_2_CO_3_ were quite effective, promoting the generation of the coupled product **3a** in high yield after 2 h, while the reaction conducted with weaker base, stronger base, or in the absence of base lead to lower efficiency (Table 1, entry 1–5). It was found that the reaction exhibited high efficiency in dark (Table 1, enrty 6). Using polar MeOH as the solvent, the XAT transformation could afford the coupled product **3a** in high yield, while other polar solvents, such as MeCN, DMF led to lower yields (Table 1, entried 8–9). Moreover, no desired product was detected when CH_2_Cl_2_ was employed as the solvent (Table 1, entry 7). In particular, the reaction was proved to be sensitive to air since poor efficiency was found when the reaction was conducted under air (Table 1, entry 10).

**Table 1:** Reaction optimization


Entry	Variation from the entry “standard conditions”	Yield of 3a (%)^*a*^
1	none	96 (94)^*b*^
2	K_2_CO_3_ instead of Cs_2_CO_3_	90
3	KOAc instead of Cs_2_CO_3_	<5
4	*t*-BuOK instead of Cs_2_CO_3_	<5
5	Et_3_N instead of Cs_2_CO_3_	57
6	In dark	95
7	CH_2_Cl_2_	<5
8	MeCN	70
9	DMF	56
10	under air	<5

^a^Reaction conditions: **1a** (0.3 mmol, 3.0 equiv), **2a** (0.1 mmol), and Cs_2_CO_3_ (0.15 mmol, 1.5 equiv) in MeOH (1.0 mL) at 22 °C for 2 h. Yield was determined by ^1^H NMR spectroscopy in the presence of CH_2_Br_2_ as an internal standard. ^b^Isolated yield.

**Fig. 2:**
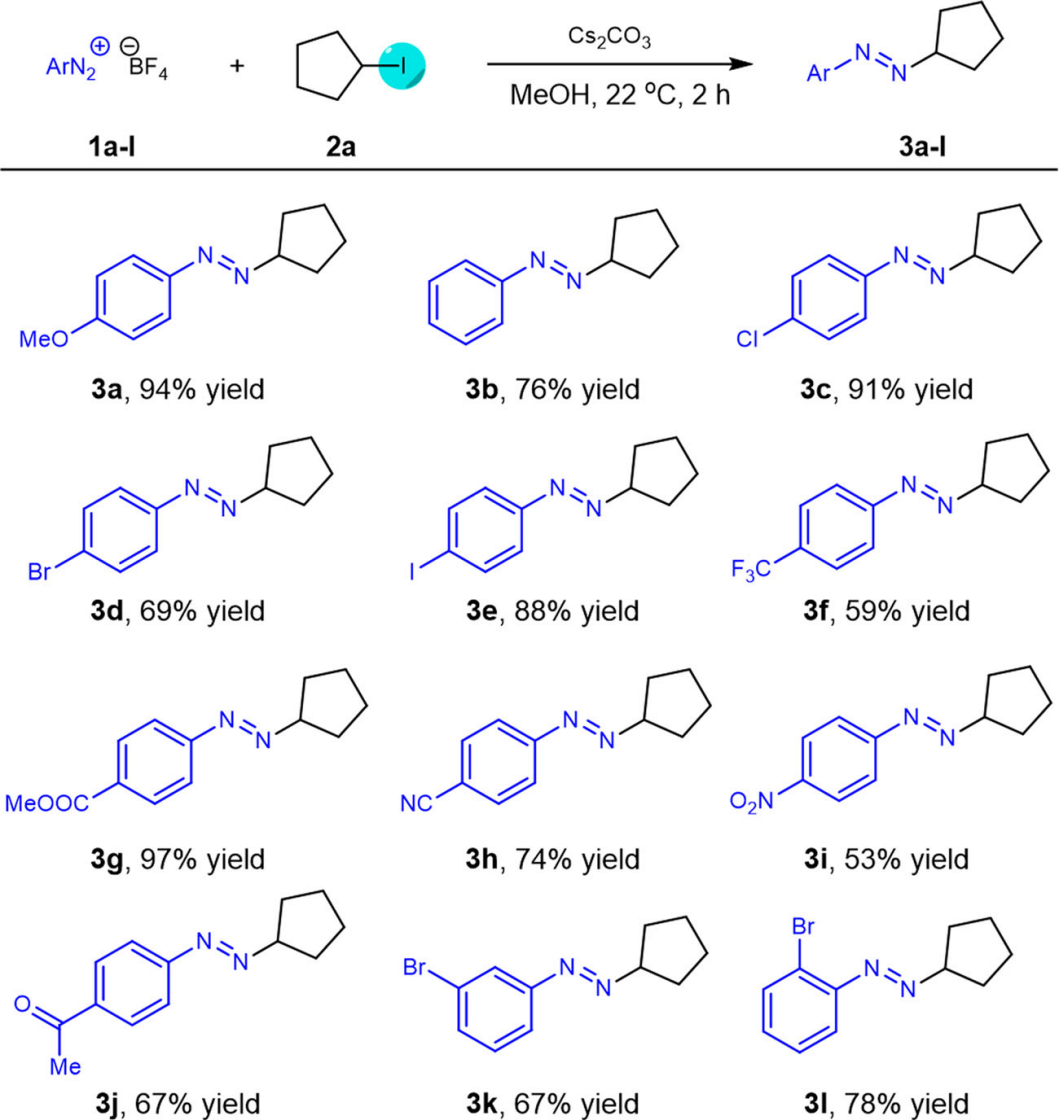
Substrate scope of diazonium salts. Reaction conditions: **1a**-**l** (1.5 mmol, 3.0 equiv), **2a** (0.5 mmol), and Cs_2_CO_3_ (0.75 mmol, 1.5 equiv) in MeOH (5.0 mL) at 22 °C for 2 h. Isolated yield.

Substrate scope. With optimized conditions in hand, we next turned our focus to explore the generality of this practical amination via XAT process with respect to diazonium salts (Fig. 2). Coupled partners bearing diverse functional substituents at *para*- position of the benzene ring underwent this mild transformation smoothly, affording the corresponding products in good to excellent yields (**3a**–**e**). It was noted that the CF_3_, a fluorine-containing group that was prevalent in pharmaceuticals, was compatible with the transformation. Electron-withdrawing groups (COOMe, CN, NO_2_, and C(O)Me) substituted substrates were also evaluated and the corresponding azo-compounds **3g**−**j** were obtained in good yields. In addition, steric hindrance did not affect the efficiency of this established process, and the products **3k**−**l** were obtained with good yields.

**Fig. 3:**
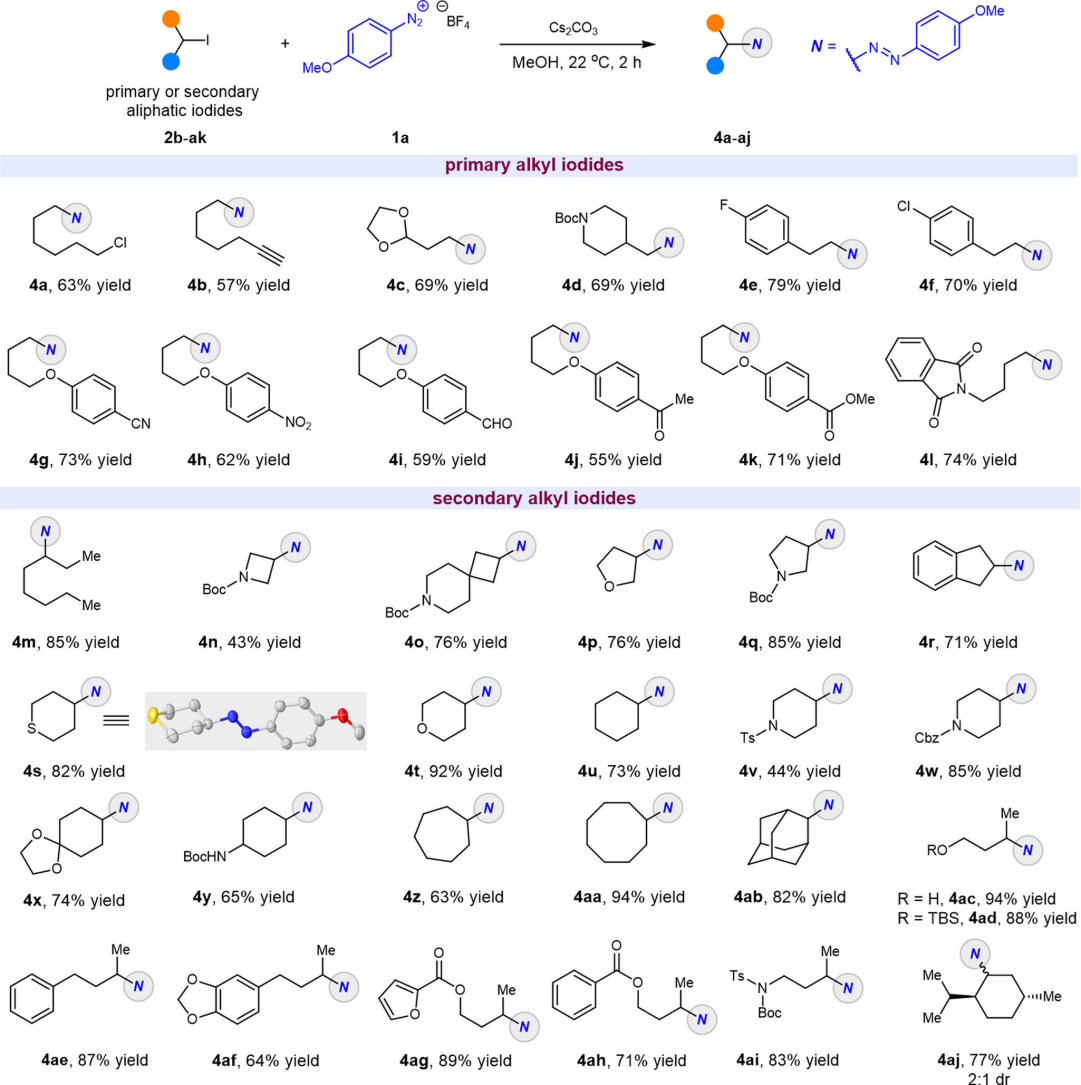
Substrate scope of primary and secondary alkyl iodides. Reaction conditions: **1a** (1.5 mmol, 3.0 equiv), **2b–aj** (0.5 mmol, 1.0 equiv), and Cs_2_CO_3_ (0.75 mmol, 1.5 equiv) in MeOH (5.0 mL) at 22 °C for 2 h. Isolated yield.

Encouraged by the general applications of various diazonium salts coupling with model alkyl iodide **2a**, the transformation towards the scope of the alkyl iodides was then systematically evaluated (Fig. 3). Over-amination usually occurred in the classic S_N_2 nucleophilic substitution when the corresponding substrates containing two reacted sites. In contrast, primary iodide **2b** bearing a chloride atom under our XAT process was chemo-selectively coupled with **1a**, thus providing an alternative opportunity to prepare challenging azo-compound **4a** and do further diversifications. Similarly, this practical protocol exhibited excellent functional compatibility and efficiency for a wide variety of alkyl iodides, furnishing a series of azo-compound in moderate to good yields. Unactivated alkyne **4b**, ethylene ketal derivate **4c**, Boc protected amine **4d**, fluobenzene **4e** and chlorobenzene derivate **4f** could participate well in the two components coupling, and the products were isolated in moderate to good yields. Alkyl iodides containing electron-withdrawing groups (CN, NO_2_, CHO, C(O)Me, COOMe, and amide) were screened and the desired products **4g**−**l** were generated in good yields. Likewise, a number of functionalized azo compounds were obtained after simple column purification through this mild and efficient strategy. The chemistry was then applied to the substrates bearing long-chain or cyclic aliphatic alkanes and enabled the amination to give the corresponding products **4m**, **4r**, **4u**, **4y**−**aa**, **4ae**−**af** in good to excellent yields. In particular, coupling of the model diazonium salt **1a** with heterocyclic iodides were successfully achieved, providing a practical route to access structure-varied azo-compounds **4n**−**q**, **4s**−**t**, **4v**−**w** which might not be easily synthesized by other reported means. It was worth noting that the single crystal structure of compound **4s** showed that the diazo group located at the *trans*-position of the aryl groups (for details, see the Supplementary Information). Moreover, alkyl iodides contained ethylene ketal, adamantane and ester were competent coupling partners, furnishing the desired products **4x**, **4ab**, and **4ag**−**ah** with high efficiency. Iodinated N-Boc and Ts protected amine **4ai** was also comparable as well as iodinated TBS-protected primary alcohol **4ad** and free alcohol **4ac**, resulting in good conversions. Finally, late-stage amination of complex molecule such as menthol was achieved, illustrating the potential synthetic utility of this practical XAT process.

**Fig. 4:**
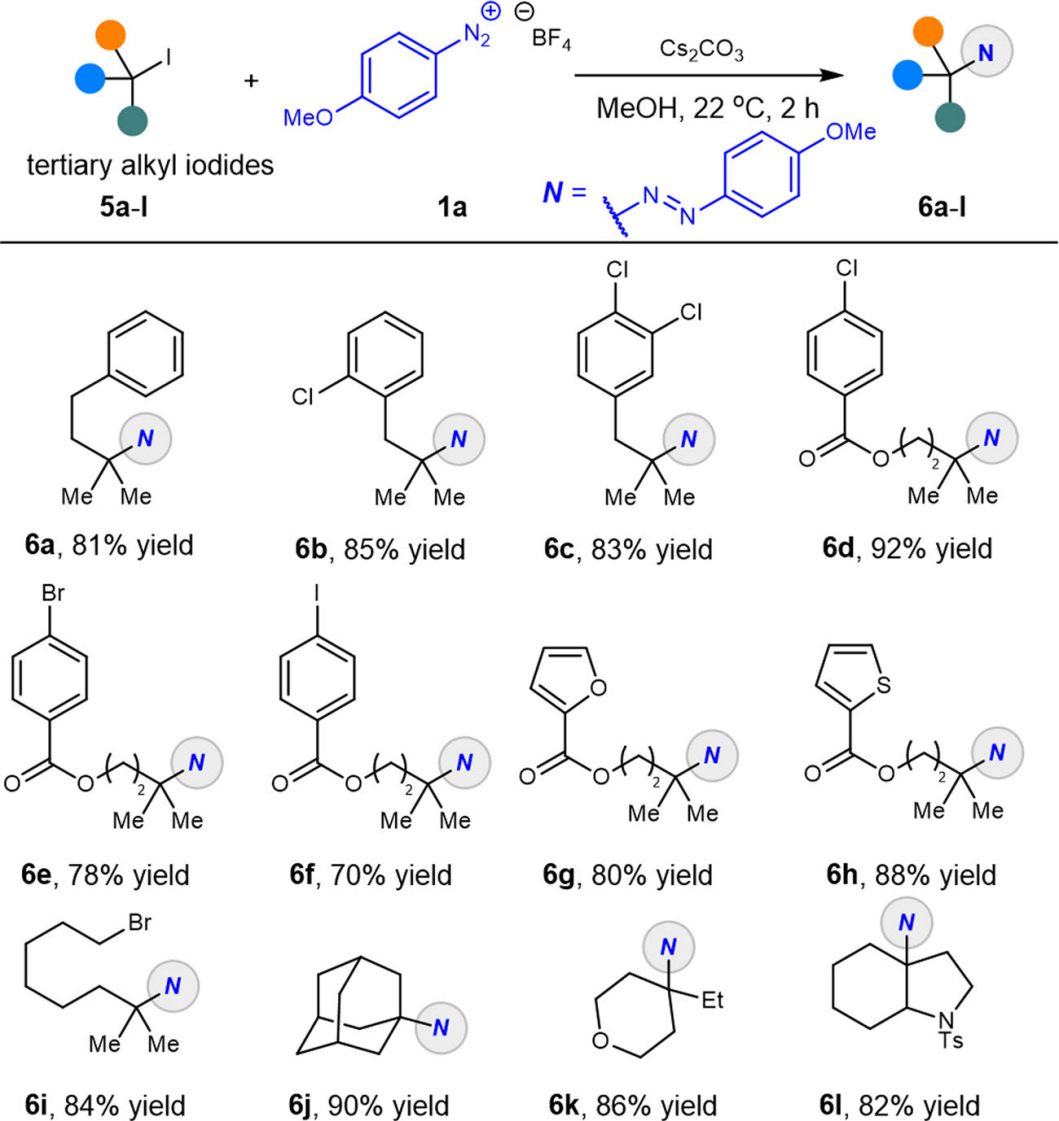
Substrate scope of tertiary iodides. Reaction conditions: **1a** (1.5 mmol, 3.0 equiv), **6a–l** (0.5 mmol, 1.0 equiv), and Cs_2_CO_3_ (0.75 mmol, 1.5 equiv) in MeOH (5.0 mL) at 22 °C for 2 h. Isolated yield.

The construction of tertiary C(sp^3^)-N bonds is still a recognized challenge in traditionally synthetic chemistry, here a wide range of tertiary alkyl iodides which were conveniently prepared from corresponding alcohols in one step could couple well with our model substrate **1a** as shown in Fig. 4. A wide variety of functional groups including Cl, Br, and I were tolerated under this mild transformation, resulting in good to excellent yields of the products **6a**–**f**. Heteroaryl iodides **5g**–**h** were also investigated, affording the final products **6g**–**h** in good to excellent yields. Excellent chemo-selectivity was also obtained when the terminal position of the tertiary alkyl iodide was installed with a bromide atom, and product **6i** was isolated in yield. Finally, cyclic alkyl iodides **5j**–**l** were also suitable for the XAT process with high efficiency, demonstrating excellent compatibility on the substrates scope. At this point it should be noted that our practical amination from alkyl iodides through XAT process provides a practical route to construct a series of C(sp^3^)-N bonds under mild and reaction conditions.

**Fig. 5:**
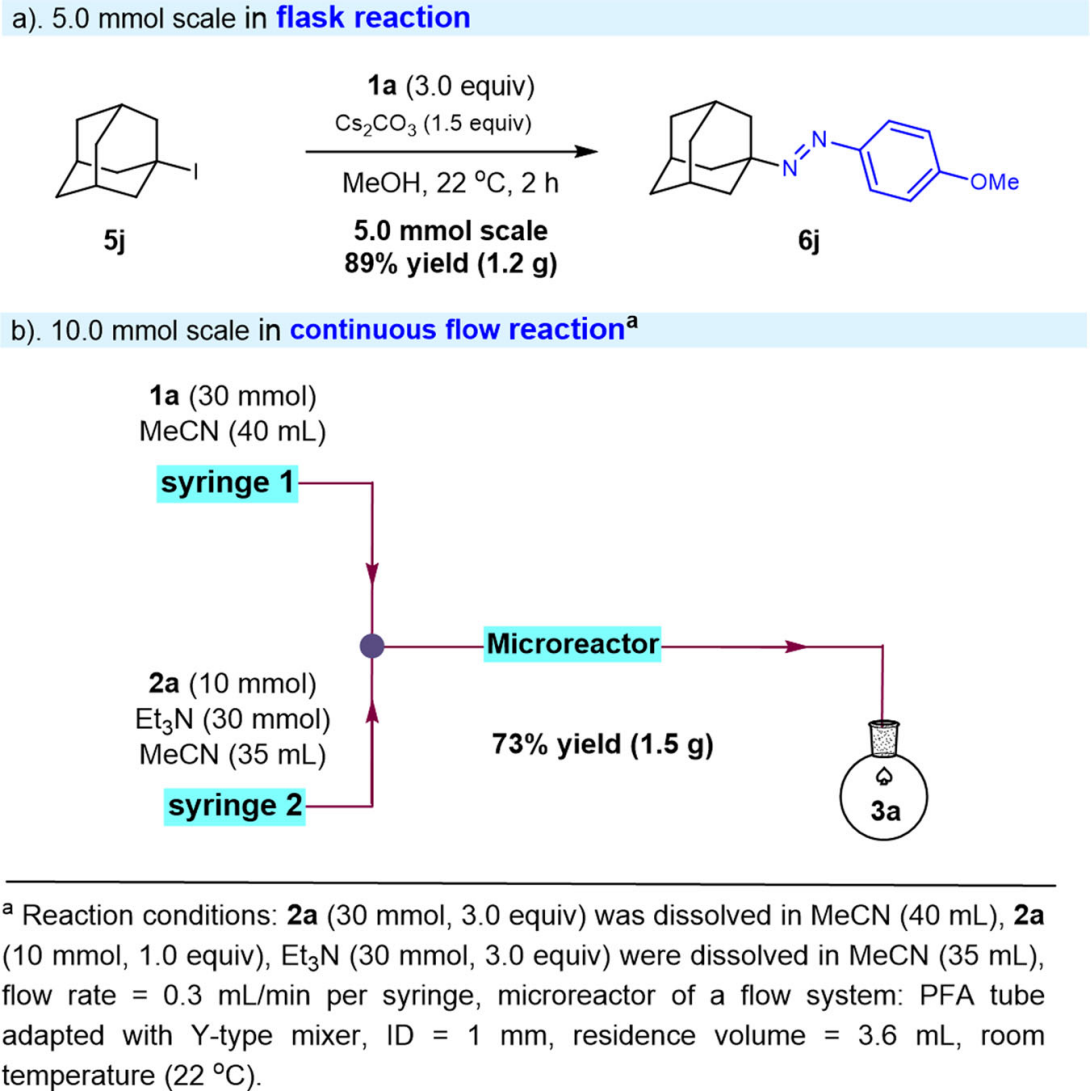
Bigger scale reaction. **a** 5.0 mmol scale in flask conditions. **b** 10.0 mmol scale in flow conditions.

To illustrate the synthetic potential of this practical amination of alkyl iodides, a gram-scale reaction was carried out with the alkyl iodide **5j** and model diazonium salts **1a** under the standard reaction conditions (Fig. 5a). The coupled product **6j** was successfully isolated in 89% yield on 5.0 mmol after simple column chromatography purification. Continuous-flow coupling on 10.0 mmol was further explored by simply switching the base to Et_3_N due to the poor solubility of diazonium salts **1a** and Cs_2_CO_3_ in MeOH and the solvent to MeCN, respectively. As a consequence, the corresponding product **3a** was obtained in 73% yield (1.5 g) after purification under these mild reaction conditions. Thus, the XAT reaction could be scaled up easily in either flask or continuous-flow system, demonstrating the practicality and potential application of the method.

### Synthetic applications

**Fig. 6:**
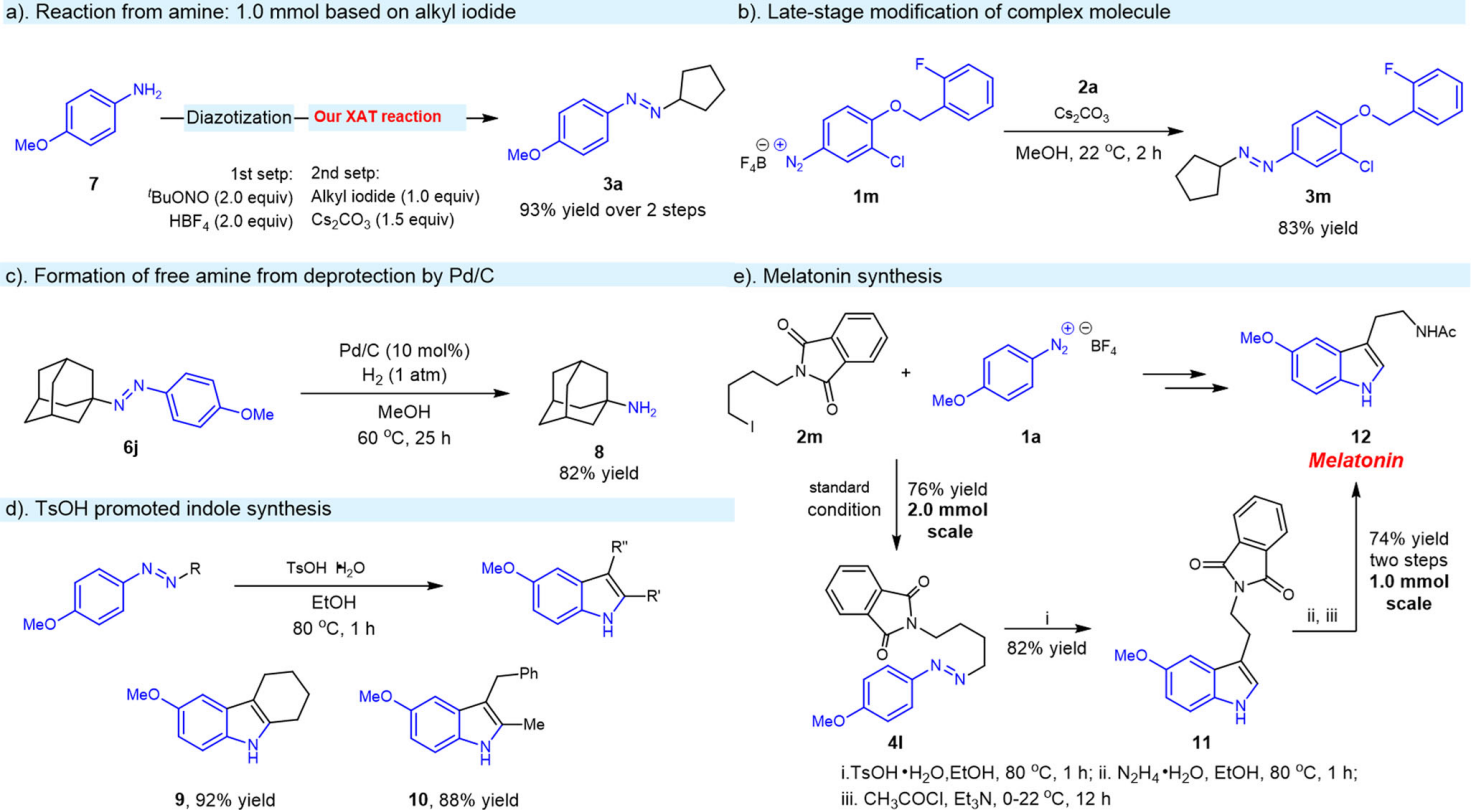
Synthetic applications. **a** Reaction from amine: 1.0 mmol based on alkyl iodide. **b** Late-stage modification of complex molecule. **c** Formation of free amine from deprotection by Pd/C. **d** *p*-Toluenesulfonic acid monohydrate promoted indole synthesis. **e** Melatonin synthesis.

Two steps involving further purification in the synthesis of the diazonium salts **1a** in EtOH and subsequent amination via XAT we established were successfully performed and the corresponding product **3a** was generated in comparable yields (Fig. 6a). Late-stage amination of a more complex molecule was also tested and the reaction underwent smoothly with high efficiency, showing the general applicability of this method (Fig. 6b). Furthermore, The N=N bond of the azo product **6j** could be successfully transformed into NH_2_ in the presence of Pd/C and H_2_ (Fig. 6c). Fischer indole synthesis by the straightforward conversion of our XAT products into the functionalized indoles **9** and **10** in high yield was achieved in the presence of *p*-toluenesulfonic acid monohydrate (Fig. 6d). Melatonin played a critical role in the mitigation of sleeping disorders and a four-step process to prepare Melatonin has been well developed from the primary alkyl iodide **2m** (Fig. 6e). 2.0 mmol scale reaction to generate the corresponding product **4l** in 76% isolated yield by employing our well-established transition-metal-free amination method was successfully conducted. Fischer indole formation, and followed traditional hydrazinolysis of the phthaloyl group led to the generation of the free amine, which was further treated with acetyl chloride in the presence of Et_3_N and the melatonin **12** could be isolated in 74% yield (1.0 mmol scale, two steps).

### Mechanistic study

**Fig. 7:**
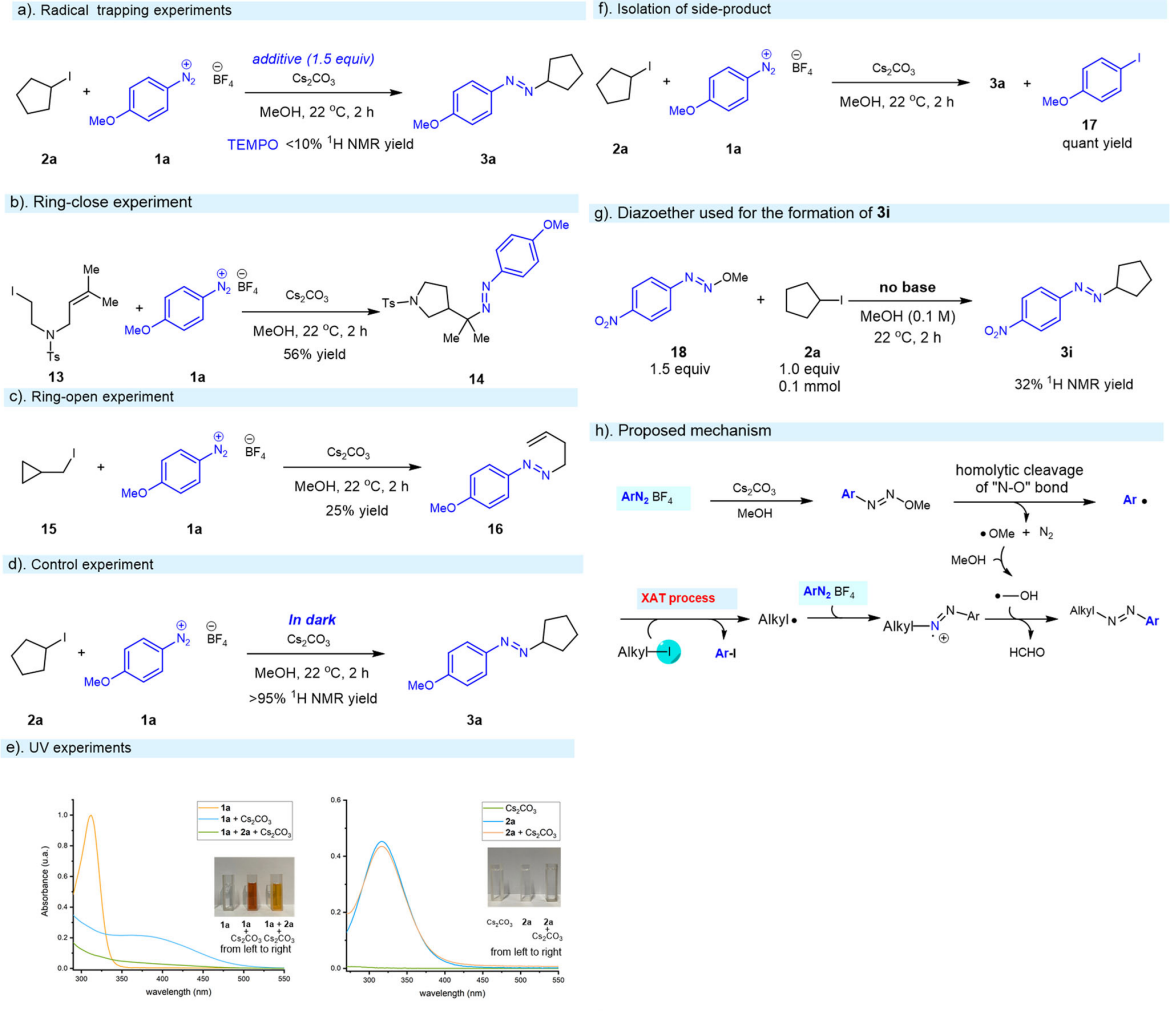
Mechanistic experiments. **a** Radical trapping experiment by employing TEMPO. **b** Radical clock reaction: ring-closed experiment. **c** Radical clock reaction: ring-open experiment. **d** Control experiment. **e** UV experiments. **f** Isolation of side product. **g** Diazoether used for the formation of **3i**. **h** Proposed mechanism.

To seek more insights into the mechanism of this mild and practical amination of alkyl iodides, several mechanistic experiments were performed (Fig. 7). To identify whether radicals are generated in the current XAT process, radical scavenger such as TEMPO was introduced as the additives to the reaction mixture. The coupled product **3a** was not observed and this result suggested a radical process was involved in this transformation (Fig. 7a). Next, radical clock experiments were further performed, delivering the corresponding products **14** (ring-closed) and **15** (ring-open) in 56% and 25% yield, respectively (Fig. 7b, c). To validate whether the light has a big impact on the XAT process, the control experiment “in dark” indicated the light did not play a significant role in the transformation since the efficiency was not affected according to the ^1^H NMR (Fig. 7d). Since the color of the reaction mixture changed to red immediately after the addition of the base such as Cs_2_CO_3_ due to the strong interaction between the diazonium salts and base (Fig. 7e). A series of UV/vis absorption spectroscopy studies were conducted to help understanding the mechanism. UV/vis absorption of the individual starting materials including base or combination of iodocyclopentane **2a** and Cs_2_CO_3_ were recorded, and the corresponding peaks of the absorption spectroscopy remained unchanged (Fig. 7e, right). However, a larger redshift of the mixture of diazonium salts **1a** and Cs_2_CO_3_ was observed, illustrating a stronger interaction between the diazonium salts **1a** and Cs_2_CO_3_, or the diazonium salts **1a** could be activated by a base such as Cs_2_CO_3_ (Fig. 7e, left). Moreover, the redshift disappeared shortly after quick addition of iodocyclopentane **2a** into the mixture of the diazonium salts **1a** and Cs_2_CO_3_, indicating the alkyl iodide might react fast with the “active” radical species generated from the diazonium salts and base. Importantly, 1-iodo-4-methoxybenzene **17** was isolated in quant yield, which supports that the aryl radical could abstract the iodine atom from the corresponding alkyl iodide in the reaction (Fig. 7f). The diazoether **18** which could be formed in the alcoholic media under basic conditions^66,67^ was successfully subjected to the coupling with alkyl iodide **2a** in the absence of base (Fig. 7g). Consequently, the desired product **3i** was detected in 32% ^1^H NMR yield and GC-MS (For details, see the Supplementary Information).

Based on the results we achieved above and related reports^58,59,68^, a proposed mechanism was outlined in Fig. 7h: The first step of the reaction could be the generation of diazoether from corresponding diazonium salt in the presence of base such as Cs_2_CO_3_^55,64^. Then it underwent homolytic cleavage of “N-O” bond, resulting in the release of nitrogen gas immediately along with the formation of aryl radical since number of bubbles were obviously observed and the color of the reaction mixture was changed to orange (Fig. 7e, UV experiments) after addition of the base^55^. The active alkyl radical and aryl iodide were formed efficiently via the abstraction of an iodine atom from an alkyl iodide by “in situ” generated aryl radical. Trapping by a diazonium ion, the active alkyl radical could transform into the nitrogen radical cation, which was quenched by the hydroxymethyl radical generated from the HAT process between a methoxyl radical and methanol (for detailed discussion and density functional theory (DFT) calculation, see the Supplementary Information and Supplementary Data 1).

### Limitations of the current method

**Fig. 8:**
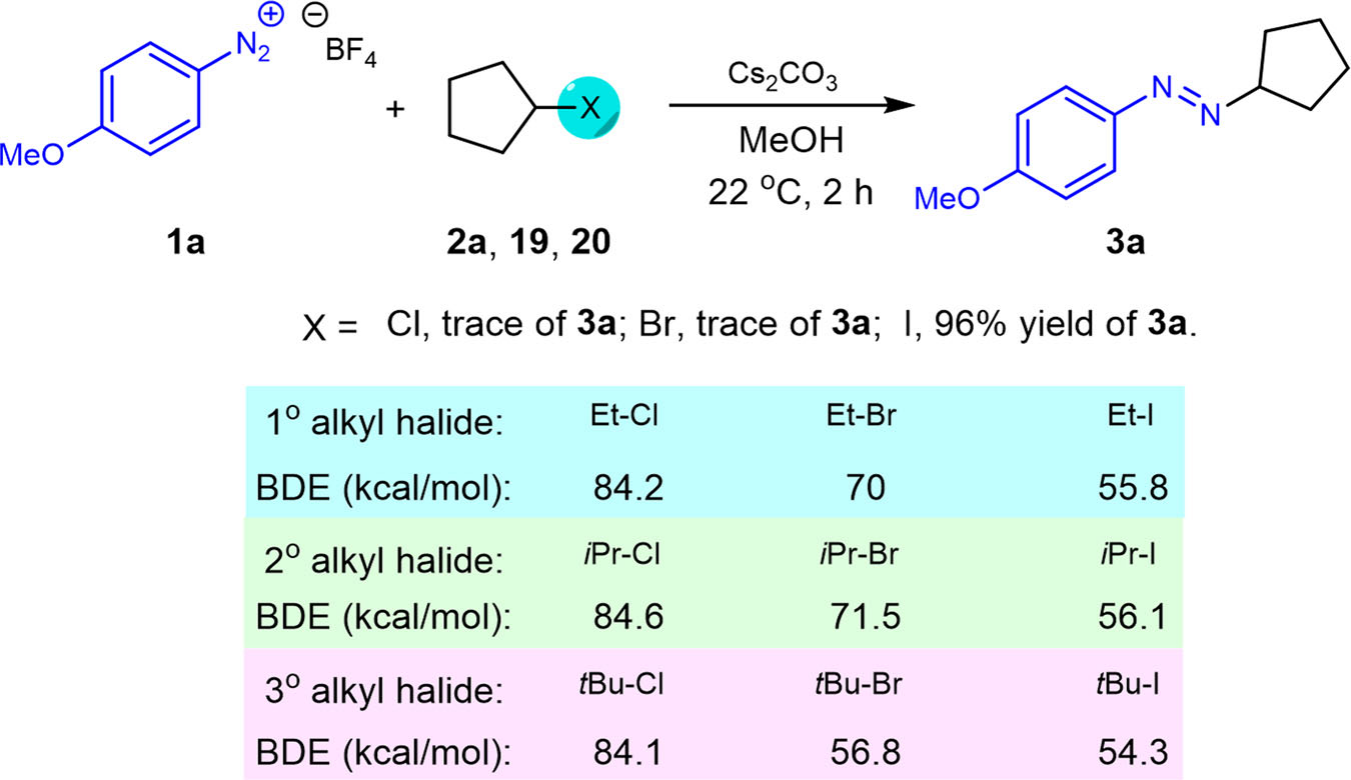
Other alkyl halides studied in current XAT process. The results employing cyclopentyl chloride, bromide, and iodide as alkyl partners in our XAT transformation and BDE difference between them.

To further expand the scope of this process, The coupling reaction bromocyclopentane **19** and chlorocyclopentane **20** were investigated as the radical precursor. However, >95% recovery of the starting materials **19** and **20** was observed with the full decomposition of the diazonium salts under current reaction conditions (Fig. 8). Since the efficiency in XAT of different alkyl halides can be varied according to the BDE^1^. As a result, the relative rates for halogen-atom abstractions were determined by the strength of the C-halogen bonds, and the result of our experiments also supports that the trend of halogen-atom abstractions is iodides>bromides>chlorides^1,69,70^.

In summary, we reported a transition-metal-free strategy for the synthesis of diverse azo-compounds via practical XAT process between diazonium salts and alkyl iodides under mild reaction conditions. Moreover, the diazonium salts were confirmed to act as both oxidants and radical acceptors in redox-neutral reactions. Initial mechanistic experiments and DFT calculation supported this practical amination undergoing generation of aryl radical from diazonium salts promoted by suitable base, XAT (halogen-atom transfer) process from alkyl iodide and aryl radical, and final coupling reaction between alkyl radical with diazonium salts to deliver functionalized azo-compounds. Detailed experiments on the in-situ formation of aryl radicals and other interesting transformations are being explored and these results will be reported in the future.

## Methods

### General procedure for the amination of alkyl iodides and diazonium tetrafluoroborate

A 25-mL glass vial fitted with a stirring bar was charged with diazonium tetrafluoroborate (1.50 mmol, 3.00 equiv) and Cs_2_CO_3_ (244 mg, 0.750 mmol, 1.50 equiv). The mixture was evacuated and backfilled with nitrogen three times. Then the alkyl iodide (0.500 mmol, 1.00 equiv) and MeOH (5.00 mL) were added under nitrogen. The mixture was allowed to stir at 22 °C for 2 h. After the completion of reaction, the reaction mixture was poured into H_2_O (50.0 mL) and extracted with EtOAc (20.0 mL × 3). The combined organic layers were washed with H_2_O (20.0 mL), dried over Na_2_SO_4_, and filtered. The solvent was removed by rotary evaporation and the residue was purified by flash silica gel chromatography (Caution: alkyl-aryl azo-compounds are usually not stable in the solution of organic solution such as CH_2_Cl_2_, CDCl_3_ and should be handled carefully). Full experimental details and characterization of new compounds in this study are provided in the Supplementary Information.

## Supplementary information

Supplementary Information

Description of Additional Supplementary Data 1

Supplementary Data 1

## Data Availability

The authors declare that all the data supporting this study, including the experimental details, data analysis, and spectra for all unknown compounds, see Supplementary Files. All data underlying the findings of this work are available from the corresponding author upon request. The X-ray crystallographic coordinates for structures reported in this study have been deposited at the Cambridge Crystallographic Data Centre (CCDC), under deposition number 2181806 (**4s**). These data are provided free of charge by the joint Cambridge Crystallographic Data Centre and Fachinformationszentrum Karlsruhe Access Structures service www.ccdc.cam.ac.uk/structures.
