# Supplementary material for: Transition-metal free C–N bond formation from alkyl iodides and diazonium salts via halogen-atom transfer
Source: Nat Commun. 2022 Dec 27;13:7961. doi: 10.1038/s41467-022-35613-7 (PMC9794826; doi:10.1038/s41467-022-35613-7)
Supplement: Supplementary file 1 — Supplementary Information [file 41467_2022_35613_MOESM1_ESM.pdf]

Supplementary Information

Transition-metal Free C-N Bond Formation from Alkyl Iodides and Diazonium Salts via Halogen-atom Transfer

Jing Zhang[1], Min Jiang[1], Chang-Sheng Wang[2*], Kai Guo[2*], Quan-Xin Li,[3] Cheng Ma,[3] Shao-Fei Ni[3], Gen-Qiang Chen[4], Yan Zong[4], Hua Lu[1*], Li-Wen Xu[1] and Xinxin Shao[1*]

[1] College of Material, Chemistry and Chemical Engineering, Key Laboratory of Organosilicon Chemistry and Material Technology of Ministry of Education, Hangzhou Normal University, Hangzhou 311121, Zhejiang, China.

[2] College of Biotechnology and Pharmaceutical Engineering, Nanjing Tech University, 30 Puzhu Rd S, Nanjing, 211816, China.

[3] Department of Chemistry and Key Laboratory for Preparation and Application of Ordered Structural Materials of Guang-dong Province, Shantou University, Shantou 515063, Guangdong, China.

[4] Academy for Advanced Interdisciplinary Studies and Department of Chemistry, Southern University of Science and Technology, 1088 Xueyuan Road, Shenzhen, 518055, China

*Correspondence to: changshengwang1989@163.com; guok@njtech.edu.cn; hualu@hznu.edu.cn; xxshao@hznu.edu.cn

Table of Contents

Supplementary Methods S4

1. General information S4

2. Synthesis of the starting materials S6

2.1. General Method A: Synthesis of arenediazonium tetrafluoroborates 1a-l S6

2.2. Synthesis of arenediazonium tetrafluoroborates 1m S7

2.3 General Method B: Synthesis of alkyl iodide 2b, 2c, 2e, 2n, 2p, 2q, 2t, 2u, 2ag, 2ah and 2ak S7

2.4 General Method C: Synthesis of primary alkyl iodide 2d, 2f, 2g, 2h, 2i, 2j, 2k, 2l, 2m S8

2.5 General Method D: Synthesis of alkyl iodide 2o, 2r-s, 2v, 2y, 2aa, 2ab-ac, 2ae-af, 2ai S9

2.6 Synthesis of 4-iodo-1-tosylpiperidine 2w S11

2.7 Synthesis of benzyl 4-iodopiperidine-1-carboxylate 2x S12

2.8 Synthesis of 3-iodobutan-1-ol 2ad S12

2.9 Synthesis of *N*-Boc, *N*-tosyl-3-Iodobutylamine 2ai S13

2.10 General Method F: Synthesis of tertiary alkyl iodide 5a, 5d-h, 5j S13

2.11 Synthesis of 4-ethyl-4-iodotetrahydro-2*H*-pyran 5k S16

2.12 Synthesis of 3-iodo-1-tosyloctahydro-1H-indole 5l 17

3. Optimization of the reaction conditions S18

3.1 Evaluation of different solvents S18

3.2 Evaluation of different base S19

3.3 Evaluation of different reaction time S19

3.4 Evaluation of equivalents of the arenediazonium tetrafluoroborates S19

4. Substrate scope: General Method F. S20

5. 5.0 mmol scale in flask reaction S47

6. Gram scale experiment in flow S48

7. Reaction from amine: 1.0 mmol based on alkyl iodide S49

8. Formation of free amine from deprotection by Pd/C S49

9. Synthetic application S50

9.1. General Method G: TsOH promoted Indole synthesis S50

9.2 Melatonin synthesis S52

10. Mechanistic studies S53

10.1 The experimental procedure for capturing radicals with TEMPO S53

10.2 Ring-close experiment S55

10.3 Ring-open experiment S55

10.4 Control experiment S56

10.5 Isolation of side-product S56

10.6 Dizaoether 18 was used directly in the coupling with alkyl iodide 1a. 58

10.7 Detection of another side-product: aldehyde S60

10.8 Detection of (*E*)-1,2-bis(4-methoxyphenyl)diazene. S61

10.9 Other alkyl halides' reaction. S62

11. The UV-vis absorption spectra of the mixtures of 1a, 2a, Cs_2CO_3 .... S63

12. Computational studies S65

13. Disscussion on the mechanism. S66

14. Crystallographic data S67

15. NMR spectrum of the products S73

Supplementary References S147

16. References S147

Supplementary Methods

1. General information

General procedures. General Information Unless specifically stated, all reagents were commercially obtained and where appropriate, purified prior to use. For example, dichloromethane (DCM), acetonitrile (MeCN) was freshly distilled from CaH_2; toluene, ether (Et_2O) was dried and distilled from metal sodium and benzophenone. Other commercially available reagents and solvents were used directly without purification. Reactions were monitored by thin layer chromatography (TLC) using silica gel plates. Flash column chromatography was performed over silica (200 - 300 mesh). ^1H, ^{13}C, ^{19}F NMR spectra were recorded on a Bruker 400 MHz or 500 MHz spectrometer in $CDCl_3$ or CD_3COCD_3 or DMSO-d_6 or CDOD. Multiplicities were given as: s (singlet); d (doublet); dd (doublets of doublet); t (triplet); q (quartet); td (triplet of doublets); m (multiplets). High resolution mass spectra (HRMS) of the products were obtained on a Bruker Daltonics micro TOF-spectrometer.

Reagents. The following chemicals were used as received: 1-Adamantanol (Energy-Chemical), 2-Adamantanone (Energy-Chemical), Aniline (Energy-Chemical), 4-Aminobenzonitrile (Energy-Chemical), Benzyl chloroformate (Energy-Chemical), Benzylacetone (Energy-Chemical), 2-Bromoaniline (Energy-Chemical), 3-Bromoaniline (Energy-Chemical), 4-Bromoaniline (Energy-Chemical), 4-Bromobenzoyl chloride (Energy-Chemical), 6-Bromo-1-hexanol (Energy-Chemical), Cesium carbonate (Adamas), 2-Chlorophenylacetic acid (Energy-Chemical), 4-Chloroaniline (Energy-Chemical), 6-Chloro-1-hexano (Energy-Chemical), Cycloheptanone (Energy-Chemical), Cyclohexenylethylamine (Energy-Chemical), Cyclooctanone (leyan.com), Cyclopentyl iodide (leyan.com), 1,4-Dioxaspiro[4.5]decan-8-one (leyan.com), 3,4-Dichlorophenylacetic acid (Energy-Chemical), Di-tert-butyl dicarbonate (Energy-Chemical), 4-Fluorophenylacetic acid (Energy-Chemical), 4-Hydroxy benzaldehyde (Energy-Chemical), 4'-Hydroxyacetophenone (leyan.com), Hept-6-yn-1-ol (Energy-Chemical), Hydrogen tetrafluoroborate (Energy-Chemical), Imidazole

(Adamas), Iodine (Energy-Chemical), 4-Iodoaniline (Energy-Chemical), 2-Methyl-4-phenyl-2-butanol (Energy-Chemical), 3-Methyl-1,3-butanediol, 4-Methoxyaniline (Energy-Chemical), 4-Methoxyphenylacetic acid (Energy-Chemical), N-Boc-4-piperidinemethanol (Energy-Chemical), 4-Nitroaniline (Energy-Chemical), 4-Oxopiperidinium chloride (Energy-Chemical), Piperonyl acetone (Energy-Chemical), Potassium Acetate (SCR), Potassium carbonate (SCR), Potassium carbonate (SCR), Potassium fluoride (Energy-Chemical), Potassium iodide (Adamas), Potassium Phosphate Dibasic (SCR), Potassium phosphate tribasic (SCR), *p*-Toluenesulfonyl chloride (Adamas), Sodium acetate trihydrate (SCR), Sodium bicarbonate (SCR), Sodium carbonate (SCR), Sodium carbonate (SCR), Sodium fluoride (Energy-Chemical), Sodium hydroxide (SCR), Sodium nitrite (SCR), Sodium nitrite (SCR), *tert*-Butyl 2-oxo-7-azaspiro[3.5]nonane-7-carboxylate (Energy-Chemical), *tert*-Butyldimethylsilyl chloride (Adamas), 2-Thiophenecarbonyl chloride (Energy-Chemical), 4-(Trifluoromethyl)aniline (Energy-Chemical), Triphenylphosphine (J&K).

2. Synthesis of the starting materials

2.1. <u>General Method A</u>: Synthesis of arenediazonium tetrafluoroborates 1a-l

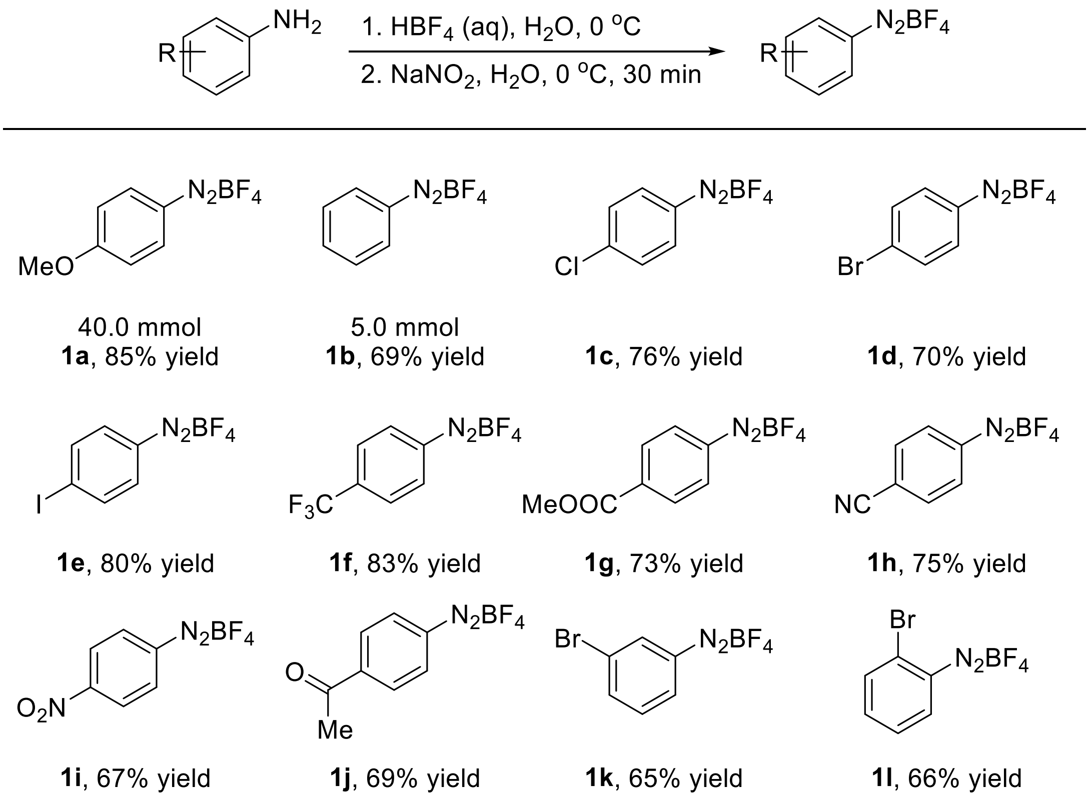

Supplementary Fig. 1. Arenediazonium tetrafluoroborates 1a-l

In a 120 °C oven-dried 10-mL glass, equipped with a stir bar, the aniline (10.0 mmol, 1.00 equiv) was dissolved in a mixture of H_2O (3.50 mL) and an aqueous solution of HBF_4 (48.0 wt%, 3.00 equiv, 5.48 g). The mixture was cooled at 0 °C with an ice bath and a solution of $NaNO_2$ (0.760 g, 1.10 equiv, in 1.50 mL H_2O) was added dropwise. The reaction was stirred at 0 °C for 30 min. The arenediazonium tetrafluoroborate was precipitated several times by the addition of the solid was filtered ice-cooled Et_2O (20.0 mL), and washed with small amounts of cooled the crude product was Et_2O, dissolved in the minimal amount of acetone. The arenediazonium tetrafluoroborate was the precipitated by the slow addition of Et_2O. Spectra were consistent with the literature data[1].

2.2. Synthesis of arenediazonium tetrafluoroborates 1m

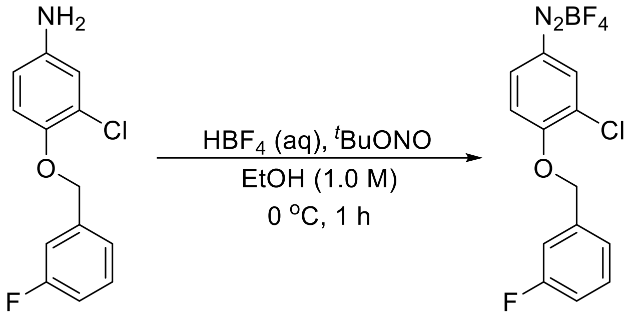

In a 120 °C oven-dried 25-mL glass, equipped with a stir bar, the aniline (5.00 mmol, 1.00 equiv) was dissolved in a mixture of absolute EtOH (5.00 mL) and an aqueous solution of HBF_4 (48.0 wt%, 1.20 mL). The mixture was cooled at 0 °C with an ice bath and ^{t}BuONO (1.35 mL) was added dropwise. The reaction was stirred at 0 °C for 1 h. The arenediazonium tetrafluoroborate was precipitated several times by the addition of the solid was filtered ice-cooled Et_2O (20.0 mL), and washed with small amounts of cooled the crude product was Et_2O, dissolved in the minimal amount of acetone. The arenediazonium tetrafluoroborate was the precipitated by the addition of Et_2O and the final product was isolated as a yellow solid (1.53 g, 4.36 mmol, 87.3% yield). Spectra were consistent with the literature data[2].

2.3 General Method B: Synthesis of alkyl iodide 2b, 2c, 2e, 2n, 2p, 2q, 2t, 2u, 2ag, 2ah and 2ak

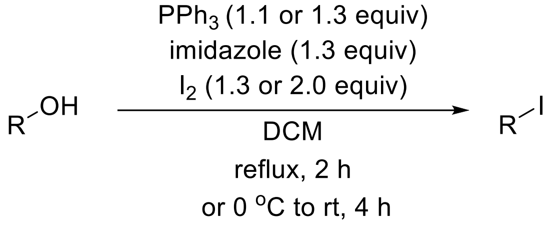

A 120 °C oven-dried 100-mL round-bottom flask, equipped with a stir bar, was charged with alcohol (5.00 mmol, 1.00 equiv), PPh_3 (1.70 g, 6.50 mmol, 1.30 equiv), imidazole (0.442 g, 6.50 mmol, 1.30 equiv) and DCM (15.0 mL) were added under nitrogen. I_2 (1.65 g, 6.50 mmol, 1.30 equiv) was then added to the mixture. The mixture was allowed to stir at 50 °C for 2 h. After the completion of reaction, H_2O (50.0 mL) was added and the mixture was extracted with DCM (50.0 mL × 3). The combined organic layers were washed with brine

(10.0 mL), dried over Na_2SO_4 and filtrated. The solvent was removed by rotary evaporation and the residue was purified by flash silica gel chromatography to give the desired product.

Compound **2c**: colorless oil (0.686 g, 3.09 mmol, 61.8% yield), Spectra were consistent with the literature data[3].

Compound **2ag**: colorless oil (1.04 g, 3.42 mmol, 68.4% yield). Spectra were consistent with the literature data[4].

For synthesis of compounds **2b**, **2e**, **2n**, **2p**, **2q**, **2t**, **2u**, **2ah** and **2ak**: 6.0 mmol scale of corresponding alcohols and PPh_3 (1.13 g, 7.20 mmol, 1.20 equiv), imidazole (0.511 g, 7.50 mmol, 1.30 equiv) and DCM (10.0 mL) were used.

Compound **2b**: colorless oil (0.764 g, 3.11 mmol, 51.8% yield), Compound **2q**: (0.793 g, 4.00 mmol, 66.7% yield), Compound **2u**: colorless oil (0.951 g, 4.49 mmol, 74.8% yield), Spectra were consistent with the literature data[5].

Compound **2e**: (0.686 g, 2.11 mmol, 35.2% yield), Spectra were consistent with the literature data[6].

Compound **2n**: (0.905 g, 3.77 mmol, 62.8% yield), Spectra were consistent with the literature data[7].

Compound **2p**: colorless oil (0.331 g, 0.942 mmol, 15.7% yield), Compound **2t**: colorless oil (0.852 g, 3.74 mmol, 62.3% yield), Spectra were consistent with the literature data[8].

Compound **2ah**: colorless oil (0.706 g, 2.65 mmol, 44.2% yield), Spectra were consistent with the literature data[9].

Compound **2ak**: colorless oil (0.865g, 3.25 mmol, 54.2% yield), Spectra were consistent with the literature data[9].

2.4 General Method C: Synthesis of primary alkyl iodide 2d, 2f, 2g, 2h, 2i, 2j, 2k, 2l, 2m

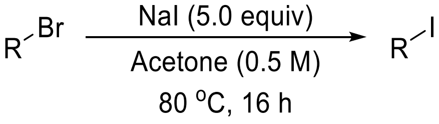

A 120 °C oven-dried 100-mL round-bottom flask, equipped with a stir bar, was charged with NaI (3.75 g, 25.0 mmol, 5.00 equiv), alkyl bromide (5.00 mmol, 1.00 equiv) and acetone

(10.0 mL) were added under nitrogen. The mixture was allowed to stir at 80 °C for 16 h. After the completion of reaction, H_2O (50.0 mL) was added and the mixture was extracted with EtOAc (30.0 mL × 3). The combined organic layers were washed with brine (10.0 mL), dried over Na_2SO_4 and filtrated. The solvent was removed by rotary evaporation and the residue was purified by flash silica gel chromatography.

Compound **2d**: (1.04 g, 4.57 mmol, 91.3% yield), Spectra were consistent with the literature data[10].

Compound **2f**: (0.686 g, 3.09 mmol, 61.8% yield), Spectra were consistent with the literature data[11].

Compound **2g**: white solid (1.35g, 5.00 mmol, 100% yield), Spectra were consistent with the literature data[12].

Compound **2h**: white solid (1.28g, 4.26 mmol, 85.2% yield), Compound **2i**: white solid (1.30 g, 4.06 mmol, 81.2% yield), Spectra were consistent with the literature data[13].

Compound **2j**: colorless oil (1.15 g, 3.79 mmol, 75.8% yield), Spectra were consistent with the literature data[14].

Compound **2k**: colorless oil (1.37 g, 4.31 mmol, 86.2% yield), Compound **2k**: colorless oil (1.32 g, 3.96 mmol, 79.2% yield), Spectra were consistent with the literature data[15].

Compound **2m**: white solid (1.33 g, 4.71 mmol, 94.2% yield), Spectra were consistent with the literature data[16].

2.5 <u>General Method D</u>: Synthesis of alkyl iodide 2o, 2r-s, 2v, 2y, 2aa, 2ab-ac, 2ae-af, 2ai

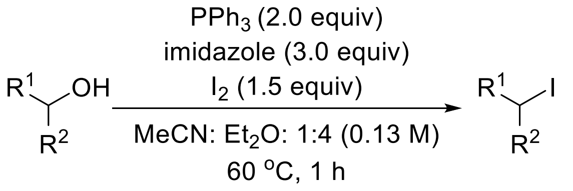

A 120 °C oven-dried 100-mL round-bottom flask, equipped with a stir bar, was charged with alcohol (10.0 mmol, 1.00 equiv), PPh_3 (5.25 g, 20.00 mmol, 2.00 equiv), imidazole (2.04 g, 30.00 mmol, 3.00 equiv) and CH_3CN: Et_2O (1: 4, 75.0 mL) were added under nitrogen. I_2 (3.81 g, 15.00 mmol, 1.50 equiv) was added to the mixture. The mixture was allowed to stir

at 60 °C for 1 h. After the completion of reaction, H_2O (50.0 mL) was added and the mixture was extracted with EtOAc (50.0 mL × 3). The combined organic layers were washed with brine (10.0 mL), dried over Na_2SO_4 and filtrated. The solvent was removed by rotary evaporation and the residue was purified by flash silica gel chromatography to give the desired products.

Compound **2o**: colorless oil (0.411 g, 1.45 mmol, 14.5% yield, at rt for 12 h), Compound **2r**: colorless oil (prepared on 5.00 mmol scale, 1.34 g, 4.51 mmol, 90.2% yield), Spectra were consistent with literature data[17].

Compound **2s**: white solid (prepared on 5.00 mmol scale, 0.969 g, 3.97 mmol, 79.4% yield, 4 h), Spectra were consistent with literature data[18].

Compound **2v**: colorless oil (prepared on 5.00 mmol scale, 0.338 g, 1.61 mmol, 32.2% yield), Compound **2aa**: colorless oil (2.19 g, 9.77 mmol, 97.7% yield, at rt for 12 h), Compound **2ab**: colorless oil (0.789 g, 3.31 mmol, 33.1% yield, at rt for 12 h), Spectra were consistent with literature data[19].

Compound **2y**: colorless oil (prepared on 5.00 mmol scale, 1.11 g, 4.14 mmol, 82.8% yield, at rt for 12 h), Compound **2z**: colorless oil (prepared on 5.00 mmol scale, 1.11 g, 3.42 mmol, 68.4% yield), Compound **2ae**: white solid (prepared on 5.00 mmol scale, 1.22 g, 3.88 mmol, 77.6% yield), Spectra were consistent with literature data[4].

Compound **2ac**: white solid (2.46 g, 9.38 mmol, 93.8% yield, at rt for 12 h), Spectra were consistent with literature data[20].

Compound **2af**: colorless oil (prepared on 20.0 mmol scale, 1.88 g, 7.23 mmol, 36.2% yield), Spectra were consistent with literature data[21].

Compound **2ai**: colorless oil (prepared on 5.00 mmol scale, 1.52 g, 5.00 mmol, 100% yield). Spectra were consistent with literature data[22].

2.6 Synthesis of 4-iodo-1-tosylpiperidine 2w

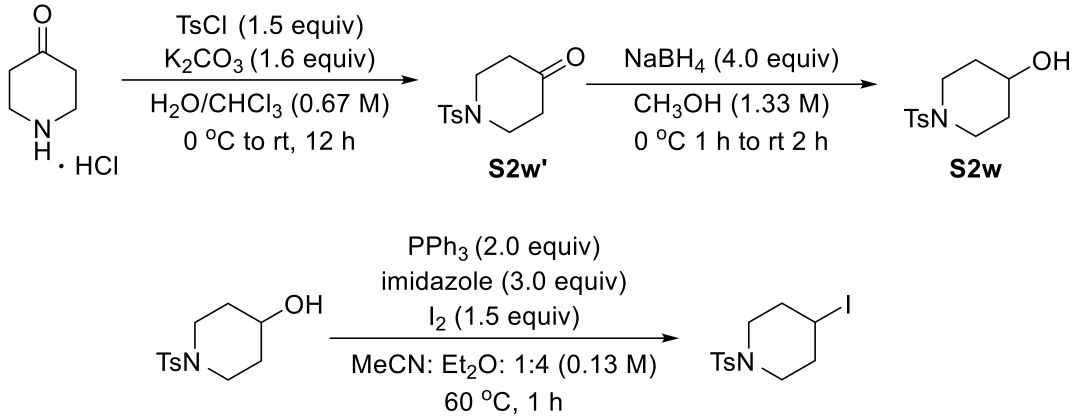

A 120 °C oven-dried 100-mL round-bottom flask, equipped with a stir bar, was charged with 4-oxopiperidinium chloride (2.71 g, 20.0 mmol, 1.00 equiv) and $CHCl_3$ (15.0 mL) were added under nitrogen. K_2CO_3 aqueous (4.42 g, 32.0 mmol, 1.60 equiv, dissolved in water 15.0 mL) was added to the mixture. The mixture was cooled to 0 °C and TsCl (5.72 g, 30.0 mmol, 1.50 equiv) was added in portions. The reaction mixture was warmed to room temperature and was monitored by TLC. After the completion of reaction, H_2O (50.0 mL) was added and the mixture was extracted with $CHCl_3$ (50.0 mL × 3). The combined organic layers were washed with brine (10.0 mL), dried over Na_2SO_4 and filtrated. The solvent was removed by rotary evaporation and the residue was purified by flash silica gel chromatography. The product (**S2w'**) was isolated as a white solid (4.72 g, 18.6 mmol, 93.2% yield). Spectra were consistent with literature data[23].

This compound (**2w**) was prepared according to General procedure C.

Compound **S2w**: white solid (prepared on 10.0 mmol scale, 2.38 g, 9.32 mmol, 93.2% yield). Spectra were consistent with literature data[24].

Compound **2w**: white solid (prepared on 5.00 mmol scale, 0.98 g, 2.68 mmol, 53.7% yield). Spectra were consistent with literature data[4].

2.7 Synthesis of benzyl 4-iodopiperidine-1-carboxylate 2x

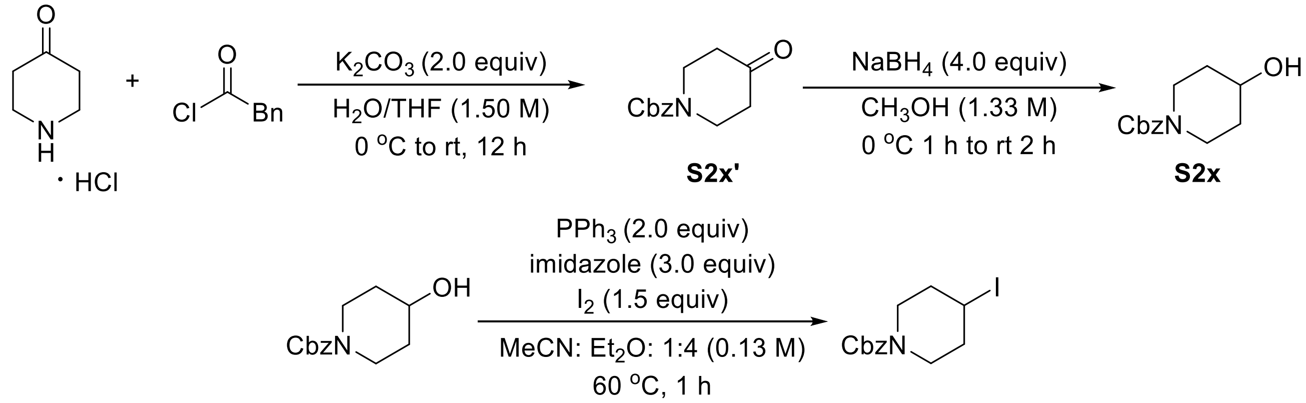

A 120 °C oven-dried 100-mL round-bottom flask, equipped with a stir bar, was charged with 4-oxopiperidinium chloride (2.71 g, 20.00 mmol, 1.00 equiv) and THF (20.0 mL) were added under nitrogen. K_2CO_3 aqueous (5.53 g, 40.00 mmol, 2.00 equiv, dissolved in water 10.0 mL) was added to the mixture. The mixture was cooled to 0 °C and benzyl chloroformate (3.75 g, 22.00 mmol, 1.10 equiv) was added dropwise. The reaction mixture was warmed to room temperature and allowed to stir for 12 h. After the completion of reaction, H_2O (50.0 mL) was added and the mixture was extracted with EtOAc (50.0 mL × 3). The combined organic layers were washed with brine (10.0 mL), dried over Na_2SO_4 and filtrated. The solvent was removed by rotary evaporation and the residue was purified by flash silica gel chromatography. The product (**S2x'**) was isolated as a colorless oil (3.97 g, 17.0 mmol, 85.1% yield).

This compound (**2x**) was prepared according to *General procedure C*.

compound **S2x**: colorless oil (prepared on 10.0 mmol scale, 2.25 g, 9.56 mmol, 95.6% yield), compound **2x**: colorless oil (prepared on 5.00 mmol scale, 1.36 g, 3.94 mmol, 78.8% yield). Spectra were consistent with literature data[4].

2.8 Synthesis of 3-iodobutan-1-ol 2ad

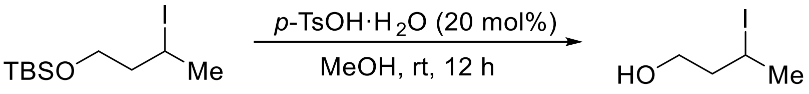

A 120 °C oven-dried 200-mL round-bottom flask, equipped with a stir bar, was charged with 2-iodo-4-(*tert*-butyldimethylsilyloxy)butane (1.50 g, 4.77 mmol, 1.00 equiv) and MeOH (48 mL) were added under nitrogen. Then *p*-toluenesulfonic acid monohydrate (182 mg,

0.950 mmol, 0.2 equiv) was added to the mixture and the mixture was allowed to stir at room temperature for 4 h. After the completion of reaction, a saturated aqueous solution of $NaHCO_3$ was added and the mixture was extracted with Et_2O (50.0 mL × 3). The combined organic layers were washed with brine (10.0 mL), dried over Na_2SO_4 and filtrated. The solvent was removed by rotary evaporation and the residue was purified by flash silica gel chromatography to provide the desired products as a colorless liquid (712 mg, 3.56 mmol, 74.6% yield). Spectra were consistent with literature data[25].

2.9 Synthesis of N-Boc, N-tosyl-3-Iodobutylamine 2ai

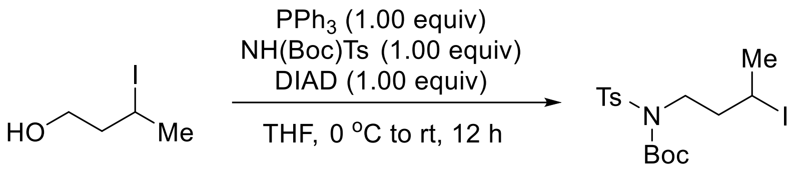

A 120 °C oven-dried 200-mL round-bottom flask, equipped with a stir bar, was charged with 3-Iodobutan-1-ol (730 mg, 3.65 mmol, 1.00 equiv), PPh_3 (957 mg, 3.65 mmol, 1.00 equiv), NH(Boc)Ts (990 mg, 3.65 mmol, 1.00 equiv) and THF (22 mL) were added under nitrogen. A solution of DIAD (1.11 g, 5.48 mmol, 1.50 equiv) in THF (5.0 mL) was added dropwise to the mixture at 0 °C. Then the mixture was allowed to stir at room temperature for 12 h. After the completion of reaction, the solvent was removed by rotary evaporation and the residue was purified by flash silica gel chromatography to provide the desired products as a colorless liquid (1.25g, 2.75 mmol, 75.3% yield). Spectra were consistent with literature data[25].

2.10 General Method F: Synthesis of tertiary alkyl iodide 5a, 5d-h, 5j

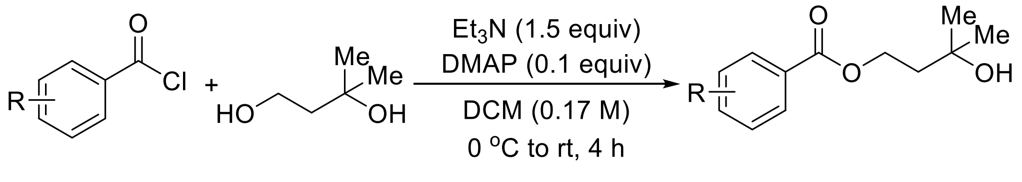

A 120 °C oven-dried 200-mL round-bottom flask, equipped with a stir bar, was charged with 3-methyl-butane-1,3-diol (1.56 g, 15.0 mmol, 1.50 equiv), DMAP (0.120 g, 1.00 mmol,

0.100 equiv), Et_3N (1.52 g, 15.0 mmol, 1.50 equiv) and DCM (50.0 mL) were added under nitrogen. The mixture was cooled to 0 °C and benzoyl chloride (10.0 mmol, 1.00 equiv) was added dropwise. The reaction mixture was warmed to room temperature and allowed to stir for 4 h. After the completion of reaction, H_2O (50.0 mL) was added and the mixture was extracted with DCM (50.0 mL × 3). The combined organic layers were washed with brine (10.0 mL), dried over Na_2SO_4 and filtrated. The solvent was removed by rotary evaporation and the residue was purified by flash silica gel chromatography to provide the desired products.

compound **S5d**: white solid (2.26 g, 6.76 mmol, 67.6% yield), compound **S5g**: colorless oil (1.98 g, 10.0 mmol, 100% yield). Spectra were consistent with literature data[26].

compound **S5e**: colorless oil (2.22 g, 7.73 mmol, 77.3% yield), compound **S5f**: white solid (2.26 g, 6.76 mmol, 67.6% yield), Spectra were consistent with literature data[27].

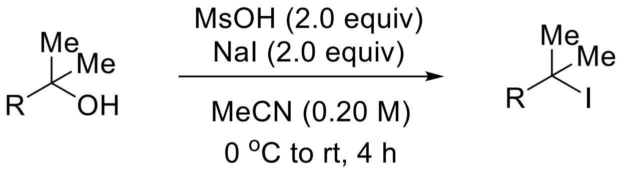

A 120 °C oven-dried 100-mL round-bottom flask, equipped with a stir bar, was charged with the tertiary alcohol (5.00 mmol, 1.50 equiv), NaI (1.50 g, 10.0 mmol, 2.00 equiv) and MeCN (25.0 mL) were added under nitrogen. The mixture was cooled to 0 °C and methylsulfonic acid (0.961 g, 10.0 mmol, 2.00 equiv) were added dropwise. The reaction mixture was allowed to warm to room temperature and stirred for 4 h. After the completion of the reaction, the reaction mixture was diluted with Et_2O (50.0 mL), washed with water, saturated aqueous $NaHCO_3$, $Na_2S_2O_3$, and brine, dried over Na_2SO_4 and filtrated. The solvent was removed by rotary evaporation and the residue was purified by flash silica gel chromatography to give the desired products.

Compound **5a**: brown oil (0.787 g, 2.87 mmol, 57.4% yield), compound **5c**: colorless liquid (1.18g, 3.61 mmol, 72.1% yield), compound **5g**: yellow liquid (0.667 g, 2.16 mmol, 43.3% yield), compound **5i**: colorless liquid (1.30g, 3.77 mmol, 75.3% yield), compound **5j**: white solid (1.00 g, 3.81 mmol, 76.3% yield). Spectra were consistent with literature data[3].

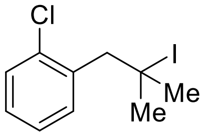

1-Chloro-2-(2-iodo-2-methylpropyl)benzene (5b): Prepared on 5.00 mmol according to **General Method D** (Eluent: PE) and the title compound was isolated as a colorless liquid (0.964 mg, 3.28 mmol, 65.6% yield). **IR** (thin film) 1474 (m), 1439 (m), 1368 (m), 1098 (s), 1089 (s), 1036 (m), 748 (m), 727 (m), 680 (m) cm^{-1}; **^{1}H NMR** (400 MHz, $CDCl_3$) δ 7.49 – 7.47 (1H, m), 7.39 – 7.37 (1H, m), 7.24 – 7.22 (2H, m), 3.47 (2H, s), 2.01 (6H, s); **^{13}C NMR** (101 MHz, $CDCl_3$) δ 135.5, 135.2, 132.9, 129.9, 128.5, 126.4, 51.9, 50.0, 37.8; **HRMS** (ESI) [M+H] calc'd for $C_{10}H_{13}ClI$: 294.9750, found: 294.9760.

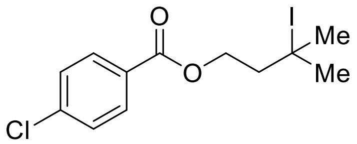

3-Iodo-3-methylbutyl 4-chlorobenzoate (5d): Prepared according to **General Method D** (Eluent: 100:1 to 10:1 petroleum ether: ethyl acetate) and the title compound was isolated as a light yellow oil (0.981 g, 2.78 mol, 55.6% yield). **IR** (thin film) 1717 (s), 1594 (m), 1401 (w), 1268 (s), 1171 (w), 1091 (s), 1015 (s), 849 (m), 757 (s), 684 (m) cm^{-1}; **^{1}H NMR** (400 MHz, $CDCl_3$) δ 7.97 (2H, d, J = 8.9 Hz), 7.41 (2H, d, J = 8.6 Hz), 4.56 (2H, t, J = 6.8 Hz), 2.16 (2H, t, J = 6.8 Hz), 2.02 (6H, s); **^{13}C NMR** (101 MHz, $CDCl_3$) δ 165.7, 139.6, 131.1, 128.9, 128.6, 65.5, 48.2, 46.0, 38.7; **HRMS** (ESI^+) $[M+Na]^+$ calc'd for $C_{12}H_{14}ClINaO_2$: 374.9619, found: 374.9620.

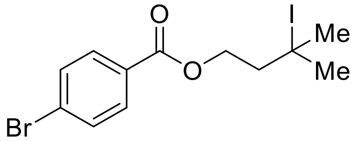

3-Iodo-3-methylbutyl 4-bromobenzoate (5e): Prepared according to **General Method D** (Eluent: 100:1 to 10:1 petroleum ether: ethyl acetate) and the title compound was isolated as a light yellow oil (1.02 g, 2.57 mmol, 51.4% yield). **IR** (thin film) 1717 (s), 1590 (m), 1397 (m), 1267 (s), 1172 (m), 1100 (s), 1068 (s), 1011 (s), 847 (m), 754 (s), 682 (m) cm^{-1}; **^{1}H NMR** (400 MHz, $CDCl_3$) δ 7.89 (2H, d, J = 8.6 Hz), 7.58 (2H, d, J = 8.6 Hz), 4.55 (2H, t, J = 6.8 Hz), 2.16 (2H, t, J = 6.8 Hz), 2.02 (6H, s); **^{13}C NMR** (101 MHz, $CDCl_3$) δ 165.7, 131.8, 131.1, 129.0,

128.2, 65.4, 48.1, 45.9, 38.5; **HRMS** (ESI$^+$) [M+Na]$^+$ calc'd for $C_{12}H_{14}BrINaO_2$: 418.9114, found: 418.9102.

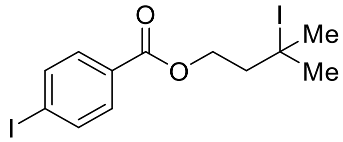

3-Iodo-3-methylbutyl 4-iodobenzoate (5f): Prepared according to **General Method D** (Eluent: 100:1 to 10:1 petroleum ether: ethyl acetate) and the title compound was isolated as a light yellow oil (1.38 g, 3.11 mol, 62.2% yield). **IR** (thin film) 1716 (s), 1585 (m), 1392 (m), 1266 (s), 1176 (m), 1099 (s), 1007 (s), 844 (m), 751 (s), 682 (m) cm^{-1}; **^{1}H NMR** (400 MHz, $CDCl_3$) δ 7.80 (2H, d, J = 8.5 Hz), 7.73 (2H, d, J = 8.5 Hz), 4.55 (2H, t, J = 6.8 Hz), 2.16 (2H, t, J = 6.8 Hz), 2.02 (6H, s); **^{13}C NMR** (101 MHz, $CDCl_3$) δ 166.1, 137.9, 131.2, 129.6, 101.0, 65.5, 48.2, 46.0, 38.7; **HRMS** (ESI$^+$) [M+Na]$^+$ calc'd for $C_{12}H_{14}I_2NaO_2$: 466.8975, found: 466.8972.

2.11 Synthesis of 4-ethyl-4-iodotetrahydro-2*H*-pyran 5k

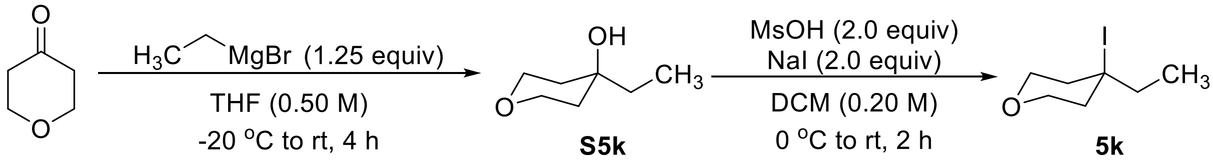

A 120 °C oven-dried 100-mL round-bottom flask, equipped with a stir bar, was charged with cyclobutanone (1.00 g, 10.0 mmol, 1.00 equiv) and THF (20.0 mL) were added under nitrogen. The mixture was cooled to –20 °C and newly prepared phenethylmagnesium bromide (12.5 mL, 12.5 mmol, 1.25 equiv) was added dropwise. The reaction mixture was warmed to room temperature and stirred for addtional 4 h. The reaction was quenched with saturated NH_4Cl aqueous solution (20.0 mL). H_2O (50.0 mL) was added and the mixture was extracted with Et_2O (50.0 mL × 3). The combined organic layers were washed with brine (10.0 mL), dried over Na_2SO_4 and filtrated. The solvent was removed by rotary evaporation and the residue was purified by flash silica gel chromatography ((Eluent: 50:1 to 5:1 petroleum ether: ethyl acetate)). The product was isolated as a colorless oil (0.669 g, 5.14 mmol, 51.4% yield).

A 120 °C oven-dried 100-mL round-bottom flask, equipped with a stir bar, was charged with 4-ethyltetrahydro-2*H*-pyran-4-ol (0.651 g, 5.00 mmol, 1.00 equiv), NaI (1.50 g, 10.0

mmol, 2.00 equiv) and MeCN (25.0 mL) were added under nitrogen. The mixture was cooled to 0 °C and methylsulfonic acid (0.961 g, 10.0 mmol, 2.00 equiv) was added dropwise. The reaction mixture was allowed to warm to room temperature and stirred for 2 h. Next, the reaction mixture was diluted with Et_2O (50.0 mL), washed with H_2O (20.0 mL), saturated aqueous $NaHCO_3$ (20.0 mL), $Na_2S_2O_3$ (10.0 mL), and brine (20.0 mL), dried over Na_2SO_4 and filtrated. The solvent was removed by rotary evaporation and the residue was purified by flash silica gel chromatography ((Eluent: 100:1 to 5:1 petroleum ether: ethyl acetate)). The product was isolated as colorless oil (0.516 g, 2.15 mmol, 43.0% yield). Spectra were consistent with literature data[3].

2.12 Synthesis of 3-iodo-1-tosyloctahydro-1H-indole 5l

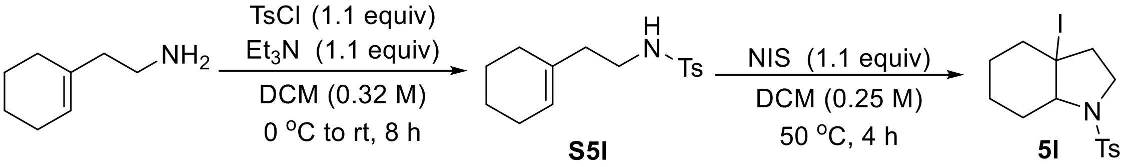

A 120 °C oven-dried 200-mL round-bottom flask, equipped with a stir bar, was charged with 2-cyclohex-1-enyl-ethylamine (2.50 g, 20.0 mmol, 1.00 equiv), Et_3N (2.23 g, 22.0 mmol, 1.10 equiv) and DCM (40.0 mL) were added under nitrogen. The mixture was cooled to 0 °C and 4-methyl-benzenesulfonyl chloride (4.19 g, 22.0 mmol, 1.10 equiv) was added dropwise. The reaction mixture was allowed to warm to room temperature and stirred for 8 h. After the completion of reaction, H_2O (50.0 mL) was added and the mixture was extracted with DCM (50.0 mL × 3). The combined organic layers were washed with brine (10.0 mL), dried over Na_2SO_4 and filtrated. The solvent was removed by rotary evaporation and the residue was purified by flash silica gel chromatography ((Eluent: 100:1 to 2:1 petroleum ether: ethyl acetate)). The product was isolated as a white solid (5.58 g, 20.0 mmol, 100% yield).

A 120 °C oven-dried 100-mL round-bottom flask, equipped with a stir bar, was charged with *N*-(2-cyclohex-1-enyl-ethyl)-4-methyl-benzenesulfonamide (1.40 g, 5.00 mmol, 1.00 equiv), NIS (1.24 g, 5.50 mmol, 1.10 equiv) and DCM (20.0 mL) was added under nitrogen. The mixture was allowed to stir at 50 °C for 4 h. After the completion of reaction, H_2O (50.0 mL) was added and the mixture was extracted with DCM (50.0 mL × 3). The

combined organic layers were washed with brine (10.0 mL), dried over Na_2SO_4 and filtrated. The solvent was removed by rotary evaporation and the residue was purified by flash silica gel chromatography ((Eluent: 100:1 to 10:1 petroleum ether: ethyl acetate)). The product was isolated as a white solid (1.03 g, 2.54 mmol, 50.8% yield). Spectra were consistent with literature data[3].

3. Optimization of the reaction conditions

3.1 Evaluation of different solvents

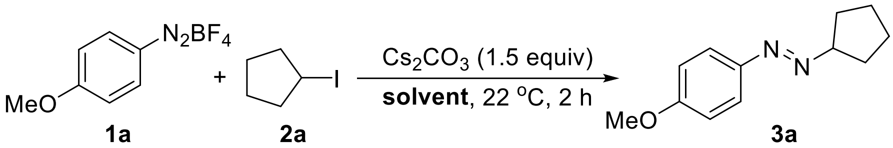

Entry	solvent	yield of **3a** (%)[a]
1	MeOH	96 (94)[b]
2	DCM	<5
3	DMF	56
4	DMA	<5
5	Ether	<5
6	DCE	<5
7	Tol	<5
8	EA	<5
9	1,4-dioxane	<5
10	MeCN	70

Reaction conditions: **1a** (0.3 mmol, 3.0 equiv), **2a** (0.1 mmol, 1.0 equiv), and Cs_2CO_3 (0.15 mmol, 1.5 equiv) in solvent (1.0 mL) at 20 ^{o}C for 2 h; [a]Yield was determined by ^{1}H NMR spectroscopy in the presence of CH_2Br_2 as an internal standard.[b]isolated yields.

Supplementary Fig. 2. Solvent effect

3.2 Evaluation of different base

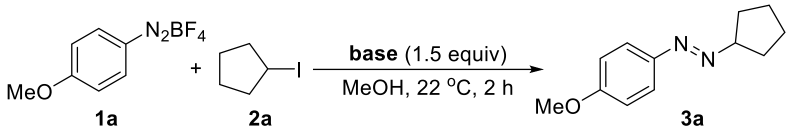

Entry	base	yield of **3a** (%)
1	no base	no reaction
2	K_2CO_3	90
3	Na_2CO_3	88
4	NaOH	72
5	$NaHCO_3$	<5
6	KOAc	<5
7	^{t}BuOK	<5
8	Et_3N	57
9	CsF	<5
10	DBU	47
11	K_3PO_4	14
12	Cs_2CO_3	96

Reaction conditions: **1a** (0.3 mmol, 3.0 equiv), **2a** (0.1 mmol, 1.0 equiv), and base (0.15 mmol, 1.5 equiv) in MeOH (1.0 mL) at 20 °C for 2 h; [a]Yield was determined by ^{1}H NMR spectroscopy in the presence of CH_2Br_2 as an internal standard.

Supplementary Fig. 3. Base effect

3.3 Evaluation of different reaction time

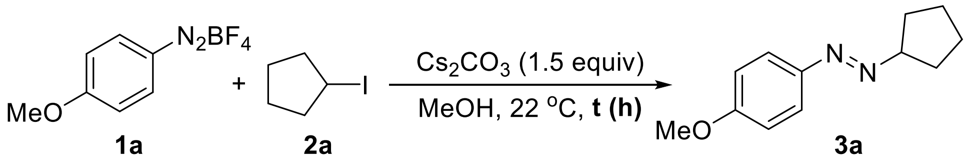

Entry	**t (h)**	yield of **3a** (%)[a]
1	1.0	92
2	2.0	96

Reaction conditions: **1a** (0.3 mmol, 3.0 equiv), **2a** (0.1 mmol, 1.0 equiv), and Cs_2CO_3 (0.15 mmol, 1.5 equiv) in MeOH (1.0 mL) at 20 °C for (t) h; [a]Yield was determined by ^{1}H NMR spectroscopy in the presence of CH_2Br_2 as an internal standard.

Supplementary Fig. 4. Time effect

3.4 Evaluation of equivalents of the arenediazonium tetrafluoroborates

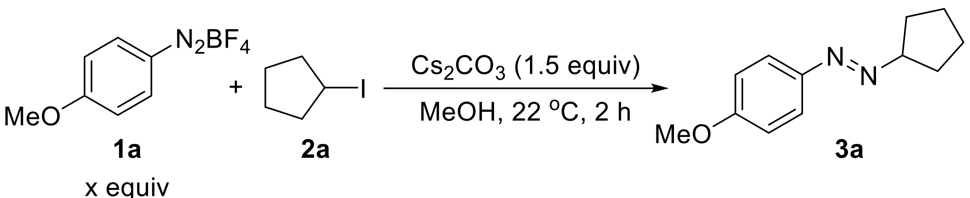

1a x equiv **2a** **3a**

Entry	x equiv	yield of **3a** (%)[a]
1	2.0	73
2	2.5	91
3	3.0	96

Reaction conditions: **1a** (x equiv), **2a** (0.1 mmol, 1.0 equiv), and Cs_2CO_3 (0.15 mmol, 1.5 equiv) in solvent (1.0 mL) at 20 °C for 2 h; [a]Yield was determined by ^1H NMR spectroscopy in the presence of CH_2Br_2 as an internal standard.

Supplementary Fig. 5. Equivalents of arenediazonium tetrafluoroborates effect

4. Substrate scope: General Method F.

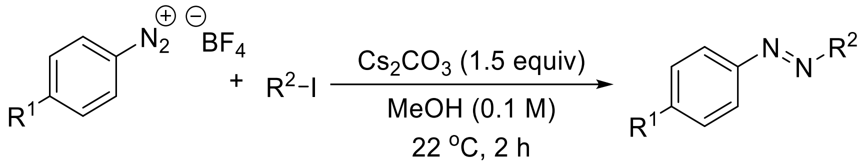

A 25-mL glass vial fitted with a stirring bar was charged with arenediazonium tetrafluoroborate (1.50 mmol, 3.00 equiv) and Cs_2CO_3 (244 mg, 0.750 mmol, 1.50 equiv). The mixture was evacuated and backfilled with nitrogen three times. Then the alkyl iodide (0.500 mmol, 1.00 equiv) and MeOH (5.00 mL) were added under nitrogen. The mixture was allowed to stir at 22 ºC for 2 h. After the completion of reaction, the reaction mixture was poured into H_2O (50.0 mL) and extracted with EtOAc (20.0 mL × 3). The combined organic layers were washed with H_2O (20.0 mL), dried over Na_2SO_4 and filtered. The solvent was removed by rotary evaporation and the residue was purified by flash silica gel chromatography. (Caution: The alkyl-aryl azo-compunds are not stable at DCM, $CDCl_3$ and needed to be handled with care

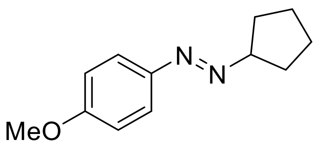

(*E*)-1-cyclopentyl-2-(4-methoxyphenyl)diazene (3a): Prepared according to **General Method F** (Eluent: petroleum ether) and the title compound was isolated as a light yellow oil

(38.6 mg, 0.189 mmol, 94.5% isolated yield, prepared on 0.200 mmol scale). **IR** (thin film) 2956 (w), 1603 (m), 1513 (m), 1246 (s), 1180 (m), 1143 (m), 1032 (m), 835 (m) cm^{-1}; **^{1}H NMR** (400 MHz, CD_3COCD_3) δ 7.66 (2H, d, J = 8.8 Hz), 7.02 (2H, d, J = 8.8 Hz), 4.15 – 4.09 (1H, m), 3.86 (3H, s), 1.96 – 1.86 (6H, m), 1.75 – 1.72 (2H, m); **^{13}C NMR** (101 MHz, CD_3COCD_3) δ 162.5, 147.0, 124.7, 114.9, 78.7, 56.0, 32.2, 26.0; **HRMS** (ESI$^+$) [M+H]$^+$ calc'd for $C_{12}H_{17}N_2O$: 205.1335, found: 205.1331.

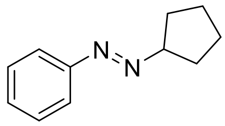

(*E*)-1-cyclopentyl-2-phenyldiazene (3b): Prepared according to **General Method F** (Eluent: 100:0 to 200:1 petroleum ether: ethyl acetate) and the title compound was isolated as a light yellow oil (26.3 mg, 0.151 mmol, 75.5% yield, prepared on 0.200 mmol scale, 6 h). **IR** (thin film) 2957 (w), 1452 (w), 764 (s), 689 (s) cm^{-1}; **^{1}H NMR** (400 MHz, CD_3COCD_3) δ 7.65 (2H, d, J = 8.3 Hz), 7.51 – 7.46 (3H, m), 4.21 – 4.15 (1H, m), 1.99 – 1.93 (4H, m), 1.92 – 1.88 (2H, m), 1.78 – 1.74 (2H, m); **^{13}C NMR** (101 MHz, CD_3COCD_3) δ 153.1, 131.2, 130.0, 122.9, 79.2, 32.2, 26.1; **HRMS** (ESI$^+$) [M+H]$^+$ calc'd for $C_{11}H_{15}N_2$: 175.1230, found: 175.1238.

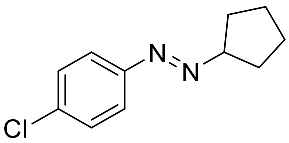

(*E*)-1-(4-chlorophenyl)-2-cyclopentyldiazene (3c): Prepared according to **General Method F** (Eluent: 100:0 to 150:1 petroleum ether: ethyl acetate) and the title compound was isolated as a light yellow oil (94.7 mg, 0.454 mmol, 90.8% yield). **IR** (thin film) 2958 (m), 1475 (m), 1402 (w), 1087 (s), 1011 (m), 833 (s) cm^{-1}; **^{1}H NMR** (400 MHz, CD_3COCD_3) δ 7.67 (2H, d, J = 8.7 Hz), 7.52 (2H, d, J = 8.7 Hz), 4.22 – 4.16 (1H, m), 1.98 – 1.91 (4H, m), 1.90 – 1.84 (2H, m), 1.77 – 1.73 (2H, m); **^{13}C NMR** (101 MHz, CD_3COCD_3) δ 151.5, 136.5, 130.1, 124.5, 79.3, 32.2, 26.0; **HRMS** (ESI$^+$) [M+H]$^+$ calc'd for $C_{11}H_{14}ClN_2$: 209.0840, found: 209.0850.

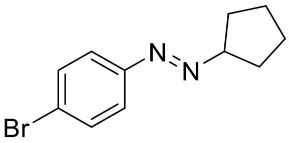

(*E*)-1-(4-bromophenyl)-2-cyclopentyldiazene (3d): Prepared according to **General Method F** (Eluent: 100:0 to 200:1 petroleum ether: ethyl acetate) and the title compound was isolated

as a light yellow oil (87.8 mg, 0.347 mmol, 69.4% yield). **IR** (thin film) 2957 (s), 1579 (w), 1471 (m), 1398 (w), 1067 (s), 1009 (s), 831 (s) cm^{-1}; **^{1}H NMR** (400 MHz, CD_3COCD_3) δ 7.68 (2H, d, J = 8.8 Hz), 7.60 (2H, d, J = 8.8 Hz), 4.22 – 4.16 (1H, m), 1.99 – 1.93 (4H, m), 1.92 – 1.84 (2H, m), 1.77 – 1.73 (2H, m); **^{13}C NMR** (101 MHz, CD_3COCD_3) δ 151.9, 133.2, 124.9, 79.4, 32.2, 26.1; **HRMS** (ESI$^+$) $[M+H]^+$ calc'd for $C_{11}H_{14}BrN_2$: 253.0335, found: 253.0342.

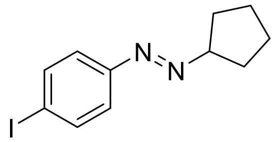

(*E*)-1-cyclopentyl-2-(4-iodophenyl)diazene (3e): Prepared according to **General Method F** (Eluent: petroleum ether) and the title compound was isolated as a light yellow oil (131.6 mg, 0.438 mmol, 87.7% yield). **IR** (thin film) 2955 (m), 1467 (m), 1392 (w), 1052 (w), 1005 (s), 826 (s), 690 (w) cm^{-1}; **^{1}H NMR** (400 MHz, CD_3COCD_3) δ 7.88 (2H, d, J = 8.6 Hz), 7.45 (2H, d, J = 8.6 Hz), 4.21 – 4.15 (1H, m), 1.98 – 1.93 (4H, m), 1.91 – 1.85 (2H, m), 1.78 – 1.73 (2H, m); **^{13}C NMR** (101 MHz, CD_3COCD_3) δ 152.4, 139.2, 124.9, 97.0, 79.3, 32.1, 26.0; **HRMS** (ESI$^+$) $[M+H]^+$ calc'd for $C_{11}H_{14}IN_2$: 301.0196, found: 301.0203.

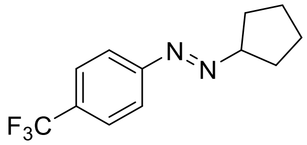

(*E*)-1-cyclopentyl-2-(4-(trifluoromethyl)phenyl)diazene (3f): Prepared according to **General Method F** (Eluent: 100:0 to 100:1 petroleum ether: ethyl acetate) and the title compound was isolated as a light yellow oil (70.8 mg, 0.292 mmol, 58.5% yield, 6 h). **IR** (thin film) 1963 (w), 1322 (s), 1166 (m), 1126 (s), 1065 (s), 849 (w) cm^{-1}; **^{1}H NMR** (400 MHz, CD_3COCD_3) δ 7.84 (4H, dd, J = 17.6, 8.6 Hz), 4.29 – 4.22 (1H, m), 2.02 – 1.89 (6H, m), 1.80 – 1.76 (2H, m); **^{19}F NMR** (471 MHz, CD_3COCD_3) δ –63.0 (3F, s); **^{13}C NMR** (101 MHz, CD_3COCD_3) δ 155.2, 132.0 (q, J = 32.3 Hz), 127.2 (q, J = 3.9 Hz), 125.1 (q, J = 272.5 Hz), 123.5, 79.7, 32.1, 26.0; **HRMS** (ESI$^+$) $[M+H]^+$ calc'd for $C_{12}H_{14}F_3N_2$: 243.1104, found: 243.1097.

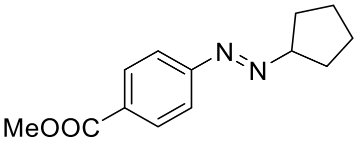

methyl (*E*)-4-(cyclopentyldiazenyl)benzoate (3g): Prepared according to **General Method F**

(Eluent: 100:0 to 100:1 petroleum ether: ethyl acetate) and the title compound was isolated as a light yellow oil (45.1 mg, 0.194 mmol, 97.1% yield, prepared on 0.200 mmol scale). **IR** (thin film) 2953 (w), 1722 (s), 1435 (m), 1272 (s), 1191 (w), 1109 (s), 1014 (w), 863 (w), 772 (m), 697 (w) cm^{-1}; **^{1}H NMR** (400 MHz, CD_3COCD_3) δ 8.13 (2H, d, J =8.4 Hz), 7.73 (2H, d, J = 8.4 Hz), 4.27 – 4.20 (1H, m), 3.92 (3H, s), 2.02 – 1.97 (4H, m), 1.94 – 1.91 (2H, m), 1.82 – 1.75 (2H, m); **^{13}C NMR** (101 MHz, CD_3COCD_3) δ 167.2, 156.1, 132.9, 131.8, 123.4, 80.2, 53.1, 32.7, 26.6; **HRMS** (ESI^+) $[M+H]^+$ calc'd for $C_{13}H_{17}N_2O_2$: 233.1285, found: 233.1282.

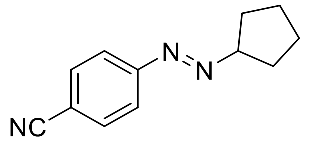

(*E*)-4-(cyclopentyldiazenyl)benzonitrile (3h): Prepared according to **General Method F** (Eluent: 100:0 to 200:1 petroleum ether: ethyl acetate) and the title compound was isolated as a light yellow oil (29.6 mg, 0.149 mmol, 74.3% yield, prepared on 0.200 mmol scale). **IR** (thin film) 2958 (m), 2230 (m), 1489 (w), 846 (s) cm^{-1}; **^{1}H NMR** (400 MHz, CD_3COCD_3) δ 7.93 (2H, d, J = 8.6 Hz), 7.79 (2H, d, J = 8.6 Hz), 4.29 – 4.23 (1H, m), 2.02 – 1.97 (4H, m), 1.94 – 1.89 (2H, m), 1.82 – 1.74 (2H, m); **^{13}C NMR** (101 MHz, CD_3COCD_3) δ 155.6, 134.8, 124.2, 119.4, 114.9, 80.3, 32.7, 26.5; **HRMS** (ESI^+) $[M+H]^+$ calc'd for $C_{12}H_{14}N_3$: 200.1182, found: 200.1182.

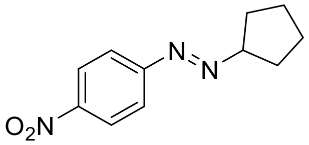

(*E*)-1-cyclopentyl-2-(4-nitrophenyl)diazene (3i): Prepared according to **General Method F** (Eluent: 100:0 to 100:1 petroleum ether: ethyl acetate) and the title compound was isolated as a light yellow oil (58.5 mg, 0.267 mmol, 53.4% yield). **IR** (thin film) 2957 (w), 1525 (s), 1343 (s), 1007 (w), 858 (s), 753 (m), 689 (m) cm^{-1}; **^{1}H NMR** (400 MHz, CD_3COCD_3) δ 8.37 (2H, d, J = 8.9 Hz), 7.84 (2H, d, J = 8.9 Hz), 4.30 – 4.24 (1H, m), 2.03 – 1.98 (4H, m), 1.95 – 1.89 (2H, m), 1.80 – 1.74 (2H, m); **^{13}C NMR** (101 MHz, CD_3COCD_3) δ 156.3, 149.5, 125.6, 123.8, 80.1, 32.2, 26.1; **HRMS** (ESI^+) $[M+H]^+$ calc'd for $C_{11}H_{14}N_3O_2$: 220.1081, found: 220.1086.

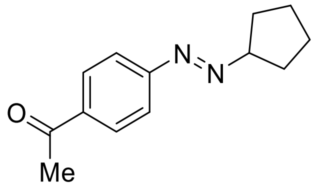

(*E*)-1-(4-(cyclopentyldiazenyl)phenyl)ethan-1-one (3j): Prepared according to **General Method F** (Eluent: petroleum ether) and the title compound was isolated as a light yellow oil (29.1 mg, 0.135 mmol, 67.3% yield, prepared on 0.200 mmol scale). **IR** (thin film) 2957 (w), 1684 (s), 1600 (w), 1406 (w), 1357 (w), 1261 (s), 957 (w), 845 (m) cm^{-1}; **^{1}H NMR** (400 MHz, CD_3COCD_3) δ 8.11 (2H, d, J = 8.6 Hz), 7.73 (2H, d, J = 8.6 Hz), 4.27 – 4.20 (1H, m), 2.63 (3H, s), 2.02 – 1.96 (4H, m), 1.94 – 1.89 (2H, m), 1.82 – 1.74 (2H, m); **^{13}C NMR** (101 MHz, CD_3COCD_3) δ 197.9, 155.9, 139.6, 130.7, 123.4, 80.2, 32.6, 27.4, 26.5; **HRMS** (ESI^+) $[M+H]^+$ calc'd for $C_{13}H_{17}N_2O$: 217.1335, found: 217.1330.

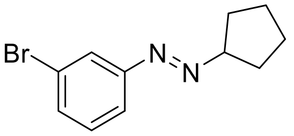

(*E*)-1-(3-bromophenyl)-2-cyclopentyldiazene (3k): Prepared according to **General Method F** (Eluent: petroleum ether) and the title compound was isolated as a light yellow oil (33.7 mg, 0.133 mmol, 66.6% yield, prepared on 0.2 mmol scale, 6 h). **IR** (thin film) 2958 (m), 1576 (m), 1463 (m), 1060 (w), 884 (m), 785 (s), 681 (s) cm^{-1}; **^{1}H NMR** (400 MHz, CD_3COCD_3) δ 7.76 – 7.75 (1H, m), 7.70 – 7.64 (2H, m), 7.50 – 7.47 (1H, m), 4.24 – 4.18 (1H, m), 2.00 – 1.95 (4H, m), 1.93 – 1.88 (2H, m), 1.80 – 1.74 (2H, m); **^{13}C NMR** (101 MHz, CD_3COCD_3) δ 154.1, 133.9, 131.9, 124.5, 123.4, 123.4, 79.4, 32.2, 26.1; **HRMS** (ESI^+) $[M+H]^+$ calc'd for $C_{11}H_{14}BrN_2$: 253.0335, found: 253.0325.

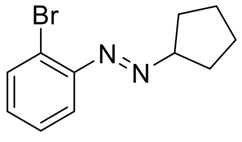

(*E*)-1-(2-bromophenyl)-2-cyclopentyldiazene (3l): Prepared according to **General Method F** ((Eluent: 100:0 to 200:1 petroleum ether: ethyl acetate) and the title compound was isolated as a light yellow oil (99.0 mg, 0.391 mmol, 78.2% yield). **IR** (thin film) 2956 (m), 1461 (m),

1028 (m), 755 (s) cm^{-1}; **^{1}H NMR** (400 MHz, CD_3COCD_3) *δ* 7.76 (1H, dd, *J* = 7.8, 1.3 Hz), 7.46 – 7.42 (1H, m), 7.41 – 7.36 (1H, m), 7.36 – 7.33 (1H, m), 4.35 – 4.29 (1H, m), 2.02 – 1.94 (4H, m), 1.92 – 1.85 (2H, m), 1.82 – 1.73 (2H, m); **^{13}C NMR** (101 MHz, CD_3COCD_3) *δ* 150.8, 134.8, 132.8, 129.8, 124.0, 119.9, 80.0, 32.6, 26.5; **HRMS** (ESI^+) $[M+H]^+$ calc'd for $C_{11}H_{14}BrN_2$: 253.0335, found: 253.0328.

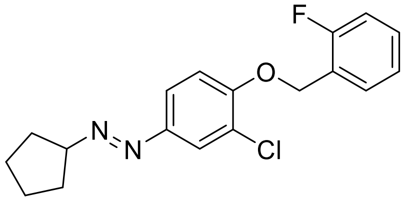

ethyl (*E*)-1-(3-chloro-4-((2-fluorobenzyl)oxy)phenyl)-2-cyclopentyldiazene (3m): Prepared according to **General Method F** (Eluent: 100:1 to 50:1 petroleum ether: ethyl acetate) and the title compound was isolated as a light yellow solid (138.3 mg, 0.416 mmol, 83.1% yield). **M.p.** = 34.8 – 35.4 °C; **IR** (thin film) 2958 (w), 1593 (m), 1488 (m), 1451 (m), 1382 (m), 1247 (s), 1139 (m), 1051 (s), 868 (m), 813 (m), 778 (s), 748 (m), 682 (m) cm^{-1}; **^{1}H NMR** (400 MHz, CD_3COCD_3) *δ* 7.73 (1H, d, *J* = 2.3 Hz), 7.66 (1H, dd, *J* = 8.6, 2.4 Hz), 7.47 – 7.42 (1H, m), 7.36 – 7.30 (2H, m), 7.28 (1H, d, *J* = 8.8 Hz), 7.13 – 7.08 (1H, m), 5.31 (2H, s), 4.18 – 4.12 (1H, m), 1.97 – 1.92 (4H, m), 1.90 – 1.83 (2H, m), 1.76 – 1.71 (2H, m); **^{19}F NMR** (471 MHz, CD_3COCD_3) *δ* –114.2 (1F, s); **^{13}C NMR** (101 MHz, CD_3COCD_3) *δ* 163.8 (d, *J* = 249.4 Hz), 156.3, 147.1, 140.3 (d, *J* = 7.7 Hz), 131.3 (d, *J* = 8.4 Hz), 124.9, 124.1, 123.8 (d, *J* = 2.8 Hz), 123.0, 115.5 (d, *J* = 21.3 Hz), 114.8 (d, *J* = 22.6 Hz), 114.5, 78.9, 70.6, 32.2, 26.0; **HRMS** (ESI^+) $[M+H]^+$ calc'd for $C_{18}H_{19}ClFN_2O$: 333.1164, found: 333.1175.

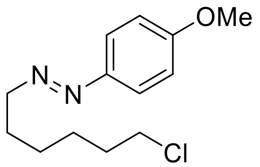

(*E*)-1-(6-chlorohexyl)-2-(4-methoxyphenyl)diazene (4a): Prepared according to **General Method F** (Eluent: 100:0 to 100:1 petroleum ether: ethyl acetate) and the title compound was isolated as a light yellow oil (79.9 mg, 0.314 mmol, 62.7% yield). **IR** (thin film) 2936 (w), 1603 (m), 1517 (m), 1463 (w), 1247 (s), 1142 (m), 1031 (m), 836 (s), 727 (w), 648 (w) cm^{-1}; **^{1}H NMR** (400 MHz, CD_3COCD_3) *δ* 7.67 (2H, d, *J* = 9.0 Hz), 7.02 (2H, d, *J* = 9.0 Hz), 3.98

(2H, t, J = 7.1 Hz), 3.86 (3H, s), 3.60 (2H, t, J = 6.7 Hz), 1.92 – 1.85 (2H, m), 1.82 – 1.75 (2H, m), 1.54 – 1.44 (4H, m); **^{13}C NMR** (101 MHz, CD_3COCD_3) δ 162.6, 147.1, 124.7, 114.9, 69.5, 56.0, 45.8, 33.3, 28.6, 27.5, 27.4; **HRMS** (ESI$^+$) [M+H]$^+$ calc'd for $C_{13}H_{20}ClN_2O$: 255.1259, found: 255.1274.

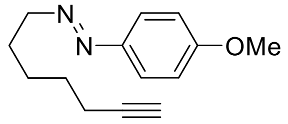

(*E*)-1-(hept-6-yn-1-yl)-2-(4-methoxyphenyl)diazene (4b): Prepared according to **General Method F** (Eluent: 100:0 to 100:1 petroleum ether: ethyl acetate) and the title compound was isolated as a yellow liquid (64.2 mg, 0.279 mmol, 55.8% yield). **IR** (thin film) 3060 (w), 2838 (w), 2367 (w), 1602 (m), 1517 (m), 1248 (s), 1143 (m), 1030 (m), 836 (s), 635 (m) cm^{-1}; **^{1}H NMR** (400 MHz, CD_3COCD_3) δ 7.67 (2H, d, J = 8.8 Hz), 7.04 (2H, d, J = 8.8 Hz), 5.01 (2H, t, J = 4.7 Hz), 3.99 (2H, t, J = 7.2 Hz), 3.88 (3H, s), 2.33 (1H, t, J = 2.7 Hz), 2.24 – 2.20 (2H, m), 1.99 – 1.86 (2H, m), 1.92 – 1.52 (4H, m). **^{13}C NMR** (101 MHz, CD_3COCD_3) δ 162.6, 147.1, 124.7, 114.9, 84.8, 70.0, 69.5, 55.9, 29.2, 28.2, 27.4, 18.7. HRMS (ESI$^+$) [M+H]$^+$ calc'd for $C_{14}H_{18}N_2ONa$: 253.1311, found: 253.1327.

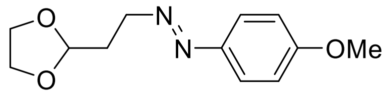

(*E*)-1-(2-(1,3-dioxolan-2-yl)ethyl)-2-(4-methoxyphenyl)diazene (4c): Prepared according to **General Method F** (Eluent: 100:0 to 100:1 petroleum ether: ethyl acetate) and the title compound was isolated as a yellow liquid (81.7 mg, 0.346 mmol, 69.2% yield). **IR** (thin film) 2884 (w), 1604 (m), 1517 (m), 1247 (s), 1137 (s), 1028 (s), 943 (s), 836 (s) cm^{-1}; **^{1}H NMR** (400 MHz, CD_3COCD_3) δ 7.67 (2H, d, J = 8.8 Hz), 7.03 (2H, d, J = 8.8 Hz), 5.01 (2H, t, J = 4.7 Hz), 4.09 (2H, t, J = 7.4 Hz), 3.96 – 3.92 (2H, m), 3.86 (3H, s), 3.85 – 3.81 (2H, m), 2.20 – 2.15 (2H, m). **^{13}C NMR** (101 MHz, CD_3COCD_3) δ 163.3, 147.7, 125.4, 115.6, 104.0, 66.2, 65.4, 56.6, 33.7. HRMS (ESI$^+$) [M+H]$^+$ calc'd for $C_{12}H_{17}N_2O_3$: 237.1234, found: 237.1225.

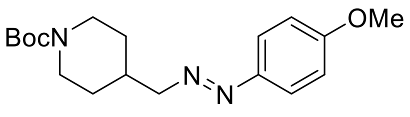

***tert*-Butyl (*E*)-4-(((4-methoxyphenyl)diazenyl)methyl)piperidine-1-carboxylate (4d):** Prepared according to **General Method F** (Eluent: 200:1 to 5:1 petroleum ether: ethyl acetate)

and the title compound was isolated as a light yellow oil (114.8 mg, 0.344 mmol, 68.9% yield). **IR** (thin film) 2929 (w), 1688 (s), 1603 (w), 1517 (w), 1420 (m), 1365 (m), 1245 (s), 1148 (s), 1031 (w), 838 (m), 769 (w) cm^{-1}; **^{1}H NMR** (400 MHz, CD_3COCD_3) δ 7.67 (2H, d, J = 9.0 Hz), 7.03 (2H, d, J = 9.0 Hz), 4.09 (2H, br), 3.87 – 3.86 (5H, m), 2.76 (2H, br), 2.23 – 2.15 (1H, m), 1.77 (2H, d, J = 12.7 Hz), 1.43 (9H, s), 1.32 – 1.22 (2H, m); **^{13}C NMR** (101 MHz, CD_3COCD_3) δ 162.7, 155.1, 147.1, 124.8, 115.0, 79.3, 75.1, 56.0, 44.9, 36.7, 31.4, 28.6; **HRMS** (ESI^+) $[M+Na]^+$ calc'd for $C_{18}H_{27}N_3NaO_3$: 356.1945, found: 356.1974.

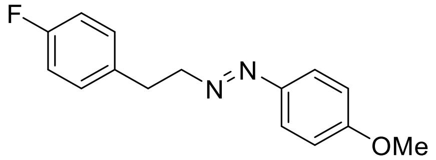

(*E*)-1-(4-fluorophenethyl)-2-(4-methoxyphenyl)diazene (4e): Prepared according to **General Method F** (Eluent: petroleum ether) and the title compound was isolated as a light yellow solid (102.4 mg, 0.396 mmol, 79.3% yield). **M.p.** = 49.8 – 50.7 °C; **IR** (thin film) 2934 (w), 1603 (m), 1516 (m), 1248 (s), 1147 (m), 1030 (m), 835 (m) cm^{-1}; **^{1}H NMR** (400 MHz, CD_3OD) δ 7.59 (2H, d, J = 9.0 Hz), 7.23 – 7.19 (2H, m), 6.97 – 6.92 (4H, m), 4.18 (2H, t, J = 7.4 Hz), 3.80 (3H, s), 3.15 (2H, t, J = 7.3 Hz); **^{19}F NMR** (471 MHz, CD_3OD) δ – 118.8 (1F, s); **^{13}C NMR** (101 MHz, CD_3OD) δ 163.3, 162.9 (d, J = 243.4 Hz), 147.4, 136.8, 131.6 (d, J = 8.0 Hz), 125.0, 116.0 (d, J = 21.5 Hz), 115.1, 71.1, 56.0, 34.5; **HRMS** (ESI^+) $[M+H]^+$ calc'd for $C_{15}H_{15}FN_2O$: 259.1241, found: 259.1241.

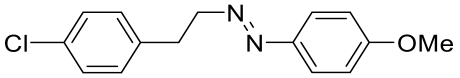

(*E*)-1-(4-chlorophenethyl)-2-(4-methoxyphenyl)diazene (4f): Prepared according to **General Method F** (Eluent: 100:0 to 100:1 petroleum ether: ethyl acetate) and the title compound was isolated as a yellow solid (96.1 mg, 0.350 mmol, 69.9% yield). **M.p.** = 65.2 – 66.3 °C; **IR** (thin film) 1600 (w), 1582 (w), 1512 (m), 1488 (w), 1294 (s), 1141 (s), 1028 (s), 943 (s), 836 (s), 636 (w) cm^{-1}; **^{1}H NMR** (400 MHz, CD_3COCD_3) δ 7.66 (2H, d, J = 8.9 Hz), 7.34 – 7.29 (4H, m), 7.02 (2H, d, J = 8.8 Hz), 4.24 (2H, t, J = 7.3 Hz), 3.86 (3H, s), 4.09 (2H, t, J = 7.3 Hz). **^{13}C NMR** (101 MHz, CD_3COCD_3) δ 163.4, 147.7, 140.4, 133.0, 132.3, 129.9, 125.4, 115.6, 71.1, 56.7, 34.9. HRMS (ESI^+) $[M+Na]^+$ calc'd for $C_{15}H_{15}N_2ClNaO$: 297.0782, found: 297.0765.

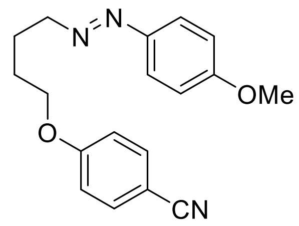

(*E*)-4-(4-((4-methoxyphenyl)diazenyl)butoxy)benzonitrile (4g): Prepared according to **General Method F** (Eluent: 300:1 to 200:1 petroleum ether: ethyl acetate) and the title compound was isolated as a light yellow solid (112.3 mg, 0.363 mmol, 72.6% yield). **M.p.** = 58.9 – 59.8 °C; **IR** (thin film) 2223 (w), 1603 (m), 1510 (m), 1300 (s), 1262 (m), 1171 (m), 1152 (m), 1027 (m), 998 (m), 839 (s), 715 (w) cm^{-1}; **^{1}H NMR** (400 MHz, CD_3COCD_3) δ 7.67 (2H, d, J = 8.8 Hz), 7.67 (2H, d, J = 8.8 Hz), 7.10 – 7.07 (2H, m), 7.02 (2H, d, J = 8.9 Hz), 4.18 – 4.14 (2H, m), 4.05 (2H, t, J = 6.9 Hz), 3.86 (3H, s), 2.10 – 2.06 (2H, m), 1.98 – 1.91 (2H, m); **^{13}C NMR** (101 MHz, CD_3COCD_3) δ 163.4, 162.6, 147.0, 134.8, 124.7, 119.7, 116.3, 114.9, 104.5, 69.1, 68.8, 55.9, 27.7, 25.2; **HRMS** (ESI^{+}) $[M+Na]^{+}$ calc'd for $C_{18}H_{19}N_3NaO_2$: 332.1369, found: 332.1352.

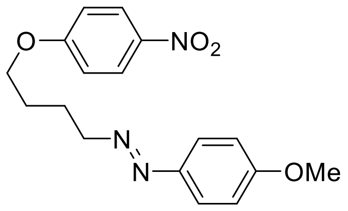

(*E*)-(4-methoxyphenyl)-2-(4-(4-nitrophenoxy)butyl)diazene (4h): Prepared according to **General Method F** (Eluent: 100:0 to 100:1 petroleum ether: ethyl acetate) and the title compound was isolated as a yellow solid (102.3 mg, 0.311 mmol, 62.1% yield). M.p. = 42.3 – 43.8 °C; **IR** (thin film) 2930 (w), 1588 (m), 1497 (m), 1470 (m), 1331 (s), 1247 (s), 1110 (m), 1026 (m), 840 (s), 691 (m), 658 (m) cm^{-1}; **^{1}H NMR** (400 MHz, CD_3COCD_3) δ 8.20 (2H, d, J = 9.2 Hz), 7.67 (2H, d, J = 8.9 Hz), 7.12 (2H, d, J = 9.2 Hz), 7.02 (2H, d, J = 8.9 Hz), 4.24 (2H, t, J = 6.4 Hz), 4.07 (2H, t, J = 6.8 Hz), 3.86 (3H, s), 2.12 – 2.06 (2H, m), 2.02 – 1.94 (2H, m). **^{13}C NMR** (101 MHz, CD_3COCD_3) δ 165.9, 163.3, 147.7, 142.9, 127.3, 125.4, 116.3, 115.6, 70.05, 69.8, 56.6, 28.4, 25.9. HRMS (ESI^{+}) $[M+Na]^{+}$ calc'd for $C_{17}H_{19}N_3NaO_4$: 352.1268, found: 352.1276.

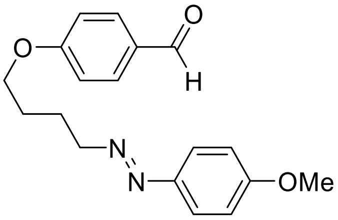

(*E*)-4-(4-((4-methoxyphenyl)diazenyl)butoxy)benzaldehyde (4i): Prepared according to **General Method F** (Eluent: 100:0 to 10:1 petroleum ether: ethyl acetate) and the title compound was isolated as a yellow liquid (92.7 mg, 0.2976 mmol, 59.4% yield). **IR** (thin film) 2920 (w), 1686 (m), 1599 (s), 1509 (m), 1247 (s), 1158 (m), 1028 (m), 831 (s) cm^{-1}; ^{1}**H NMR** (400 MHz, CD_3COCD_3) δ 9.90 (1H, s), 7.86 (2H, d, *J* = 8.7 Hz), 7.68 (2H, d, *J* = 9.0 Hz), 7.11 (2H, d, *J* = 8.7 Hz), 7.03 (2H, d, *J* = 9.0 Hz), 4.19 (2H, t, *J* = 6.4 Hz), 4.06 (2H, t, *J* = 6.8 Hz), 3.86 (3H, s), 2.12 – 2.05 (2H, m), 2.00 – 1.93 (2H, m). ^{13}**C NMR** (101 MHz, CD_3COCD_3) δ 191.9, 165.6, 163.3, 147.7, 133.2, 131.7, 125.4, 116.4, 115.6, 69.8, 69.5, 56.6, 28.5, 26.0. HRMS (ESI^+) $[M+K]^+$ calc'd for $C_{18}H_{20}N_2KO_2$: 335.1156, found: 335.1160.

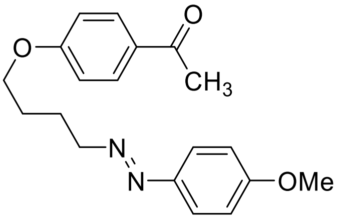

(*E*)-1-(4-(4-((4-methoxyphenyl)diazenyl)butoxy)phenyl)ethan-1-one (4j): Prepared according to **General Method F** (Eluent: 100:0 to 100:1 petroleum ether: ethyl acetate) and the title compound was isolated as a yellow liquid (90.0 mg, 0.276 mmol, 55.2% yield). **IR** (thin film) 1673 (m), 1599 (s), 1510 (m), 1358 (w), 1245 (s), 1170 (m), 1028 (m), 833 (s) cm^{-1}; ^{1}**H NMR** (400 MHz, CD_3COCD_3) δ 7.95 (2H, d, *J* = 8.9 Hz), 7.68 (2H, d, *J* = 8.9 Hz), 7.05 – 6.98 (4H, m), 4.18 – 14 (2H, m), 4.06 (2H, t, *J* = 7.0 Hz), 3.86 (3H, s), 2.51 (3H, s), 2.06 – 2.04 (2H, m), 1.99 – 1.91 (2H, m). ^{13}**C NMR** (101 MHz, CD_3COCD_3) δ 197.1, 164.5, 163.3, 147.8, 132.0, 131.9, 125.4, 115.7, 115.6, 69.9, 69.3, 56.7, 28.6, 27.1, 26.0. HRMS (ESI^+) $[M+Na]^+$ calc'd for $C_{19}H_{22}N_2NaO_3$: 349.1537, found: 349.1523.

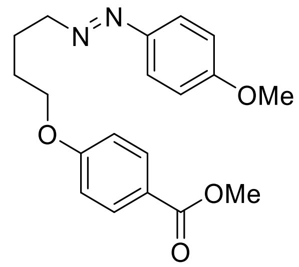

methyl (*E*)-4-(4-((4-methoxyphenyl)diazenyl)butoxy)benzoate (4k): Prepared according to **General Method F** (Eluent: 100:1 to 3:1 petroleum ether: ethyl acetate) and the title compound was isolated as a light yellow solid (121.7 mg, 0.355 mmol, 71.1% yield). **M.p.** = 42.4 –43.3 °C; **IR** (thin film) 1713 (m), 1604 (m), 1511 (w), 1434 (w), 1246 (s), 1167 (s), 1030 (m), 1029 (m), 838 (m), 769 (m), 696 (w), 649 (w) cm^{-1}; **^{1}H NMR** (400 MHz, CD_3COCD_3) δ 7.94 (2H, d, J = 9.0 Hz), 7.68 (2H, d, J = 9.0 Hz), 7.03 – 7.00 (4H, m), 4.14 (2H, t, J = 6.4 Hz), 4.05 (2H, t, J = 6.9 Hz), 3.85 (3H, s), 3.83 (3H, s), 2.11 – 2.06 (2H, m), 1.98 – 1.91 (2H, m); **^{13}C NMR** (101 MHz, CD_3COCD_3) δ 166.9, 163.9, 162.6, 147.1, 132.2, 124.7, 123.3, 115.1, 114.9, 69.2, 68.6, 56.0, 52.0, 27.9, 25.3; **HRMS** (ESI^+) $[M+H]^+$ calc'd for $C_{19}H_{22}N_2NaO_4$: 365.1472, found: 365.1478.

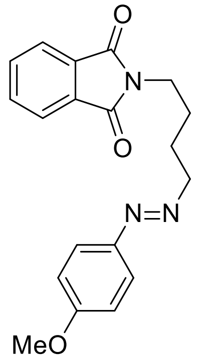

(*E*)-2-(4-((4-methoxyphenyl)diazenyl)butyl)isoindoline-1,3-dione (4l): Prepared according to **General Method F** (Eluent: 100:0 to 100:1 petroleum ether: ethyl acetate) and the title compound was isolated as a yellow solid (124.9 mg, 0.370 mmol, 74.1% yield). **M.p.** = 66.9 – 68.0 °C. **IR** (thin film) 1768 (w), 1706 (s), 1603 (w), 1583 (w), 1515 (m), 1467 (m), 1248 (m), 1133 (m), 1052 (m), 832 (s), 717 (s) cm^{-1}; **^{1}H NMR** (400 MHz, CD_3COCD_3) δ 7.85 – 7.81 (4H, m), 7.64 (2H, d, J = 8.9 Hz), 7.01 (2H, d, J = 8.9 Hz), 4.02 (2H, t, J = 6.8 Hz), 3.86 (3H, s), 3.74 (2H, t, J = 7.0 Hz), 1.99 – 1.91 (2H, m), 1.86 – 1.79 (2H, m). **^{13}C NMR** (101 MHz, CD_3COCD_3) δ 169.5, 163.3, 147.7, 135.6, 133.8, 125.4, 124.4, 115.6, 69.7, 56.6, 39.0, 28.0, 26.8. HRMS (ESI^+) $[M+Na]^+$ calc'd for $C_{19}H_{19}N_3NaO_3$: 360.1330, found: 360.1319.

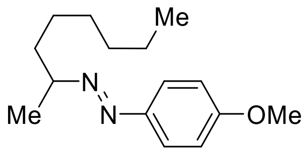

(*E*)-1-(4-methoxyphenyl)-2-(octan-2-yl)diazene (4m): Prepared according to **General Method F** (Eluent: 100:0 to 100:1 petroleum ether: ethyl acetate) and the title compound was isolated as a yellow oil (105.7 mg, 0.426 mmol, 85.2% yield). **IR** (thin film) 2929 (w), 1603 (m), 1517 (m), 1462 (w), 1248 (s), 1144 (m), 1034 (m), 836 (s) cm^{-1}; **^{1}H NMR** (400 MHz, CD_3COCD_3) δ 7.67 (2H, d, J = 7.2 Hz), 7.02 (2H, d, J = 7.2 Hz), 3.87 (3H, s), 3.46 – 3.40 (1H, m), 1.92 – 1.86 (2H, m), 1.80 – 1.68 (2H, m), 1.34 – 1.15 (6H, m), 0.96 – 0.81 (6H, m). **^{13}C NMR** (101 MHz, CD_3COCD_3) δ 163.2, 147.8, 125.4, 115.6, 80.3, 56.7, 34.8, 33.3, 28.1, 27.3, 24.0, 15.1, 15.0, 11.8, 11.7. HRMS (ESI$^+$) [M+Na]$^+$ calc'd for $C_{15}H_{24}N_2NaO$: 271.1779, found: 271.1781.

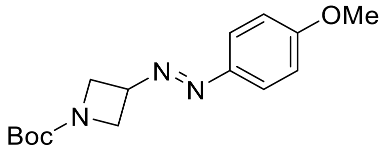

***tert*-Butyl *(E)*-3-((4-methoxyphenyl)diazenyl)azetidine-1-carboxylate (4n):** Prepared according to **General Method F** (Eluent: 100:0 to 10:1 petroleum ether: ethyl acetate) and the title compound was isolated as a light yellow oil (62.6 mg, 0.215 mmol, 43.0% yield). **IR** (thin film) 2976 (m), 1697 (s), 1603 (m), 1510 (m), 1391 (s), 1249 (m), 1137 (s), 1029 (m), 837 (s), 771 (m) cm^{-1}; **^{1}H NMR** (400 MHz, CD_3COCD_3) δ 7.75 (2H, d, J = 9.0 Hz), 7.06 (2H, d, J = 9.0 Hz), 4.72 – 4.66 (1H, m), 4.29 – 4.22 (4H, m), 3.88 (3H, s), 1.44 (9H, s); **^{13}C NMR** (101 MHz, CD_3COCD_3) δ 163.2, 157.0, 146.8, 125.1, 115.1, 79.6, 64.3, 56.0, 54.5, 28.6; **HRMS** (ESI$^+$) [M+Na]$^+$ calc'd for $C_{15}H_{21}N_3NaO_3$: 314.1475, found: 314.1482.

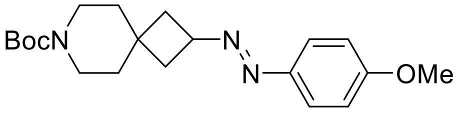

***tert*-Butyl (*E*)-2-((4-methoxyphenyl)diazenyl)-7-azaspiro[3.5]nonane-7-carboxylate (4o):** Prepared according to **General Method F** (Eluent: 100:0 to 10:1 petroleum ether: ethyl acetate) and the title compound was isolated as a yellow solid (153.0 mg, 0.426 mmol, 85.1% yield).

M.p. = 75.2 – 76.4 °C; **IR** (thin film) 1679 (s), 1601 (w), 1506 (m), 1423 (m), 1244 (s), 1178 (s), 1143 (s), 1022 (m), 842 (s), 764 (w) cm^{-1}; **^{1}H NMR** (400 MHz, CD_3COCD_3) δ 7.70 (2H, d, J = 9.0 Hz), 7.03 (2H, d, J = 9.0 Hz), 4.55 – 4.49 (1H, m), 3.87 (3H, s), 3.42 – 3.38 (2H, m), 3.34 – 3.29 (2H, m), 2.32 – 2.22 (4H, m), 1.68 – 1.65 (2H, m), 1.61 – 1.57 (2H, m), 1.44 (9H, s). **^{13}C NMR** (101 MHz, CD_3COCD_3) δ163.3, 155.8, 147.7, 125.4, 115.6, 79.9, 66.4, 56.6, 40.2, 38.3, 38.1, 34.8, 29.3. HRMS (ESI$^+$) $[M+H]^+$ calc'd for $C_{20}H_{29}F_4N_3O_3Na$: 382.2116, found: 382.2101.

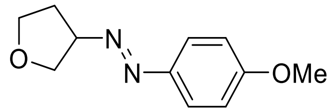

(*E*)-1-(4-methoxyphenyl)-2-(tetrahydrofuran-3-yl)diazene (4p): Prepared according to **General Method F** (Eluent: 100:0 to 50:1 petroleum ether: ethyl acetate) and the title compound was isolated as a yellow oil (78.5 mg, 0.381 mmol, 76.2% yield). **IR** (thin film) 2849 (w), 1620 (m), 1512 (s), 1248 (s), 1141 (s), 1103 (m), 1076 (m), 1028 (m), 910 (w), 836 (s) cm^{-1}; **^{1}H NMR** (500 MHz, CD_3COCD_3) δ 7.69 (2H, d, J = 9.0 Hz), 7.03 (2H, d, J = 9.0 Hz), 4.42 – 4.37 (1H, m), 4.06 – 3.97 (3H, m), 3.90 – 3.82 (4H, m), 2.32 – 2.20 (2H, m). **^{13}C NMR** (125 MHz, CD_3COCD_3) δ 163.5, 147.5, 125.6, 115.7, 78.3, 72.6, 69.2, 56.7, 33.1. HRMS (ESI$^+$) $[M+Na]^+$ calc'd for $C_{11}H_{14}NaN_2O_2$: 229.0941, found: 229.0947.

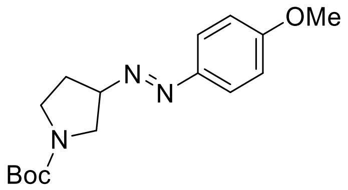

(*E*)-1-cyclohexyl-2-(4-methoxyphenyl)diazene (4q): Prepared according to **General Method F** (Eluent: 100:0 to 5:1 petroleum ether: ethyl acetate) and the title compound was isolated as a light yellow oil (129.0 mg, 0.422 mmol, 84.5% yield). **IR** (thin film) 2974 (w), 1691 (s), 1603 (m), 1513 (m), 1399 (s), 1365 (m), 1249 (m), 1145 (s), 1107 (s), 1030 (m), 879 (m), 838 (s), 770 (w) cm^{-1}; **^{1}H NMR** (400 MHz, CD_3COCD_3) δ 7.69 (2H, d, J = 9.0 Hz), 7.03 (2H, d, J = 9.0 Hz), 4.37 – 4.34 (1H, m), 3.87 (3H, s), 3.68 – 3.67 (2H, m), 3.55 – 3.51 (2H, m), 2.30 – 2.19 (2H, m), 1.45 (9H, s); **^{13}C NMR** (101 MHz, CD_3COCD_3) δ 162.9, 154.8, 146.8, 125.0, 115.0, 79.2, 75.7 (d, J = 90.1 Hz), 56.0, 50.5 (d, J = 65.2 Hz), 45.6 (d, J = 113.4 Hz), 31.1 (d, J = 84.8 Hz), 28.7; **HRMS** (ESI$^+$) $[M+H]^+$ calc'd for $C_{16}H_{23}N_3NaO_3$: 328.1632, found:

328.1643.

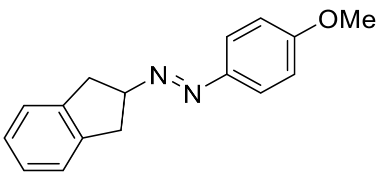

(*E*)-1-(2,3-dihydro-1*H*-inden-2-yl)-2-(4-methoxyphenyl)diazene (4r): Prepared according to **General Method F** (Eluent: 100:0 to 100:1 petroleum ether: ethyl acetate) and the title compound was isolated as a light yellow oil (89.5 mg, 0.355 mmol, 70.9% isolated yield). **IR** (thin film) 2951 (w), 1602 (m), 1585 (m), 1510 (m), 1295 (m), 1245 (s), 1143 (m), 1029 (m), 834 (s), 744 (s) cm^{-1}; **^{1}H NMR** (400 MHz, CD_3COCD_3) δ 7.68 (2H, d, J = 9.0 Hz), 7.29 – 7.27 (2H, m), 7.19 – 7.18 (2H, m), 7.00 (2H, d, J = 9.0 Hz), 4.63 – 4.57 (1H, m), 3.84 (3H, s), 3.33 – 3.31 (4H, m); **^{13}C NMR** (101 MHz, CD_3COCD_3) δ 162.7, 146.9, 142.6, 127.5, 125.4, 124.9, 115.0, 77.6, 56.0, 38.6; **HRMS** (ESI^+) $[M+Na]^+$ calc'd for $C_{16}H_{16}N_2NaO$: 275.1155, found: 275.1164.

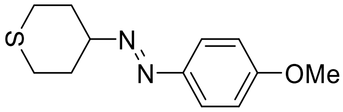

(*E*)-1-(4-methoxyphenyl)-2-(tetrahydro-2H-thiopyran-4-yl)diazene (4s): Prepared according to **General Method F** (Eluent: 100:0 to 10:1 petroleum ether: ethyl acetate) and the title compound was isolated as a yellow solid (96.2 mg, 0.408 mmol, 81.5% yield). M.p. = 67.4 – 68.6 °C. **IR** (thin film) 2912 (w), 1600 (m), 1586 (m), 1509 (s), 1450 (m), 1244 (s), 1145 (s), 1024 (s), 843 (s), 655 (m) cm^{-1}; **^{1}H NMR** (400 MHz, CD_3COCD_3) δ 7.69 (2H, d, J = 9.0 Hz), 7.03 (2H, d, J = 9.0 Hz), 3.86 (3H, s), 3.68 – 3.62 (1H, m), 2.83 – 1.77 (4H, m), 2.16 – 2.00 (4H, m). **^{13}C NMR** (101 MHz, CD_3COCD_3) δ 163.4, 147.7, 125.5, 115.6, 75.8, 56.7, 33.6, 27.9. HRMS (ESI^+) $[M+Na]^+$ calc'd for $C_{12}H_{16}F_4N_2NaOS$: 259.0876, found: 259.0870.

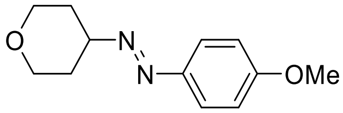

(*E*)-1-(4-methoxyphenyl)-2-(tetrahydro-2H-pyran-4-yl)diazene (4t): Prepared according to **General Method F** (Eluent: 100:0 to 10:1 petroleum ether: ethyl acetate) and the title

compound was isolated as a yellow oil (101.2 mg, 0.460 mmol, 91.9% yield). **IR** (thin film) 2849 (w), 1602 (m), 1515 (m), 1461 (w), 1298 (w), 1243 (s), 1141 (m), 1103 (m), 1085 (m), 840 (s), 653 (w) cm^{-1}; **^{1}H NMR** (400 MHz, CD_3COCD_3) δ 7.69 (2H, d, J = 9.0 Hz), 7.02 (2H, d, J = 9.0 Hz), 4.05 – 3.99 (2H, m), 3.88 – 3.80 (1H, m), 3.86 (3H, s), 3.52 (2H, td, J = 11.4, 2.4 Hz), 2.01 – 1.91 (2H, m), 1.82 – 1.75 (2H, m). **^{13}C NMR** (101 MHz, CD_3COCD_3) δ 163.3, 147.7, 125.4, 115.6, 73.8, 67.2, 56.6, 32.3. HRMS (ESI^{+}) [M+H]$^{+}$ calc'd for $C_{12}H_{17}F_4N_2O_2$: 221.1275, found: 221.1285.

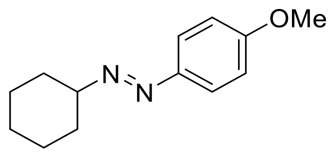

(*E*)-1-cyclohexyl-2-(4-methoxyphenyl)diazene (4u): Prepared according to **General Method F** (Eluent: 100:0 to 50:1 petroleum ether: ethyl acetate) and the title compound was isolated as a light yellow oil (79.6 mg, 0.365 mmol, 72.9% isolated yield). **IR** (thin film) 2929 (m), 1603 (m), 1514 (m), 1450 (m), 1245 (s), 1142 (m), 1102 (m), 1032 (m), 835 (s) cm^{-1}; **^{1}H NMR** (400 MHz, CD_3COCD_3) δ 7.66 (2H, d, J = 9.0 Hz), 7.01 (2H, d, J = 9.0 Hz), 3.85 (3H, s), 3.61 – 3.54 (1H, m), 1.90 – 1.85 (2H, m), 1.79 – 1.76 (3H, m), 1.74 – 1.68 (2H, m), 1.48 – 1.32 (3H, m); **^{13}C NMR** (101 MHz, CD_3COCD_3) δ 162.5, 147.2, 124.7, 114.9, 76.4, 55.9, 31.7, 26.4, 25.1; **HRMS** (ESI^{+}) [M+Na]$^{+}$ calc'd for $C_{13}H_{18}N_2NaO$: 241.1311, found: 241.1307.

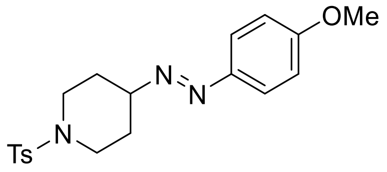

(*E*)-4-((4-methoxyphenyl)diazenyl)-1-tosylpiperidine (4v): Prepared according to **General Method F** (Eluent: 100:0 to 2:1 petroleum ether: ethyl acetate) and the title compound was isolated as a light yellow solid (82.9 mg, 0.222 mmol, 44.4% isolated yield, 4 h). **M.p.** = 151.0 – 151.9 °C; **IR** (thin film) 1605 (w), 1514 (w), 1339 (m), 1245 (s), 1160 (s), 1094 (m), 1037 (m), 928 (m), 843 (m), 814 (m), 733 (s), 649 (w) cm^{-1}; **^{1}H NMR** (400 MHz, CD_3COCD_3) δ 7.71 (2H, d, J = 8.3 Hz), 7.64 (2H, d, J = 9.0 Hz), 7.47 (2H, d, J = 8.0 Hz), 7.02 (2H, d, J = 9.0 Hz), 3.86 (3H, s), 3.78 – 3.73 (2H, m), 3.67 – 3.60 (1H, m), 2.69 – 2.62 (2H, m), 2.45 (3H, s), 2.02 – 1.95 (4H, m); **^{13}C NMR** (101 MHz, CD_3COCD_3) δ 162.9, 147.0, 144.5, 134.7, 130.6,

128.7, 124.8, 115.0, 72.5, 56.0, 45.5, 30.5, 21.5; **HRMS** (ESI$^+$) [M+Na]$^+$ calc'd for $C_{19}H_{23}N_3NaO_3S$: 396.1352, found: 396.1371.

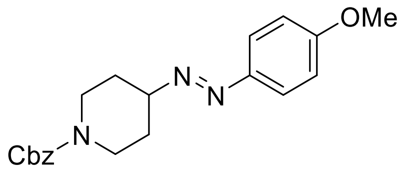

Benzyl (*E*)-4-((4-methoxyphenyl)diazenyl)piperidine-1-carboxylate (4w): Prepared according to **General Method F** (Eluent: 100:0 to 3:1 petroleum ether: ethyl acetate) and the title compound was isolated as a light yellow oil (150.6 mg, 0.426 mmol, 85.2% yield). **IR** (thin film) 1694 (s), 1603 (m), 1513 (m), 1428 (m), 1248 (s), 1224 (s), 1147 (m), 1027 (m), 837 (m), 763 (m), 697 (m) cm^{-1}; **^{1}H NMR** (400 MHz, CD_3COCD_3) δ 7.70 (2H, d, J = 7.5 Hz), 7.44 – 7.30 (5H, m), 7.02 (2H, d, J = 9.0 Hz), 5.17 (2H, s), 4.20 – 4.17 (2H, m), 3.87 – 3.79 (4H, m), 3.14 (2H, s), 1.89 – 1.84 (4H, m); **^{13}C NMR** (101 MHz, CD_3COCD_3) δ 162.7, 155.6, 147.0, 138.3, 129.3, 128.6, 128.6, 124.8, 114.9, 73.6, 67.3, 55.9, 43.0, 31.7; **HRMS** (ESI$^+$) [M+Na]$^+$ calc'd for $C_{20}H_{23}N_3NaO_3$: 376.1632, found: 376.1639.

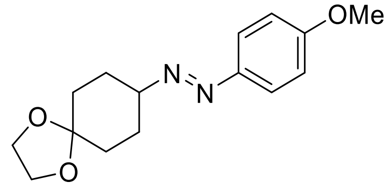

(*E*)-1-(4-methoxyphenyl)-2-(1,4-dioxaspiro[4.5]decan-8-yl)diazene (4x): Prepared according to **General Method F** (Eluent: 100:0 to 100:1 petroleum ether: ethyl acetate, MeCN instead of MeOH) and the title compound was isolated as a light yellow oil (102.4 mg, 0.371 mmol, 74.1% isolated yield). **IR** (thin film) 2920 (w), 1602 (w), 1586 (m), 1514 (w), 1245 (s), 1151 (m), 1092 (s), 1064(s), 1029(s), 928 (s), 834 (s), 674 (m) cm^{-1}; **^{1}H NMR** (400 MHz, CD_3COCD_3) δ 7.68 (2H, d, J = 9.0 Hz), 7.03 (2H, d, J = 9.0 Hz), 3.95 (4H, s), 3.86 (3H, s), 3.69 – 3.62 (1H, m), 2.07 – 2.00 (2H, m), 1.91 – 1.81 (4H, m), 1.72 – 1.65 (2H, m); **^{13}C NMR** (101 MHz, CD_3COCD_3) δ 162.6, 147.2, 124.7, 114.9, 108.7, 74.5, 64.9, 56.0, 33.4, 29.0; **HRMS** (ESI$^+$) [M+H]$^+$ calc'd for $C_{15}H_{21}N_2O_3$: 277.1547, found: 277.1543.

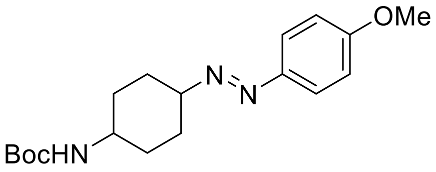

***tert*-Butyl (*E*)-(4-((4-methoxyphenyl)diazenyl)cyclohexyl)carbamate (4y):** Prepared according to **General Method F** (Eluent: 100:0 to 20:1 petroleum ether: ethyl acetate) and the title compound was isolated as a light yellow oil (109.1 mg, 0.327 mmol, 65.4% isolated yield). **IR** (thin film) 2919 (w), 1709 (m), 1678 (m), 1601 (m), 1548 (m), 1514 (m), 1364 (w), 1310 (w), 1250 (s), 1139 (m), 1025 (s), 833 (m) cm^{-1}; **^{1}H NMR** (400 MHz, CD_3COCD_3) δ 7.68 (2H, d, J = 9.0 Hz), 7.02 (2H, d, J = 9.0 Hz), 6.05 (1H, br), 3.86 (3H, s), 3.71 – 3.65 (2H, m), 2.09 – 2.01 (2H, m), 1.92 – 1.87 (2H, m), 1.82 – 1.75 (4H, m), 1.41 (9H, s); **^{13}C NMR** (101 MHz, CD_3COCD_3) δ 162.6, 156.0, 147.3, 124.7, 114.9, 78.4, 72.7, 56.0, 48.5, 29.3, 28.7, 28.6; **HRMS** (ESI$^+$) $[M+H]^+$ calc'd for $C_{18}H_{27}N_3O_3$: 356.1945, found: 356.1945.

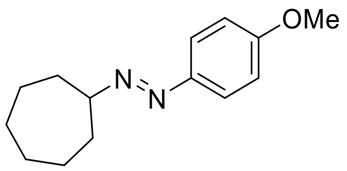

(*E*)-1-cycloheptyl-2-(4-methoxyphenyl)diazene (4z): Prepared according to **General Method F** (Eluent: 100:0 to 100:1 petroleum ether: ethyl acetate) and the title compound was isolated as a light yellow oil (73.5 mg, 0.316 mmol, 63.3% isolated yield). **IR** (thin film) 2924 (m), 1603 (m), 1515 (m), 1461 (w), 1247 (s), 1144 (m), 1033 (m), 837 (m) cm^{-1}; **^{1}H NMR** (400 MHz, CD_3COCD_3) δ 7.65 (2H, d, J = 9.0 Hz), 7.02 (2H, d, J = 9.0 Hz), 3.85 (3H, s), 3.74 – 3.68 (1H, m), 1.95 – 1.79 (6H, m), 1.66 – 1.56 (6H, m); **^{13}C NMR** (101 MHz, CD_3COCD_3) δ 162.5, 147.1, 124.7, 114.9, 78.6, 55.9, 33.5, 29.7, 25.5; **HRMS** (ESI$^+$) $[M+Na]^+$ calc'd for $C_{14}H_{20}N_2NaO$: 255.1468, found: 255.1477.

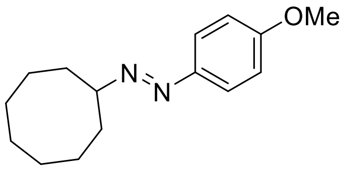

(*E*)-1-cyclooctyl-2-(4-methoxyphenyl)diazene (4aa): Prepared according to **General Method F** (Eluent: 100:0 to 100:1 petroleum ether: ethyl acetate) and the title compound was isolated as a light yellow oil (115.3 mg, 0.468 mmol, 93.6% isolated yield). **IR** (thin film) 2919 (m), 1603 (m), 1515 (m), 1246 (s), 1146 (m), 1033 (m), 836 (m) cm^{-1}; **^{1}H NMR** (400 MHz, CD_3COCD_3) δ 7.65 (2H, d, J = 9.0 Hz), 7.02 (2H, d, J = 9.0 Hz), 3.85 (3H, s), 3.76 – 3.70 (1H, m), 2.00 – 1.85 (4H, m), 1.78 – 1.57 (10H, m); **^{13}C NMR** (101 MHz, CD_3COCD_3) δ 162.5,

147.2, 124.7, 114.9, 78.0, 56.0, 31.5, 27.9, 26.7, 25.0; **HRMS** (ESI^+) $[M+Na]^+$ calc'd for $C_{15}H_{22}N_2NaO$: 269.1624, found: 269.1638

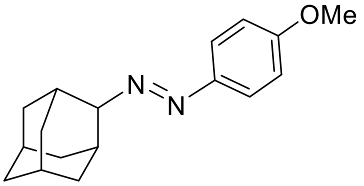

(*E*)-1-((1*r*,3*r*,5*r*,7*r*)-adamantan-2-yl)-2-(4-methoxyphenyl)diazene (4ab): Prepared according to **General Method F** (Eluent: 100:0 to 200:1 petroleum ether: ethyl acetate) and the title compound was isolated as a light yellow solid (111.3 mg, 0.412 mmol, 82.3% isolated yield). **M.p.** = 68.4 – 69.2 °C; **IR** (thin film) 2898 (s), 2849 (m), 1602 (m), 1514 (m), 1439 (w), 1308 (m), 1244 (s), 1183 (m), 1142 (m), 1099 (m), 1028 (m), 850 (m), 835 (s) cm^{-1}; **^{1}H NMR** (400 MHz, CD_3COCD_3) δ 7.71 (2H, d, J = 8.9 Hz), 7.03 (2H, d, J = 8.9 Hz), 3.86 (3H, s), 3.67 (1H, s), 2.39 (2H, d, J = 12.2 Hz), 2.02 – 1.86 (10H, m), 1.70 – 1.67 (2H, m); **^{13}C NMR** (101 MHz, CD_3COCD_3) δ 162.5, 147.6, 124.7, 114.9, 80.7, 56.0, 38.5, 37.9, 34.3, 33.1, 29.1, 28.4; **HRMS** (ESI^+) $[M+H]^+$ calc'd for $C_{17}H_{23}N_2O$: 271.1805, found: 271.1795.

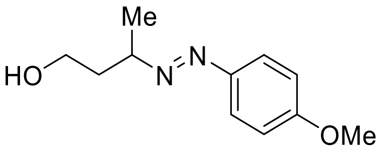

(*E*)-3-((4-methoxyphenyl)diazenyl)butan-1-ol (4ac): Prepared according to **General Method F** (Eluent: 5:1 to 1:1 petroleum ether: ethyl acetate) and the title compound was isolated as a light yellow oil (98.3 mg, 0.472 mmol, 94.4% yield). **IR** (thin film) 3381 (w), 2934 (w), 1603 (m), 1516 (m), 1248 (s), 1147 (m), 1030 (m), 835 (m) cm^{-1}; **^{1}H NMR** (400 MHz, CD_3OD) δ 7.65 (2H, d, J = 9.0 Hz), 6.97 (2H, d, J = 9.0 Hz), 3.88 – 3.83 (1H, m), 3.83 (3H, s), 3.63 – 3.54 (2H, m), 2.20 – 2.11 (1H, m), 1.98 – 1.89 (1H, m), 1.32 (3H, d, J = 6.6 Hz); **^{13}C NMR** (101 MHz, CD_3OD) δ 163.1, 147.3, 124.9, 115.1, 70.5, 59.9, 56.0, 39.0, 19.3; **HRMS** (ESI^+) $[M+Na]^+$ calc'd for $C_{11}H_{16}N_2NaO_2$: 231.1104, found: 231.1105.

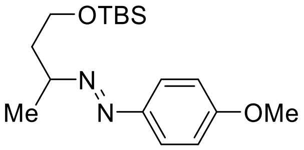

(*E*)-1-(4-((tert-butyldimethylsilyl)oxy)butan-2-yl)-2-(4-methoxyphenyl)diazene (4ad):

Prepared according to **General Method F** (Eluent: 100:0 to 100:1 petroleum ether: ethyl acetate) and the title compound was isolated as a yellow oil (142.2 mg, 0.441 mmol, 88.3% yield). **IR** (thin film) 2929 (w), 1603 (m), 1518 (m), 1248 (s), 1144 (m), 1099 (m), 832 (s), 774 (s) cm^{-1}; **^{1}H NMR** (400 MHz, CD_3COCD_3) δ 7.65 (2H, d, J = 8.8 Hz), 6.99 (2H, d, J = 8.8 Hz), 3.92 – 3.84 (1H, m), 3.82 (3H, s), 3.70 – 3.57 (2H, m), 2.15 – 2.07 (1H, m), 1.93 – 1.84 (1H, m), 1.27 (3H, d, J = 6.7 Hz), 0.86 (9H, s), 0 (6H, s). **^{13}C NMR** (101 MHz, CD_3COCD_3) δ 163.2, 147.7, 125.4, 115.5, 70.4, 61.4, 56.6, 39.7, 27.0, 20.1, 19.5, –4.42. HRMS (ESI^+) $[M+Na]^+$ calc'd for $C_{17}H_{30}N_2NaO_2Si$: 345.1971, found: 345.1969.

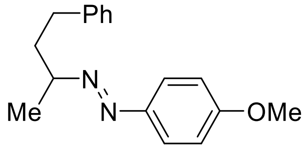

(*E*)-1-(4-methoxyphenyl)-2-(4-phenylbutan-2-yl)diazene (4ae): Prepared according to **General Method C** (Eluent: 100:0 to 100:1 petroleum ether: ethyl acetate) and the title compound was isolated as a yellow oil (116.9 mg, 0.436 mmol, 87.2% yield). **IR** (thin film) 3311 (w), 1602 (m), 1515 (m), 1495 (m), 1247 (s), 1144 (m), 1031 (m), 835 (s), 698 (s) cm-1; **^{1}H NMR** (400 MHz, CD_3COCD_3) δ 7.71 (2H, d, J = 8.8 Hz), 7.26 (2H, dd, J = 7.3, 7.3 Hz), 7.20 (2H, d, J = 7.3 Hz), 7.15 (1H, dd, J = 6.9, 6.9 Hz), 7.03 (2H, d, J = 8.8 Hz), 3.86 (3H, s), 3.79 – 3.69 (1H, m), 2.61 (2H, t, J = 8.0 Hz), 2.26 – 2.17 (1H, m), 2.03 – 1.96 (1H, m), 1.32 (3H, d, J = 6.5 Hz). **^{13}C NMR** (101 MHz, CD_3COCD_3) δ 163.3, 147.8, 143.7, 130.0, 130.0, 127.3, 125.4, 115.6, 73.2, 56.6, 38.6, 33.9, 20.0. HRMS (ESI^+) $[M+Na]^+$ calc'd for $C_{17}H_{20}F_4N_2NaO$: 291.1450, found: 291.1468.

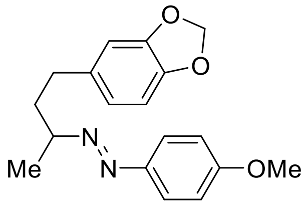

(*E*)-1-(4-(benzo[d][1,3]dioxol-5-yl)butan-2-yl)-2-(4-methoxyphenyl)diazene (4af): Prepared according to **General Method F** (Eluent: 100:0 to 10:1 petroleum ether: ethyl acetate) and the title compound was isolated as a yellow oil (100.2 mg, 0.321 mmol, 64.2% yield). **IR** (thin film) 2929 (w), 1603 (m), 1488 (s), 1440 (m), 1245 (s), 1145 (m), 1034 (s), 927 (m), 836 (m) cm^{-1}; **^{1}H NMR** (400 MHz, CD_3COCD_3) δ 7.69 (2H, d, J = 8.9 Hz), 7.02 (2H, d, J = 8.9

Hz), 6.74 – 6.64 (3H, m), 5.92 (2H, s), 3.86 (3H, s), 3.77 – 3.68 (1H, m), 2.52 (2H, t, *J* = 8.0 Hz), 2.23 – 2.14 (1H, m), 2.02 – 1.93 (1H, m), 1.31 (3H, d, *J* = 6.6 Hz). **^{13}C NMR** (101 MHz, CD_3COCD_3) δ 163.2, 149.2, 147.7, 147.3, 137.5, 125.4, 122.7, 115.6, 110.3, 109.5, 102.4, 73.1, 56.6, 38.8, 33.6, 20.0. HRMS (ESI$^+$) [M+H]$^+$ calc'd for $C_{18}H_{21}N_2O_3$: 313.1556, found: 313.1547.

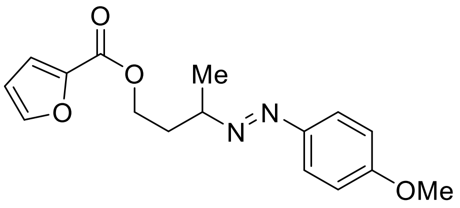

(*E*)-3-((4-methoxyphenyl)diazenyl)butyl furan-2-carboxylate (4ag): Prepared according to **General Method F** (Eluent: 100:0 to 5:1 petroleum ether: ethyl acetate) and the title compound was isolated as a light yellow oil (134.6 mg, 0.445 mmol, 89.0% isolated yield). **IR** (thin film) 2925 (w), 1717 (m), 1603 (w), 1515 (m), 1473 (m), 1398 (w), 1294 (s), 1248 (s), 1179 (s), 1115 (s), 1030 (w), 885 (w), 837 (s), 761 (s) cm^{-1}; **^{1}H NMR** (400 MHz, CD_3COCD_3) δ 7.78 – 7.77 (1H, m), 7.68 (2H, d, *J* = 9.0 Hz), 7.19 (1H, d, *J* = 4.1 Hz), 7.01 (2H, d, *J* = 9.0 Hz), 6.61 – 6.59 (1H, m), 4.39 – 4.27 (2H, m), 3.97 – 3.89 (1H, m), 3.85 (3H, s), 2.41 – 2.32 (1H, m), 2.21 – 2.13 (1H, m), 1.36 (3H, d, *J* = 6.6 Hz); **^{13}C NMR** (101 MHz, CD_3COCD_3) δ 162.6, 158.8, 147.7, 146.9, 145.6, 124.8, 118.7, 114.9, 112.8, 70.1, 62.6, 56.0, 34.8, 19.3; **HRMS** (ESI$^+$) [M+Na]$^+$ calc'd for $C_{16}H_{18}N_2NaO_4$: 325.1159, found: 325.1181.

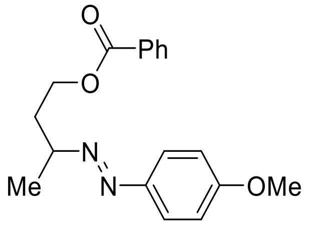

(*E*)-3-((4-methoxyphenyl)diazenyl)butyl benzoate (4ah): Prepared according to **General Method F** (Eluent: 100:0 to 100:1 petroleum ether: ethyl acetate) and the title compound was isolated as a yellow oil (110.1 mg, 0.353 mmol, 70.5% yield). **IR** (thin film) 1716 (s), 1602 (m), 1515 (m), 1452 (m), 1247 (s), 1108 (m), 1028 (m), 836 (m), 709 (s) cm^{-1}; **^{1}H NMR** (400 MHz, CD_3COCD_3) δ 7.99 (2H, d, *J* = 8.4 Hz), 7.69 (2H, d, *J* = 8.8 Hz), 7.60 (1H, dd, *J* = 7.4, 7.4 Hz), 7.46 (2H, dd, *J* = 8.0, 7.4 Hz), 7.01 (2H, d, *J* = 8.4 Hz), 4.43 – 4.31 (2H, m), 4.00 – 3.93 (1H, m), 3.84 (3H, s), 2.46 – 2.37 (1H, m), 2.26 – 2.17 (1H, m), 1.38 (3H, d, *J* = 6.6 Hz).

^{13}C NMR (101 MHz, CD_3COCD_3) *δ* 167.3, 163.4, 147.6, 134.5, 132.0, 130.9, 130.0, 125.5, 115.6, 71.1, 63.6, 56.6, 35.6, 20.0. HRMS (ESI$^+$) [M+Na]$^+$ calc'd for $C_{18}H_{20}N_2NaO_3$: 335.1370, found: 335.1366.

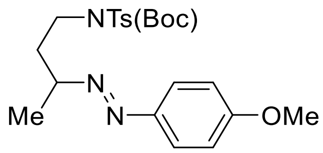

***tert*-butyl (*E*)-(3-((4-methoxyphenyl)diazenyl)butyl)(tosyl)carbamate (4ai)**: Prepared according to **General Method F** (Eluent: 100:0 to 100:1 petroleum ether: ethyl acetate) and the title compound was isolated as a yellow solid (190.9 mg, 0.414 mmol, 82.8% yield). **IR** (thin film) 1725 (s), 1602 (m), 1515 (m), 1351 (s), 1287 (s), 1140 (m), 1029 (m), 838 (m), 673 (s) cm^{-1}; **^{1}H NMR** (400 MHz, CD_3COCD_3) *δ* 7.83 (2H, d, *J* = 8.4 Hz), 7.71 (2H, d, *J* = 8.9 Hz), 7.42 (2H, d, *J* = 8.7 Hz), 7.03 (2H, d, *J* = 8.8 Hz), 3.95 – 3.79 (3H, m), 3.89 (3H, s), 2.43 (3H, s), 2.40 – 2.30 (1H, m), 2.20 – 2.10 (1H, m), 1.36 (2H, d, *J* = 6.6 Hz), 1.31 (9H, s). **^{13}C NMR** (101 MHz, CD_3COCD_3) *δ* 163.4, 152.3, 147.8, 145.9, 139.4, 131.0, 129.4, 125.6, 115.7, 85.2, 71.7, 56.7, 45.9, 37.2, 28.7, 28.7, 22.2, 19.8. HRMS (ESI$^+$) [M+Na]$^+$ calc'd for $C_{23}H_3F_4N_3NaSO_5$: 484.1844, found: 484.1877.

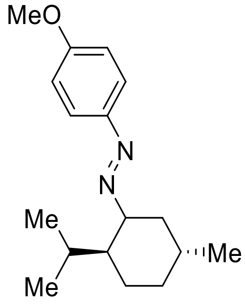

(*E*)-1-((*2S,5R*)-2-isopropyl-5-methylcyclohexyl)-2-(4-methoxyphenyl)diazene (4aj): Prepared according to **General Method F** (Eluent: 100:0 to 200:1 petroleum ether: ethyl acetate) and the title compound was isolated as a light yellow oil (105.8 mg, 0.386 mmol, 77.1% isolated yield, dr = 2: 1). **IR** (thin film) 2918 (w), 1603 (m), 1514 (m), 1456 (w), 1247 (s), 1143 (m), 1034 (m), 835 (s) cm^{-1}; **Isomer 1: ^{1}H NMR** (400 MHz, CD_3COCD_3) *δ* 7.67 (2H, d, *J* = 8.9 Hz), 7.02 (2H, d, *J* = 8.9 Hz), 3.95 – 3.92 (1H, m), 3.86 (3H, s), 1.69 – 1.64 (1H, m), 1.38 – 1.34 (1H, m), 1.28 – 1.27 (2H, m), 1.26 – 1.23 (1H, m), 1.21 – 1.17 (2H, m), 1.11 – 1.02 (2H, m), 0.88 – 0.85 (6H, m), 0.80 (3H, d, *J* = 6.6 Hz); **Isomer 2: ^{1}H NMR** (400 MHz, CD_3COCD_3) *δ* 7.67 (2H, d, *J* = 8.9 Hz), 7.02 (2H, d, *J* = 8.9 Hz), 3.86 (3H, s), 3.54 – 3.47 (1H, m), 2.08 –

2.00 (1H, m), 1.99 – 1.93 (2H, m), 1.82 – 1.75 (2H, m), 1.58 – 1.54 (2H, m), 1.49 – 1.47 (1H, m), 1.44 – 1.40 (1H, m), 0.95 (3H, d, *J* = 6.3 Hz), 0.89 (3H, d, *J* = 7.0 Hz), 0.70 (3H, d, *J* = 7.0 Hz); **Isomer 1:** ^{13}**C NMR** (101 MHz, CD_3COCD_3) δ 162.5, 147.5, 124.7, 114.9, 74.7, 55.9, 48.9, 42.8, 36.6, 30.5, 27.9, 27.0, 23.2, 21.3, 20.7; **Isomer 2:** ^{13}**C NMR** (101 MHz, CD_3COCD_3) δ 162.5, 147.2, 124.7, 114.9, 78.7, 55.9, 47.0, 40.6, 35.4, 32.0, 28.4, 24.3, 22.8, 21.2, 16.1; **HRMS** (ESI^+) $[M+Na]^+$ calc'd for $C_{17}H_{26}N_2NaO$: 297.1937, found: 297.1920.

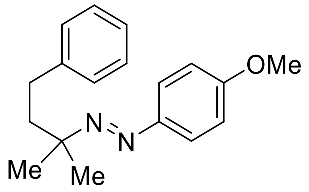

(*E*)-1-(4-methoxyphenyl)-2-(2-methyl-4-phenylbutan-2-yl)diazene (6a): Prepared according to **General Method F** (Eluent: 100:0 to 100:1 petroleum ether: ethyl acetate) and the title compound was isolated as a light yellow oil (114.1 mg, 0.404 mmol, 80.8% isolated yield). **IR** (thin film) 2967 (w), 1603 (m), 1520 (m), 1500 (m), 1455 (w), 1247 (s), 1141 (m), 1032 (m), 836 (s), 738 (m), 698 (s) cm^{-1}; ^{1}**H NMR** (400 MHz, CD_3COCD_3) δ 7.72 (2H, d, *J* = 8.8 Hz), 7.27 – 7.19 (4H, m), 7.16 – 7.13 (1H, m), 7.03 (2H, d, *J* = 9.0 Hz), 3.85 (3H, s), 2.64 – 2.59 (2H, m), 2.09 – 2.06 (2H, m), 1.34 (6H, s); ^{13}**C NMR** (101 MHz, CD_3COCD_3) δ 162.5, 147.1, 143.7, 129.2, 129.1, 126.5, 124.6, 114.9, 69.7, 55.9, 43.9, 31.3, 25.2; **HRMS** (ESI^+) $[M+H]^+$ calc'd for $C_{18}H_{23}N_2O$: 283.1805, found: 283.1796.

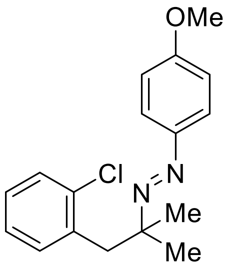

(*E*)-1-(1-(2-chlorophenyl)-2-methylpropan-2-yl)-2-(4-methoxyphenyl)diazene (6b): Prepared according to **General Method F** (Eluent: 100:0 to 100:1 petroleum ether: ethyl acetate) and the title compound was isolated as a yellow liquid (127.8 mg, 0.423 mmol, 84.6% yield). **IR** (thin film) 1603 (m), 1519 (m), 1440 (w), 1247 (s), 1137 (m), 1103 (w), 1030 (m), 835 (s), 744 (s), 681 (s) cm^{-1}; ^{1}**H NMR** (400 MHz, CD_3COCD_3) δ 7.71 (2H, d, *J* = 8.8 Hz), 7.37– 7.35 (1H, m), 7.24– 7.22 (1H, m), 7.20– 7.12 (2H, m), 7.03 (2H, d, *J* = 8.8 Hz), 3.85 (3H, s), 3.33 (2H, s), 1.30 (6H, s). ^{13}**C NMR** (101 MHz, CD_3COCD_3) δ 162.6, 146.9, 137.0, 135.6,

133.97, 130.3, 128.8, 127.2, 124.8, 114.9, 71.3, 56.0, 43.6, 25.3. HRMS (ESI$^+$) $[M+H]^+$ calc'd for $C_{17}H_{19}N_2ClO$: 303.1259, found: 303.1259.

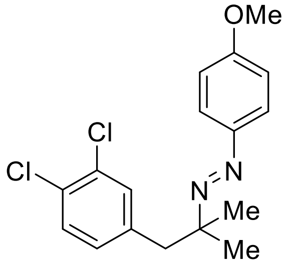

(*E*)-1-(1-(2-chlorophenyl)-2-methylpropan-2-yl)-2-(4-methoxyphenyl)diazene (6c): Prepared according to **General Method F** (Eluent: 100:0 to 100:1 petroleum ether: ethyl acetate) and the title compound was isolated as a yellow liquid (139.6 mg, 0.414 mmol, 82.8% yield). **IR** (thin film) 1603 (m), 1519 (m), 1469 (m), 1247 (s), 1140 (m), 1030 (m), 835 (s), 748 (m), 681 (2) cm^{-1}; **^{1}H NMR** (400 MHz, CD_3COCD_3) δ 7.69 (2H, d, J = 9.0 Hz), 7.41– 7.36 (2H, m), 7.13 (1H, dd, J = 8.3, 2.0 Hz), 7.04 (2H, d, J = 9.0 Hz), 3.85 (3H, s), 3.33 (2H, s), 1.30 (6H, s). **^{13}C NMR** (101 MHz, CD_3COCD_3) δ 162.6, 146.8, 140.3, 133.5, 131.8, 131.7, 130.6, 130.4, 124.7, 115.0, 70.2, 56.0, 46.5, 25.2. HRMS (ESI$^+$) $[M+H]^+$ calc'd for $C_{17}H_{18}N_2Cl_2O$: 337.0869, found: 337.0869.

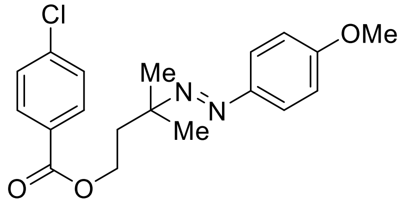

(*E*)-3-((4-methoxyphenyl)diazenyl)-3-methylbutyl 4-chlorobenzoate (6d): Prepared according to **General Method F** (Eluent: 100:0 to 20:1 petroleum ether: ethyl acetate) and the title compound was isolated as a light yellow oil (165.0 mg, 0.457 mmol, 91.5% isolated yield). **IR** (thin film) 2976 (w), 1718 (s), 1595 (m), 1520 (w), 1489 (w), 1271 (s), 1248 (s), 1171 (w), 1141 (s), 1091 (s), 1015 (m), 836 (s), 759 (s), 684 (w) cm^{-1}; **^{1}H NMR** (400 MHz, CD_3COCD_3) δ 7.91 (2H, d, J = 8.9 Hz), 7.66 (2H, d, J = 9.0 Hz), 7.44 (2H, d, J = 8.9 Hz), 6.99 (2H, d, J = 9.0 Hz), 4.44 (2H, t, J = 6.9 Hz), 3.85 (3H, s), 2.30 (2H, t, J = 6.9 Hz), 1.36 (6H, s); **^{13}C NMR** (101 MHz, CD_3COCD_3) δ 165.8, 162.6, 146.9, 139.5, 131.9, 130.0, 129.5, 124.6, 114.8, 68.9, 62.6, 56.0, 39.8, 25.5; **HRMS** (ESI$^+$) $[M+Na]^+$ calc'd for $C_{19}H_{21}ClN_2NaO_3$: 383.1133, found:

383.1137.

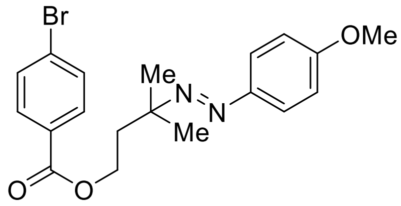

(E)-3-((4-methoxyphenyl)diazenyl)-3-methylbutyl 4-bromobenzoate (6e): Prepared according to **General Method F** (Eluent: 100:0 to 50:1 petroleum ether: ethyl acetate) and the title compound was isolated as a light yellow oil (157.1 mg, 0.388 mmol, 77.5% isolated yield). **IR** (thin film) 2958 (w), 1706 (s), 1590 (m), 1523 (m), 1495 (m), 1399 (m), 1271 (s), 1252 (s), 1172 (m), 1139 (m), 1104 (s), 1027 (m), 1012 (m), 833 (s), 757 (s), 685 (w) cm^{-1}; **^{1}H NMR** (400 MHz, CD_3COCD_3) δ 7.82 (2H, d, J = 8.6 Hz), 7.66 (2H, d, J = 9.0 Hz), 7.58 (2H, d, J = 8.6 Hz), 6.98 (2H, d, J = 9.0 Hz), 4.43 (2H, t, J = 6.9 Hz), 3.85 (3H, s), 2.29 (2H, t, J = 6.9 Hz), 1.35 (6H, s); **^{13}C NMR** (101 MHz, CD_3COCD_3) δ 165.9, 162.5, 146.8, 132.5, 132.0, 130.3, 128.1, 124.6, 114.8, 68.9, 62.5, 55.9, 39.8, 25.5; **HRMS** (ESI^+) $[M+Na]^+$ calc'd for $C_{19}H_{21}BrN_2NaO_3$: 427.0628, found: 427.0627.

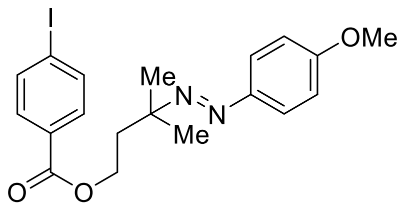

(E)-3-((4-methoxyphenyl)diazenyl)-3-methylbutyl 4-iodobenzoate (6f): Prepared according to **General Method F** (Eluent: 100:0 to 50:1 petroleum ether: ethyl acetate) and the title compound was isolated as a light yellow solid (158.3 mg, 0.350 mmol, 70.0% isolated yield). **M.p.** = 64.8 – 65.4 °C. **IR** (thin film) 2962 (w), 1708 (s), 1586 (m), 1523 (w), 1494 (w), 1394 (w), 1269 (s), 1248 (s), 1175 (m), 1139 (m), 1102 (s), 1025 (m), 1008 (m), 832 (s), 753 (s), 684 (w) cm^{-1}; **^{1}H NMR** (400 MHz, CD_3COCD_3) δ 7.81 (2H, d, J = 8.4 Hz), 7.68 – 7.64 (4H, m), 6.99 (2H, d, J = 9.0 Hz), 4.43 (2H, t, J = 6.9 Hz), 3.86 (3H, s), 2.29 (2H, t, J = 6.9 Hz), 1.35 (6H, s); **^{13}C NMR** (101 MHz, CD_3COCD_3) δ 166.2, 162.6, 146.9, 138.6, 131.8, 130.8, 124.7, 114.9, 101.0, 69.0, 62.5, 56.0, 39.8, 25.5; **HRMS** (ESI^+) $[M+Na]^+$ calc'd for $C_{19}H_{21}IN_2NaO_3$: 475.0489, found: 475.0484.

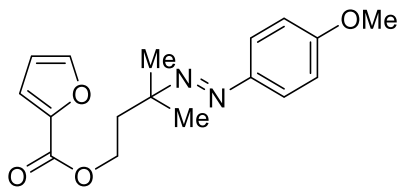

(*E*)-3-((4-methoxyphenyl)diazenyl)-3-methylbutyl furan-2-carboxylate (6g): Prepared according to **General Method F** (Eluent: 100:0 to 20:1 petroleum ether: ethyl acetate) and the title compound was isolated as a light yellow oil (125.7 mg, 0.397 mmol, 79.5% isolated yield). **IR** (thin film) 2963 (w), 1716 (s), 1604 (m), 1519 (m), 1472 (m), 1399 (m), 1294 (s), 1248 (s), 1179 (s), 1141 (s), 1117 (s), 1030 (w), 885 (w), 836 (s), 761 (s) cm^{-1}; **^{1}H NMR** (400 MHz, CD_3COCD_3) δ 7.76 – 7.76 (1H, m), 7.69 (2H, d, J = 9.0 Hz), 7.15 (1H, d, J = 3.5 Hz), 7.01 (2H, d, J = 9.0 Hz), 6.59 – 6.58 (1H, m), 4.40 (2H, t, J =7.1 Hz), 3.85 (3H, s), 2.25 (2H, t, J = 7.1 Hz), 1.35 (6H, s); **^{13}C NMR** (101 MHz, CD_3COCD_3) δ 162.6, 158.9, 147.7, 147.0, 145.7, 124.7, 118.6, 114.9, 112.7, 68.9, 62.0, 56.0, 39.8, 25.5; **HRMS** (ESI^+) $[M+Na]^+$ calc'd for $C_{17}H_{20}N_2NaO_4$: 339.1315, found: 339.1306.

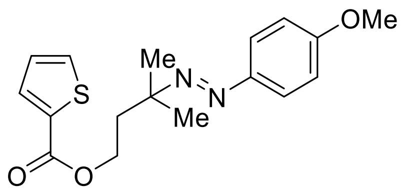

(*E*)-3-((4-methoxyphenyl)diazenyl)-3-methylbutyl thiophene-2-carboxylate (6h): Prepared according to **General Method F** (Eluent: 100:0 to 20:1 petroleum ether: ethyl acetate) and the title compound was isolated as a light yellow oil (146.1 mg, 0.440 mmol, 87.9% isolated yield). **IR** (thin film) 2967 (w), 1706 (m), 1603 (m), 1520 (m), 1419 (m), 1359 (m), 1249 (s), 1141 (m), 1094 (s), 1031 (m), 836 (m), 749 (m), 719(m) cm^{-1}; **^{1}H NMR** (400 MHz, CD_3COCD_3) δ 7.77 – 7.76 (1H, m), 7.75 – 7.74 (1H, m), 7.69 (2H, d, J = 9.0 Hz), 7.15 – 7.13 (1H, m), 7.00 (2H, d, J = 9.0 Hz), 4.42 (2H, t, J = 7.0 Hz), 3.85 (3H, s), 2.26 (2H, t, J = 7.0 Hz), 1.36 (6H, s);**^{13}C NMR** (101 MHz, CD_3COCD_3) δ 162.5, 162.4, 146.9, 134.7, 134.1, 133.7, 128.8, 124.6, 114.8, 68.8, 62.4, 55.9, 39.8, 25.5; **HRMS** (ESI^+) $[M+Na]^+$ calc'd for $C_{17}H_{20}N_2NaO_3S$: 355.1087, found: 355.1086.

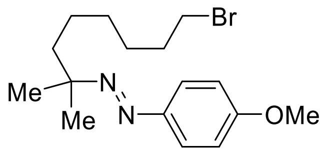

(*E*)-1-(8-bromo-2-methyloctan-2-yl)-2-(4-methoxyphenyl)diazene (6i): Prepared according to **General Method F** (Eluent: 100:0 to 100:1 petroleum ether: ethyl acetate) and the title compound was isolated as a yellow liquid (142.2 mg, 0.418 mmol, 83.6% yield). **IR** (thin film) 2934 (w), 1603 (m), 1520 (m), 1494 (w), 1247 (s), 1140 (m), 1032 (m), 835 (s) cm^{-1}; **^{1}H NMR** (400 MHz, CD_3COCD_3) δ 7.67 (2H, d, J = 9.0 Hz), 7.03 (2H, d, J = 9.0 Hz), 3.86 (3H, s), 3.46 (2H, t, J = 6.8 Hz), 1.86 – 1.73 (4H, m), 1.47 – 1.39 (2H, m), 1.34 – 1.28 (4H, m), 1.25 (6H, s). **^{13}C NMR** (101 MHz, CD_3COCD_3) δ 162.4, 147.1, 124.5, 114.9, 69.8, 56.0, 41.6, 34.8, 33.6, 28.8, 25.2, 24.7. HRMS (ESI$^+$) $[M+H]^+$ calc'd for $C_{16}H_{25}N_2BrO$: 341.1223, found: 341.1229.

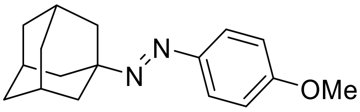

(*E*)-1-((*3s*,*5s*,*7s*)-adamantan-1-yl)-2-(4-methoxyphenyl)diazene (6j): Prepared according to **General Method F** (Eluent: 100:0 to 200:1 petroleum ether: ethyl acetate) and the title compound was isolated as a light yellow solid (121.0 mg, 0.448 mmol, 89.5% isolated yield). **M.p.** = 69.4 – 70.9 °C. **IR** (thin film) 2903 (m), 2848 (w), 1601 (m), 1515 (m), 1452 (w), 1312 (s), 1247 (s), 1177 (w), 1147 (w), 1105 (w), 1029 (s), 832 (s) cm^{-1}; **^{1}H NMR** (400 MHz, CD_3COCD_3) δ 7.67 (2H, d, J = 9.0 Hz), 7.00 (2H, d, J = 9.0 Hz), 3.84 (3H, s), 2.19 – 1.15 (3H, m), 1.90 – 1.89 (6H, m), 1.81 – 1.72 (6H, m); **^{13}C NMR** (101 MHz, CD_3COCD_3) δ 162.3, 147.3, 124.4, 114.8, 68.1, 55.9, 41.3, 37.3, 30.2; **HRMS** (ESI$^+$) $[M+H]^+$ calc'd for $C_{17}H_{23}N_2O$: 271.1805, found: 271.1786.

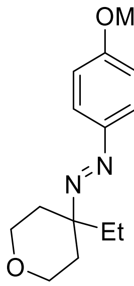

(*E*)-1-(4-ethyltetrahydro-2H-pyran-4-yl)-2-(4-methoxyphenyl)diazene (6k): Prepared according to **General Method F** (Eluent: 100:0 to 100:1 petroleum ether: ethyl acetate) and the title compound was isolated as a yellow liquid (108.3 mg, 0.431mmol, 86.1% yield). **IR** (thin film) 2956 (w), 1603 (m), 1518 (m), 1462 (w), 1247 (s), 1142 (m), 1102 (m), 1030 (m), 836 (s) cm^{-1}; **^{1}H NMR** (500 MHz, CD_3COCD_3) δ 7.71 (2H, d, *J* = 8.8 Hz), 7.05 (2H, d, *J* = 8.8 Hz), 3.87 (3H, s), 3.72 – 3.68 (2H, m), 3.48 – 3.42 (2H, m), 2.17 – 2.10 (2H, m), 1.85 – 1.79 (2H, m), 1.75 (2H, q, *J* = 7.5 Hz), 0.74 (3H, t, *J* = 7.5 Hz). **^{13}C NMR** (101 MHz, CD_3COCD_3) δ 163.3, 147.8, 125.1, 115.6, 69.6, 65.4, 56.7, 35.5, 33.2, 8.1. HRMS (ESI^+) $[M+Na]^+$ calc'd for $C_{14}H_{20}N_2NaO_2$: 271.1430, found: 271.1417.

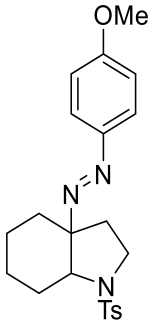

(*E*)-3a-((4-methoxyphenyl)diazenyl)-1-tosyloctahydro-1H-indole (6l): Prepared according to **General Method F** (Eluent: 100:0 to 100:1 petroleum ether: ethyl acetate) and the title compound was isolated as a yellow solid (169.6 mg, 0.411 mmol, 82.1% yield). **M.p.** = 138.6 – 139.2 °C. **IR** (thin film) 1601 (m), 1519 (w), 1337 (m), 1253 (s), 1156 (s), 1108 (w), 1033 (w), 806 (m), 665 (s) cm^{-1}; **^{1}H NMR** (500 MHz, CD_3COCD_3) δ 7.53 (2H, d, *J* = 8.3 Hz), 7.24 (2H, d, *J* = 9.0 Hz), 6.94 (2H, d, *J* = 8.3 Hz), 6.83 (2H, d, *J* = 9.0 Hz), 4.11 (1H, dd, *J* = 10.7, 6.4 Hz), 3.86 (3H, s), 3.56 (1H, td, *J* = 9.0, 1.6 Hz), 3.04 – 2.99 (1H, m), 2.46 – 2.4 (1H, m), 2.37 – 2.33 (1H, m), 2.28 – 2.22 (1H, m), 2.15 (3H, s), 1.79 – 1.75 (1H, m), 1.72 – 1.66 (1H, m), 1.64 – 1.58 (2H, m), 1.43 – 1.26 (2H, m). **^{13}C NMR** (126 MHz, CD_3COCD_3) δ 163.3, 147.8, 125.2, 115.6, 69.6, 65.4, 56.7, 35.5, 33.2, 8.1. HRMS (ESI^+) $[M+Na]^+$ calc'd for $C_{22}H_{27}N_3NaSO_3$: 436.1665, found: 436.1687.

5. 5.0 mmol scale in flask reaction

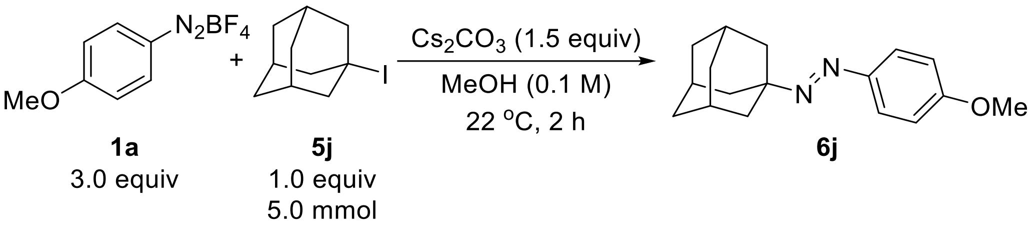

A 120 °C oven-dried 100-mL round-bottom flask, equipped with a stir bar, was charged with 4-methoxybenzenediazonium salt **1a** (3.33 g, 15.0 mmol, 3.00 equiv) and Cs_2CO_3 (2.44 g, 7.50 mmol, 1.50 equiv). The mixture was evacuated and backfilled with nitrogen for three times. Then 1-iodoadamantane **5j** (1.31 g, 5.00 mmol, 1.00 equiv) and MeOH (50.00 mL) were added in one portion under nitrogen. The mixture was allowed to stir for 2 h at 22 °C. The reaction mixture was poured into H_2O (50.0 mL) and extracted with EtOAc (40.0 mL × 3). The combined organic layers were washed with H_2O (50.0 mL), dried over Na_2SO_4 and filtered. The solvent was removed by rotary evaporation and the residue was purified by flash silica gel chromatography. The product **6j** was isolated as light yellow solid (1.20 g, 4.44 mmol, 88.8% yield).

6. Gram scale experiment in flow

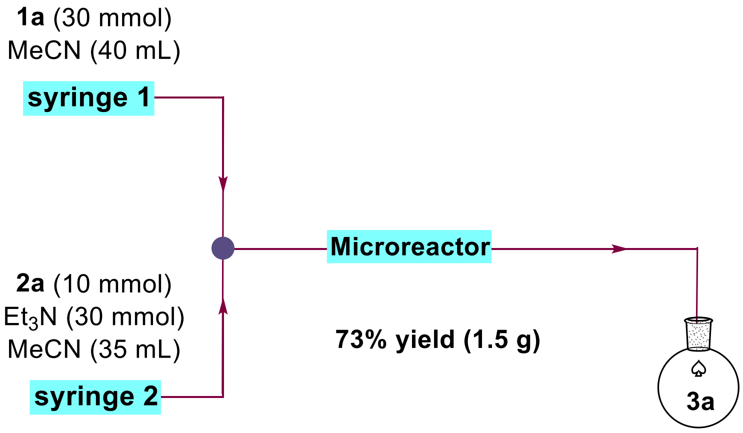

[a] Reaction conditions: **1a** (30 mmol, 3.0 equiv) was dissolved in MeCN (40 mL), **2a** (10 mmol, 1.0 equiv), Et_3N (30 mmol, 3.0 equiv) were dissolved in MeCN (35 mL), flow rate = 0.3 mL/min per syringe, microreactor of a flow system: PFA tube adapted with Y-type mixer, ID = 1mm, residence volume = 3.6 mL, room temperature (22 ^oC).

Supplementary Fig. 6. Details of the reactor for the amination of alkyl iodide via halogen-atom transfer process

Under an argon condition, a 100 mL Schlenk tube was charged with 4-methoxybenzenediazonium salt **1a** (6.66 g, 30.0 mmol, 3.00 equiv) and anhydrous MeCN (40.0 mL). Another 100 mL Schlenk tube was charged with anhydrous MeCN (35.0 mL), then iodocyclopentane **2a** (1.96 g, 10.0 mmol, 1.00 equiv) and Et_3N (3.03 g, 30.0 mmol, 3.00 equiv) were added into the MeCN solution. The above two reaction solutions were transferred into two 50.0 mL BD plastic syringes under argon atmosphere and introduced into the microreactor through syringe pump (The microreactor was wash with anhydrous MeCN 10.0 mL×2 in advance to remove air inside it). The two liquid streams were merged with a Y-Mixer before entering the microreactor. The flow rate was set to 0.30 mL/min per syringe, and the residence time was around 5.2 min (volume of microreactor = 3.60 mL, however: due to the generate of N_2-gas during the reaction, thus the residence time of the reaction was around 5.2 min). After reaching stable state, the reaction solution was collected in a 200 mL beaker. Solution remaining in the microreactor was then discharged with MeCN (5.00 mL × 2) via each syringe pump, and was also collected in the same beaker. The collected reaction solution was evaporated under reduced pressure, the resulting residue was extracted with EtOAc (3×100 mL)

and brine, and the organic layer was collected, dried with Na_2SO_4 and evaporated under reduced pressure. The resulting crude compound was purified by flash chromatography on silica gel to afford the desired product **3a** as light yellow oil (1.49 g, 7.29 mmol, 72.9% yield).

7. Reaction from amine: 1.0 mmol based on alkyl iodide

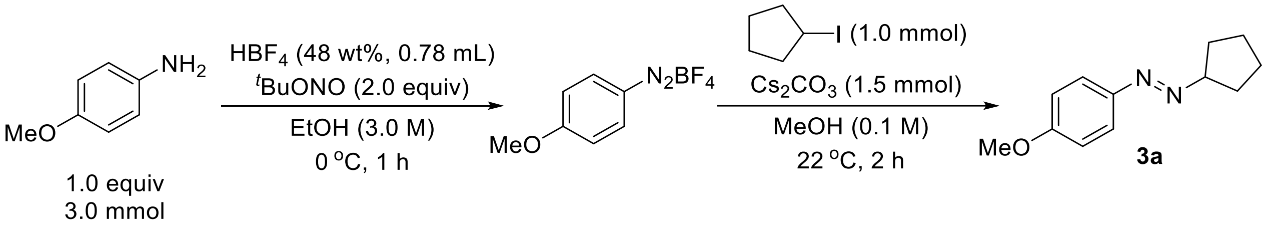

A 120 °C oven-dried 25-mL glass, equipped with a stir bar, the 4-methoxyaniline **1a** (0.370 g, 3.00 mmol, 1.00 equiv) was dissolved in a mixture of absolute EtOH (1.00 mL) and an aqueous solution of HBF_4 (48.0 wt%, 0.780 mL). The mixture was cooled at 0 °C with an ice bath and ^{t}BuONO (0.619 g, 6.00 mmol, 2.00 equiv) was added dropwise. The reaction was stirred at 0 °C for 1 h. Then, the arenediazonium tetrafluoroborate was collected by filtration and was directly used without further purification.

A 120 °C oven-dried 50-mL round-bottom flask, equipped with a stir bar, was charged with arenediazonium tetrafluoroborate and Cs_2CO_3 (489 mg, 1.50 mmol). The mixture was evacuated and backfilled with nitrogen for three times. Then iodide **2a** (196 mg, 1.00 mmol) and MeOH (10.00 mL) were added in one portion under nitrogen. The mixture was allowed to stir for 2 h at 22 °C. The reaction mixture was poured into H_2O (50.0 mL) and extracted with EtOAc (30.0 mL × 3). The combined organic layers were washed with H_2O (30.0 mL), dried over Na_2SO_4 and filtered. The solvent was removed by rotary evaporation and the residue was purified by flash silica gel chromatography. The product **3a** was isolated as light yellow oil (190.1 mg, 0.930 mmol, 93.0% yield).

8. Formation of free amine from deprotection by Pd/C

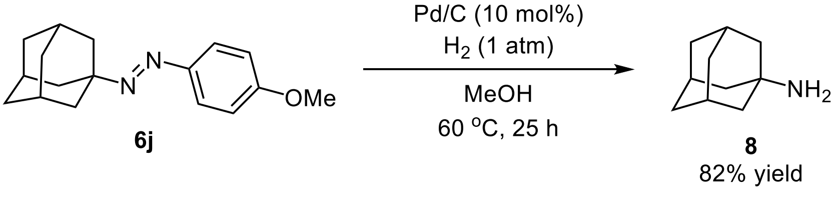

A 10-mL Parr stainless steel autoclave, equipped with a stir bar, was charged with **6j** (54.1 mg, 0.200 mmol, 1.00 equiv), Pd/C (10.0 mol%) and MeOH (2.00 mL). Once prepared and sealed, the autoclave was flushed three with H_2 gas at 1 atm in order to remove ambient air. The mixture was heated to 60 °C and held at that temperature for 25 h. The reaction was cooled to room temperature, and the reaction mixture was then filtered through celite and concentrated in vacuum, and the crude mixture was subjected to ^{1}H NMR spectroscopy in the presence of CH_2Br_2. Crude ^{1}H NMR yield of **8** is 82%.

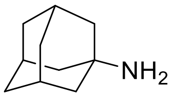

Amantadine (8): ^{1}H NMR (400 MHz, CD_3OD) δ 2.03 – 2.00 (3H, m), 1.71 – 1.60 (12H, m); **^{13}C NMR** (101 MHz, CD_3OD) δ 48.3, 46.4, 37.5, 31.3.

9. Synthetic application

9.1. General Method G: TsOH promoted Indole synthesis

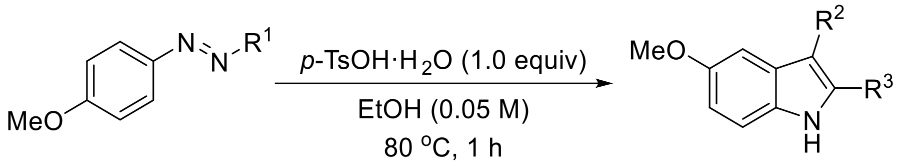

A 120 °C oven-dried 25.0 mL round-bottom flask, equipped with a stir bar, **4l**, **4u**, **4ae** (0.200 mmol, 1.00 equiv) and 4-methylbenzenesulfonic acid monohydrate (38.0 mg, 0.200 mmol, 1.00 equiv) and EtOH (4.00 mL) were added under nitrogen. The resulting mixture was allowed to stir at 80 °C for 1 h. After the completion of the reaction, water (10.0 mL) was added and the mixture was extracted with EtOAc (10.0 mL × 3). The combined organic layers were washed with brine (10.0 mL), dried over Na_2SO_4 and filtrate. The solvent was removed by rotary evaporation and the residue was purified by flash silica gel chromatography to give the desired product.

compound **9**: white solid (37.0 mg, 0.184 mmol, 91.9% yield), compound **10**: brown solid (44.1 mg, 0.175 mmol, 87.7% yield), Spectra were consistent with the literature data[28].

compound **12**: yellow solid (52.2 mg, 0.163 mmol, 81.5% yield), Spectra were consistent with the literature data[29].

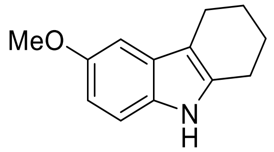

6-Methoxy-2,3,4,9-tetrahydro-1*H*-carbazole (9): ^{1}H NMR (400 MHz, CD_3COCD_3) δ 9.57 (1H, br), 7.15 (1H, d, *J* = 8.7 Hz), 6.88 (1H, d, *J* = 2.4 Hz), 6.64 (1H, dd, *J* = 8.7, 2.5 Hz), 3.77 (3H, s), 2.72 – 2.69 (2H, m), 2.64 – 2.61 (2H, m), 1.87 – 1.82 (4H, m); ^{13}C NMR (101 MHz, CD_3COCD_3) δ 154.5, 136.0, 132.2, 129.2, 113.1, 111.8, 110.7, 100.6, 55.9, 24.2, 24.1, 21.8.

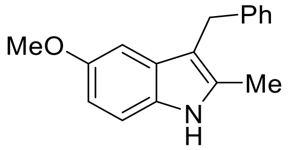

3-Benzyl-5-methoxy-2-methyl-1*H*-indole (10): ^{1}H NMR (400 MHz, CD_3COCD_3) δ 9.77 (1H, br), 7.27 – 7.17 (5H, m), 7.14 – 7.10 (1H, m), 6.90 (1H, d, *J* = 2.4 Hz), 6.67 (1H, dd, *J* = 8.7, 2.4 Hz), 4.03 (2H, s), 3.71 (3H, s), 2.38 (3H, s); ^{13}C NMR (101 MHz, CD_3COCD_3) δ 154.6, 143.2, 133.6, 131.8, 130.3, 129.1, 129.0, 126.3, 111.7, 110.7, 110.7, 101.3, 55.8, 30.7, 11.8.

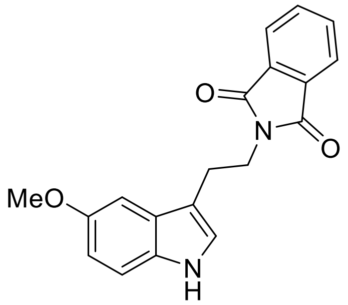

2-(2-(5-Methoxy-1*H*-indol-3-yl)ethyl)isoindoline-1,3-dione (11): ^{1}H NMR (400 MHz, $CDCl_3$) δ 7.93 (1H, s), 7.85 – 7.82 (2H, m), 7.71 – 7.69 (2H, m), 7.23 (1H, d, *J* = 8.8 Hz), 7.17 (1H, d, *J* = 2.4 Hz), 7.08 (1H, d, *J* = 2.2 Hz), 6.83 (1H, dd, *J* = 8.8, 2.4 Hz), 3.99 (2H, t, *J* = 7.8 Hz), 3.87 (3H, s), 3.12 (2H, t, *J* = 8.0 Hz); **^{13}C NMR** (101 MHz, $CDCl_3$) δ 168.4, 154.1, 133.9, 132.2, 131.3, 127.8, 123.2, 122.7, 112.6, 112.2, 111.9, 100.2, 55.8, 38.4, 24.5.

9.2 Melatonin synthesis

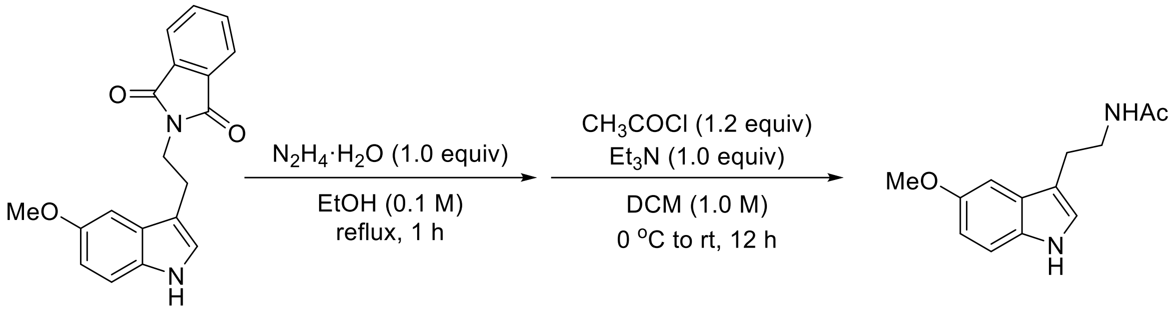

11 **12**

In a 120 °C oven-dried 50-mL round-bottom flask, equipped with a stir bar, was charged with **11** (334 mg, 1.00 mmol, 1.00 equiv), hydrazinemonohydrate (110 mg, 2.20 mmol, 2.20 equiv) and EtOH (10.0 mL) were added under nitrogen. The mixture was stirred at reflux for 1 h. After the completion of reaction, H_2O (50.0 mL) was added and the mixture was extracted with DCM (50.0 mL × 3). The combined organic layers were washed with brine (10.0 mL), dried over Na_2SO_4 and filtrated. The solvent was removed by rotary evaporation and was directly used without further purification.

A 120 °C oven-dried 25-mL round-bottom flask, equipped with a stir bar, was charged with the crude 2-(5-methoxy-*1H*-indol-3-yl)ethan-1-amine, Et_3N (101 mg, 1.00 mmol, 1.00 equiv) and DCM (1.00 mL) were added under nitrogen. The mixture was cooled to 0 °C and CH_3COCl (94.2 mg, 1.20 mmol, 1.20 equiv) was added dropwise. The reaction mixture was allowed to warm to room temperature and stirred for 12 h. After the completum of reaction, H_2O (50.0 mL) was added and the mixture was extracted with DCM (20.0 mL × 3). The combined organic layers were washed with brine (10.0 mL), dried over Na_2SO_4 and filtrated. The solvent was removed by rotary evaporation and the residue was purified by flash silica gel chromatography ((Eluent: 1:1 to 0:100 petroleum ether:ethyl acetate)). The product was isolated as white solid (171.7 mg, 73.9 mmol, 73.9% yield). Spectra were consistent with the literature data[30].

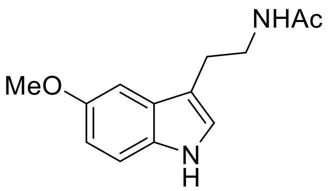

N-(2-(5-methoxy-1*H*-indol-3-yl)ethyl)acetamide (12): ^{1}H NMR** (400 MHz, DMSO) δ 10.65 (1H, s), 7.96 (1H, s), 7.22 (1H, d, J = 8.7 Hz), 7.10 (1H, s), 7.00 (1H, s), 6.70 (1H, d, J = 8.9 Hz), 3.76 (3H, s), 3.32 – 3.27 (2H, m), 2.77 (2H, t, J = 7.5 Hz), 1.80 (3H, s); **^{13}C NMR (101 MHz, DMSO)** δ 178.5, 162.5, 140.9, 137.1, 132.8, 121.5, 121.2, 120.6, 109.6, 64.8, 48.8, 34.8, 32.2.

10. Mechanistic studies

10.1 The experimental procedure for capturing radicals with TEMPO

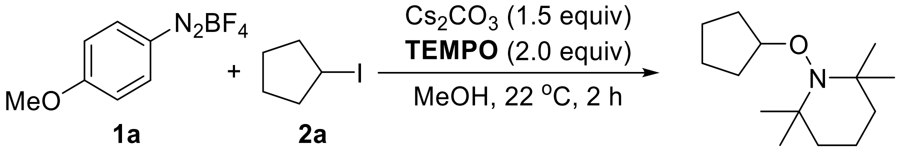

Chemical Formula: $C_{14}H_{27}NO$
Molecular Weight: 225.3760

A 25-mL glass vial fitted with a stirring bar was charged with **1a** (66.6 mg, 0.300 mmol, 3.00 equiv), 2,2,6,6-tetramethyl-1-piperidinyloxy (TEMPO) (32.3 mg, 0.200 mmol, 2.00 equiv) and Cs_2CO_3 (48.9 mg, 0.150 mmol, 1.50 equiv). The mixture was evacuated and backfilled with nitrogen for three times. Then **2a** (19.6 mg, 0.100 mmol, 1.00 equiv) and MeOH (1.00 mL) were added in one portion under N_2. The mixture was allowed to stir for 2 h at 22 °C. After the completion of the reaction, the reaction mixture was poured into H_2O (50.0 mL) and extracted with EtOAc (20.0 mL × 3). The combined organic layers were washed with H_2O (20.0 mL), dried over Na_2SO_4 and filtered. The solvent was removed by rotary evaporation, the crude mixture was subjected to ^{1}H NMR spectroscopy in the presence of CH_2Br_2 (17.4 mg, 0.100 mmol). Then, a TEMPO trapped compound was detected by GC-MS.

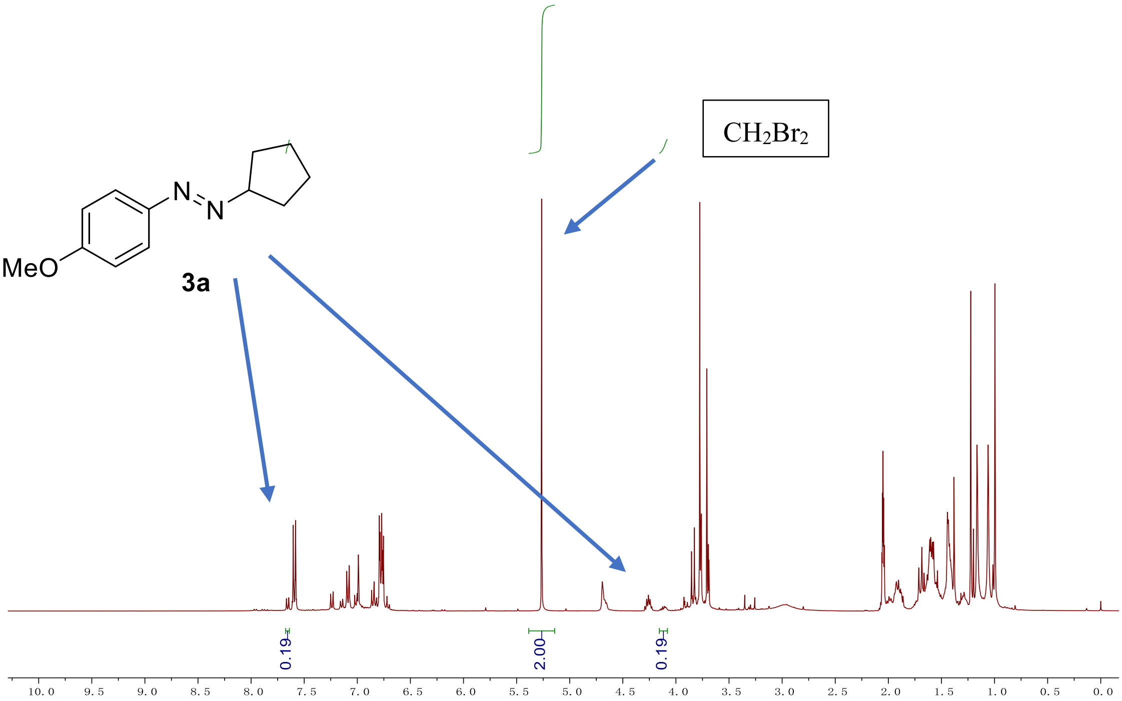

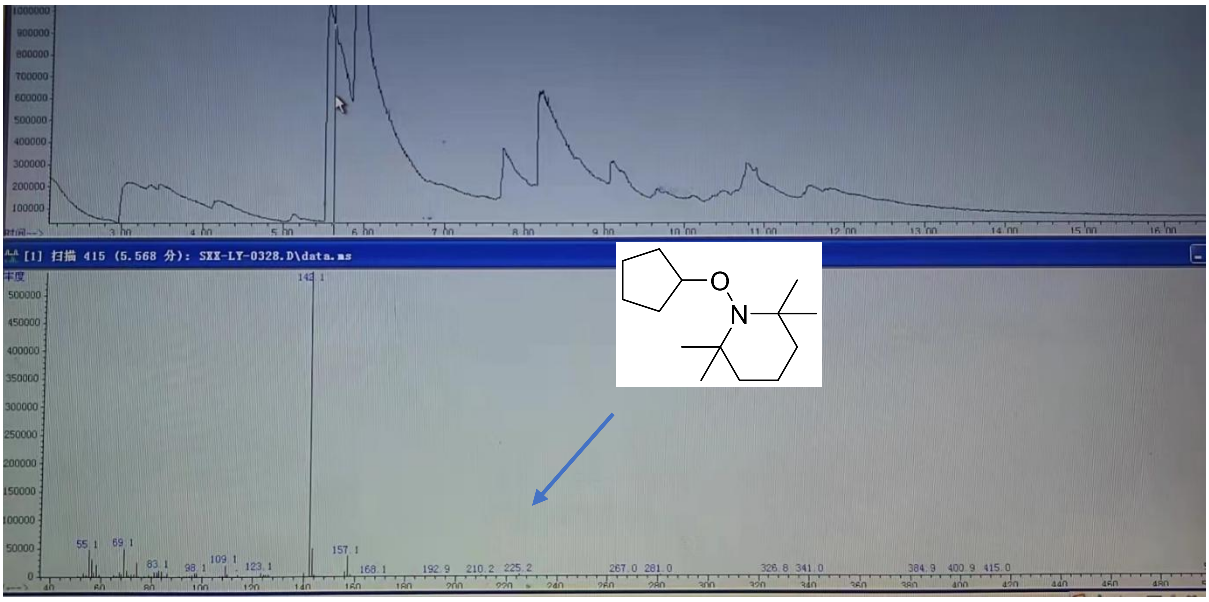

Supplementary Fig. 7. TEMPO trapped compound was detected by GC-MS in the reaction.[54]

10.2 Ring-close experiment

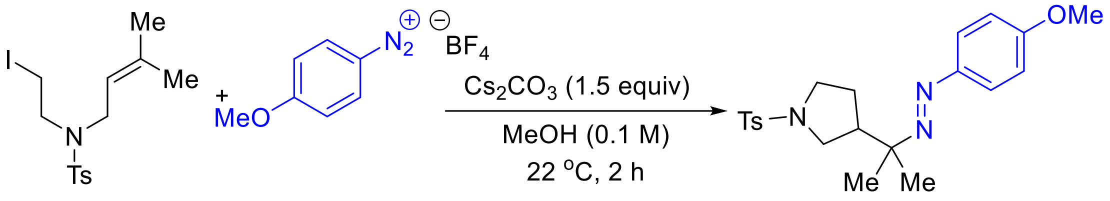

13 1a 14

***tert*-butyl (*E*)-(3-((4-methoxyphenyl)diazenyl)butyl)(tosyl)carbamate (14)**: Prepared according to **General Method F** (Eluent: 100:0 to 50:1 petroleum ether: ethyl acetate) and the title compound was isolated as a yellow solid (111.9 mg, 0.279 mmol, 55.8% yield). **M.p.** = 102.7-103.8 °C. **IR** (thin film) 1604 (w), 1521 (w), 1337 (m), 1247 (s), 1159 (s), 1104 (m), 1019 (m), 834 (m), 661 (s) cm^{-1}; ^{1}**H NMR** (400 MHz, CD_3COCD_3) δ 7.71 (2H, d, J = 8.2 Hz), 7.55 (2H, d, J = 8.9 Hz), 7.40 (2H, d, J = 8.0 Hz), 7.02 (2H, d, J = 8.9 Hz), 3.88 (3H, s), 3.46 – 3.41 (1H, m), 3.32 – 3.27 (1H, m), 3.20 – 3.10 (2H, m), 2.59 – 2.49 (1H, m), 2.41 (3H, s), 1.97 – 1.88 (1H, m), 1.70 – 1.61 (1H, m),1.17 (6H, s). ^{13}**C NMR** (101 MHz, CD_3COCD_3) δ 163.3, 147.6, 144.9, 135.3, 131.2, 129.3, 125.3, 115.6, 70.5, 56.7, 50.2, 49.6, 49.5, 27.8, 25.0, 24.2, 22.2. HRMS (ESI$^+$) [M+Na]$^+$ calc'd for $C_{21}H_{27}N_3O_3S$: 424.1665, found: 424.1665.

10.3 Ring-open experiment

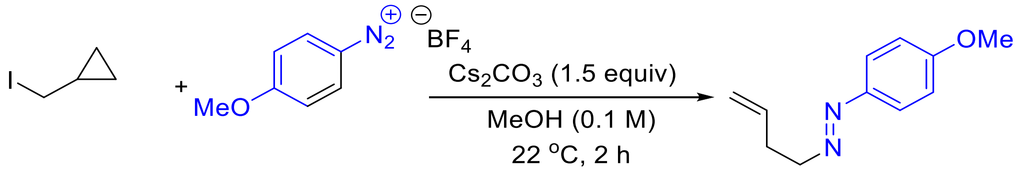

15 1a 16

***tert*-butyl (*E*)-(3-((4-methoxyphenyl)diazenyl)butyl)(tosyl)carbamate (16)**: Prepared according to **General Method F** (Eluent: 100:0 to 50:1 petroleum ether: ethyl acetate) and the title compound was isolated as a yellow solid (23.5 mg, 0.123 mmol, 24.7% yield). **IR** (thin film) 1603 (w), 1518 (m), 1249 (s), 1145 (w), 1104 (m), 1032 (m), 836 (m) cm^{-1}; ^{1}**H NMR** (400 MHz, CD_3COCD_3) δ 7.67 (2H, d, J = 9.0 Hz), 7.03 (2H, d, J = 9.0 Hz), 5.98 – 5.87 (1H, m), 5.16 – 5.00 (2H, m), 4.05 (2H, t, J = 7.3 Hz), 2.66 – 2.59 (2H, m). ^{13}**C NMR** (101 MHz,

CD_3COCD_3) δ 161.7, 146.2, 136.1, 123.8, 115.6, 114.0, 67.9, 55.0, 32.0. HRMS (ESI^+) $[M+H]^+$ calc'd for $C_{11}H_{15}N_2O$: 191.1179, found: 191.1181.

10.4 Control experiment

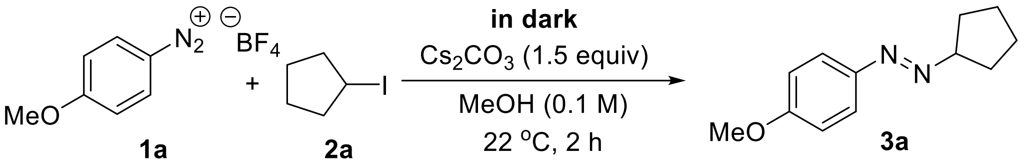

A 25-mL glass vial fitted with a stirring bar was charged with 1a (66.6 mg, 0.300 mmol, 3.00 equiv) and Cs_2CO_3 (48.9 mg, 0.150 mmol, 1.50 equiv). The mixture was evacuated and backfilled with nitrogen for three times. Then 2a (19.6 mg, 0.100 mmol, 1.00 equiv) and MeOH (1.00 mL) were added in one portion under N_2. The mixture was allowed to stir for 2 h at 22 °C in dark. After the completion of the reaction, the reaction mixture was poured into H_2O (50.0 mL) and extracted with EtOAc (20.0 mL × 3). The combined organic layers were washed with H_2O (20.0 mL), dried over Na_2SO_4 and filtered. The solvent was removed by rotary evaporation, the crude mixture was subjected to ^1H NMR spectroscopy in the presence of CH_2Br_2 (17.4 mg, 0.100 mmol). Crude yield of 3a is >95%.

10.5 Isolation of side-product

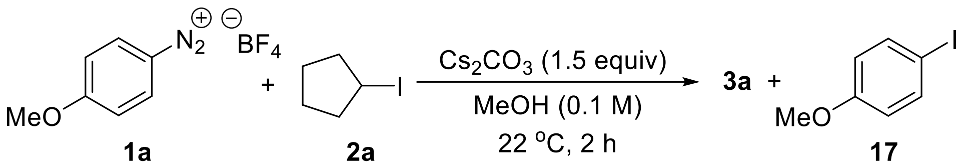

A 25-mL glass vial fitted with a stirring bar was charged with **1a** (66.6 mg, 0.300 mmol, 3.00 equiv) and Cs_2CO_3 (48.9 mg, 0.150 mmol, 1.50 equiv). The mixture was evacuated and backfilled with nitrogen for three times. Then **2a** (19.6 mg, 0.100 mmol, 1.00 equiv) and MeOH (1.00 mL) were added in one portion under N_2. The mixture was allowed to stir for 2 h at 22 °C. After the completion of the reaction, the reaction mixture was poured into H_2O (50.0 mL) and extracted with EtOAc (20.0 mL × 3). The combined organic layers were washed with H_2O (20.0 mL), dried over Na_2SO_4 and filtered. The solvent was removed by rotary evaporation, the crude mixture was subjected to ^1H NMR spectroscopy in the presence of

CH_2Br_2 (0.200 mmol). Crude ^{1}H NMR yield of **17** is 71%.

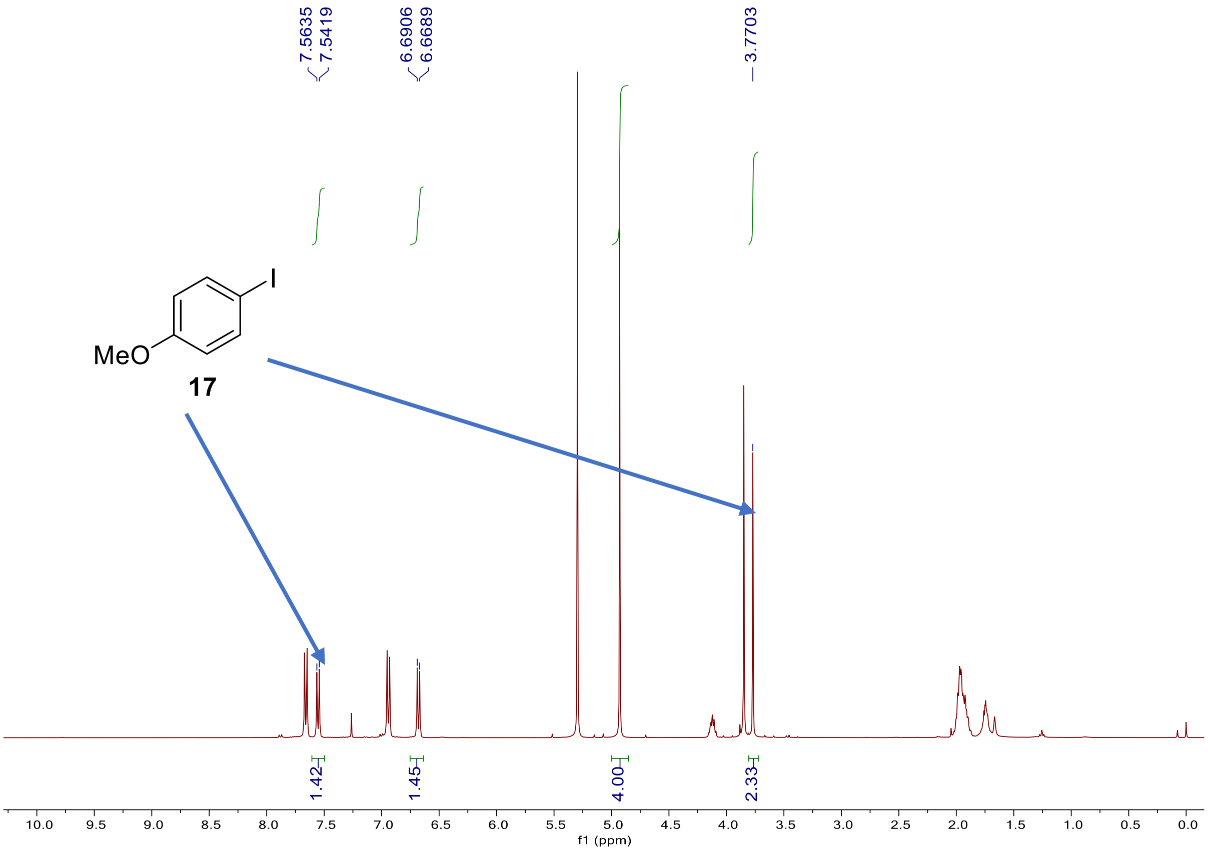

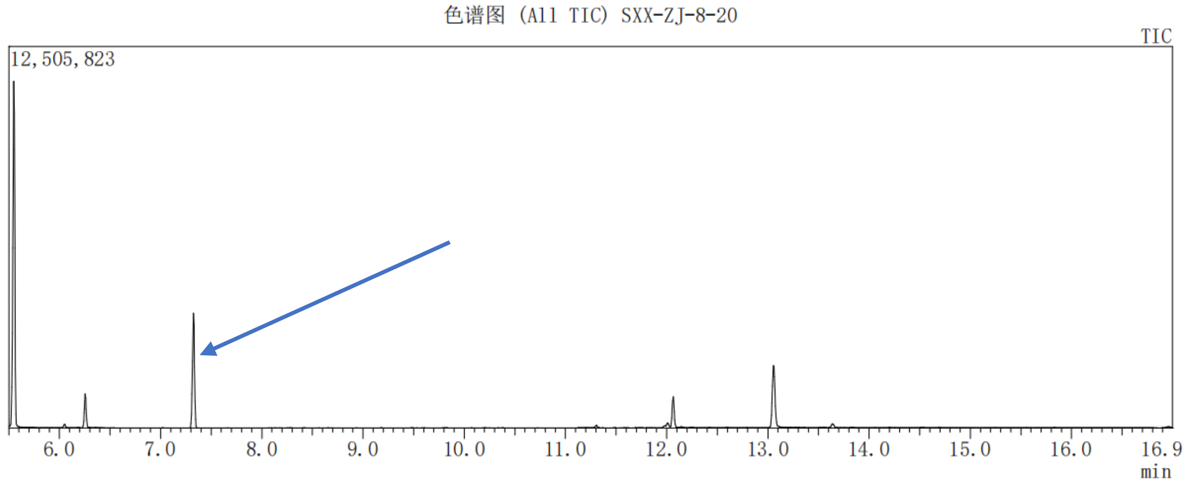

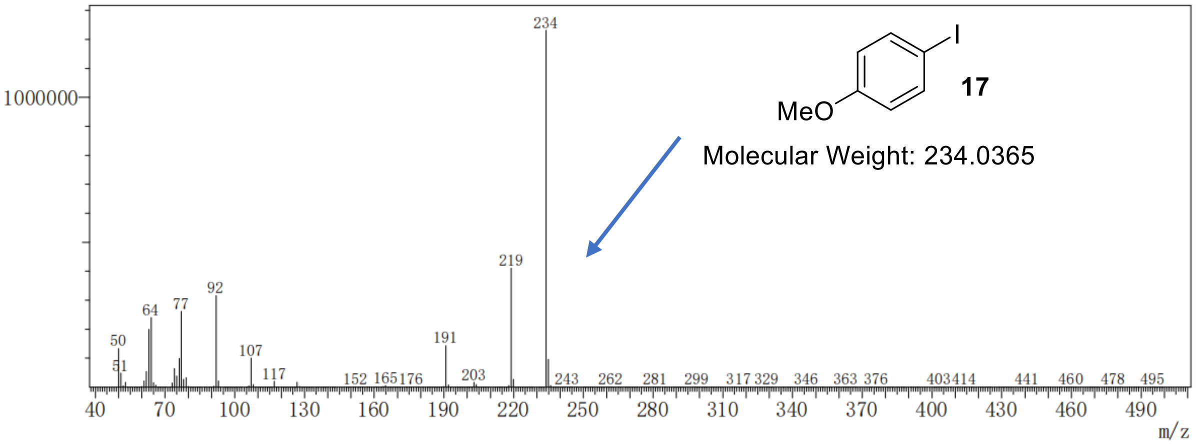

Supplementary Fig. 8. Compound 17 was detected by GC-MS in the reaction.

10.6 Dizaoether 18 was used directly in the coupling with alkyl iodide 1a.

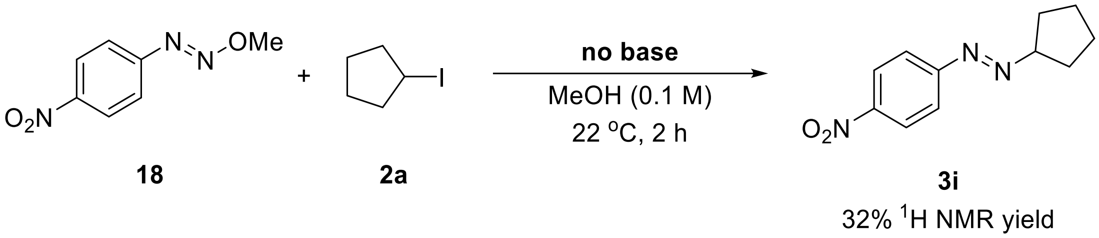

A 25-mL glass vial fitted with a stirring bar was charged with **18** (27.2 mg, 0.150 mmol, 1.50 equiv). The mixture was evacuated and backfilled with nitrogen for three times. Then **2a** (19.6 mg, 0.100 mmol, 1.00 equiv) and MeOH (1.00 mL) were added under nitrogen. The mixture was allowed to stir for 2 h at 22 °C. After the completion of the reaction, the reaction mixture was poured into H_2O (50.0 mL) and extracted with EtOAc (20.0 mL × 3). The combined organic layers were washed with H_2O (20.0 mL), dried over Na_2SO_4 and filtered. The solvent was removed by rotary evaporation, the crude mixture was subjected to ^1H NMR spectroscopy in the presence of CH_2Br_2 (17.4 mg, 0.100 mmol). Crude yield of **3i** is 32%. Then, compound **3i** was detected by GC-MS.

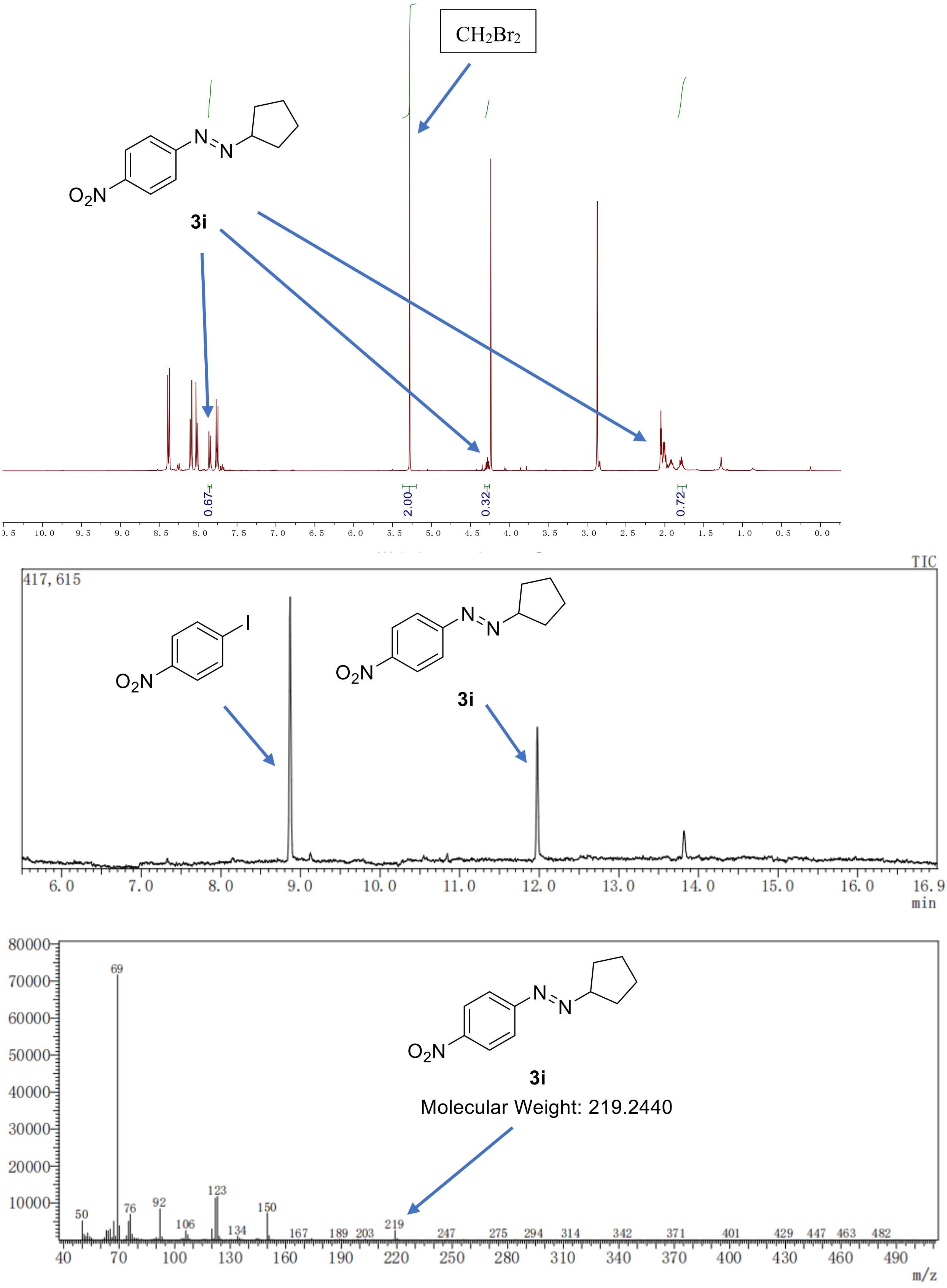

Supplementary Fig. 9. Compound 3i was detected by GC-MS in the reaction.

10.7 Detection of another side-product: aldehyde.

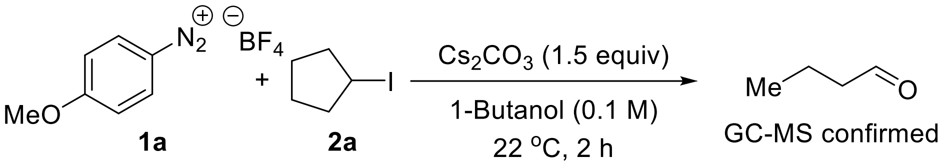

A 25-mL glass vial fitted with a stirring bar was charged with **1a** (66.6 mg, 0.300 mmol, 3.00 equiv) and Cs_2CO_3 (48.9 mg, 0.150 mmol, 1.50 equiv). The mixture was evacuated and backfilled with nitrogen for three times. Then **2a** (19.6 mg, 0.100 mmol, 1.00 equiv) and 1-Butanol (1.00 mL) were added in one portion under N_2. The mixture was allowed to stir for 2 h at 22 °C. Butyraldehyde was detected by GC-MS. Combination with the results of EPR experiments, the methoxyl radical (if the solvent is MeOH) might be oxidized by the active arylalkyl-azadiazene radical cations, thus providing the final product. However

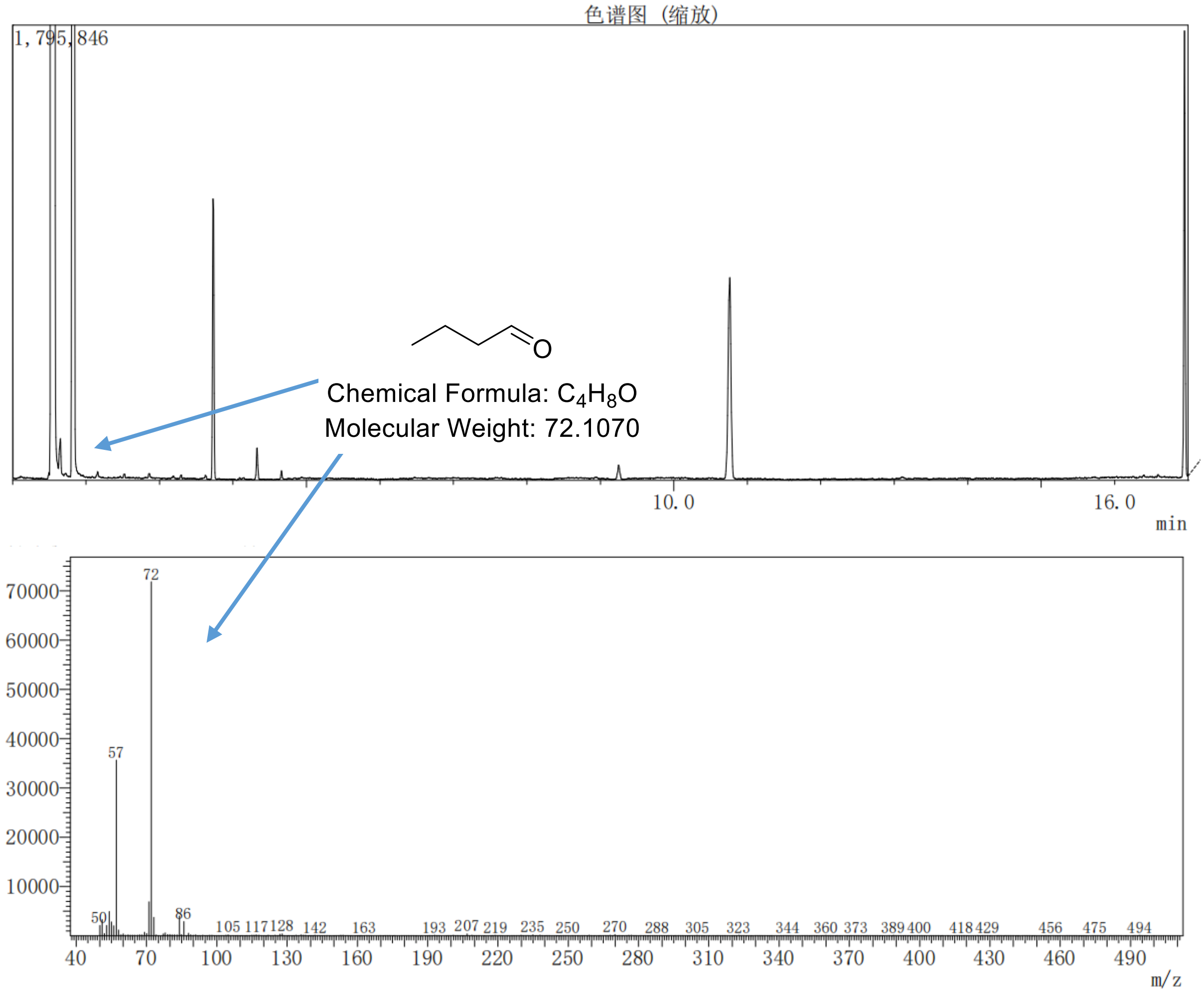

Supplementary Fig. 10. Butyraldehyde was detected by GC-MS in the reaction.

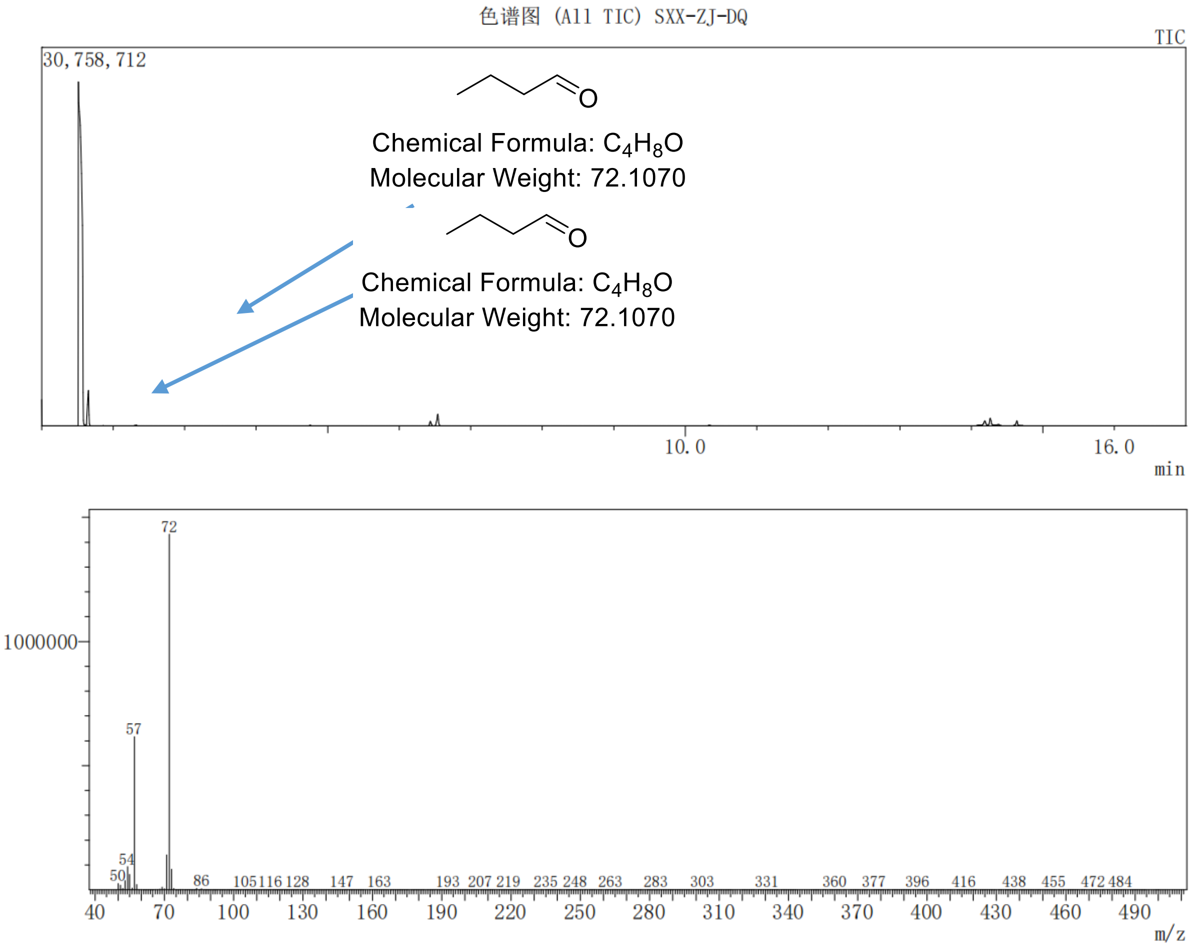

Supplementary Fig. 11. Butyraldehyde by GC-MS.

10.8 Detection of (*E*)-1,2-bis(4-methoxyphenyl)diazene.

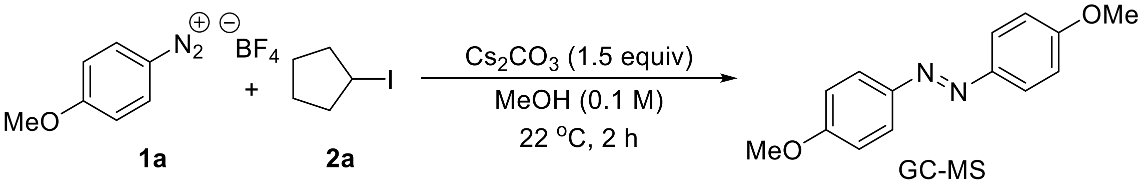

A 25-mL glass vial fitted with a stirring bar was charged with **1a** (66.6 mg, 0.150 mmol, 3.00 equiv) and Cs_2CO_3 (48.9 mg, 0.150 mmol, 1.50 equiv). The mixture was evacuated and backfilled with nitrogen three times. Then the **2a** (19.6 mg, 0.100 mmol, 1.00 equiv) and MeOH (1.00 mL) were added under nitrogen. The mixture was allowed to stir at 22 °C for 2 h. Then, (*E*)-1,2-bis(4-methoxyphenyl)diazene was detected by GC-MS.

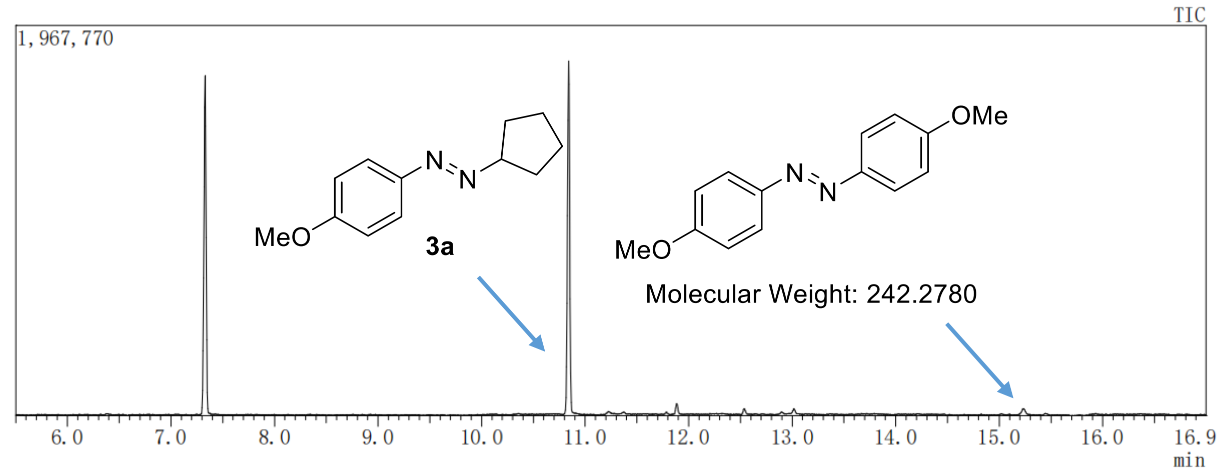

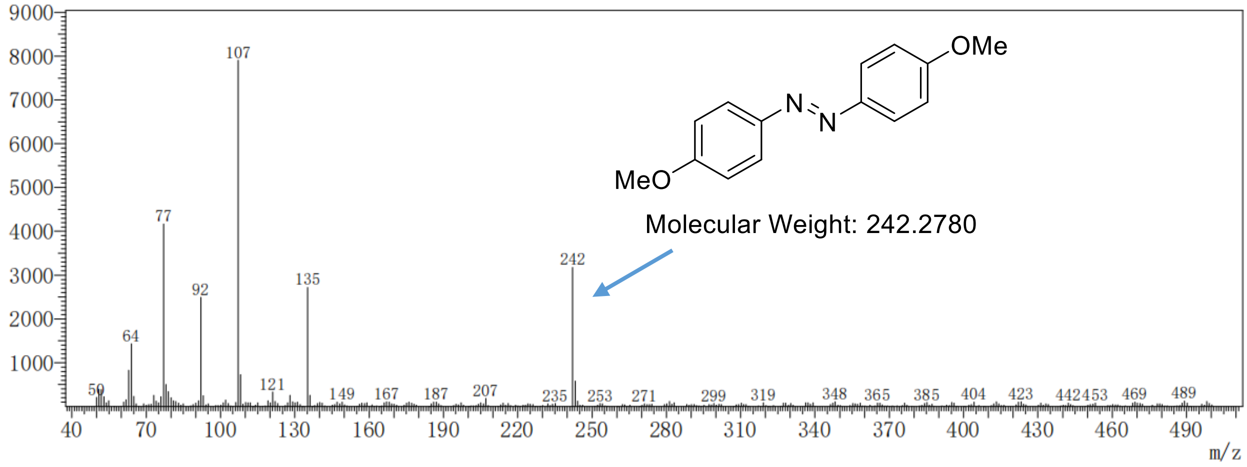

Supplementary Fig. 12. (*E*)-1,2-bis(4-methoxyphenyl)diazene was detected by GC-MS in the reaction.

10.9 Other alkyl halides' reaction.

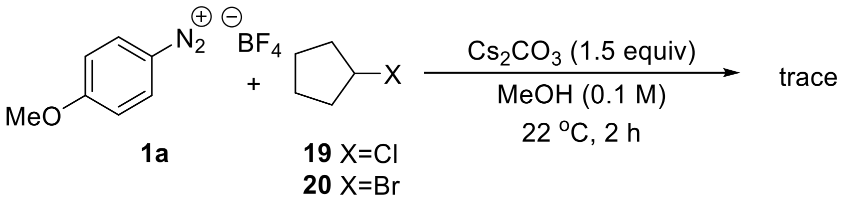

A 25-mL glass vial fitted with a stirring bar was charged with **1a** (66.6 mg, 0.150 mmol, 3.00 equiv) and Cs_2CO_3 (48.9 mg, 0.150 mmol, 1.50 equiv). The mixture was evacuated and backfilled with nitrogen three times. Then the **19**, **20** (0.100 mmol, 1.00 equiv) and MeOH (1.00 mL) were added under nitrogen. The mixture was allowed to stir at 22 °C for 2 h.After the completion of the reaction, the reaction mixture was poured into H_2O (50.0 mL) and extracted with EtOAc (20.0 mL × 3). The combined organic layers were washed with H_2O (20.0 mL), dried over Na_2SO_4 and filtered.

The solvent was removed by rotary evaporation, the crude mixture was subjected to ^{1}H NMR spectroscopy in the presence of CH_2Br_2 (17.4 mg, 0.100 mmol). No desired product with the full recovery of the starting alkyl halides was detected according to ^{1}H NMR.

11. The UV-vis absorption spectra of the mixtures of 1a, 2a, Cs_2CO_3

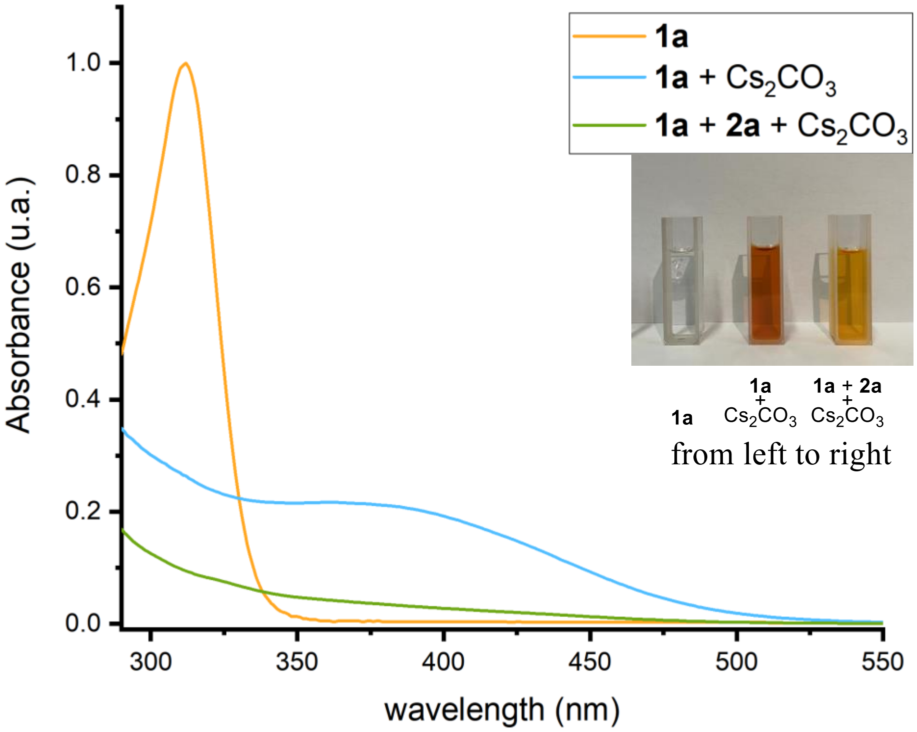

Supplementary Fig. 13. The combined UV-vis absorption spectra of 1a, the 1: 1 mixture of 1a and Cs_2CO_3, and the 1: 1: 1 mixture of 1a, 2a and Cs_2CO_3 in MeOH (c = 0.001 M).

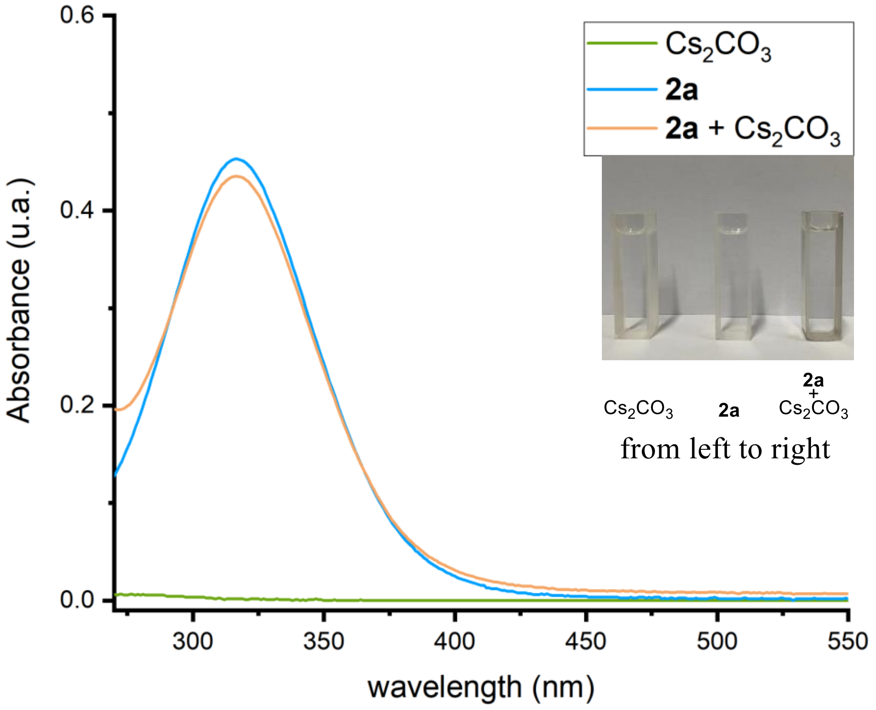

Supplementary Fig. 14. The combined UV-vis absorption spectra of Cs_2CO_3, 2a, and the 1: 1 mixture of 2a and Cs_2CO_3 in MeOH (c = 0.001 M).

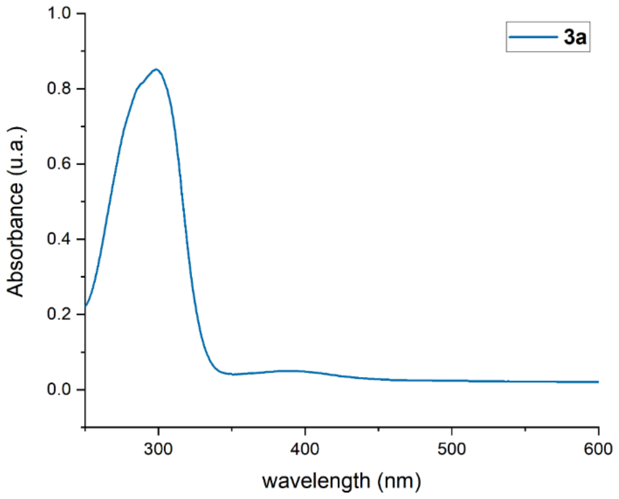

Supplementary Fig. 15. The combined UV-vis absorption spectra of 3a in MeOH (c = 0.001 M).

12. Computational studies

Computational details:

All calculations were performed using Gaussian 16, Revision A.03 package.[31] All of the reactants, intermediates, transition states, products were optimized by the DFT with the ωB97X-D functional.[32] For geometry optimizations and frequency calculations, BS-I basis set system was employed. In BS-I, we employed SDD basis set for I and Cs with effective core potentials, 6-31G(d) basis sets for C, O, H, N, B and F. All the stationary structures were characterized with no imaginary frequency and the transition state structures (TSs) were characterized with a single imaginary frequency. Intrinsic reaction coordinate (IRC) calculations were performed on the TSs. The solvent effect of methanol was evaluated through the SMD method,[33] in which a better basis system BS-II was used. In BS-II, we employed def2-tzvp basis set for I, Cs, C, O, H, N, B and F. All reported energies are free energies at a concentration of 1 M (For more details, see Supplementary Data 1).

Part A:

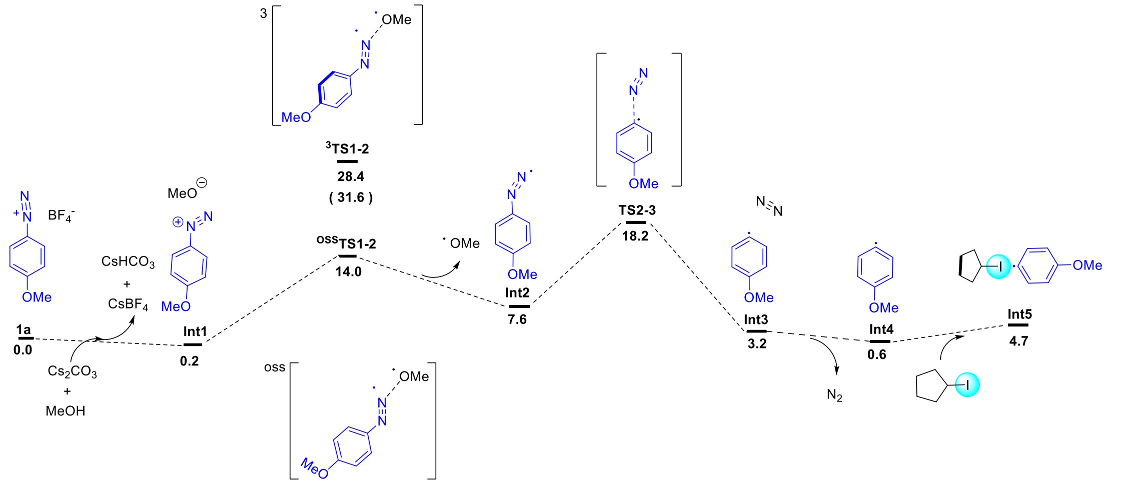

Part B (Continued to part A):

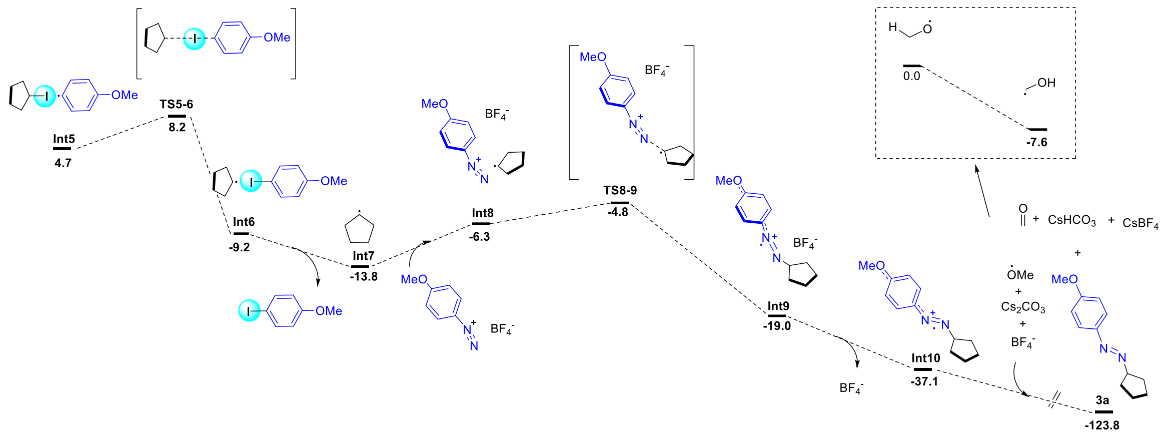

Supplementary Fig. 16. DFT supported pathway

13. Disscussion on the mechanism.

Based on the experimental information and related reference provided in the main manuscript, the diazoethers from original diazonium salts could be formed in alcoholic media (MeOH is our case) under basic conditions and might be a reasonable key intermediate for the reaction. However, diazoanhydride (ArN_2ON_2Ar) could also go through homolytic cleavage of "N-O" bond to generate the aryl radical. At this time, this specie could not be ruled out. The active alkyl radical and aryl iodide were formed efficiently via the abstraction of an iodine atom from an alkyl iodide by aryl radical, which was known as XAT process. Another key factor is the quenching of the nitrogen radical cation generated by the combination of alkyl radical and the diazonium salts. Since the methoxyl radical was oxidative and it could abstract a hydrogen from the MeOH to generate an important hydroxymethyl radical which could finally quench the radical cation. Moreover, the corresponding aldehyde was also detected in the GC-MS after the reaction. Additive DFT calculation was also conducted to support the mechanism we proposed. It was found that most of the steps were favorable especially on the XAT process. Thus, the reaction mechanism was showed as follows:

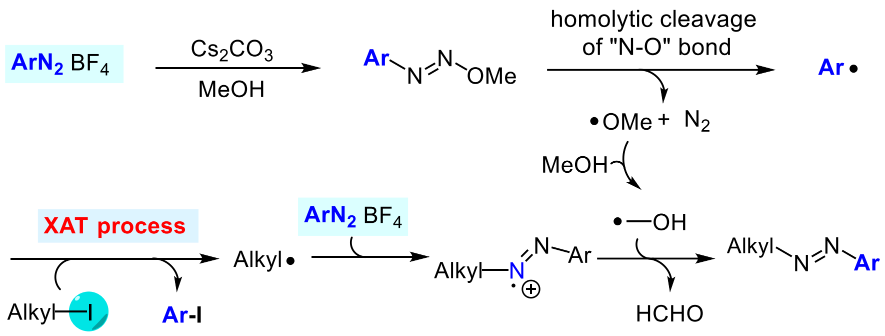

Supplementary Fig. 17. Proposed mechanism

14. Crystallographic data

Light yellow crystals of **4s** were slowly grown from *d*$_6$-acetone solution of the compound at 22 °C. For X-ray structure analyses, the oil-coated crystals were mounted onto a loop, and the diffraction data were collected on a Bruker Smart Apex II CCD diffractometer with graphite-monochromated Mo Kα (λ = 0.71073 Å). An empirical (multi-scan) absorption correction was applied with the program SADABS. The structures were solved by Olex2 with the ShelXT solution program using the intrinsic phasing method and subsequently refined on F2 by using full-matrix least-squares techniques (SHELXL-2014). If not noted otherwise, all non-hydrogen atoms were refined anisotropically, and hydrogen atoms were located at calculated positions or found in the ΔF map. Figures of the solid-state molecular structures were generated using XP as implemented in the SHELXTL program.

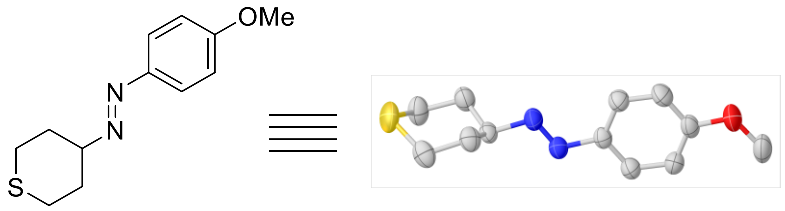

Supplementary Fig. 18. ORTEP drawing of 4s with 50% thermal ellipsoid.

Table 1 Crystal data and structure refinement for mo220223a (CCDC 2181806).

**Identification code**	mo220223a
**Empirical formula**	$C_{12}H_{16}N_2OS$
**Formula weight**	236.33
**Temperature/K**	296.15
**Crystal system**	monoclinic
**Space group**	$P2_1/n$
**a/Å**	14.0646(19)
**b/Å**	5.5477(7)
**c/Å**	16.038(2)
**α/°**	90
**β/°**	92.135(3)

**γ/°**	90
**Volume/Å^3**	1250.5(3)
**Z**	4
**$ρ_{calc}$g/cm^3**	1.255
**μ/mm^{-1}**	0.240
**F(000)**	504.0
**Crystal size/mm^3**	0.15 × 0.12 × 0.12
**Radiation**	MoKα (λ = 0.71073)
**2Θ range for data collection/°**	3.782 to 60.962
**Index ranges**	-19 ≤ h ≤ 19, -7 ≤ k ≤ 7, -21 ≤ l ≤ 22
**Reflections collected**	12951
**Independent reflections**	3511 [R_{int} = 0.0356, R_{sigma} = 0.0352]
**Data/restraints/parameters**	3511/0/146
**Goodness-of-fit on F^2**	1.014
**Final R indexes [I>=2σ (I)]**	R_1 = 0.0465, wR_2 = 0.1080
**Final R indexes [all data]**	R_1 = 0.0870, wR_2 = 0.1287
**Largest diff. peak/hole / e Å^{-3}**	0.20/-0.34

Table 1 Crystal data and structure refinement for mo220223a.

**Identification code**	mo220223a
**Empirical formula**	$C_{12}H_{16}N_2OS$
**Formula weight**	236.33
**Temperature/K**	296.15
**Crystal system**	monoclinic
**Space group**	$P2_1/n$

**a/Å**	14.0646(19)
**b/Å**	5.5477(7)
**c/Å**	16.038(2)
**α/°**	90
**β/°**	92.135(3)
**γ/°**	90
**Volume/Å^3**	1250.5(3)
**Z**	4
**ρ_{calc}g/cm^3**	1.255
**μ/mm^{-1}**	0.240
**F(000)**	504.0
**Crystal size/mm^3**	0.15 × 0.12 × 0.12
**Radiation**	MoKα (λ = 0.71073)
**2Θ range for data collection/°**	3.782 to 60.962
**Index ranges**	-19 ≤ h ≤ 19, -7 ≤ k ≤ 7, -21 ≤ l ≤ 22
**Reflections collected**	12951
**Independent reflections**	3511 [R_{int} = 0.0356, R_{sigma} = 0.0352]
**Data/restraints/parameters**	3511/0/146
**Goodness-of-fit on F^2**	1.014
**Final R indexes [I>=2σ (I)]**	R_1 = 0.0465, wR_2 = 0.1080
**Final R indexes [all data]**	R_1 = 0.0870, wR_2 = 0.1287
**Largest diff. peak/hole / e Å^{-3}**	0.20/-0.34

Table 2 Fractional Atomic Coordinates (×10^4) and Equivalent Isotropic Displacement Parameters (Å^2×10^3) for mo220223a.

U_{eq} is defined as 1/3 of the trace of the orthogonalised U_{IJ} tensor.

**Atom**	***x***	***y***	***z***	**U(eq)**
S1	7107.5(4)	-1023.8(10)	6479.7(4)	73.5(2)

O1	263.0(9)	7542(3)	5759.3(9)	74.0(4)
N1	4325.7(10)	3052(3)	6141.1(9)	53.1(4)
N2	3995.8(9)	4778(2)	6521.1(8)	45.5(3)
C1	5310.1(11)	2481(3)	6432.6(11)	45.9(4)
C2	5937.3(12)	2347(4)	5687.9(11)	58.9(5)
C3	6963.5(13)	1797(4)	5944.6(14)	67.2(5)
C4	6258.8(14)	-584(4)	7269.0(13)	62.0(5)
C5	5283.8(12)	139(3)	6918.1(12)	55.0(4)
C6	3034.9(11)	5421(3)	6288.6(10)	42.5(4)
C7	2453.5(12)	4172(3)	5718.0(11)	53.9(4)
C8	1538.3(13)	4947(4)	5557.0(12)	61.0(5)
C9	1188.3(11)	6954(3)	5956.3(11)	51.1(4)
C10	1762.0(13)	8211(4)	6513.8(11)	54.7(4)
C11	2685.0(12)	7431(3)	6673.7(11)	53.5(4)
C12	-132.4(15)	9596(5)	6145.0(14)	76.4(6)

Table 3 Anisotropic Displacement Parameters (Å^2×10^3) for mo220223a.

The Anisotropic displacement factor exponent takes the form: -2π^2[h^{2}a*^{2}U$_{11}$+2hka*b*U$_{12}$+…].

Atom	U_{11}	U_{22}	U_{33}	U_{23}	U_{13}	U_{12}
S1	53.3(3)	62.8(3)	104.4(5)	6.3(3)	1.4(3)	18.6(2)
O1	38.5(7)	100.7(11)	82.3(9)	-5.6(9)	-7.3(6)	14.0(7)
N1	40.0(7)	54.8(9)	64.0(9)	-10.0(7)	-7.5(6)	5.2(7)
N2	38.2(7)	44.3(8)	53.8(8)	2.0(6)	-0.6(6)	-0.6(6)
C1	35.1(8)	45.4(9)	56.6(9)	-5.6(7)	-6.8(7)	0.4(7)
C2	47.3(10)	71.6(12)	57.5(10)	6.9(9)	-2.4(8)	6.0(9)
C3	45.3(10)	77.4(14)	79.2(13)	16.1(11)	7.1(9)	5.9(9)
C4	57.2(11)	53.3(11)	74.4(12)	15.6(9)	-9.5(9)	-3.5(9)
C5	41.9(9)	53.2(10)	69.9(11)	3.1(9)	-0.1(8)	-7.4(8)
C6	34.8(8)	46.4(9)	46.2(8)	5.6(7)	1.3(6)	-1.2(7)
C7	46.3(9)	53.4(10)	61.5(11)	-8.9(8)	-2.6(8)	1.2(8)
C8	44.2(10)	70.2(12)	67.5(12)	-10.8(10)	-11.2(8)	-3.1(9)
C9	34.1(8)	69.0(11)	50.3(9)	7.4(9)	2.1(7)	2.7(8)
C10	45.9(9)	61.4(11)	56.9(10)	-4.0(9)	3.3(8)	10.5(8)
C11	43.7(9)	58.1(11)	58.1(10)	-8.1(8)	-5.6(8)	3.2(8)
C12	47.5(11)	105.5(18)	76.4(13)	9.8(13)	5.4(10)	26.2(11)

Table 4 Bond Lengths for mo220223a.

Atom	Atom	Length/Å	Atom	Atom	Length/Å
S1	C3	1.793(2)	C2	C3	1.517(3)
S1	C4	1.789(2)	C4	C5	1.517(2)

O1	C9	1.367(2)	C6	C7	1.389(2)
O1	C12	1.420(3)	C6	C11	1.375(2)
N1	N2	1.2346(19)	C7	C8	1.372(2)
N1	C1	1.479(2)	C8	C9	1.384(3)
N2	C6	1.434(2)	C9	C10	1.372(3)
C1	C2	1.513(2)	C10	C11	1.383(2)
C1	C5	1.516(2)			

Table 5 Bond Angles for mo220223a.

Atom	Atom	Atom	Angle/°	Atom	Atom	Atom	Angle/°
C4	S1	C3	98.80(9)	C7	C6	N2	124.99(15)
C9	O1	C12	118.40(16)	C11	C6	N2	115.76(14)
N2	N1	C1	112.00(13)	C11	C6	C7	119.25(15)
N1	N2	C6	115.62(13)	C8	C7	C6	119.52(17)
N1	C1	C2	109.12(14)	C7	C8	C9	120.81(17)
N1	C1	C5	107.79(13)	O1	C9	C8	115.91(16)
C2	C1	C5	112.91(15)	O1	C9	C10	124.11(17)
C1	C2	C3	111.87(15)	C10	C9	C8	119.98(16)
C2	C3	S1	113.24(15)	C9	C10	C11	119.12(17)
C5	C4	S1	113.05(14)	C6	C11	C10	121.32(16)
C1	C5	C4	112.22(14)				

Table 6 Torsion Angles for mo220223a.

A	B	C	D	Angle/°	A	B	C	D	Angle/°
S1	C4	C5	C1	-60.13(19)	C3	S1	C4	C5	52.56(16)
O1	C9	C10	C11	179.11(17)	C4	S1	C3	C2	-52.80(17)
N1	N2	C6	C7	4.0(2)	C5	C1	C2	C3	-60.9(2)
N1	N2	C6	C11	-176.17(15)	C6	C7	C8	C9	0.0(3)
N1	C1	C2	C3	179.24(16)	C7	C6	C11	C10	1.0(3)
N1	C1	C5	C4	-178.38(15)	C7	C8	C9	O1	-178.96(18)
N2	N1	C1	C2	-128.57(16)	C7	C8	C9	C10	0.7(3)
N2	N1	C1	C5	108.47(17)	C8	C9	C10	C11	-0.6(3)
N2	C6	C7	C8	178.97(17)	C9	C10	C11	C6	-0.3(3)
N2	C6	C11	C10	-178.81(16)	C11	C6	C7	C8	-0.8(3)
C1	N1	N2	C6	-178.74(13)	C12	O1	C9	C8	-179.51(18)
C1	C2	C3	S1	60.3(2)	C12	O1	C9	C10	0.8(3)
C2	C1	C5	C4	61.0(2)					

Table 7 Hydrogen Atom Coordinates (Å×10^4) and Isotropic Displacement Parameters (Å^2×10^3) for mo220223a.

Atom	*x*	*y*	*z*	U(eq)
H1	5544	3768	6805	55

H2A	5909	3870	5391	71
H2B	5699	1101	5311	71
H3A	7206	3080	6304	81
H3B	7340	1775	5450	81
H4A	6201	-2064	7585	74
H4B	6491	658	7650	74
H5A	5038	-1130	6554	66
H5B	4854	315	7373	66
H7	2683	2821	5447	65
H8	1149	4113	5175	73
H10	1533	9570	6781	66
H11	3077	8283	7049	64
H12A	-793	9735	5978	115
H12B	201	11016	5978	115
H12C	-73	9426	6740	115

15. NMR spectrum of the products

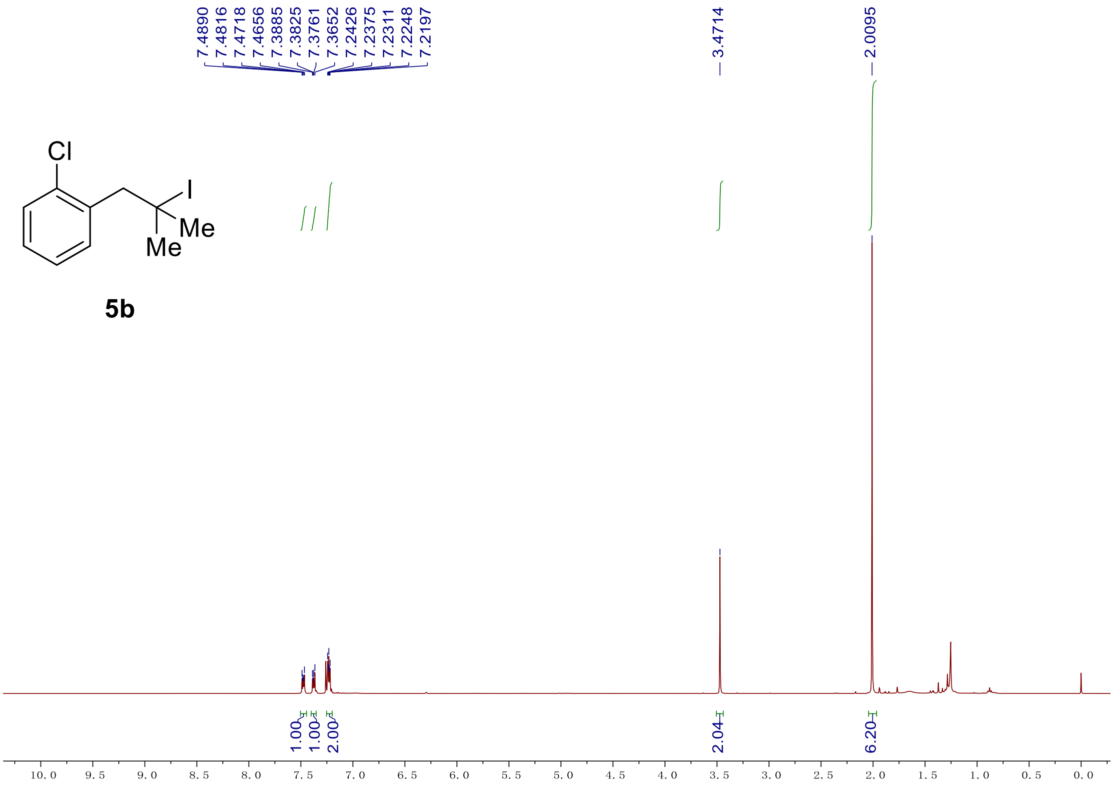

Supplementary Fig. 19. ^{1}H NMR of compound **5b.**

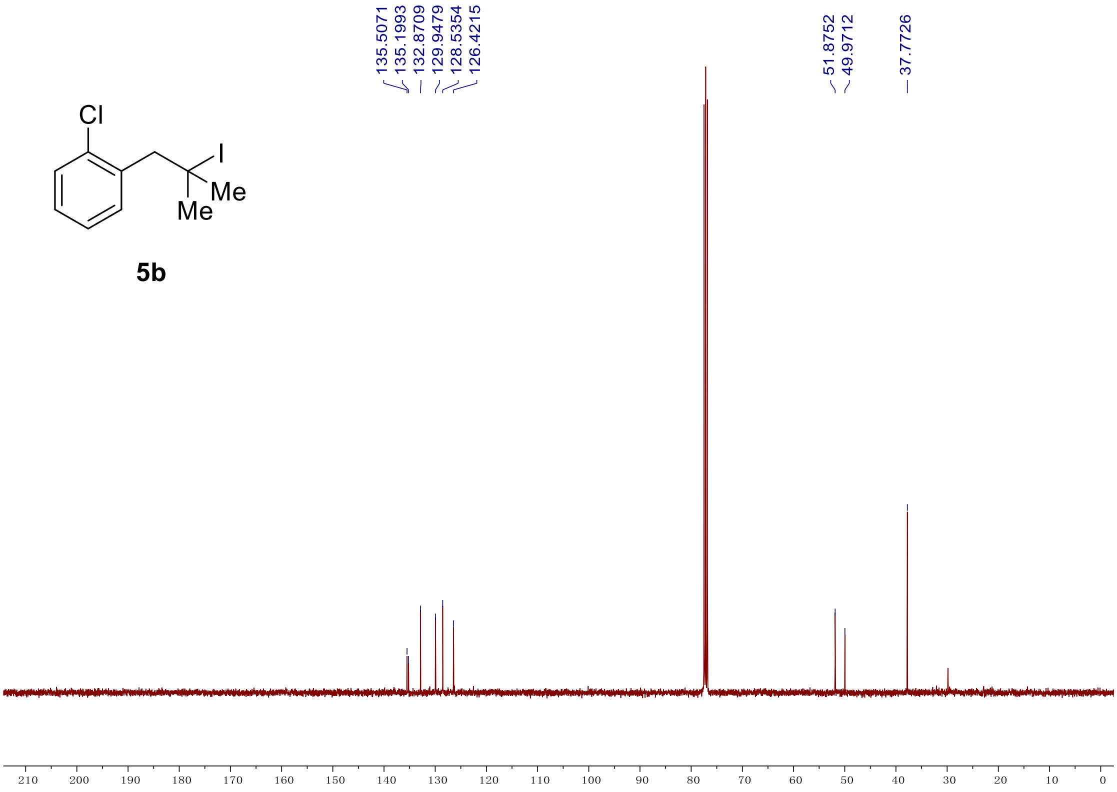

Supplementary Fig. 20. ^{13}C NMR of compound **5b.**

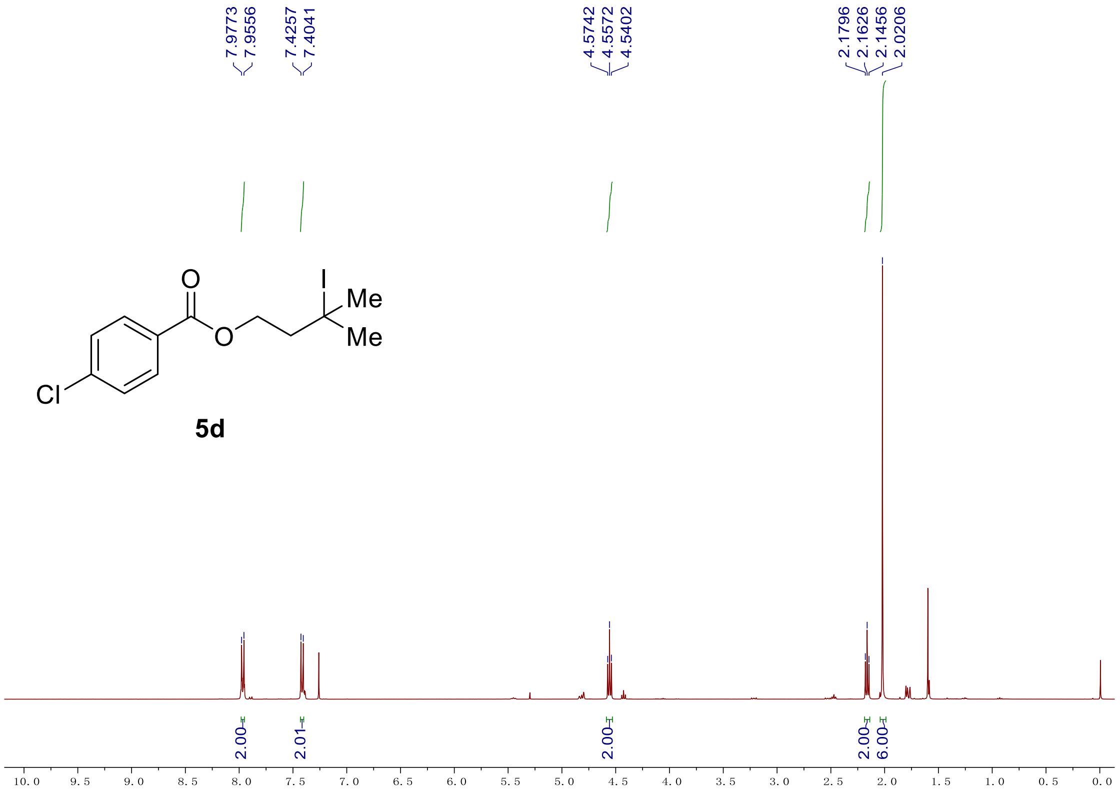

Supplementary Fig. 21. ^{1}H NMR of compound **5d.**

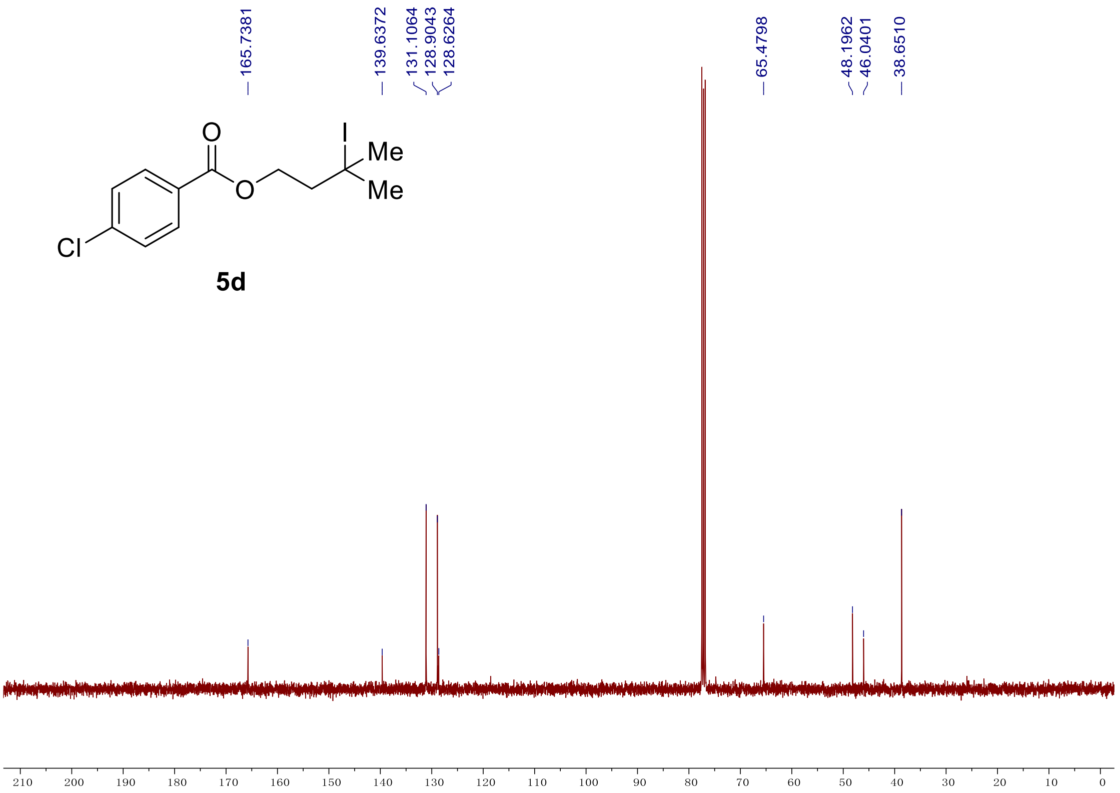

Supplementary Fig. 22. ^{13}C NMR of compound **5d.**

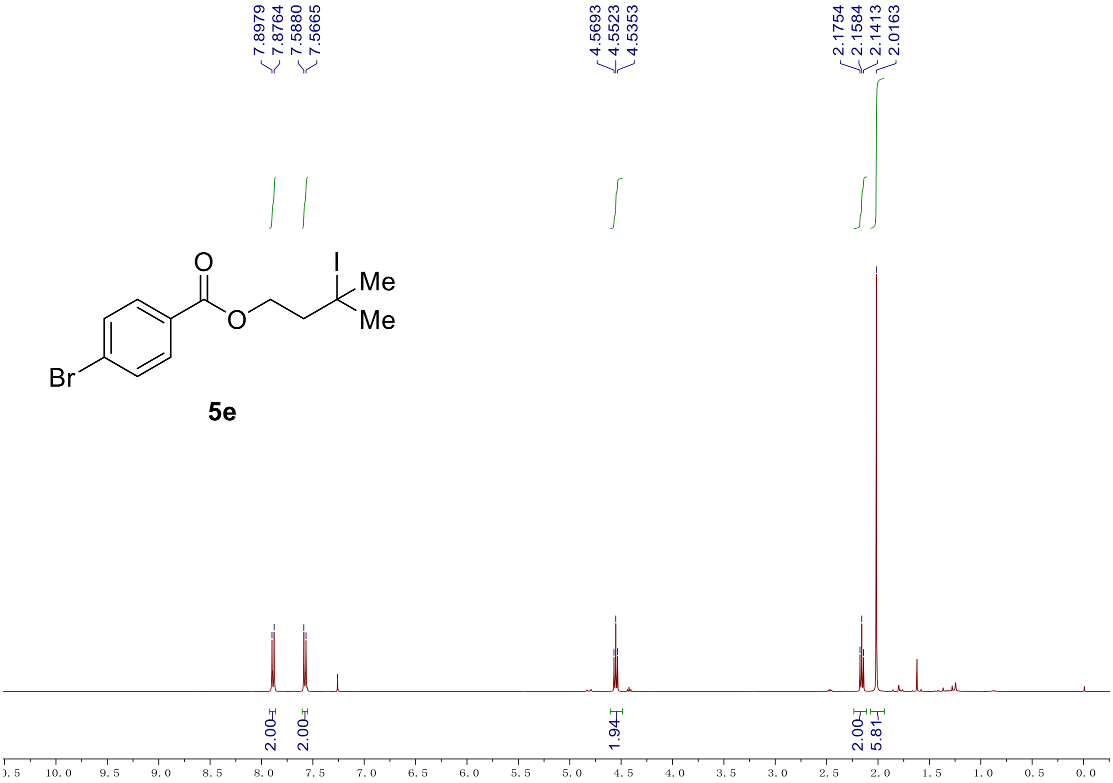

Supplementary Fig. 23. ^{1}H NMR of compound **5e.**

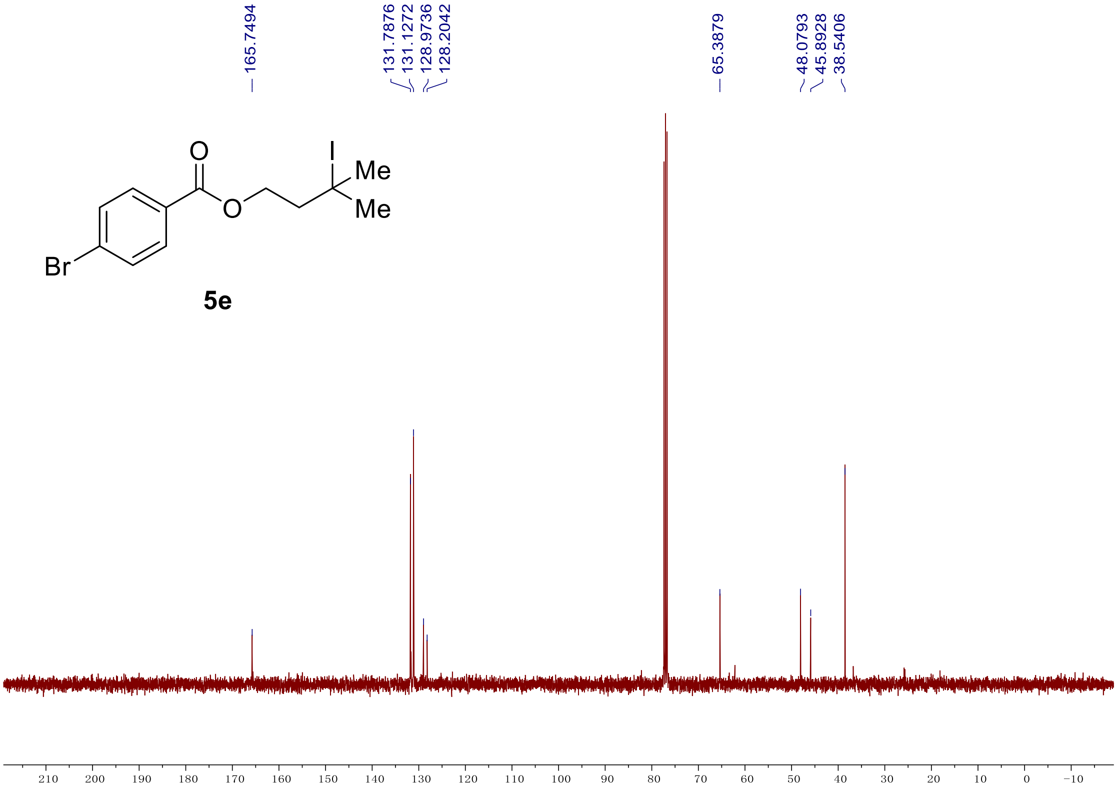

Supplementary Fig. 24. ^{13}C NMR of compound **5e.**

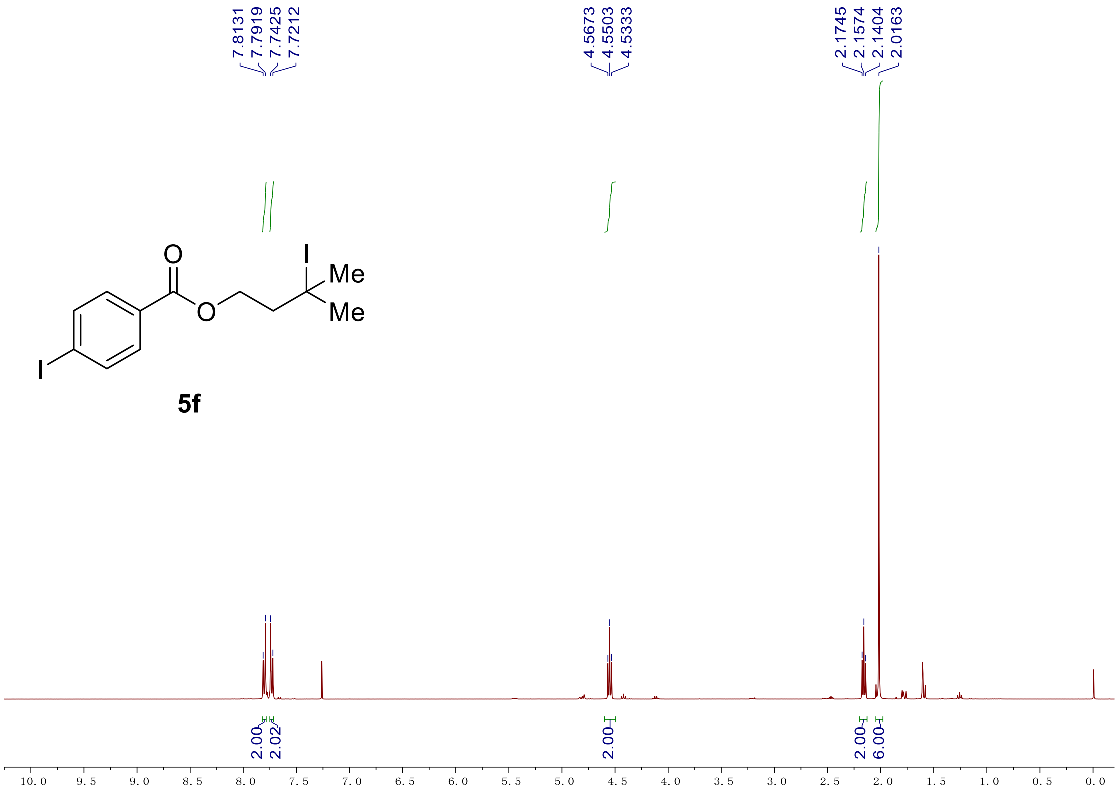

Supplementary Fig. 25. ^{1}H NMR of compound **5f.**

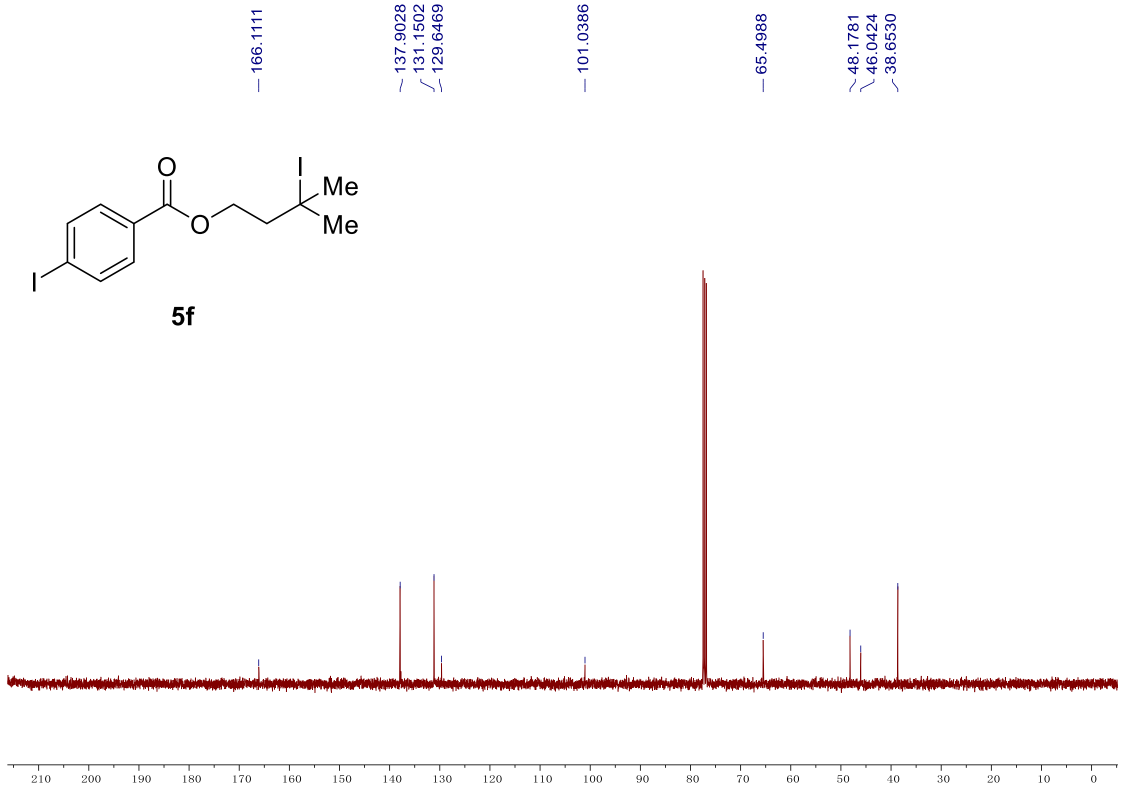

Supplementary Fig. 26. ^{13}C NMR of compound **5f.**

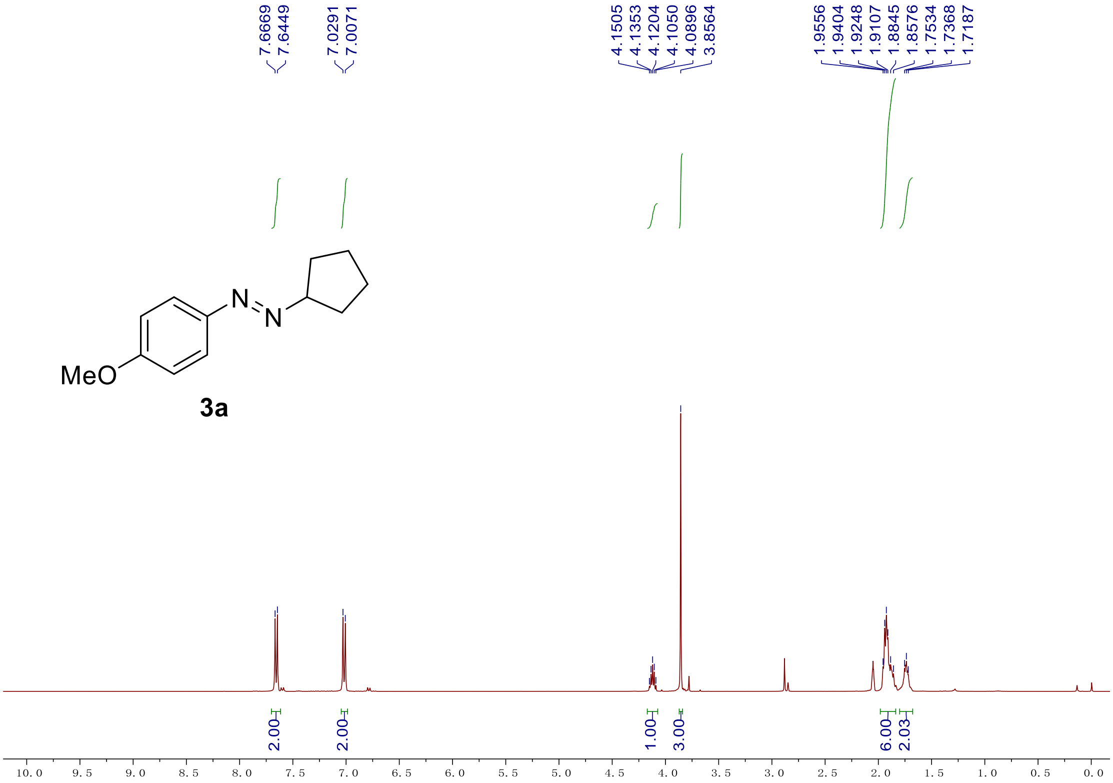

Supplementary Fig. 27. ^{1}H NMR of compound **3a.**

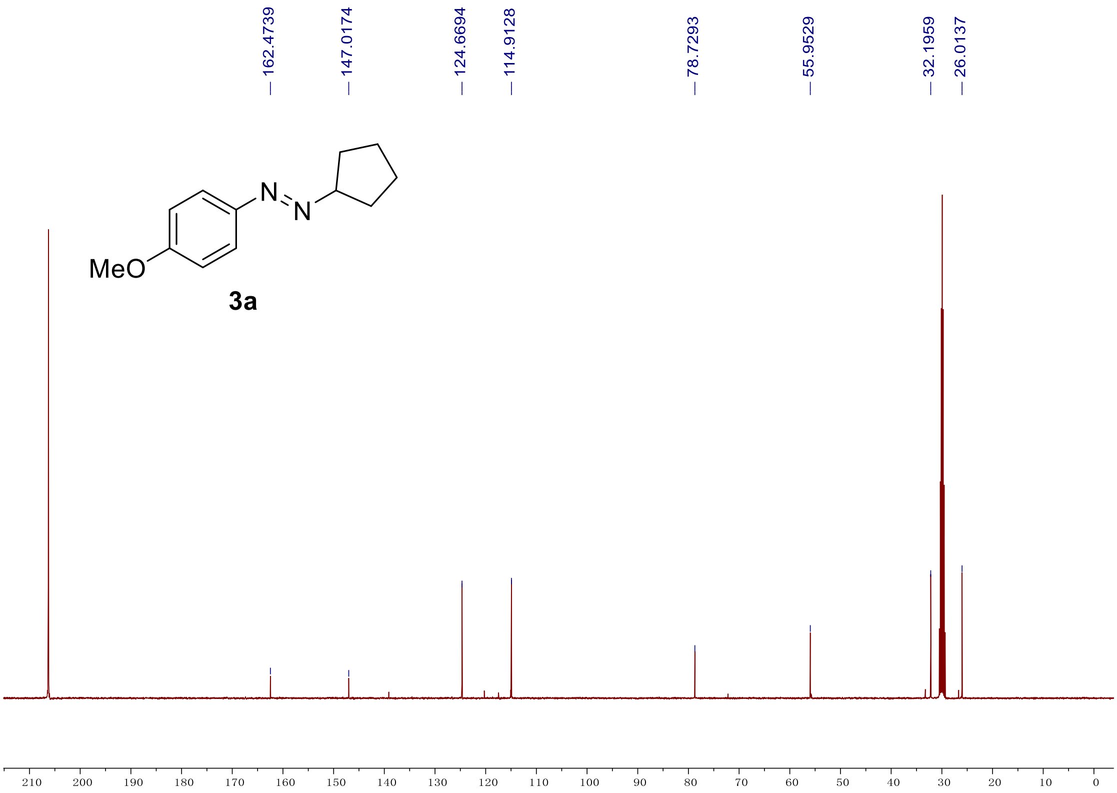

Supplementary Fig. 28. ^{13}C NMR of compound **3a.**

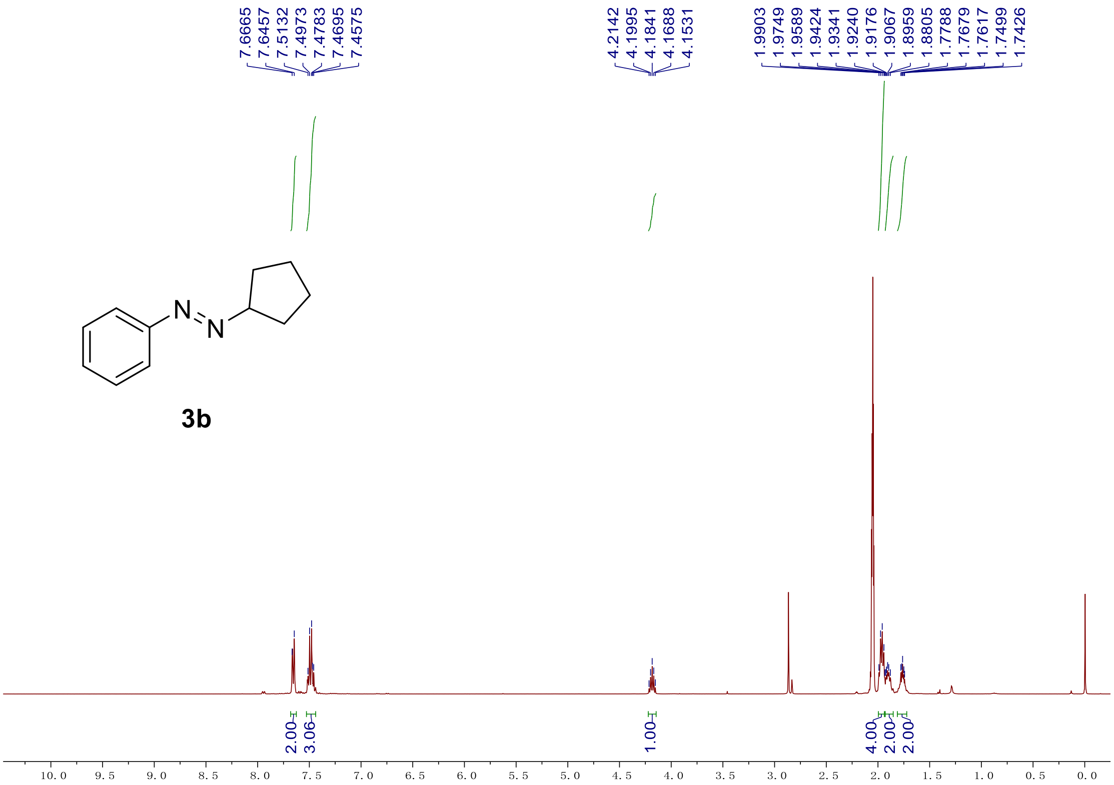

Supplementary Fig. 29. ^{1}H NMR of compound **3b.**

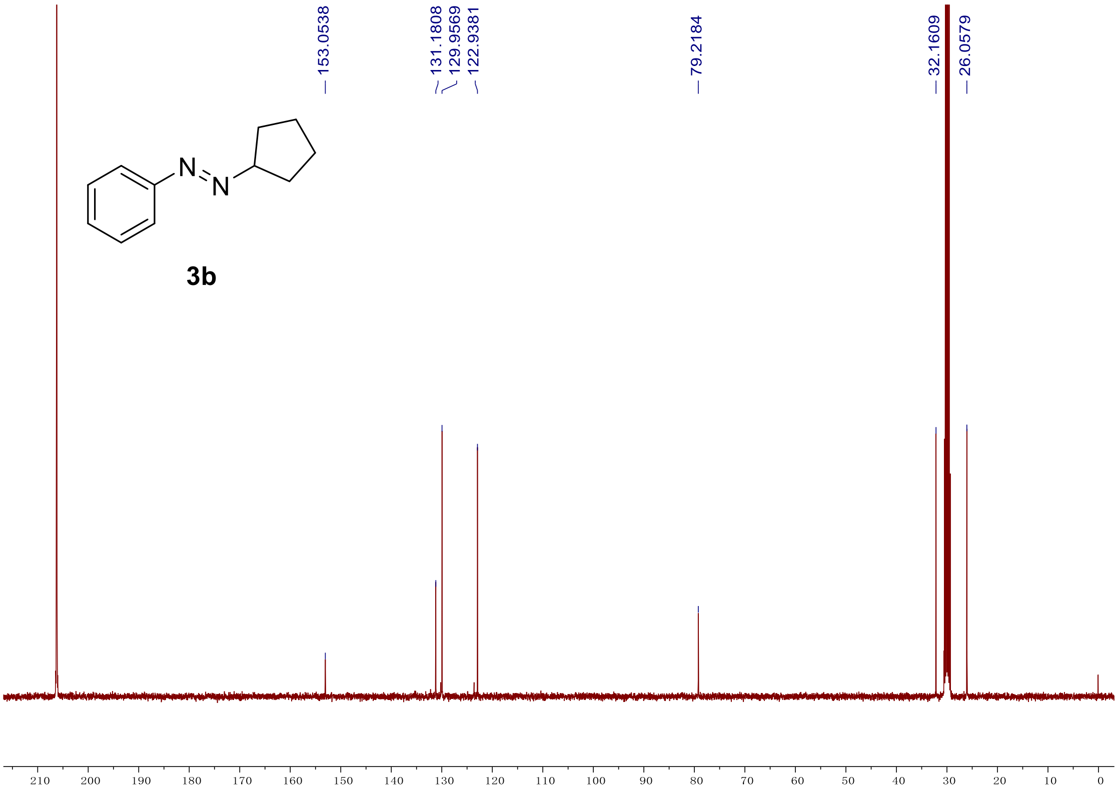

Supplementary Fig. 30. ^{13}C NMR of compound **3b.**

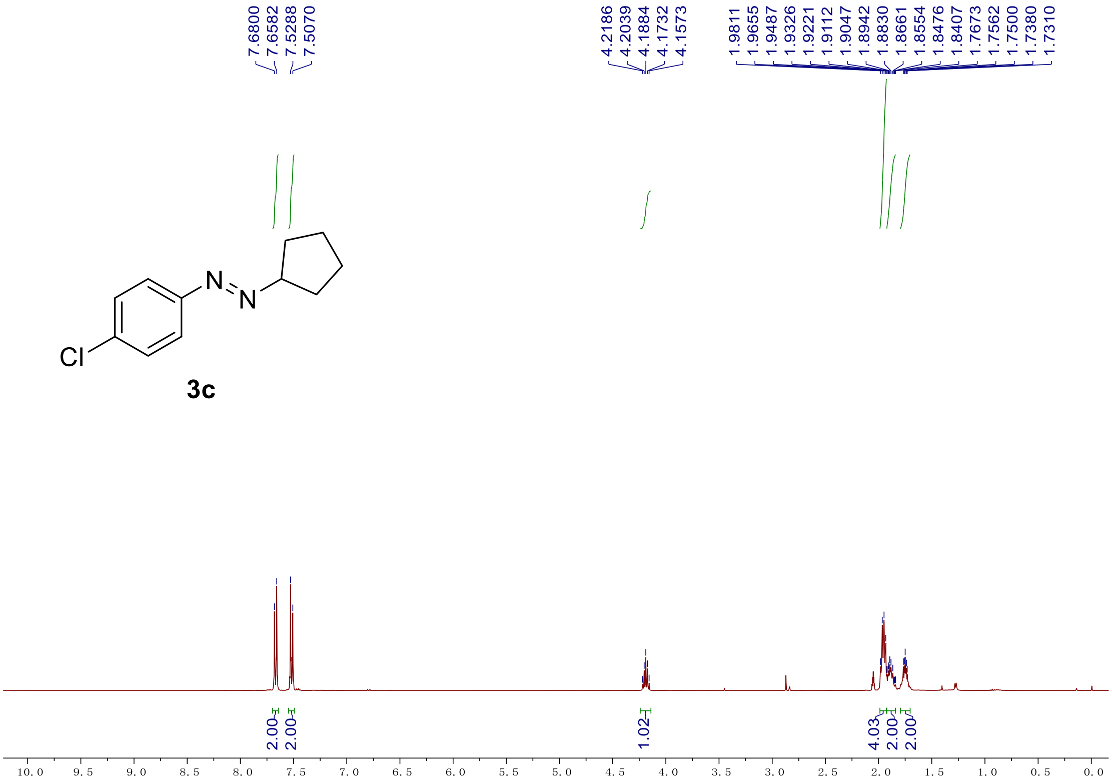

Supplementary Fig. 31. ^{1}H NMR of compound **3c.**

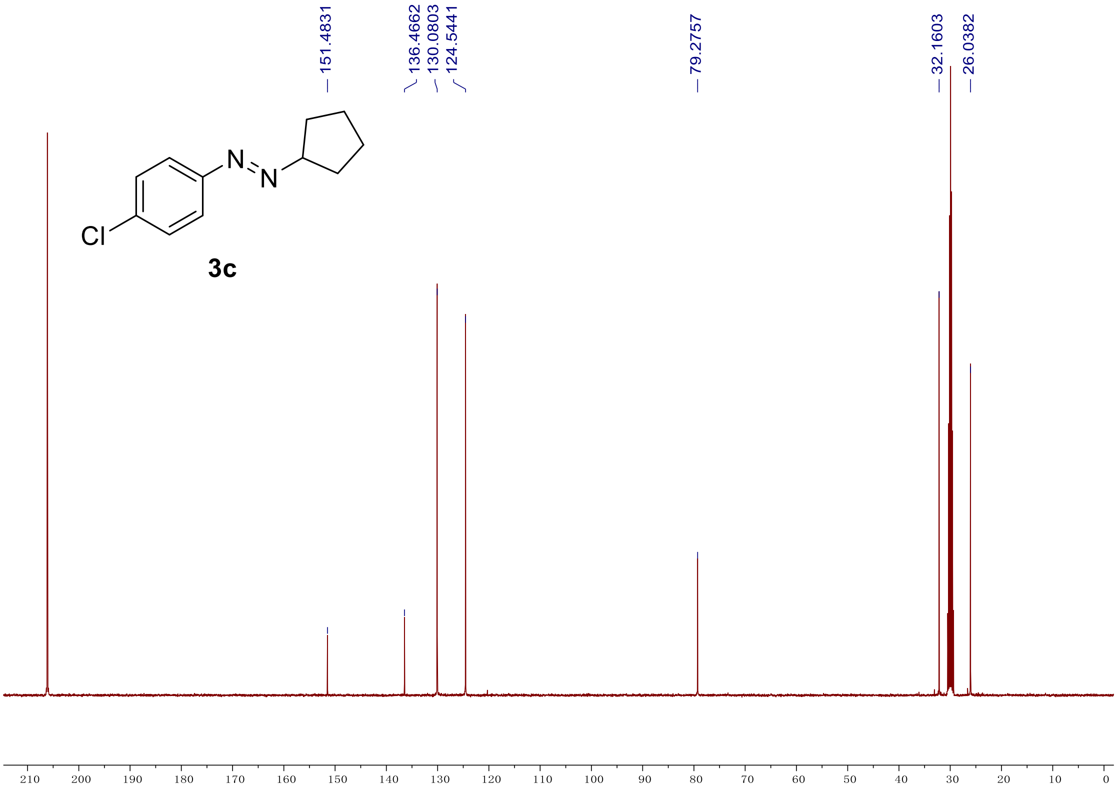

Supplementary Fig. 32. ^{13}C NMR of compound **3c.**

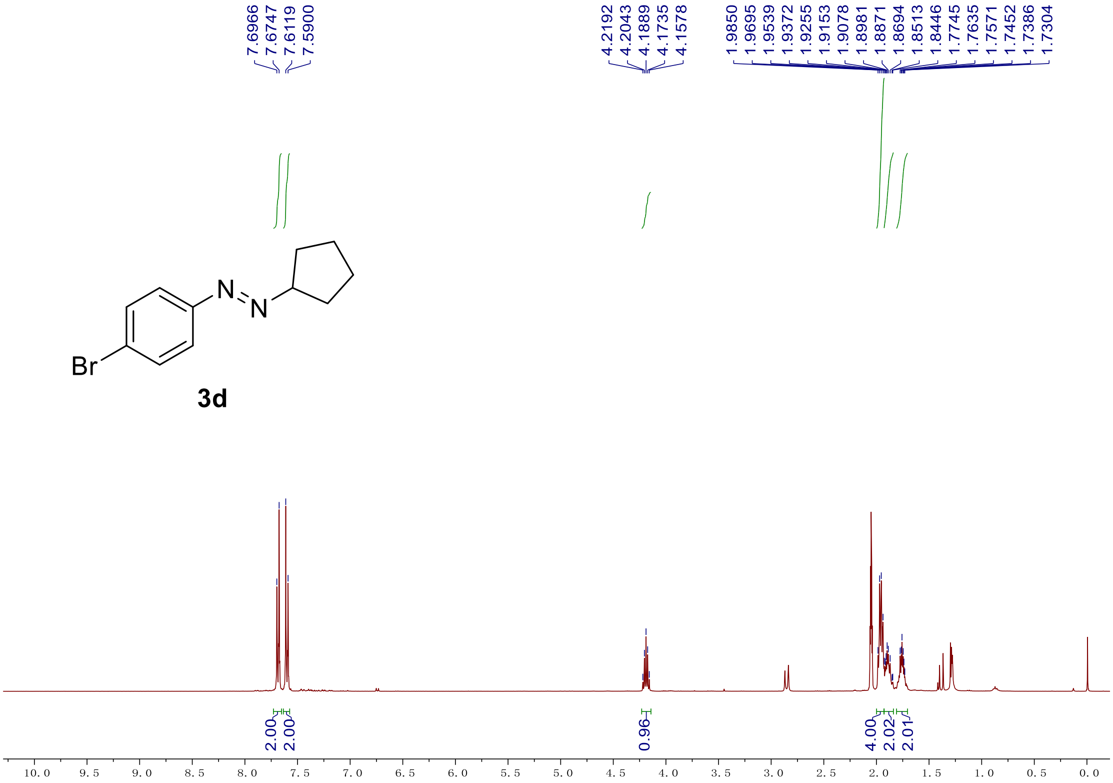

Supplementary Fig. 33. ^{1}H NMR of compound **3d.**

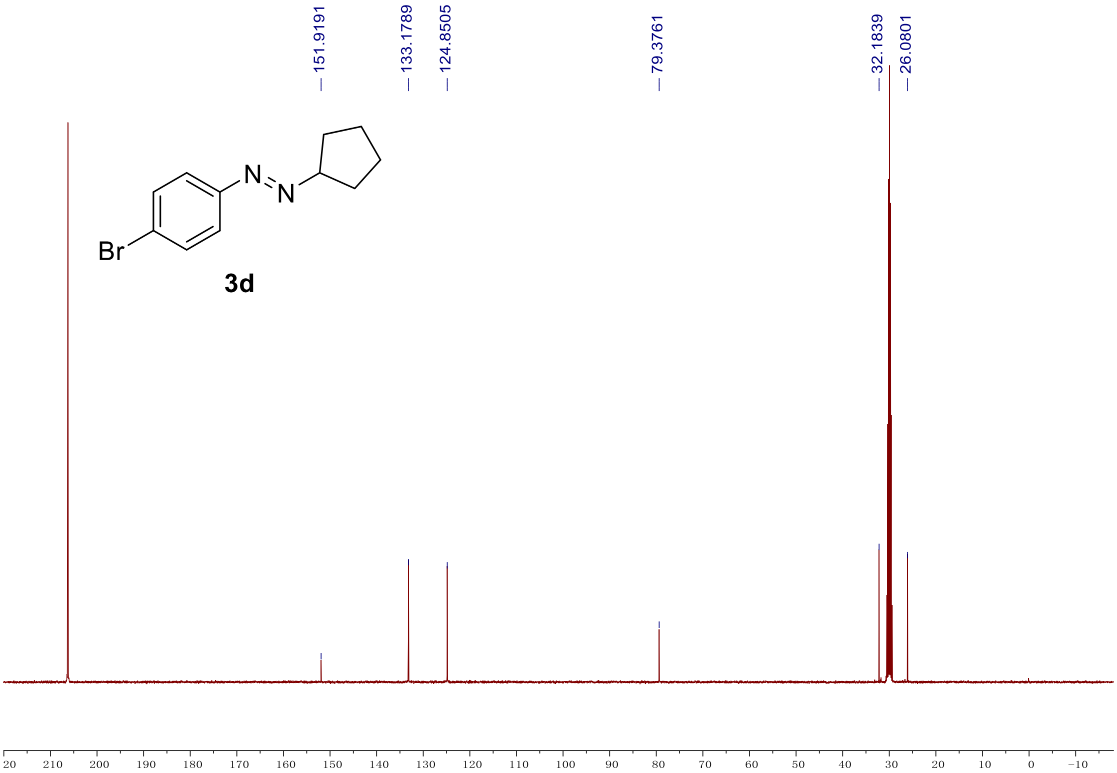

Supplementary Fig. 34. ^{13}C NMR of compound **3d.**

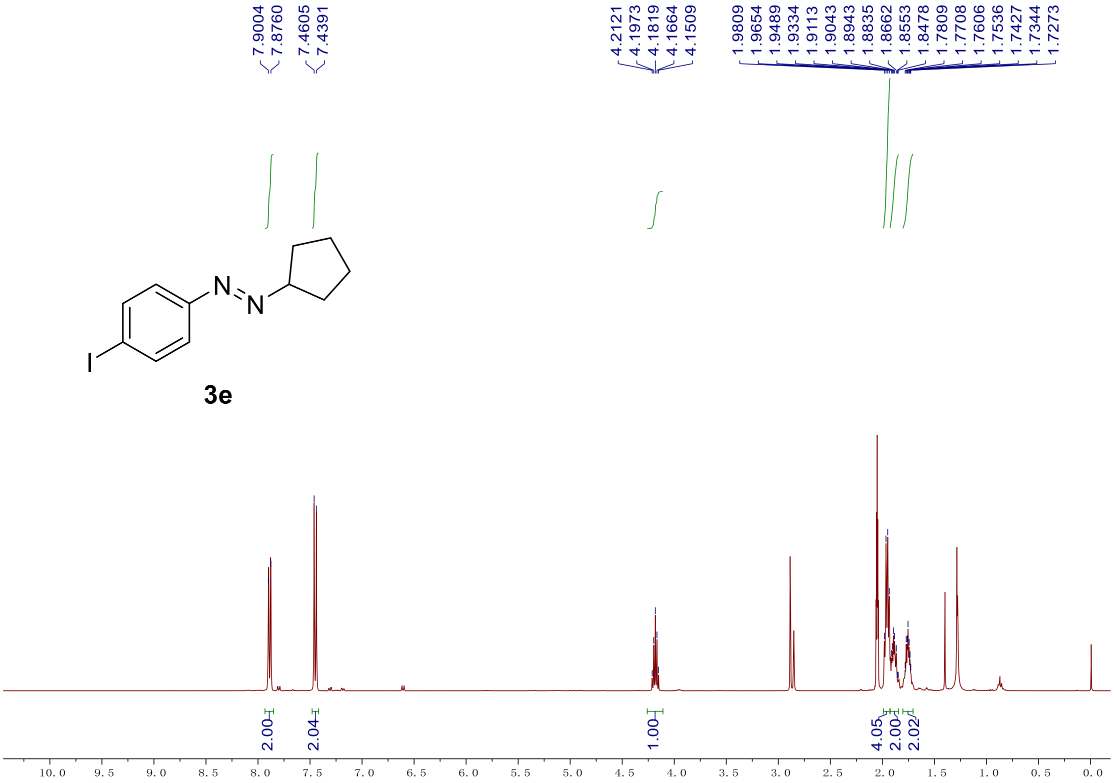

Supplementary Fig. 35. ^{1}H NMR of compound **3e.**

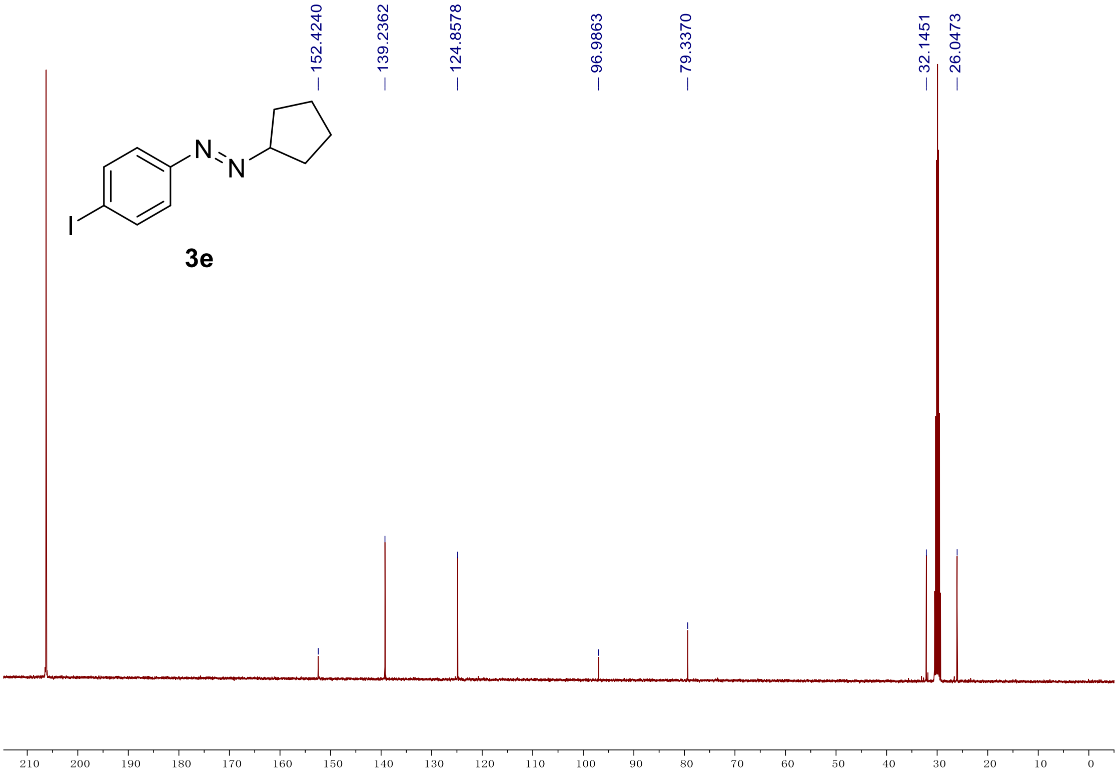

Supplementary Fig. 36. ^{13}C NMR of compound **3e.**

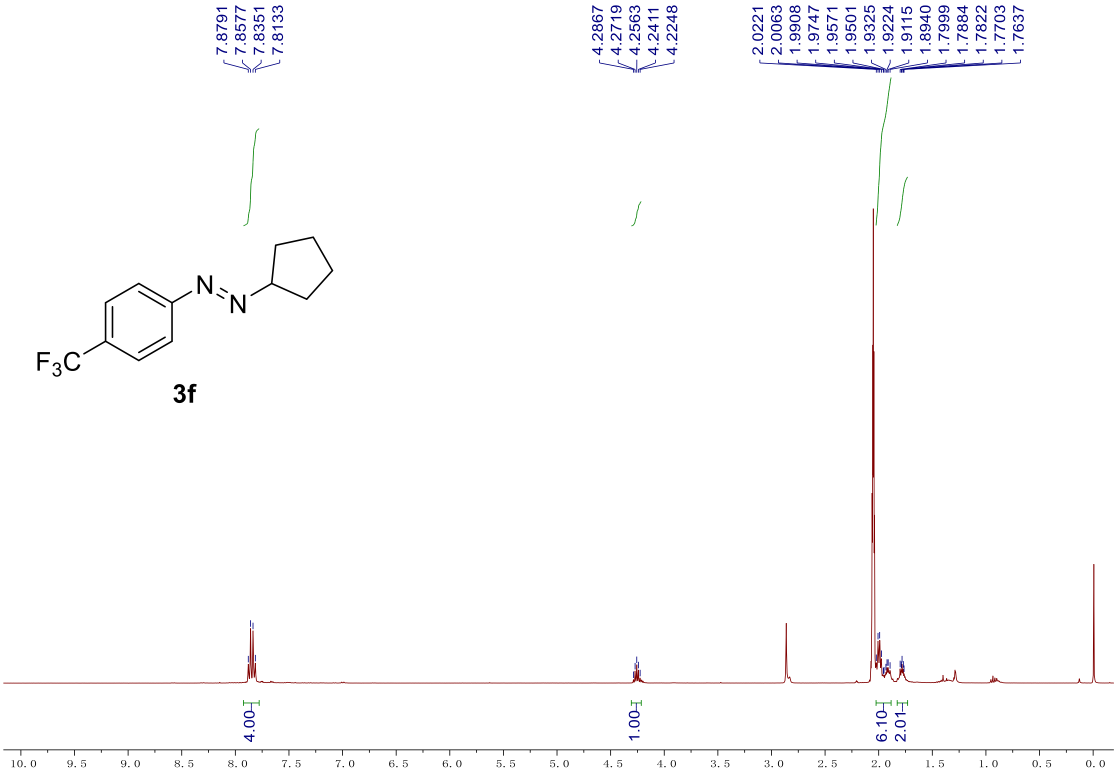

Supplementary Fig. 37. ^{1}H NMR of compound **3f.**

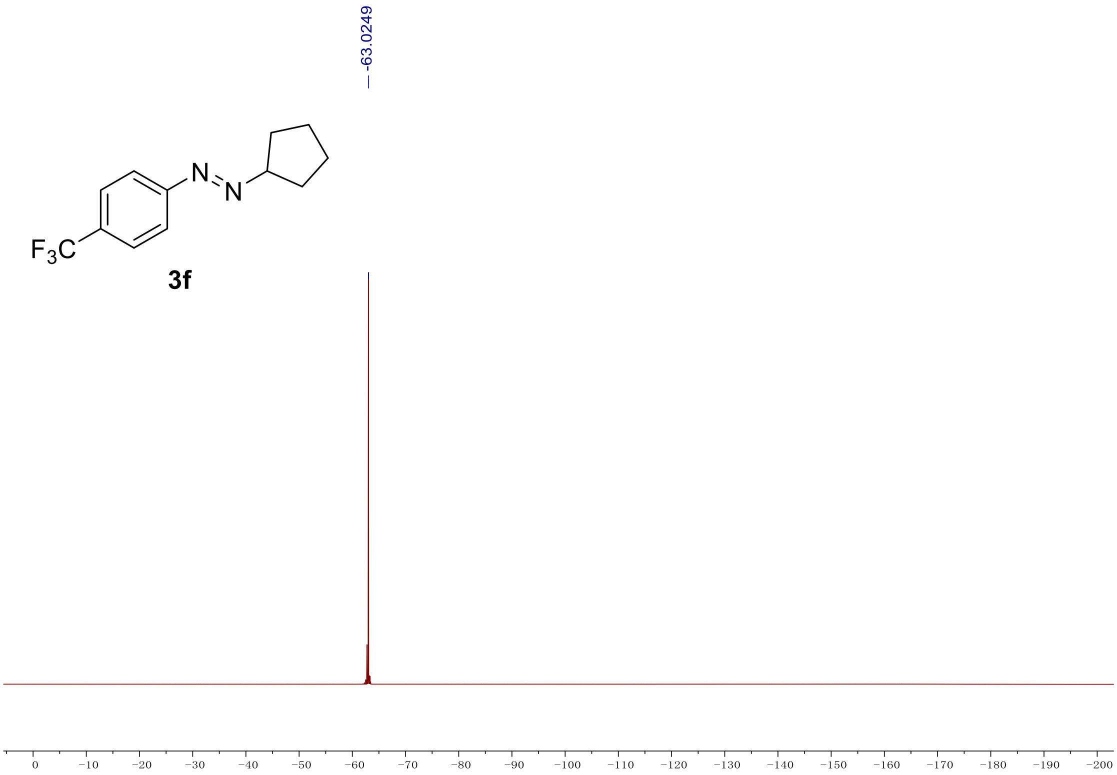

Supplementary Fig. 38. ^{19}F NMR of compound **3f.**

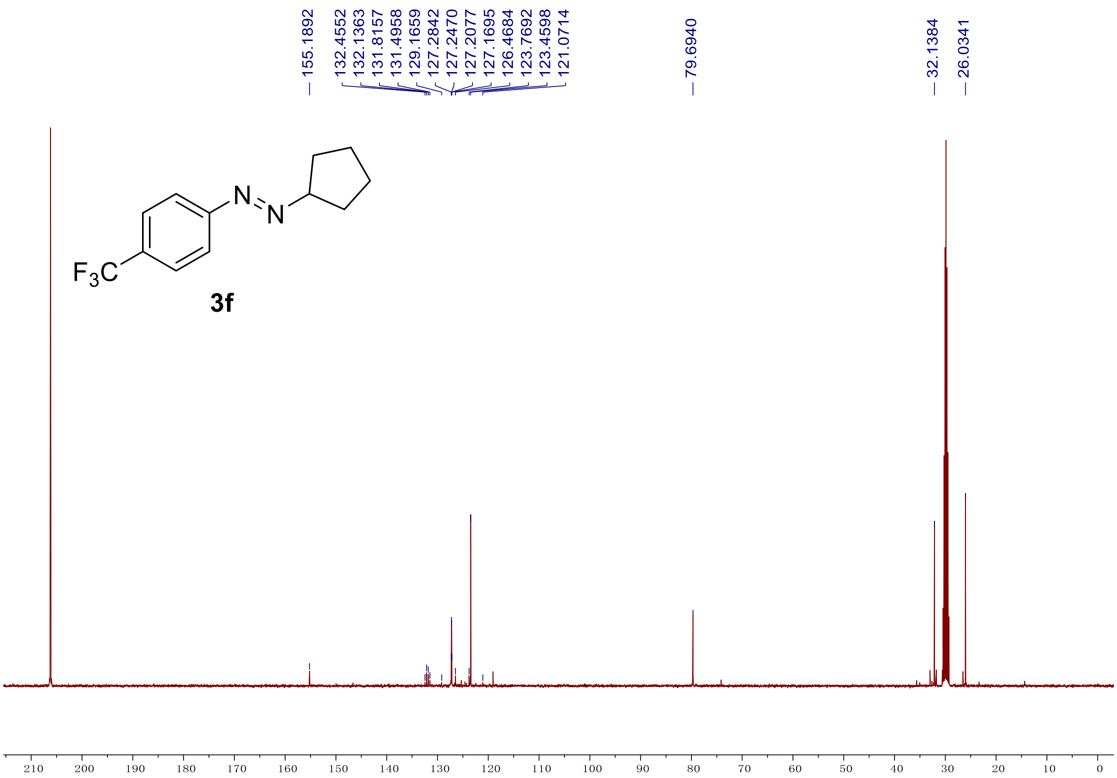

Supplementary Fig. 39. ^{13}C NMR of compound **3f.**

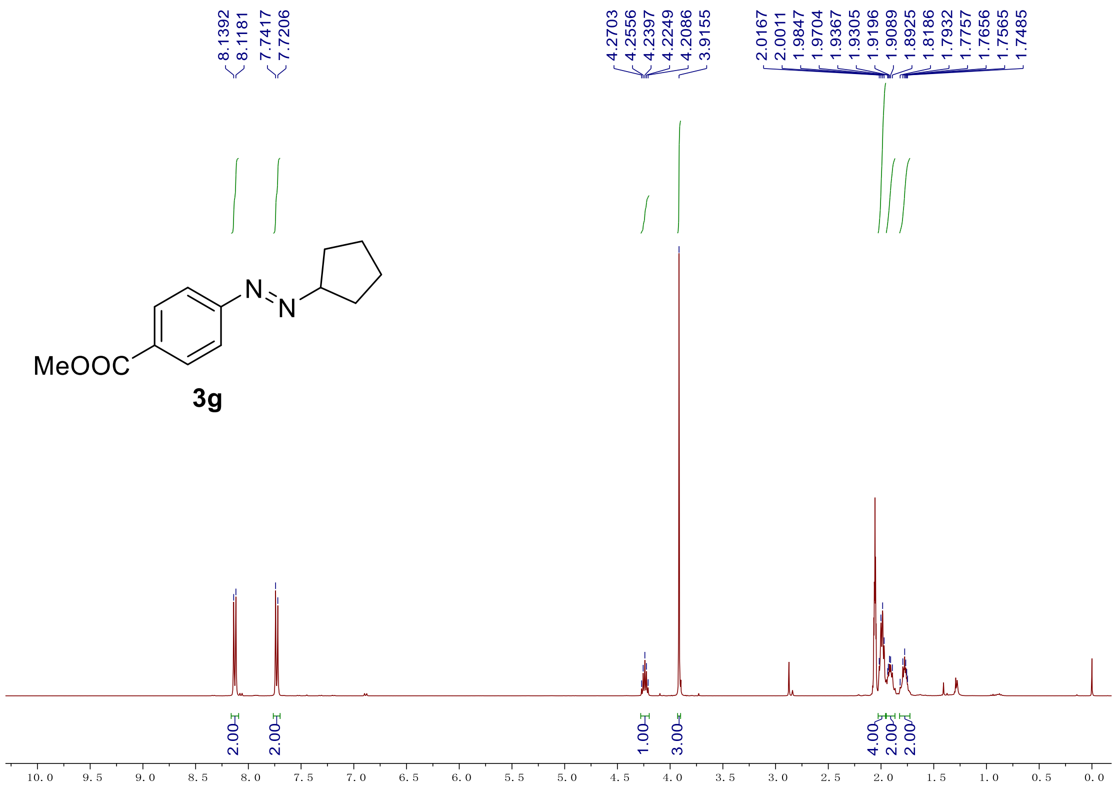

Supplementary Fig. 40. ^{1}H NMR of compound **3g.**

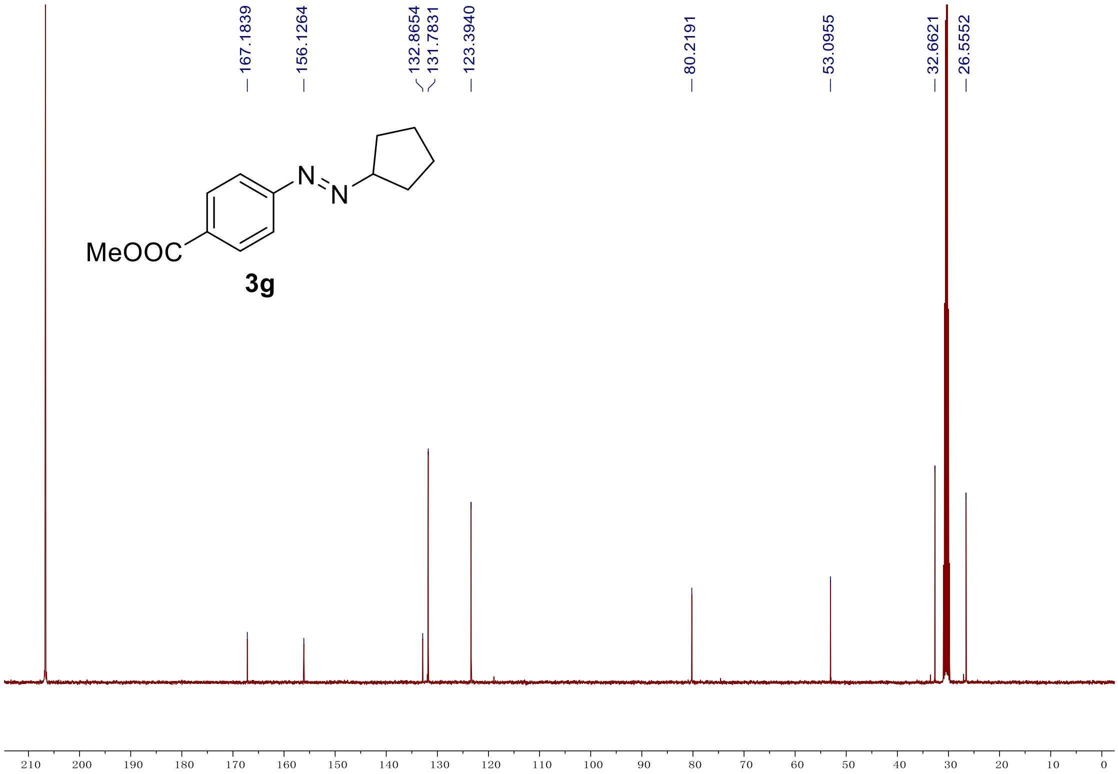

Supplementary Fig. 41. ^{13}C NMR of compound **3g.**

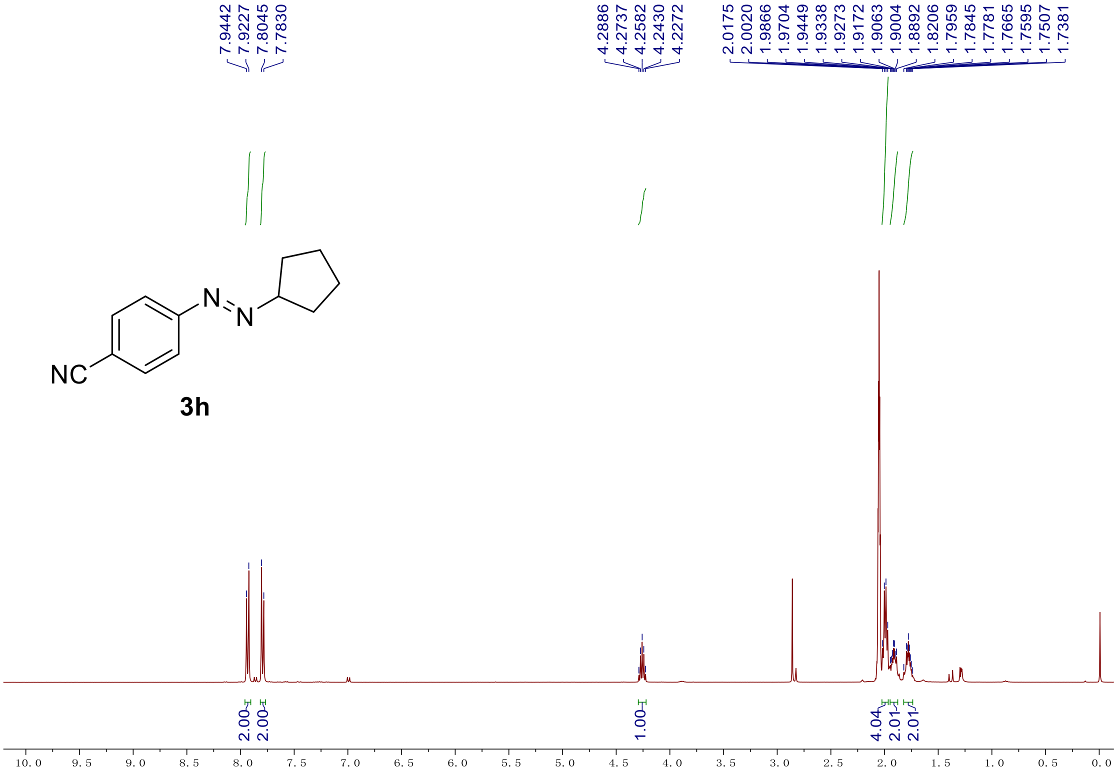

Supplementary Fig. 42. ^{1}H NMR of compound **3h.**

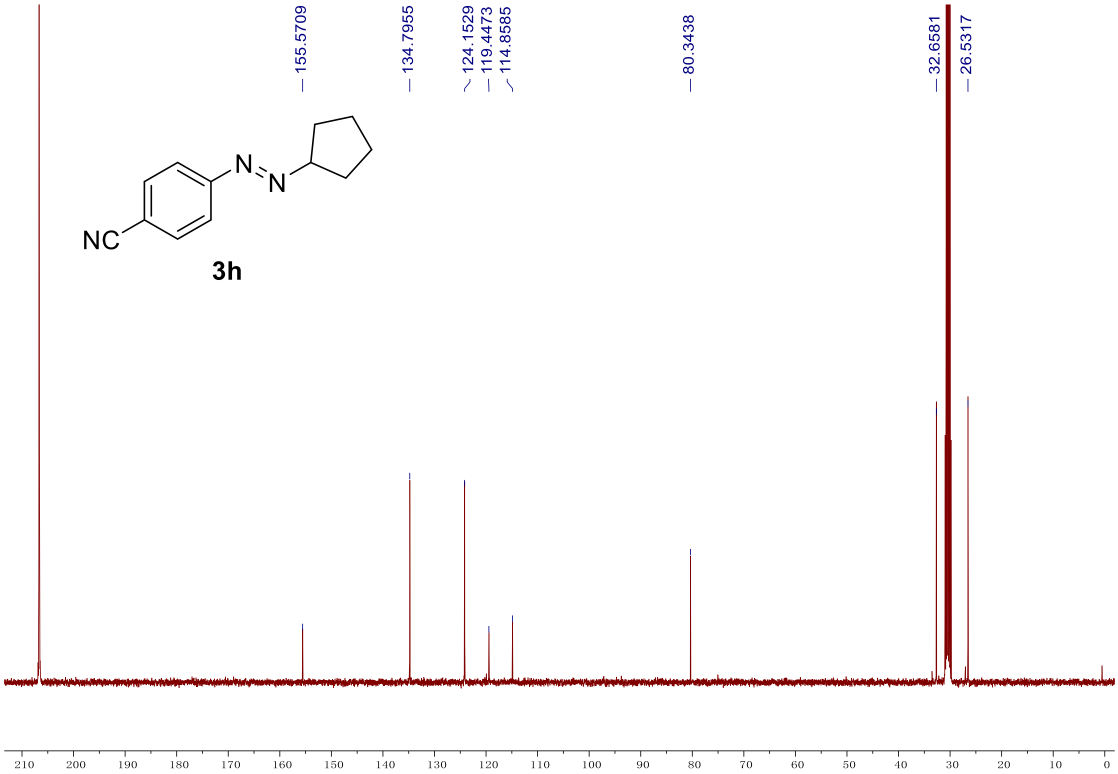

Supplementary Fig. 43. ^{13}C NMR of compound **3h.**

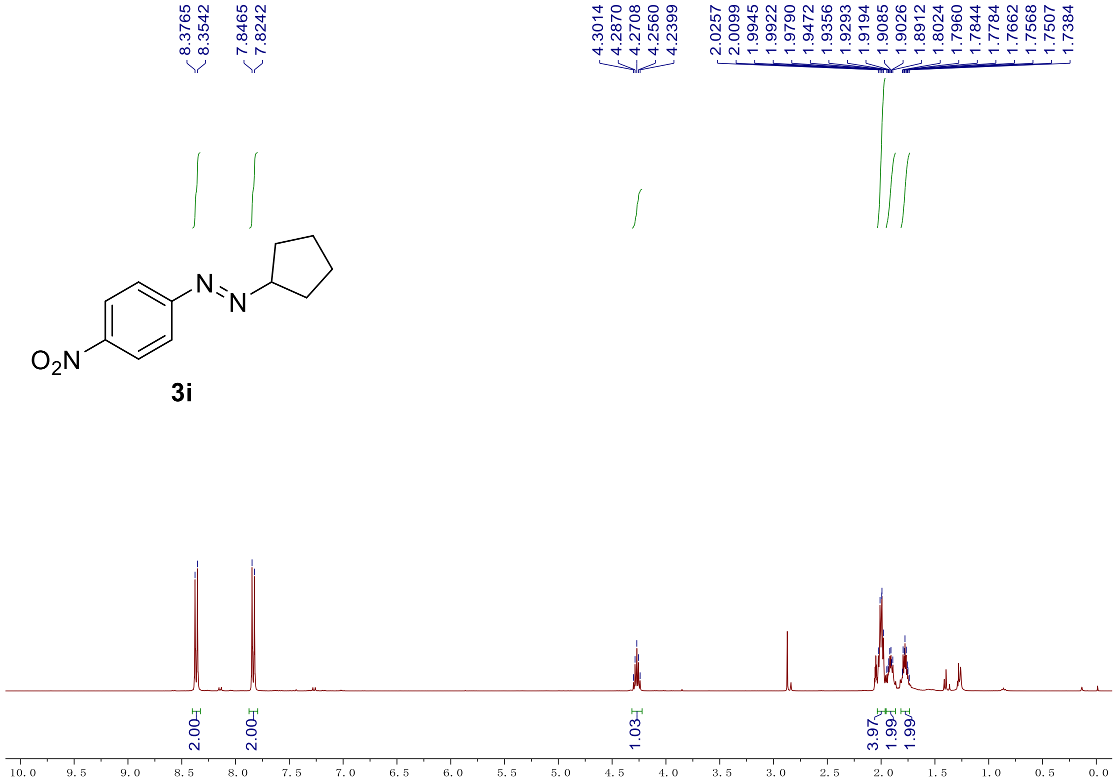

Supplementary Fig. 44. ^{1}H NMR of compound **3i.**

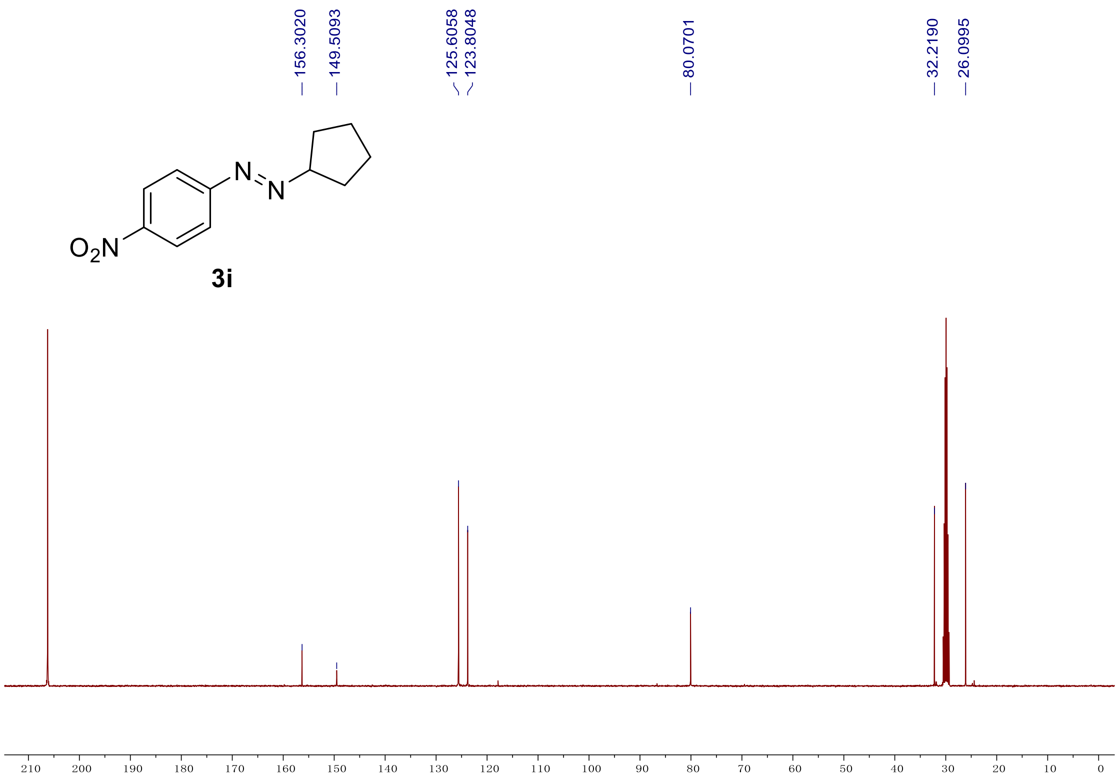

Supplementary Fig. 45. ^{13}C NMR of compound **3i.**

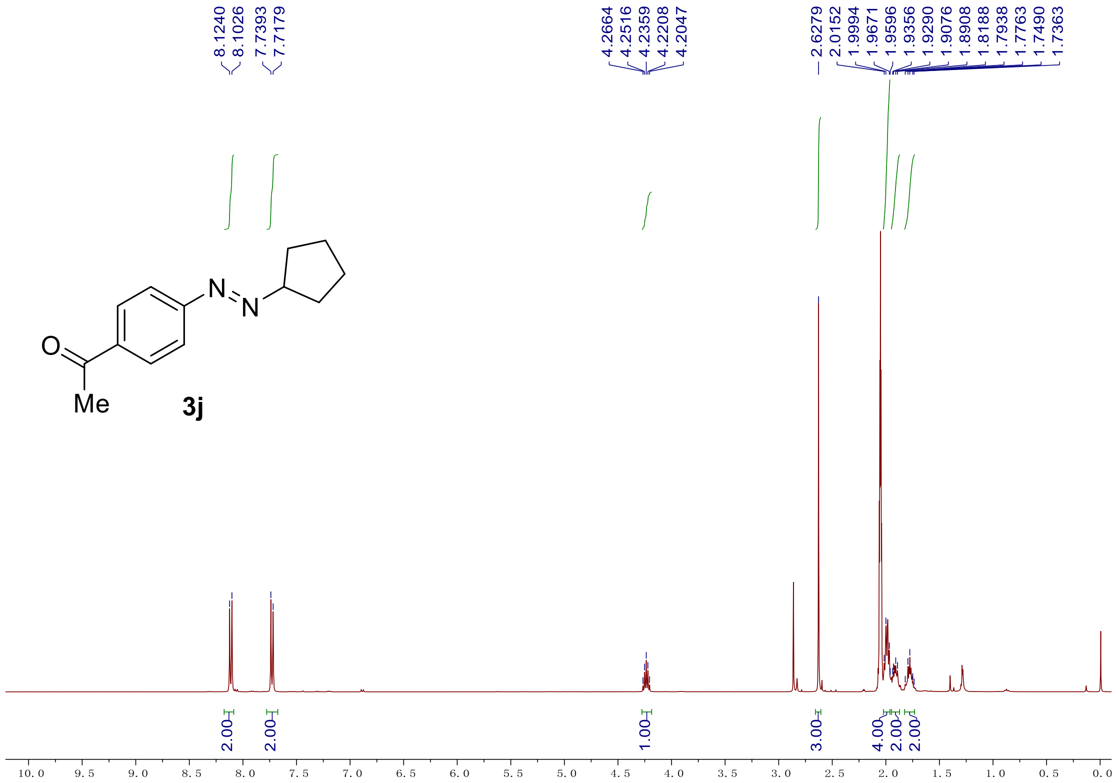

Supplementary Fig. 46. ^{1}H NMR of compound **3j.**

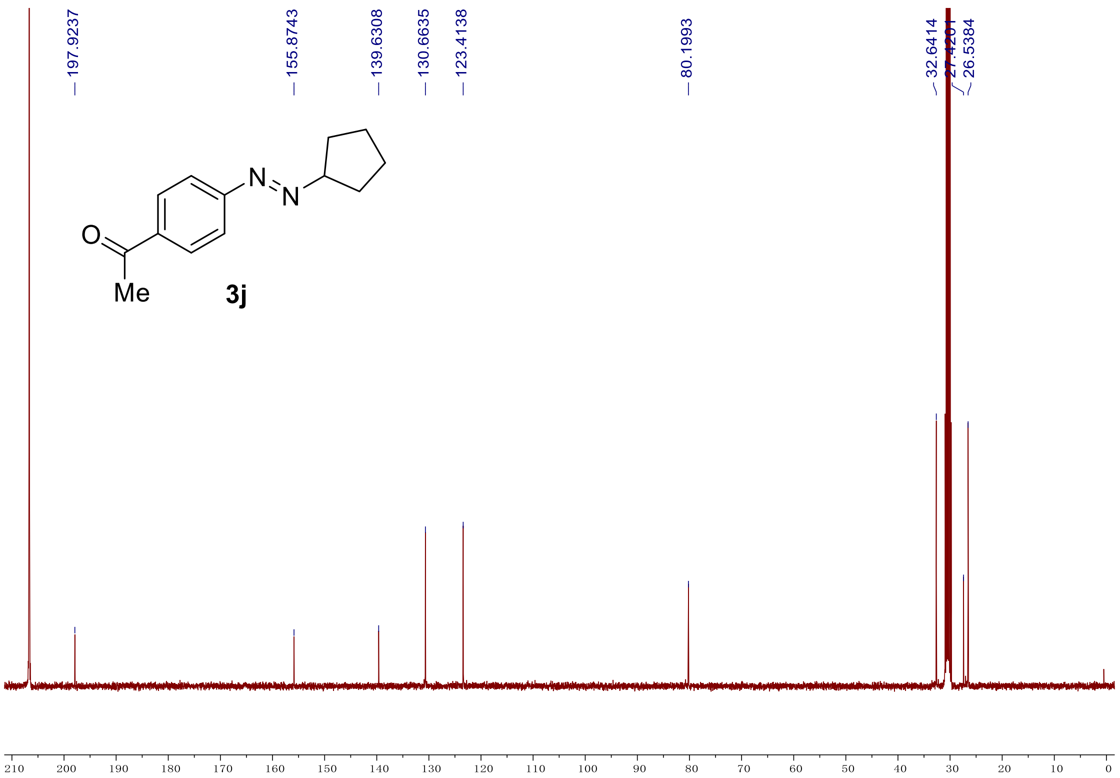

Supplementary Fig. 47. ^{13}C NMR of compound **3j.**

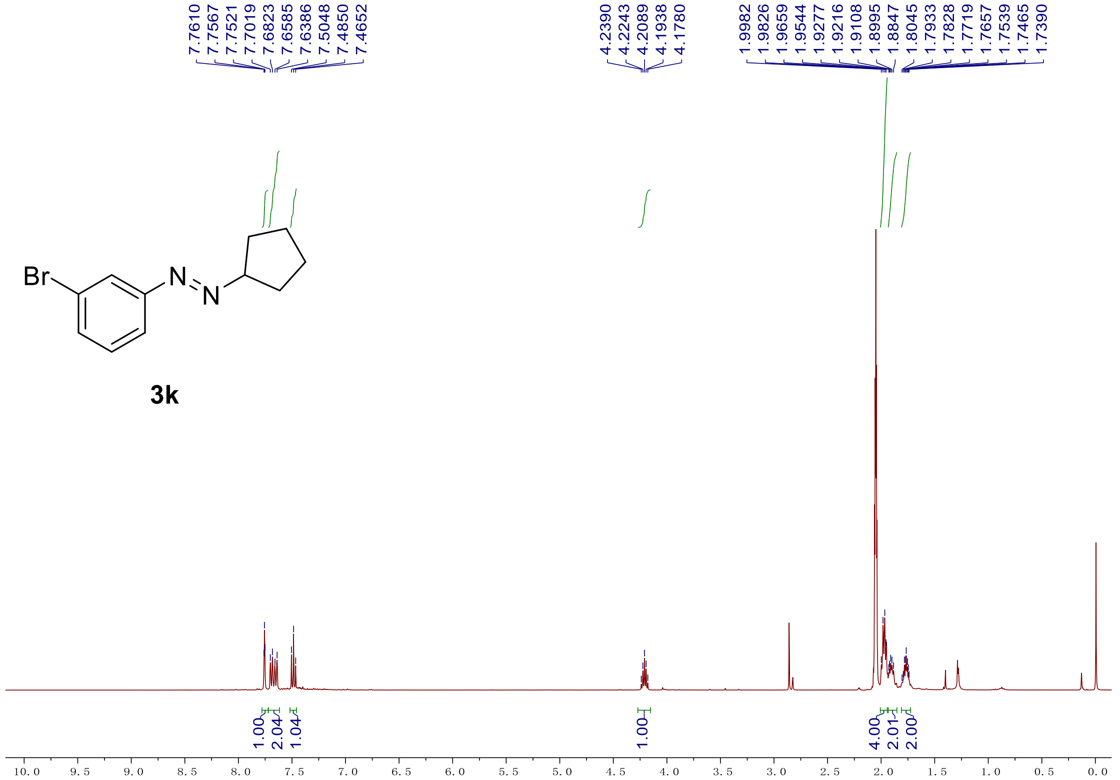

Supplementary Fig. 48. ^{1}H NMR of compound **3k.**

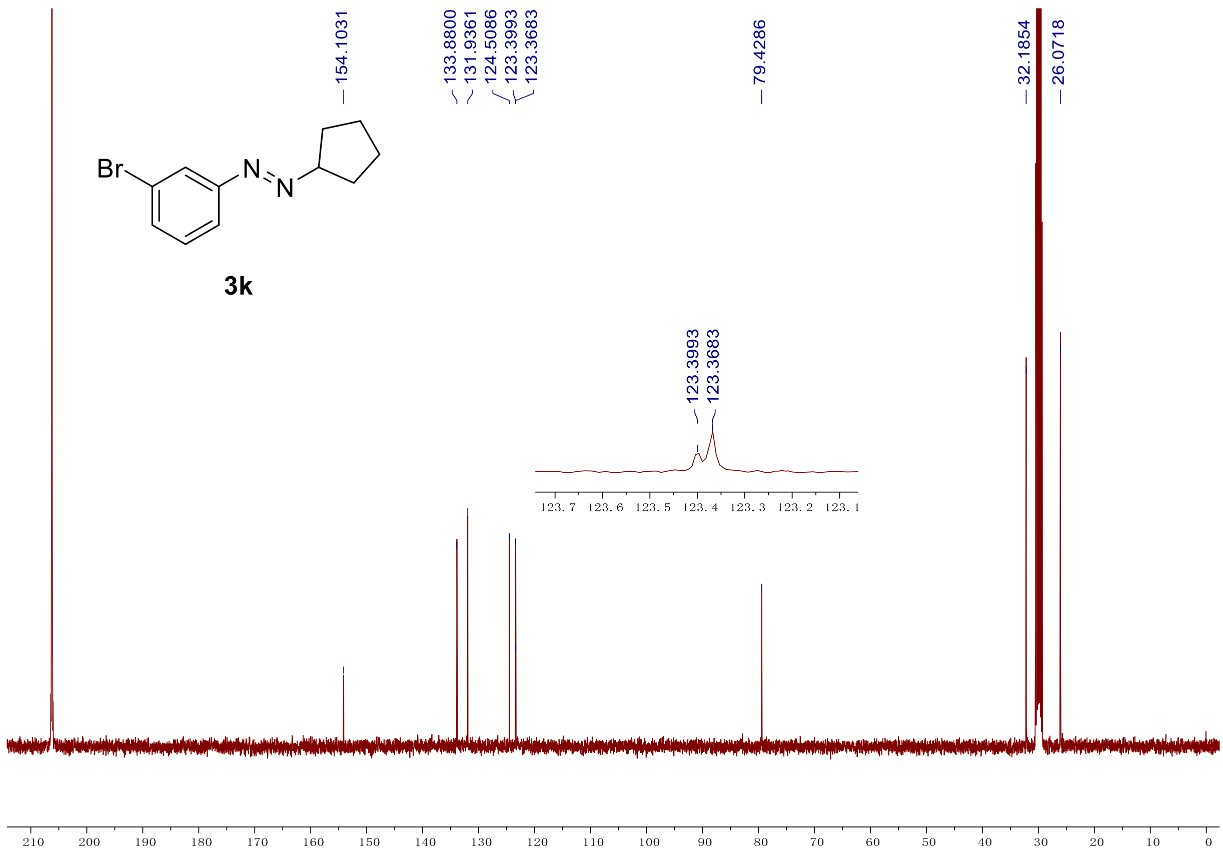

Supplementary Fig. 49. ^{13}C NMR of compound **3k.**

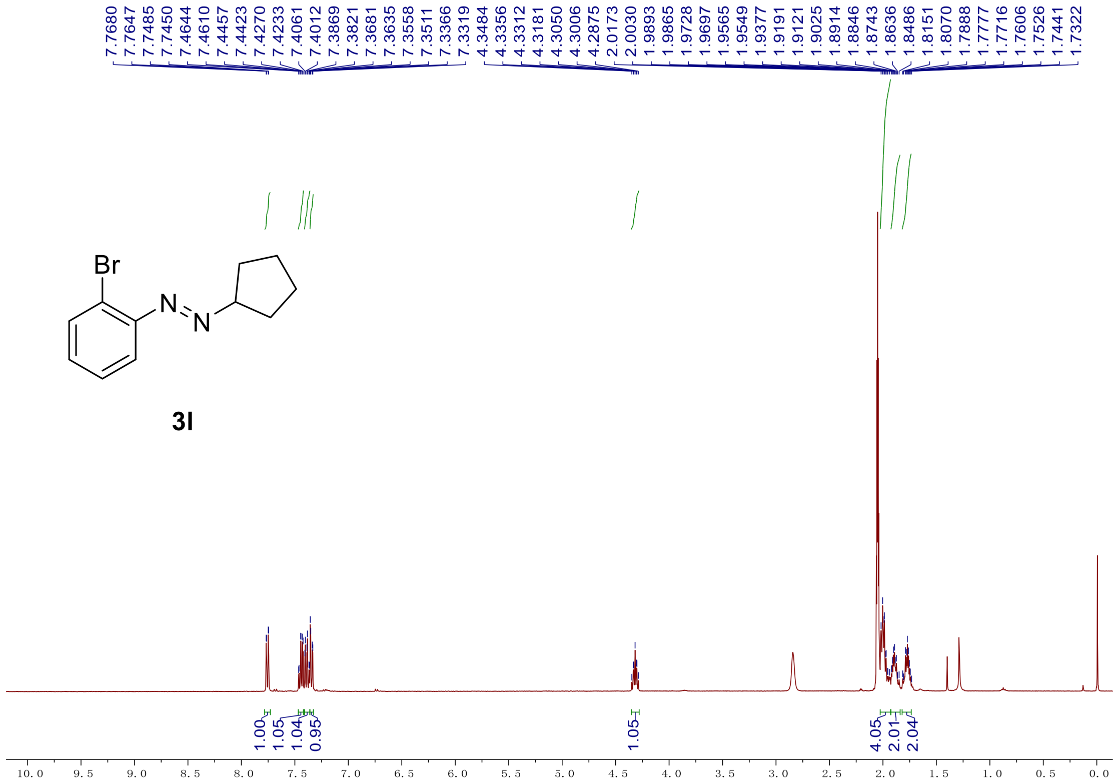

Supplementary Fig. 50. ^{1}H NMR of compound **3l.**

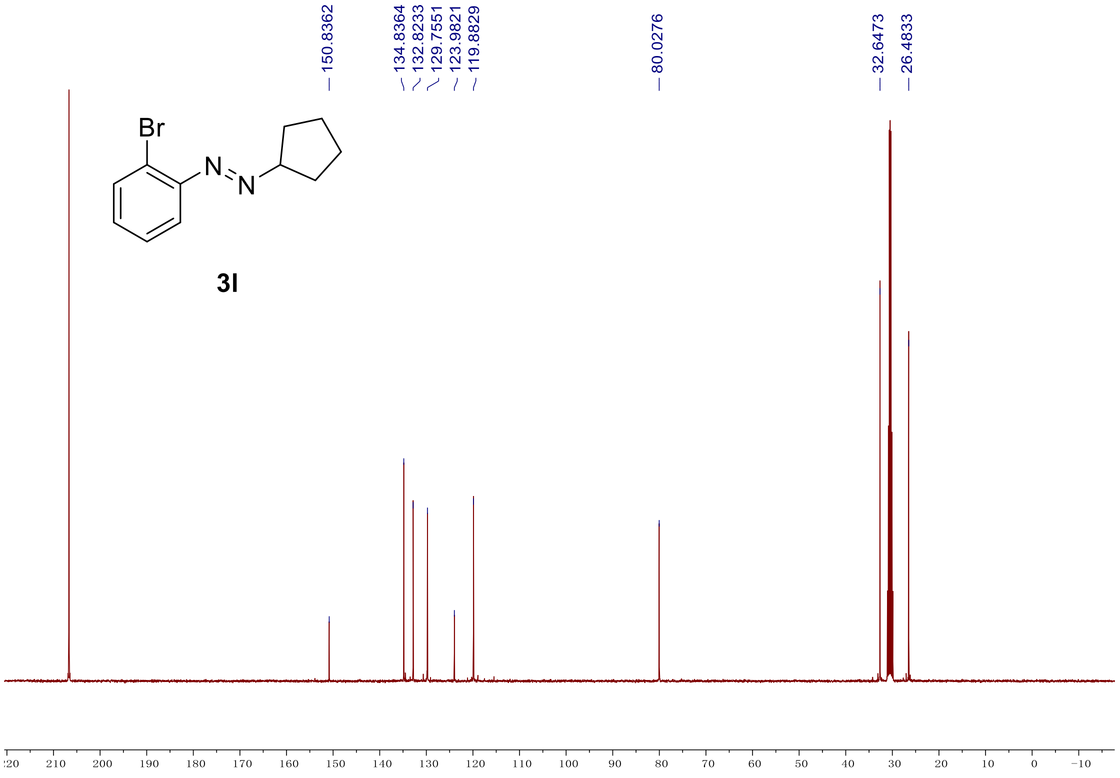

Supplementary Fig. 51. ^{13}C NMR of compound **3l.**

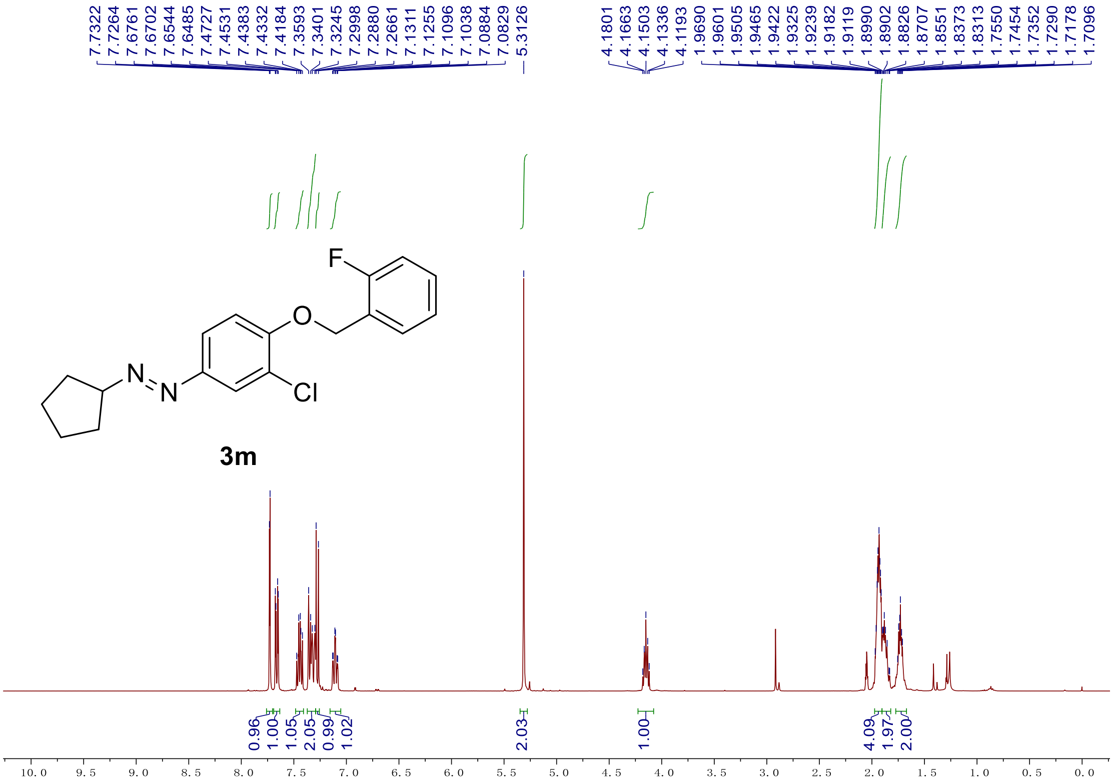

Supplementary Fig. 52. ^{1}H NMR of compound **3m.**

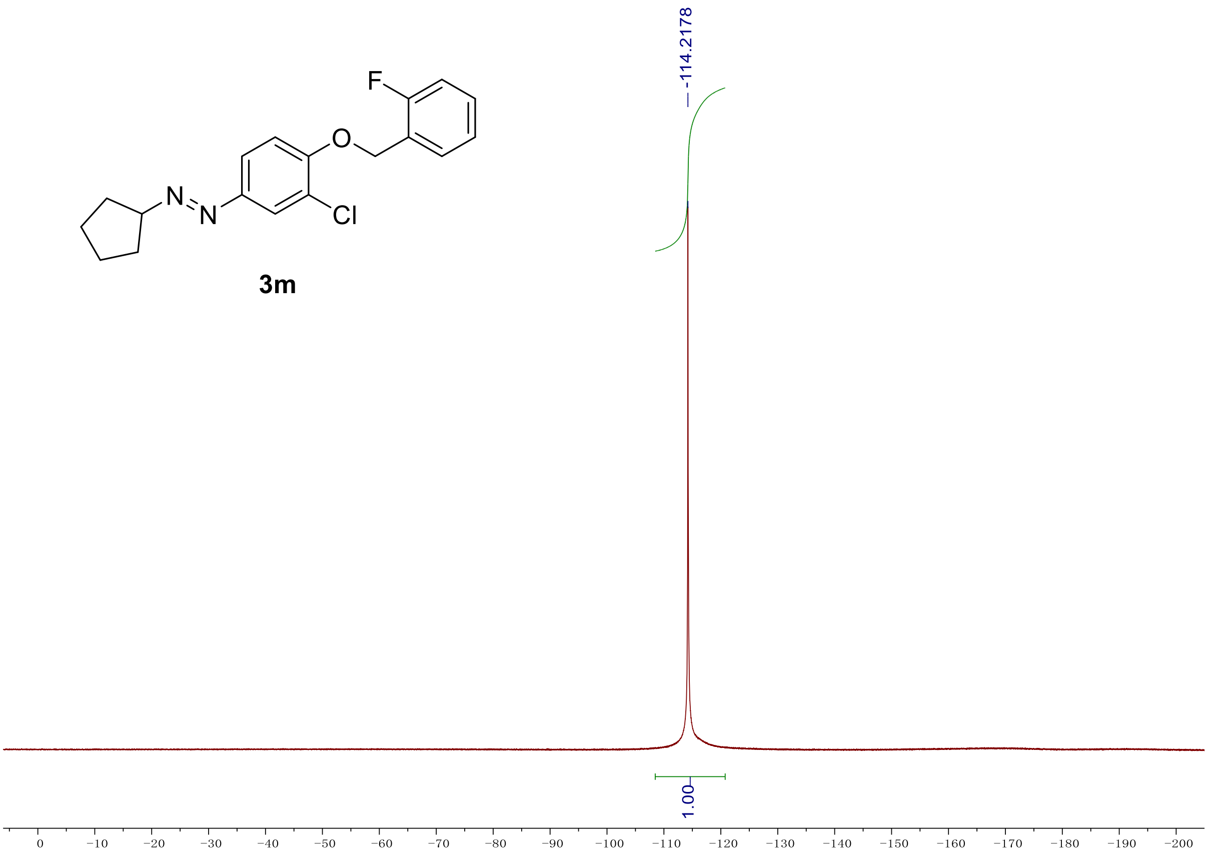

Supplementary Fig. 53. ^{19}F NMR of compound **3m.**

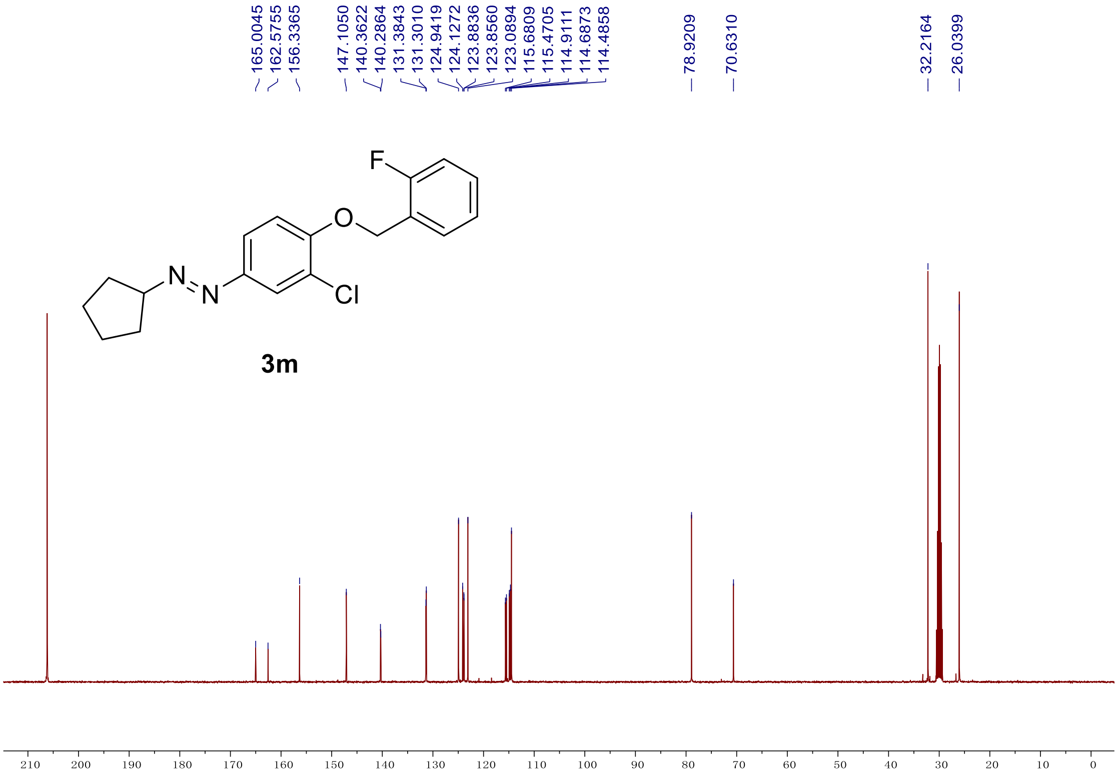

Supplementary Fig. 54. ^{13}C NMR of compound **3m.**

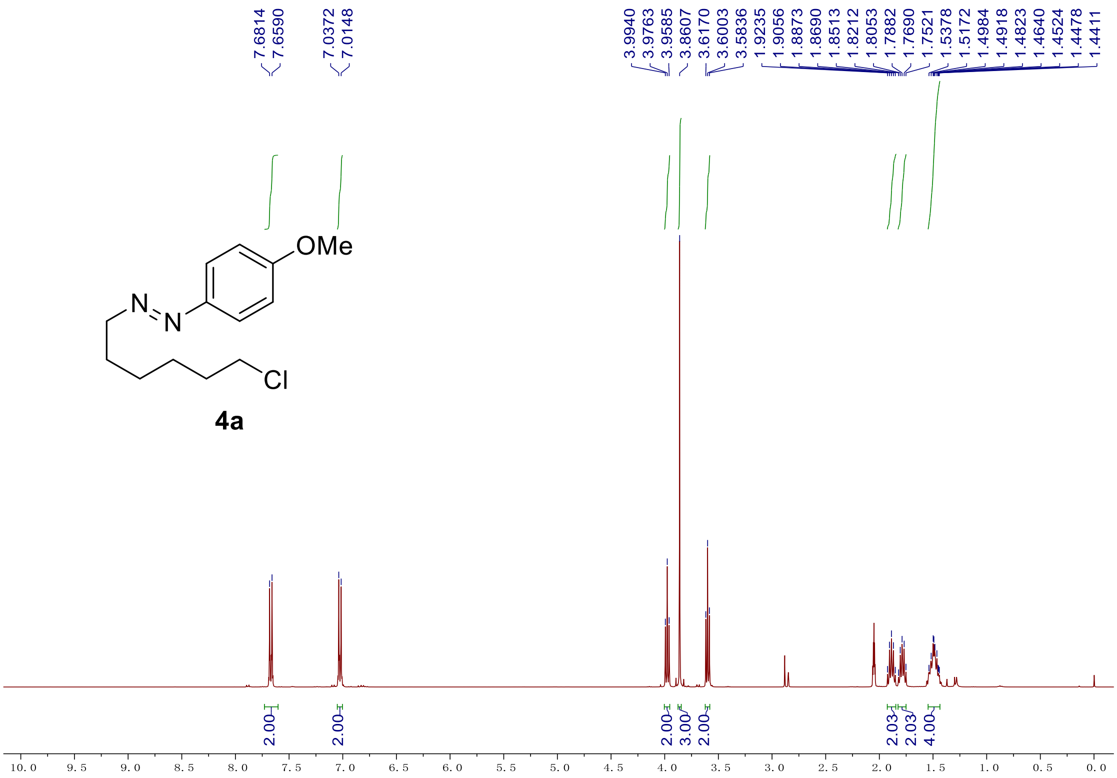

Supplementary Fig. 55. ^{1}H NMR of compound **4a.**

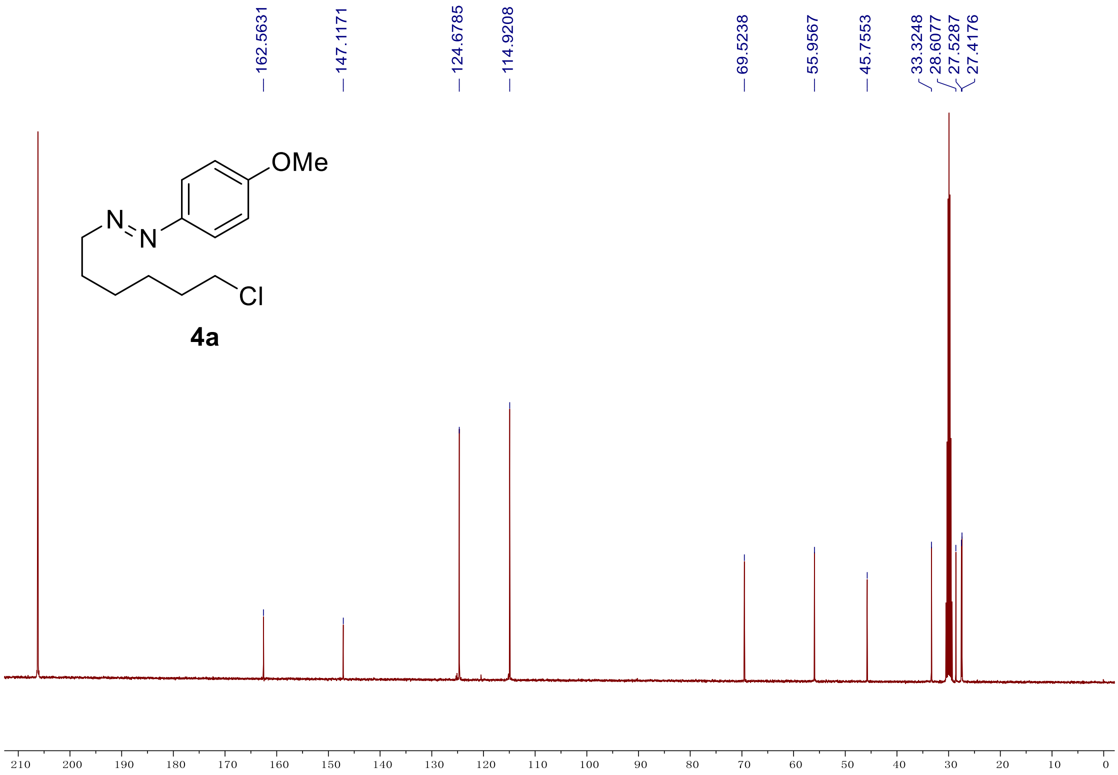

Supplementary Fig. 56. ^{13}C NMR of compound **4a.**

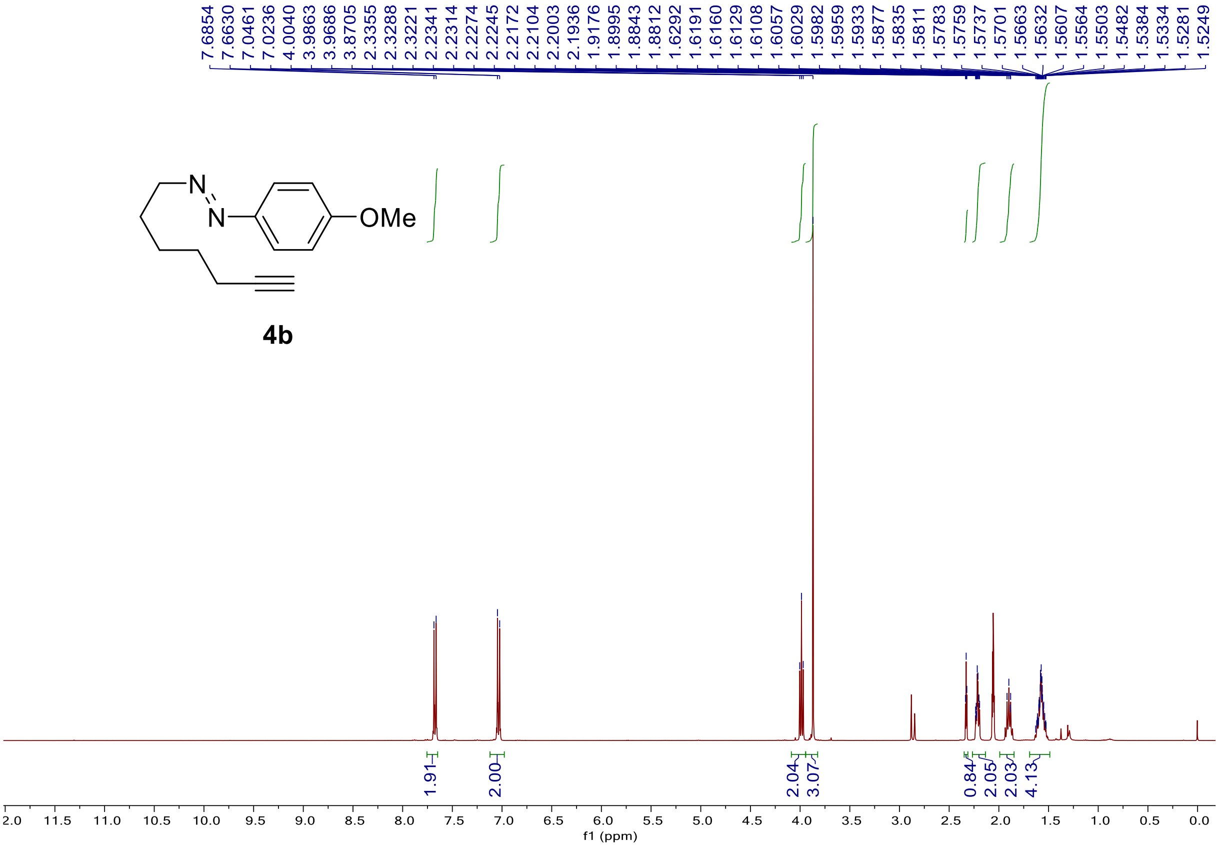

Supplementary Fig. 57. ^{1}H NMR of compound **4b.**

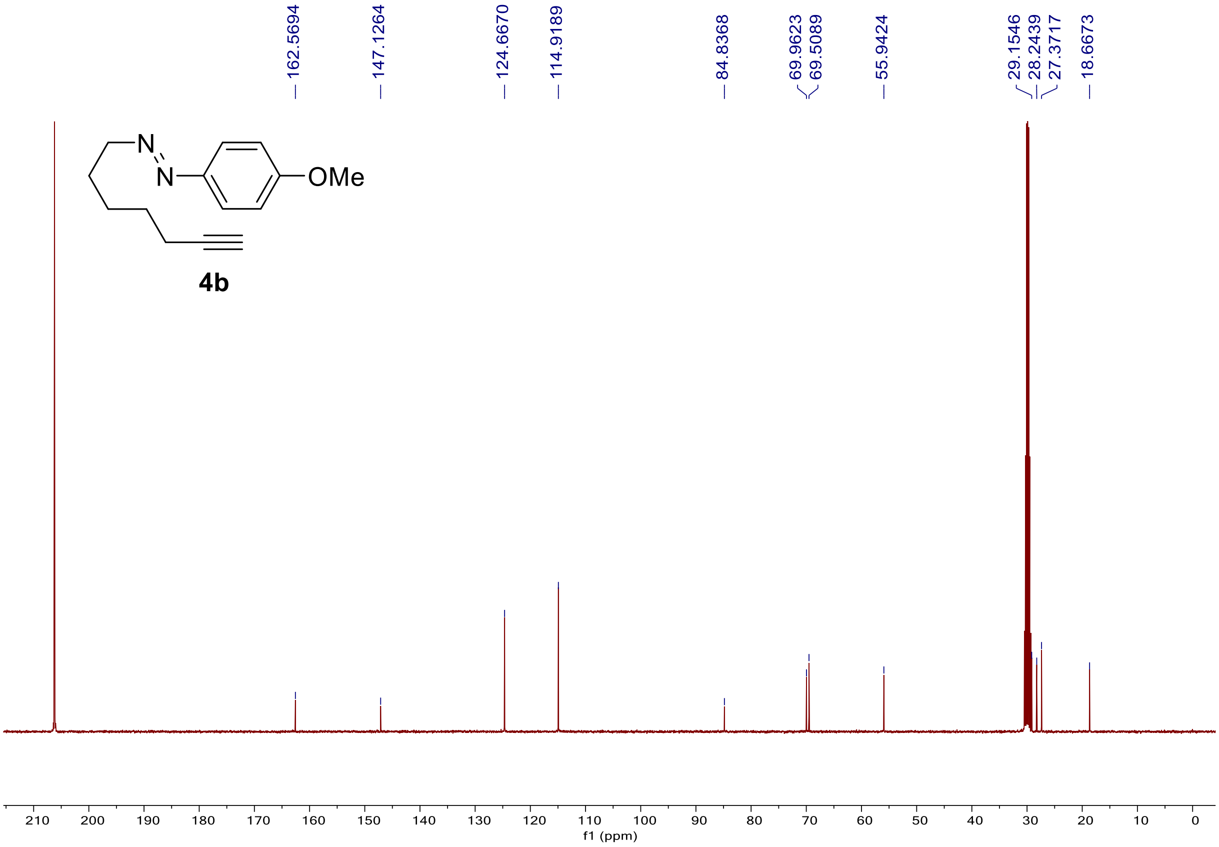

Supplementary Fig. 58. ^{13}C NMR of compound **4b.**

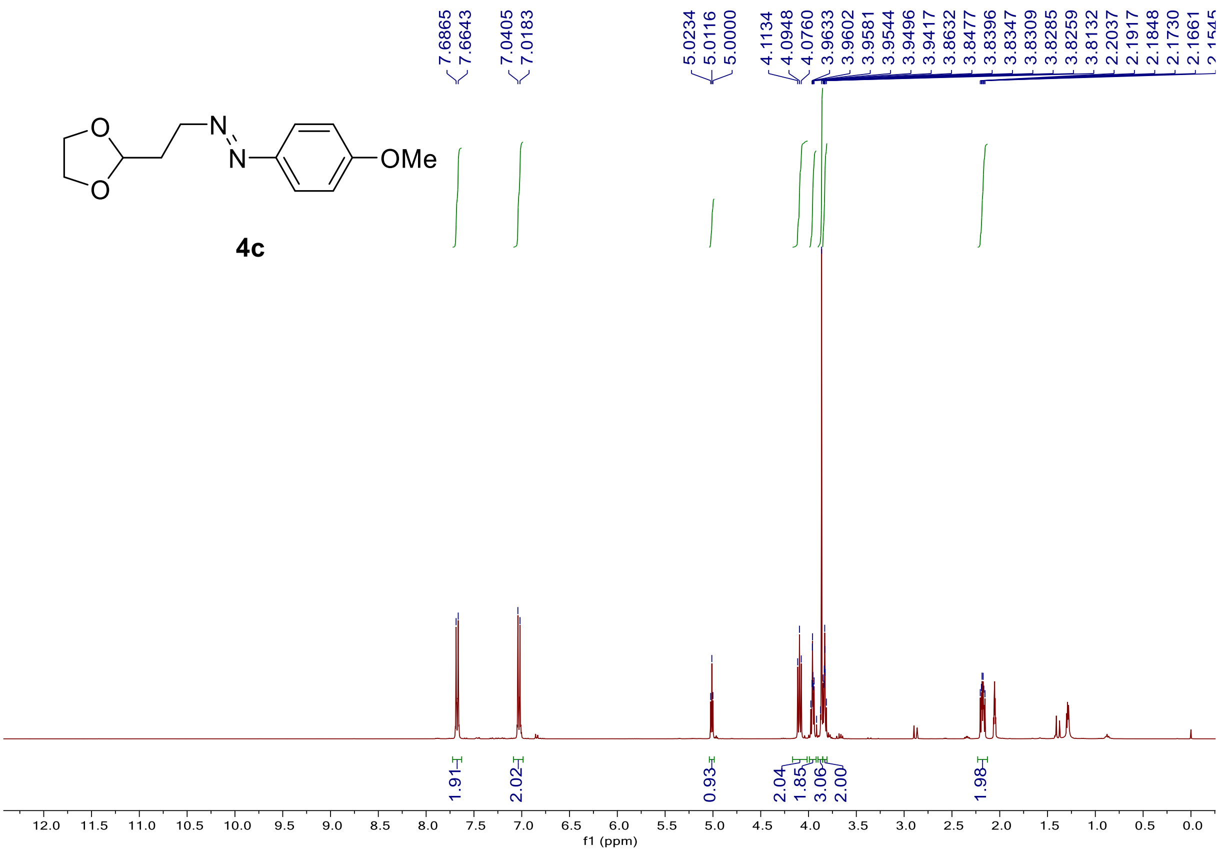

Supplementary Fig. 59. ^{1}H NMR of compound **4c.**

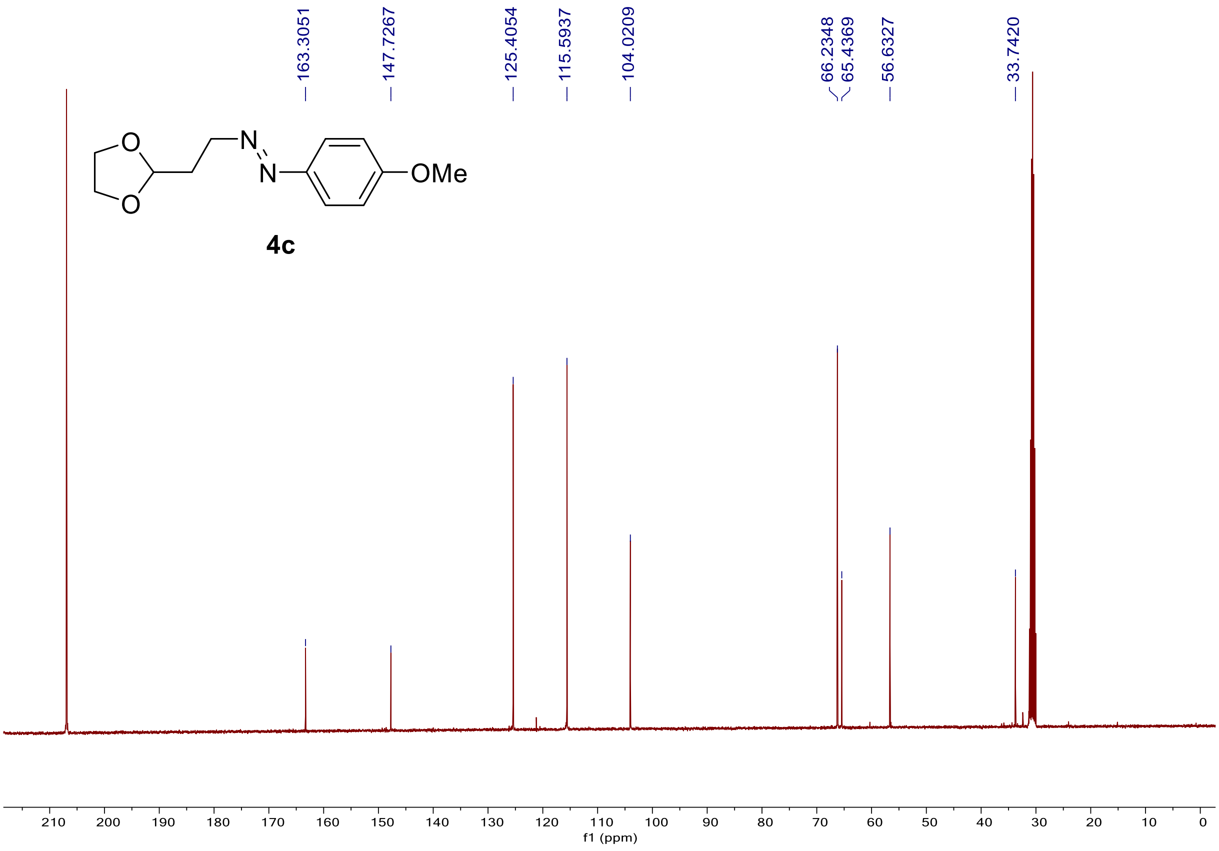

Supplementary Fig. 60. ^{13}C NMR of compound **4c.**

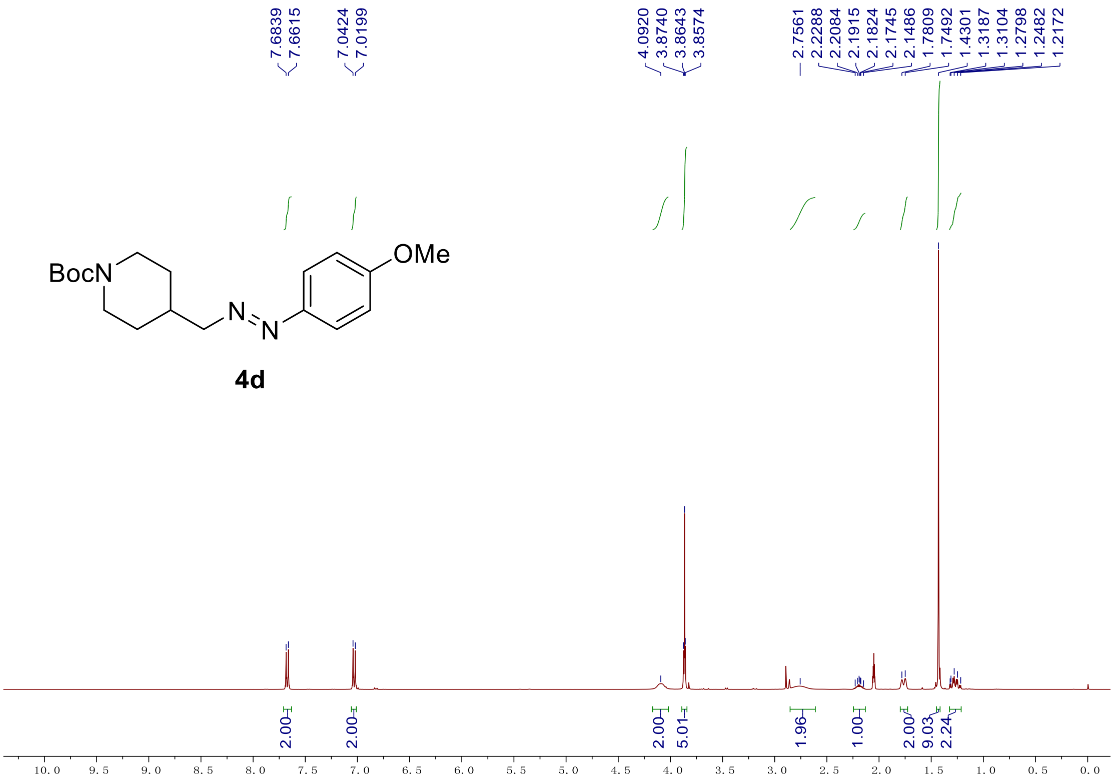

Supplementary Fig. 61. ^{1}H NMR of compound **4d.**

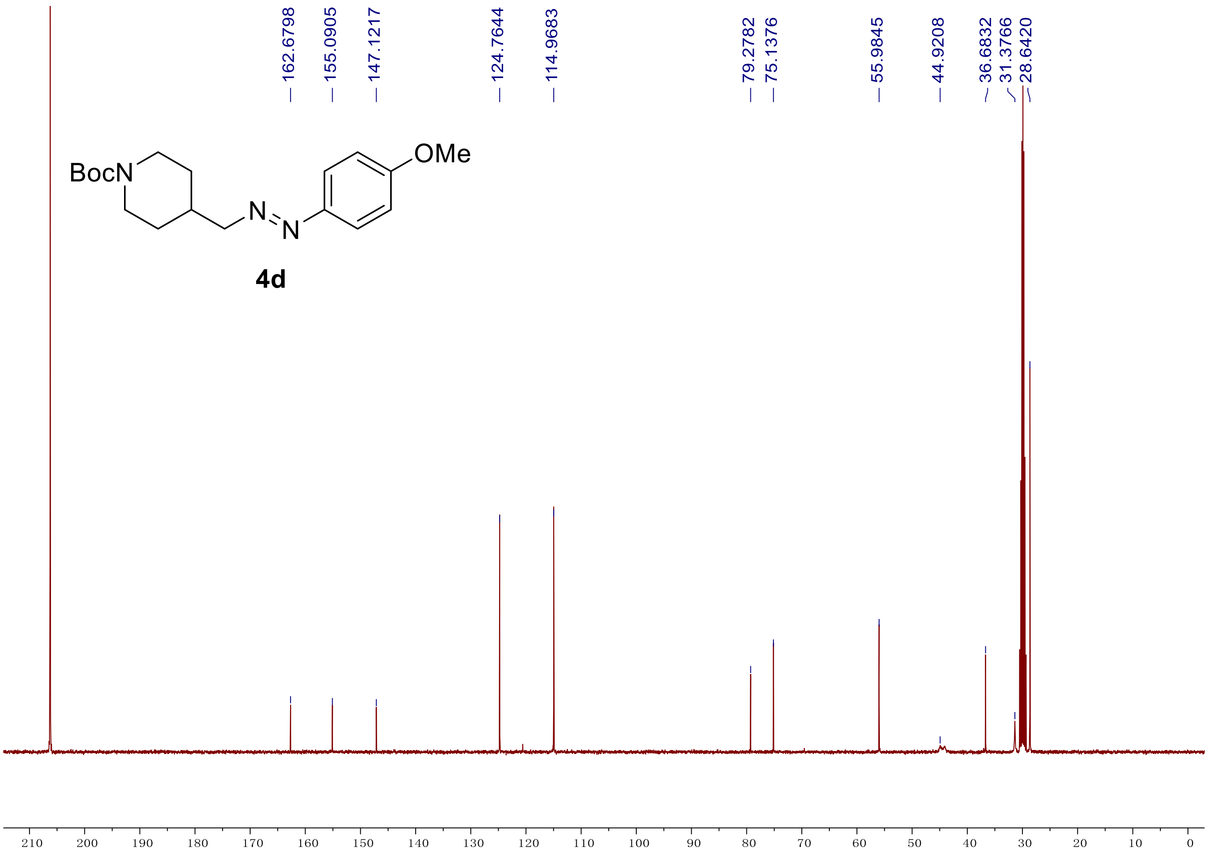

Supplementary Fig. 62. ^{13}C NMR of compound **4d.**

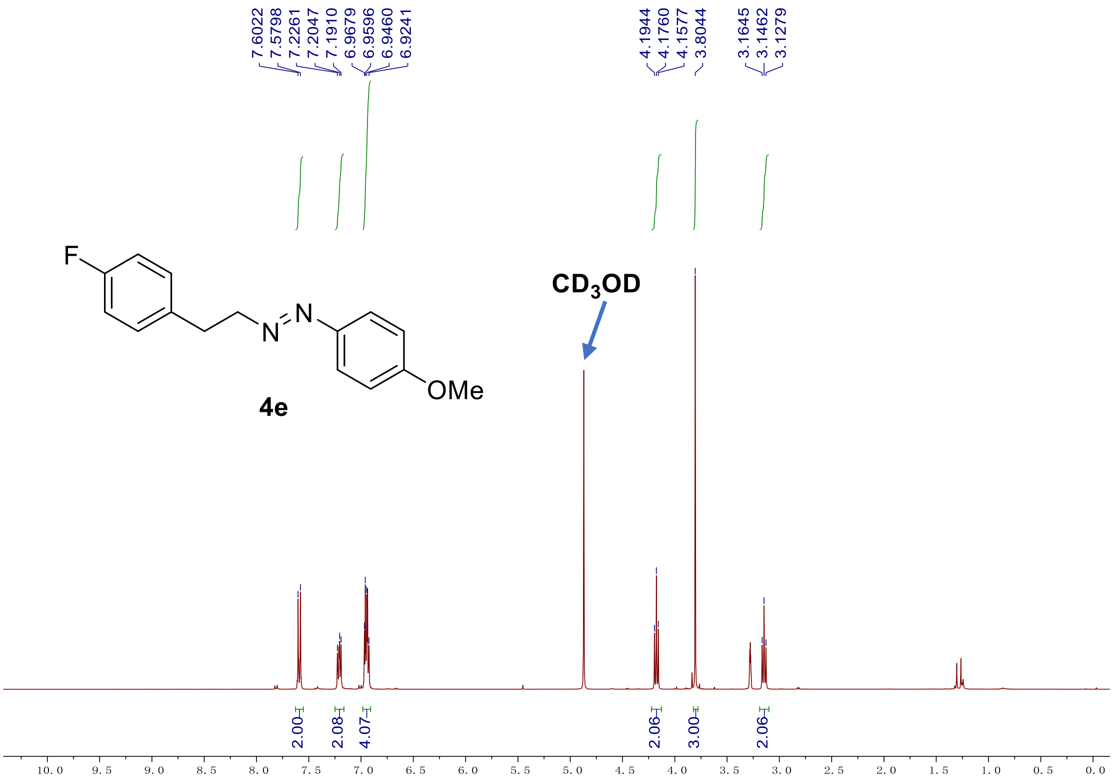

Supplementary Fig. 63. ^{1}H NMR of compound **4e.**

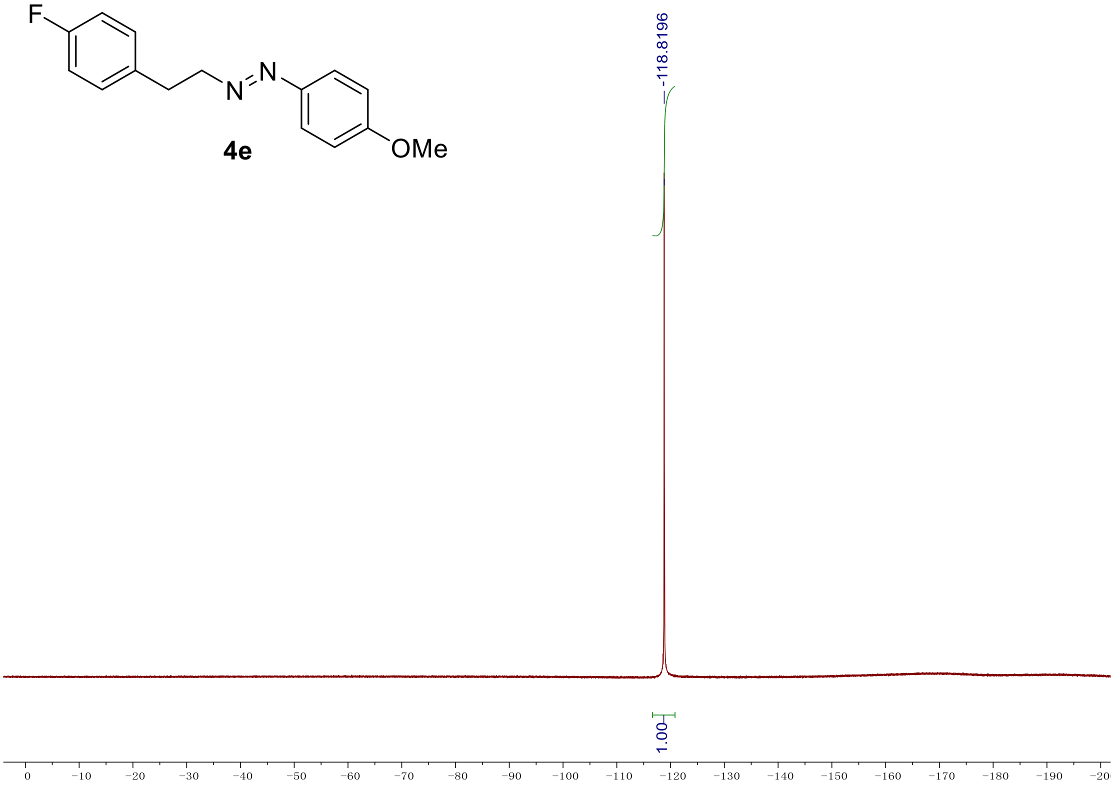

Supplementary Fig. 64. ^{19}F NMR of compound **4e.**

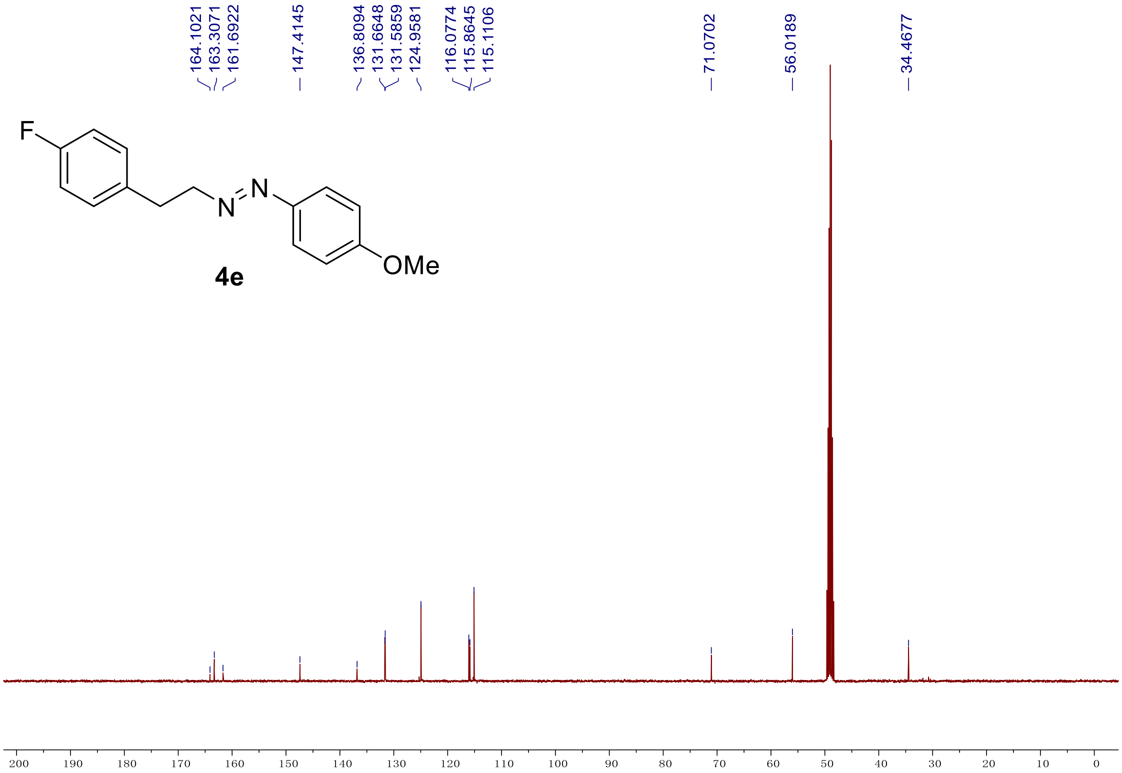

Supplementary Fig. 65. ^{13}C NMR of compound **4e.**

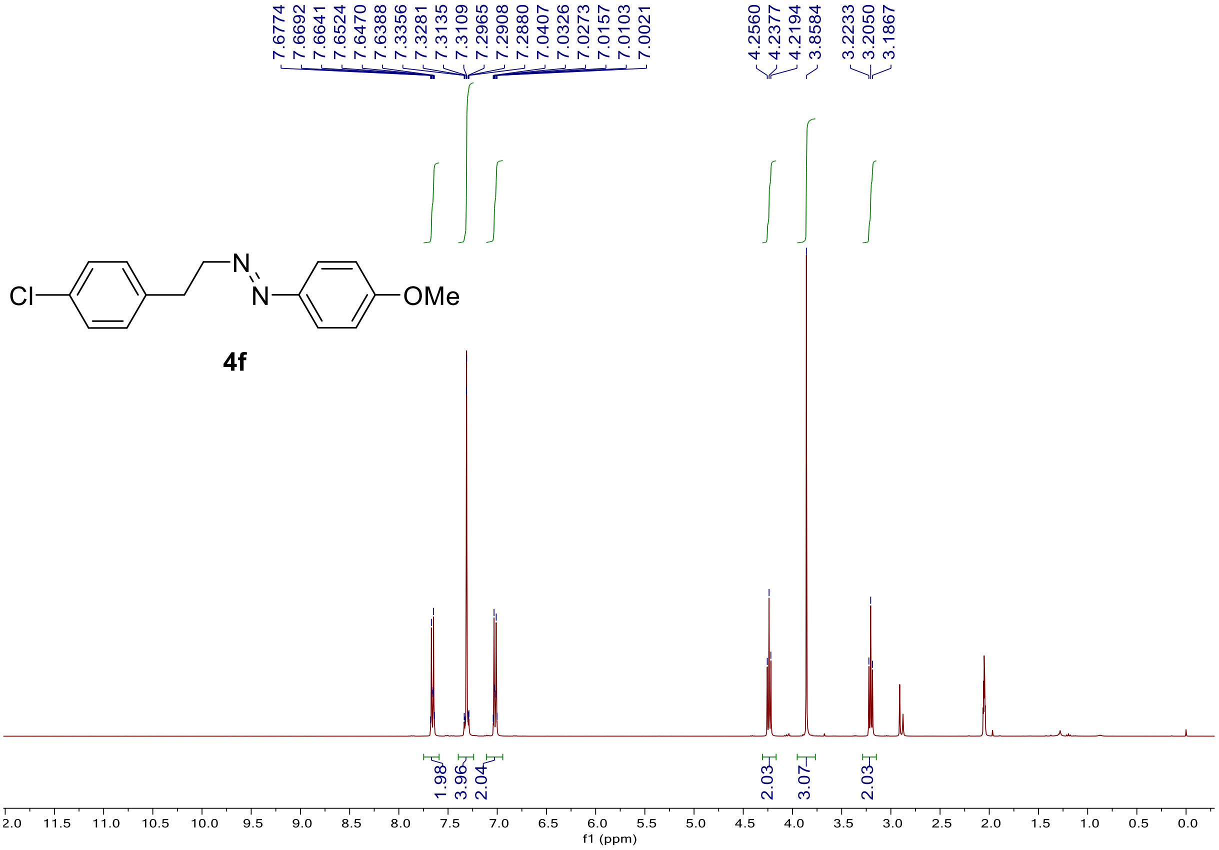

Supplementary Fig. 66. ^{1}H NMR of compound **4f.**

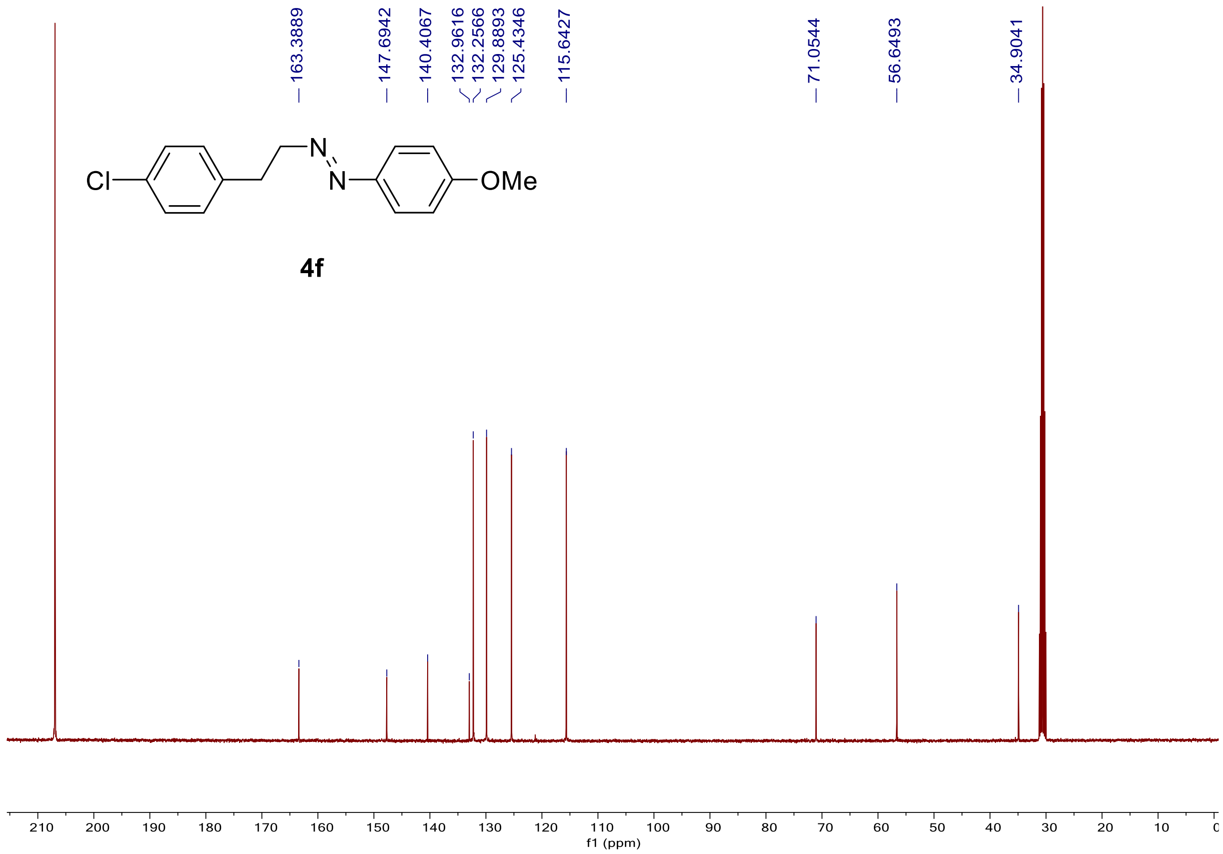

Supplementary Fig. 67. ^{13}C NMR of compound **4f.**

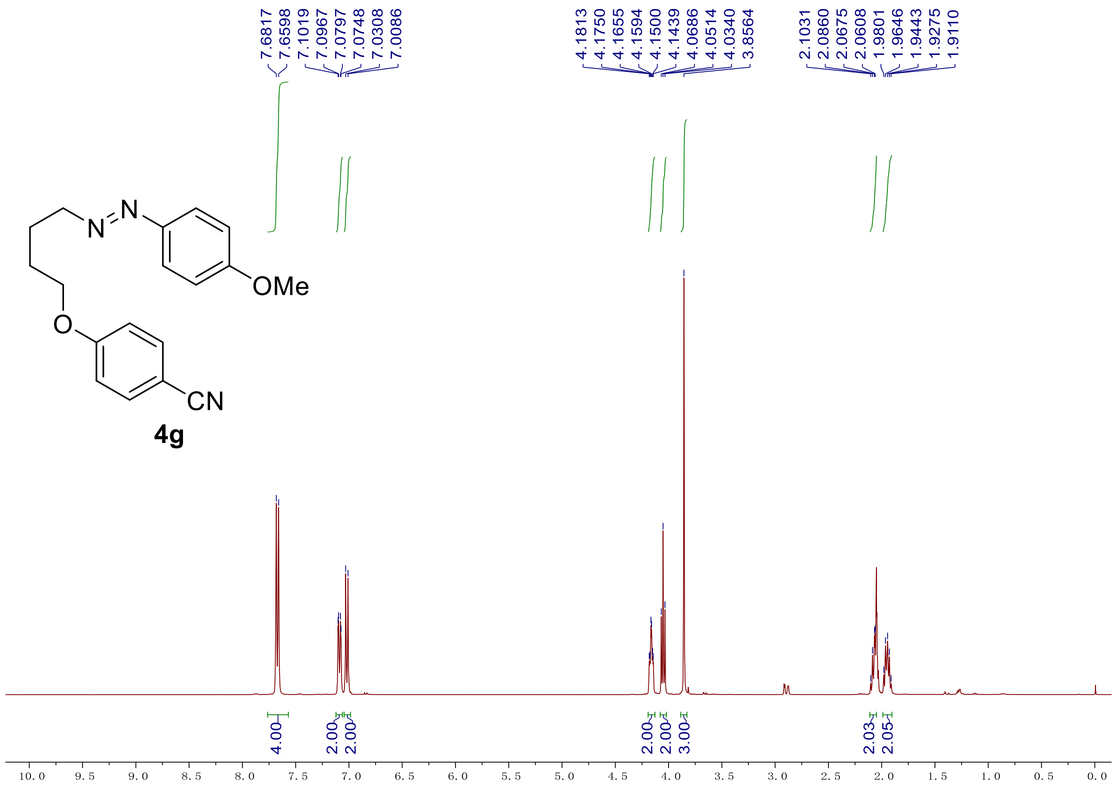

Supplementary Fig. 68. ^{1}H NMR of compound **4g.**

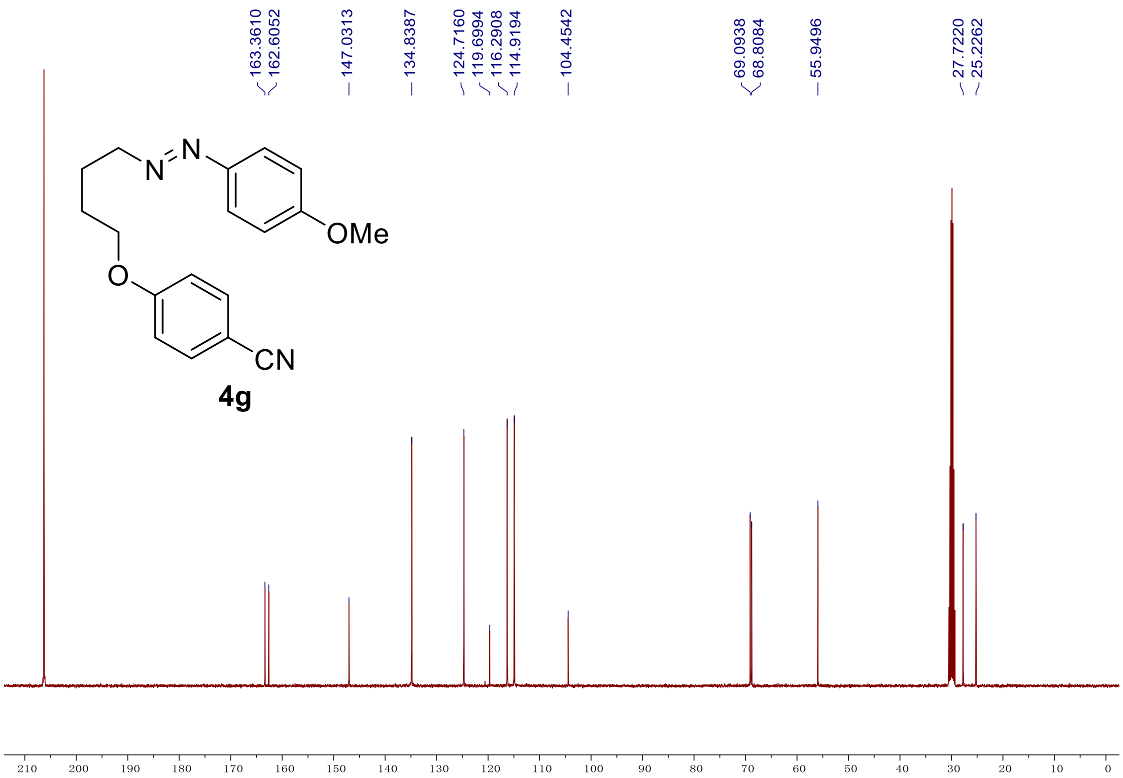

Supplementary Fig. 69. ^{13}C NMR of compound **4g.**

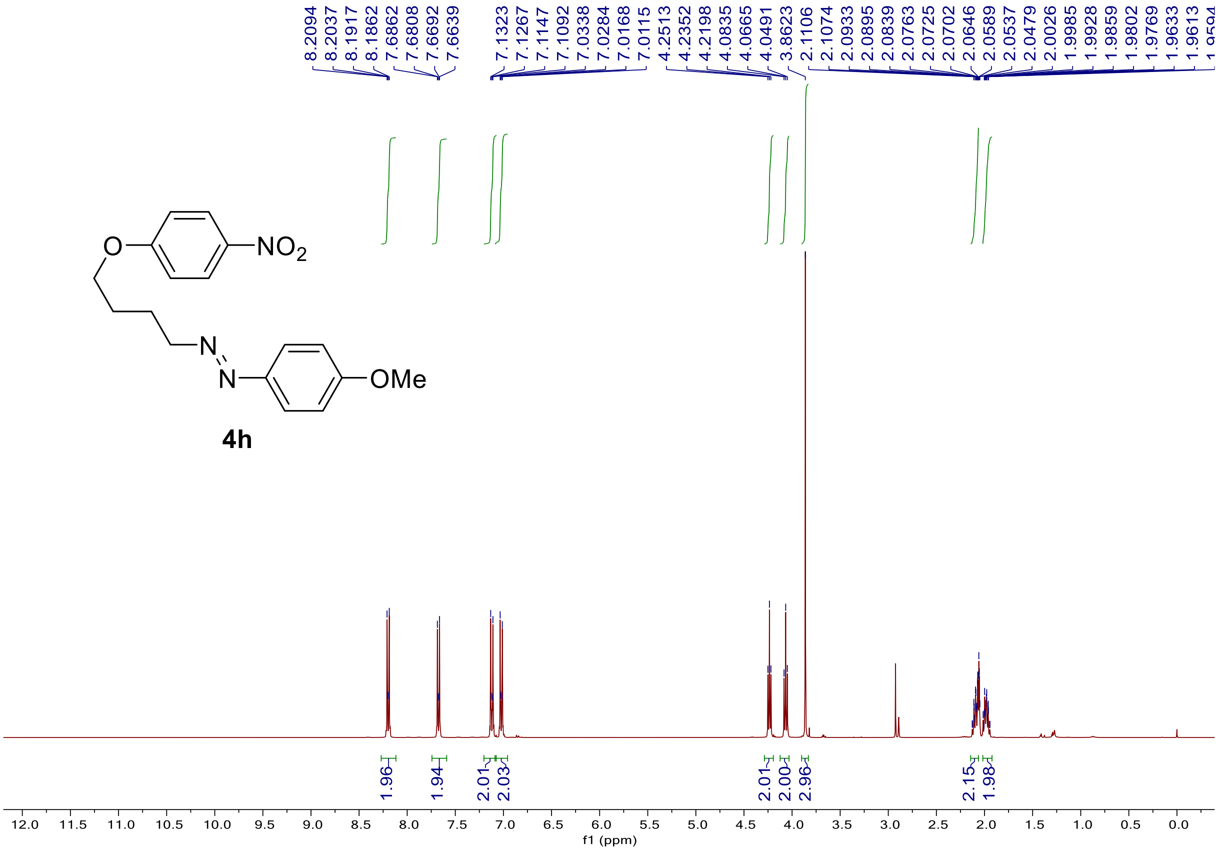

Supplementary Fig. 70. ^{1}H NMR of compound **4h.**

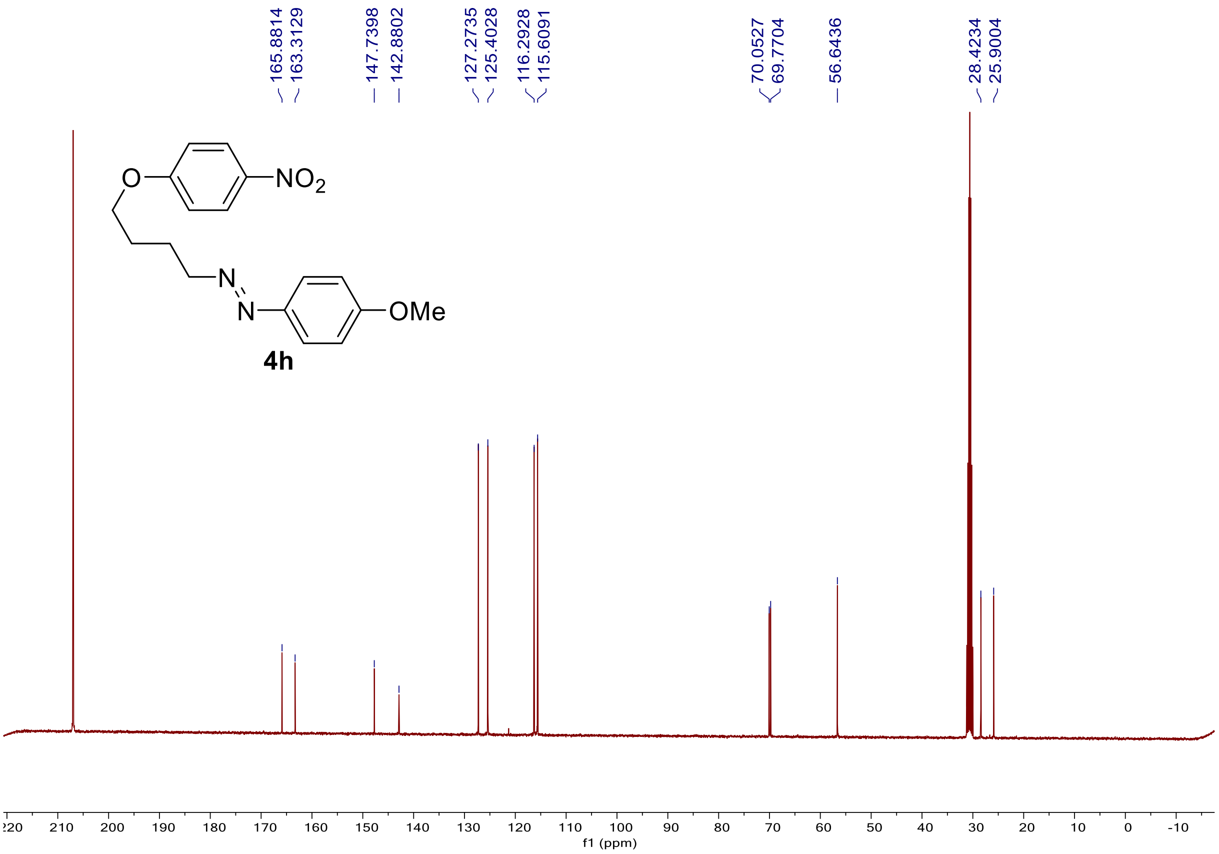

Supplementary Fig. 71. ^{13}C NMR of compound **4h.**

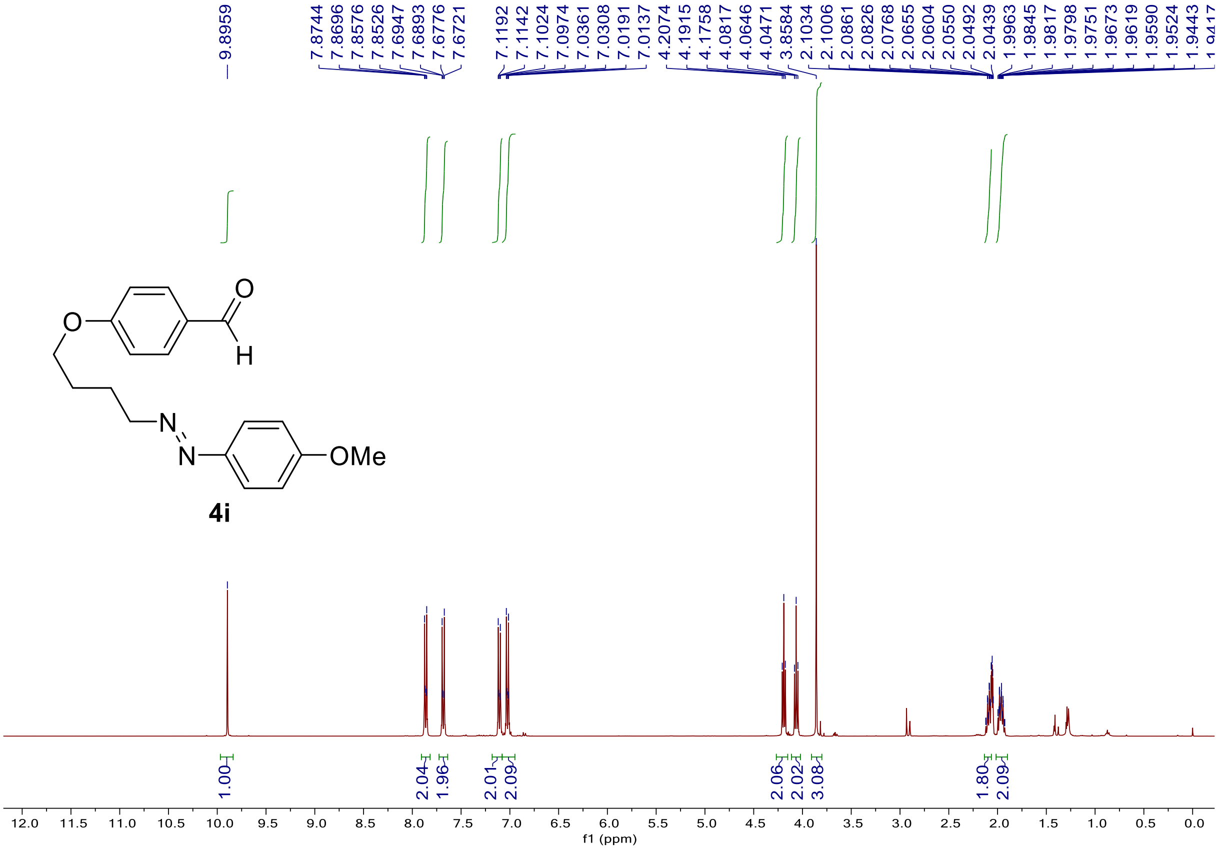

Supplementary Fig. 72. ^{1}H NMR of compound **4i.**

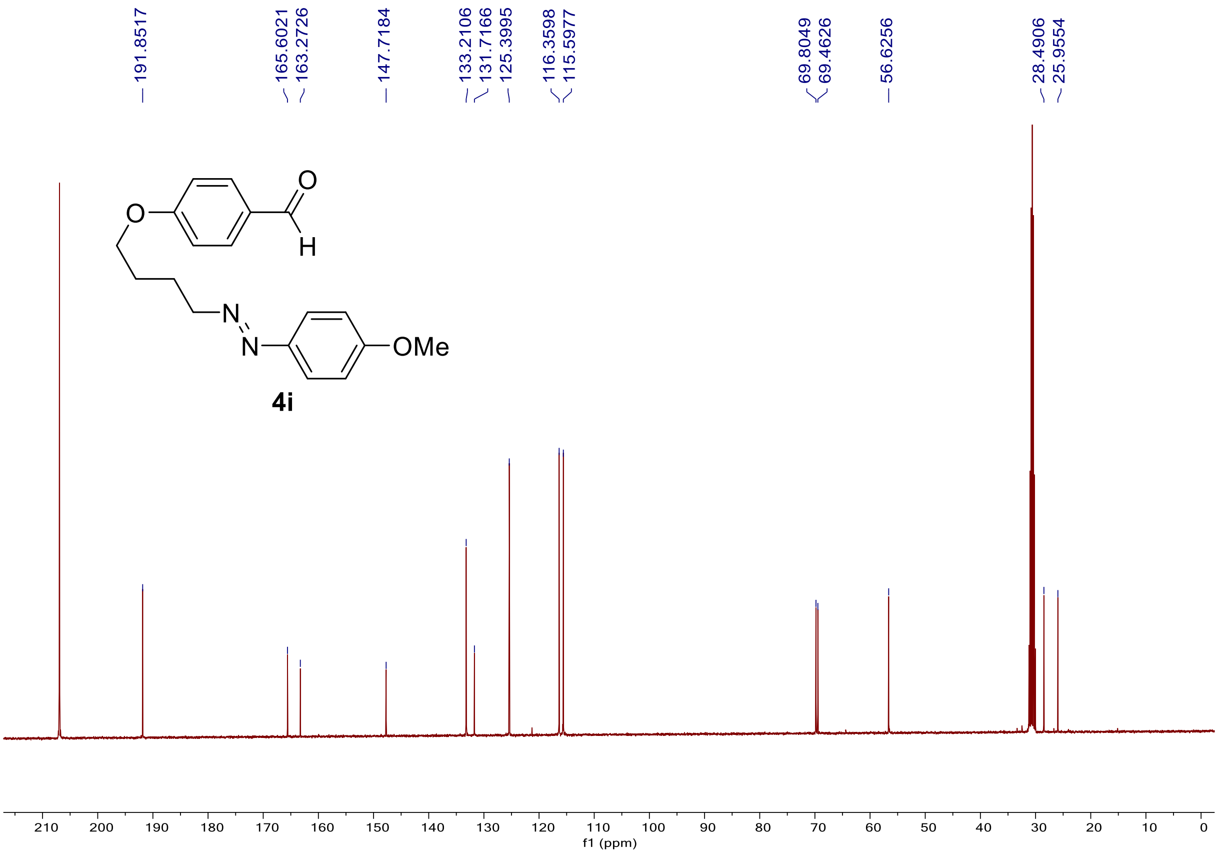

Supplementary Fig. 73. ^{13}C NMR of compound **4i.**

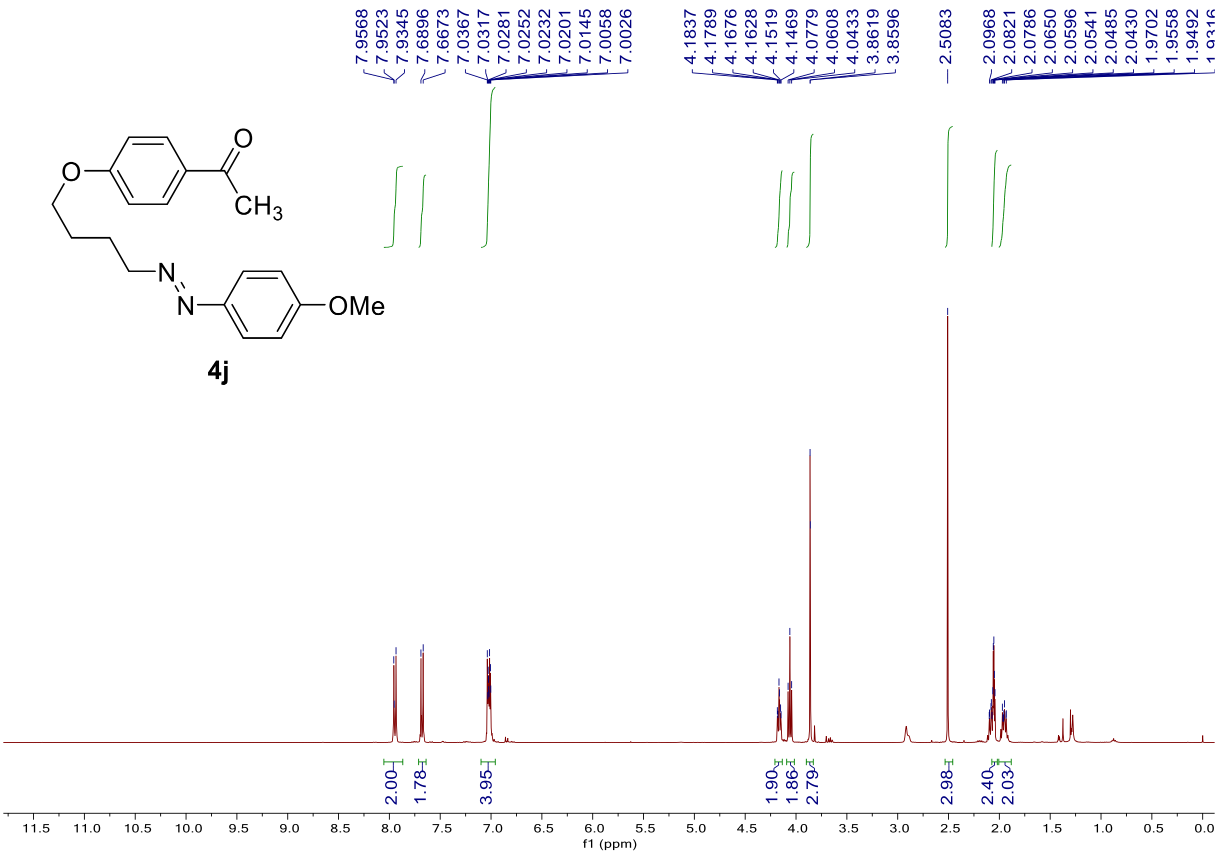

Supplementary Fig. 74. ^{1}H NMR of compound **4j.**

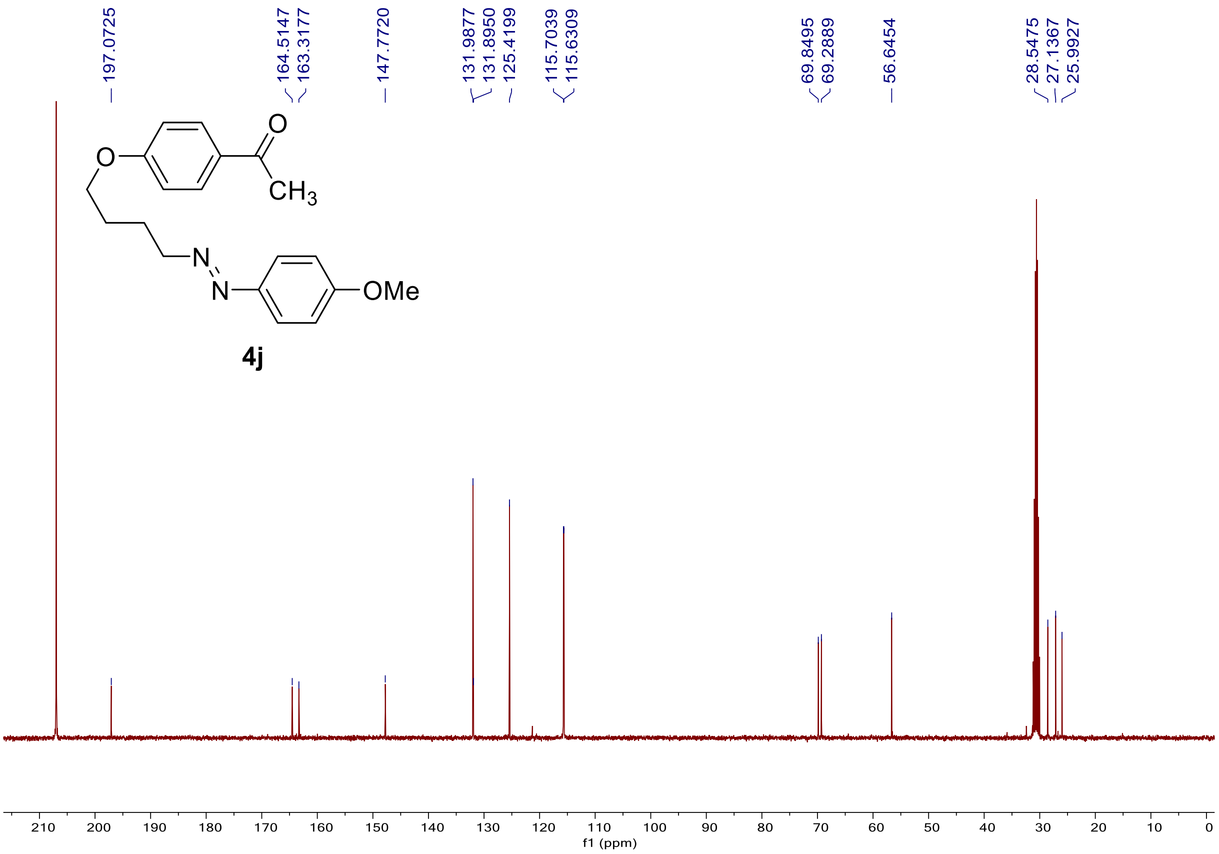

Supplementary Fig. 75. ^{13}C NMR of compound **4j.**

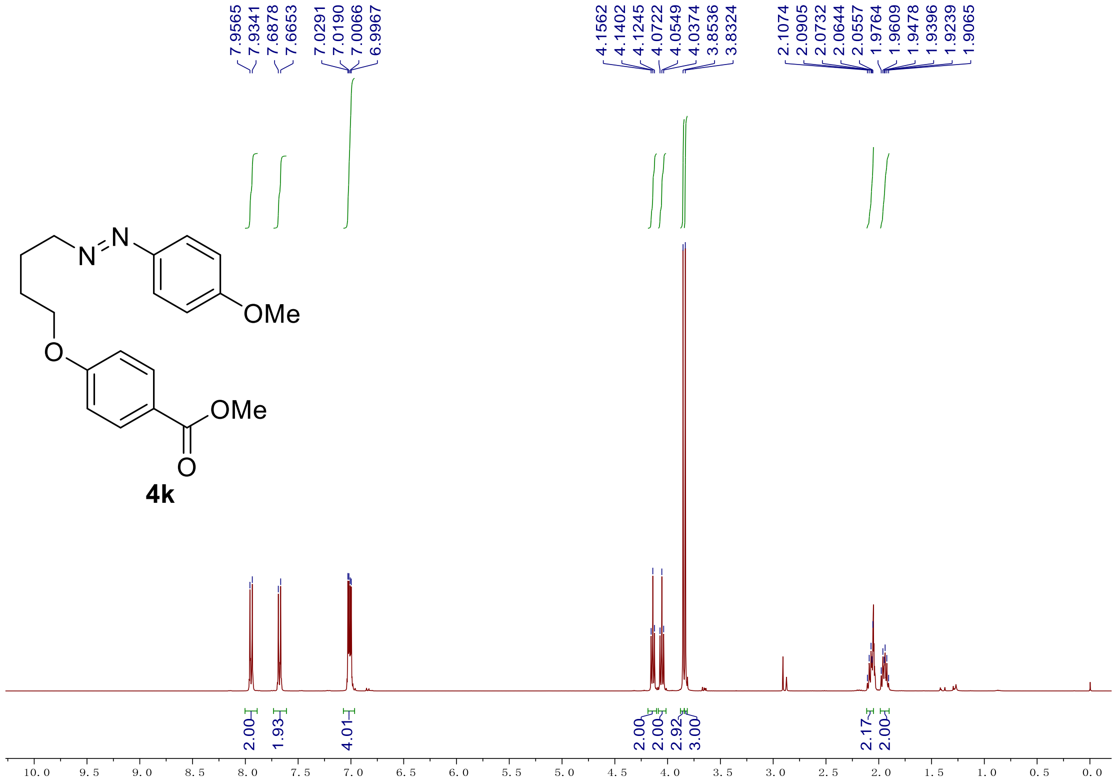

Supplementary Fig. 76. ^{1}H NMR of compound **4k.**

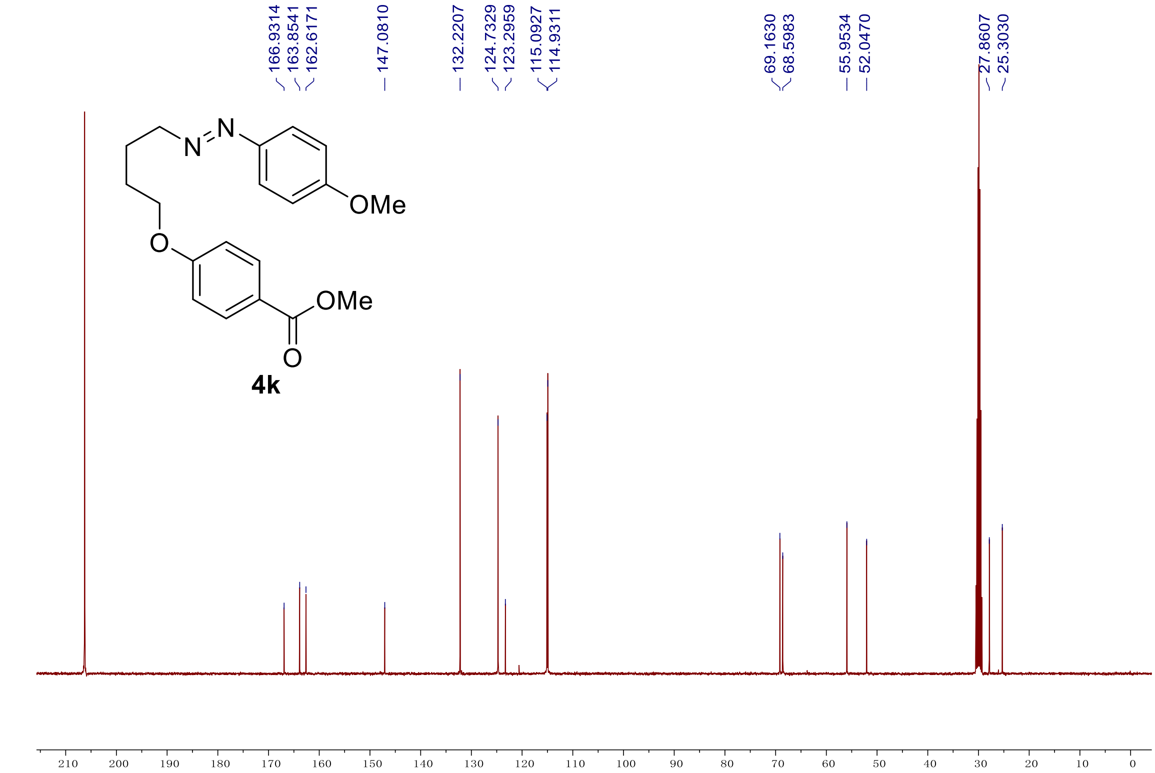

Supplementary Fig. 77. ^{13}C NMR of compound **4k.**

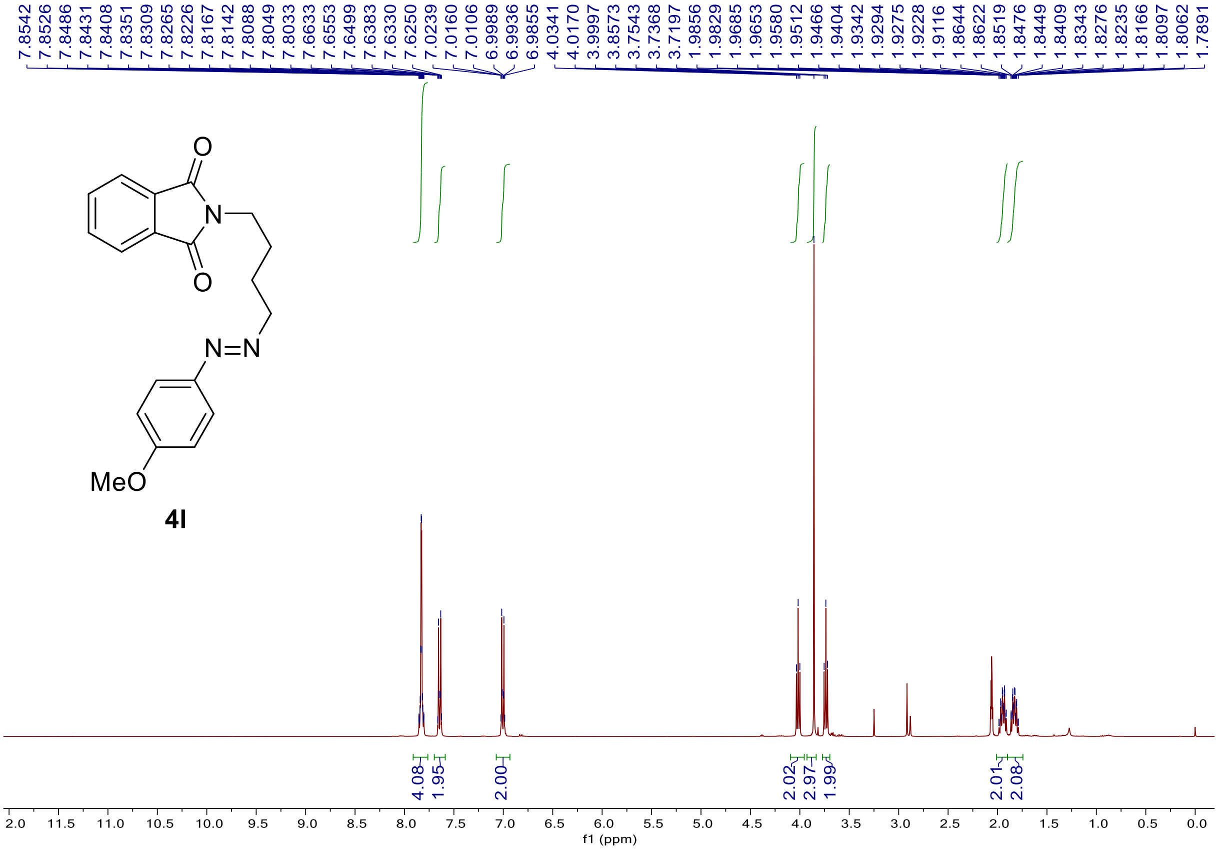

Supplementary Fig. 78. ^{1}H NMR of compound **4l.**

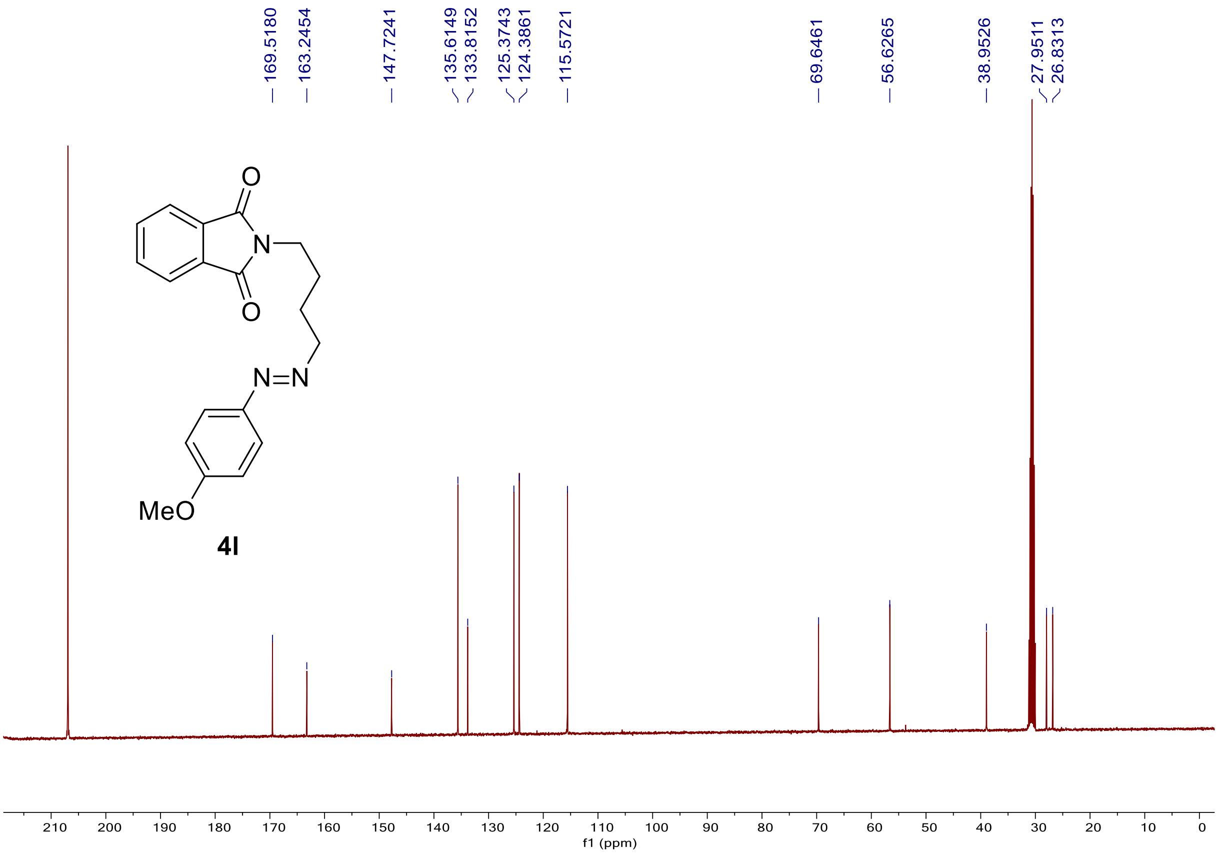

Supplementary Fig. 79. ^{13}C NMR of compound **4l.**

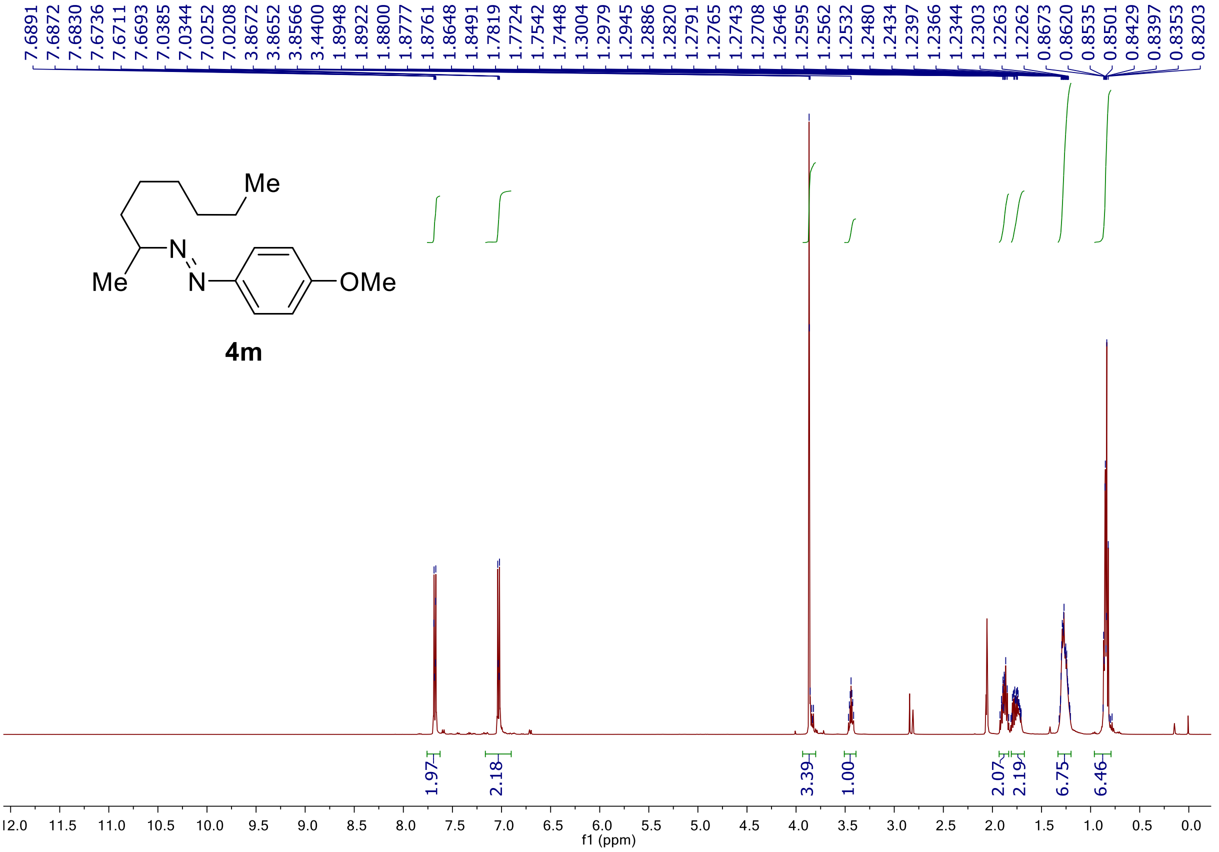

Supplementary Fig. 80. ^{1}H NMR of compound **4m.**

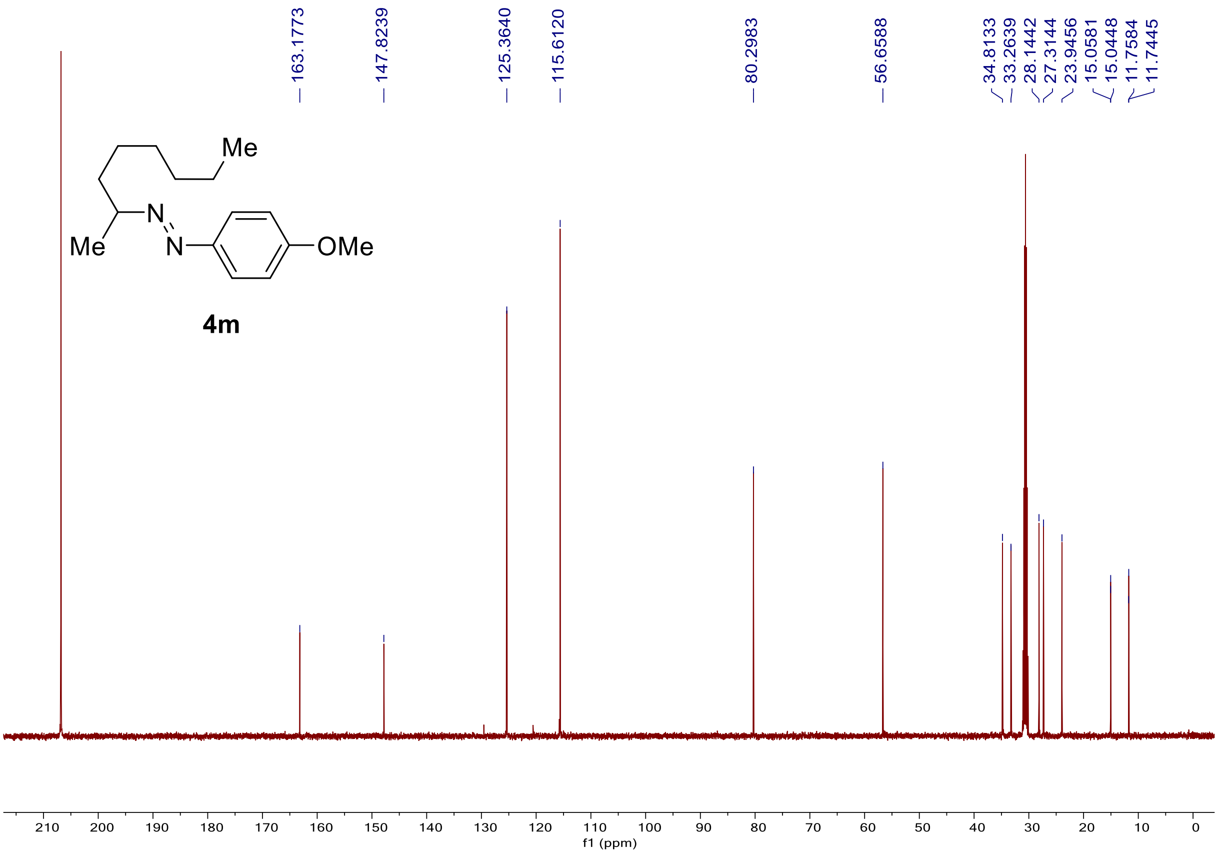

Supplementary Fig. 81. ^{13}C NMR of compound **4m.**

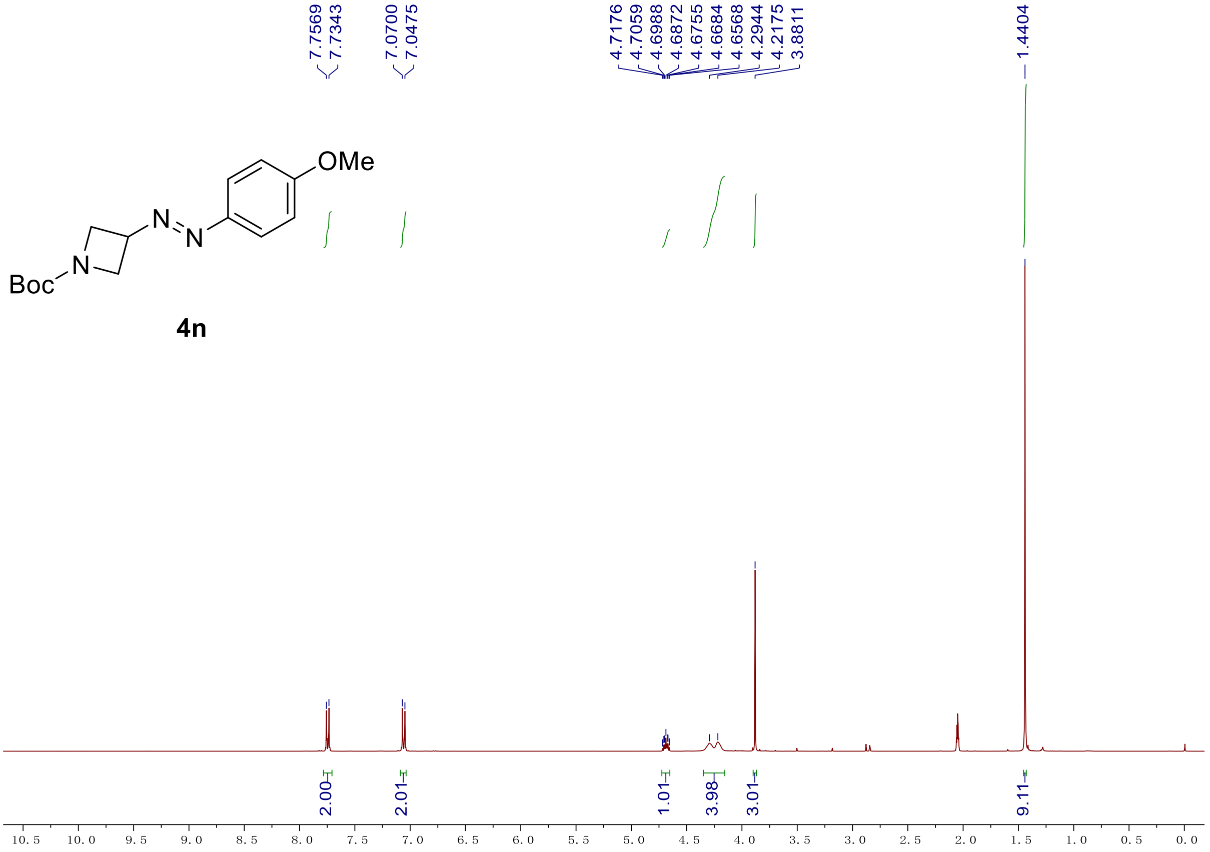

Supplementary Fig. 82. ^{1}H NMR of compound **4n.**

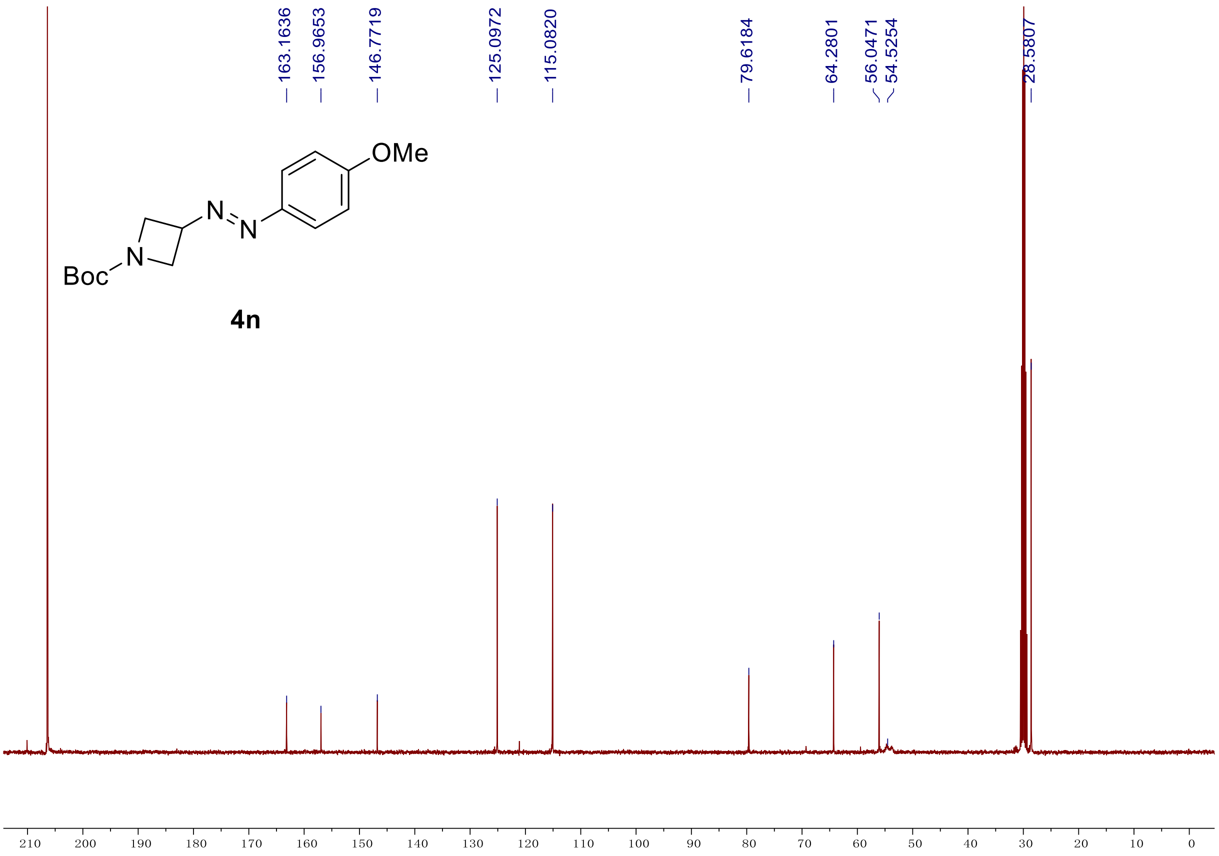

Supplementary Fig. 83. ^{13}C NMR of compound **4n.**

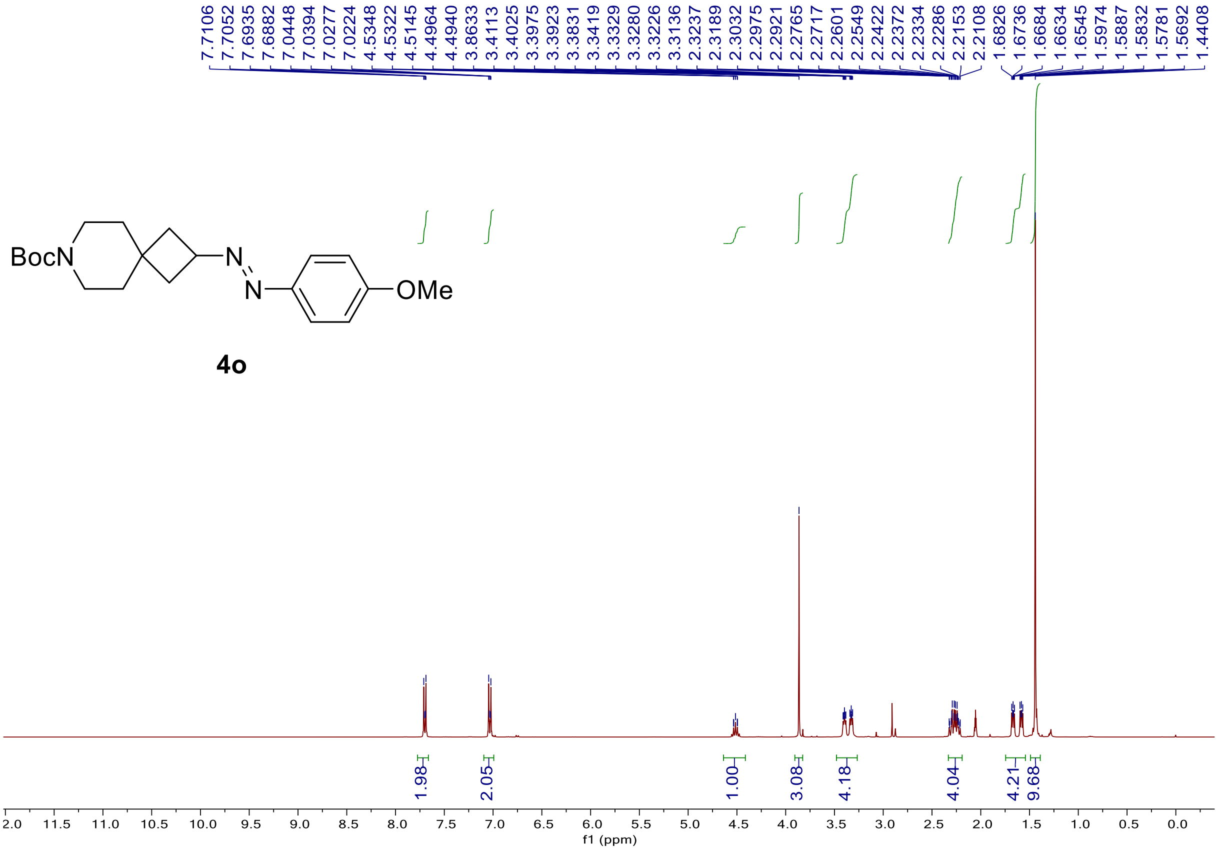

Supplementary Fig. 84. ^{1}H NMR of compound **4o.**

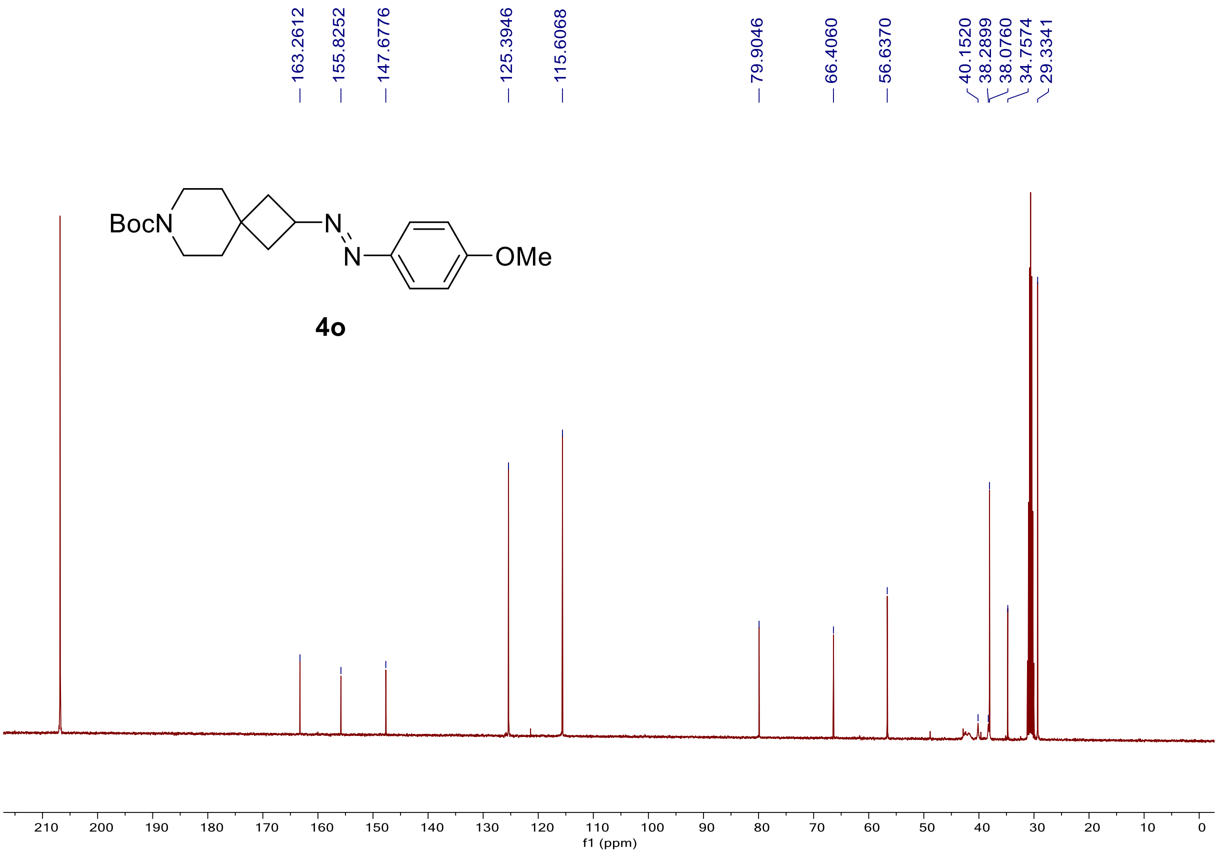

Supplementary Fig. 85. ^{13}C NMR of compound **4o.**

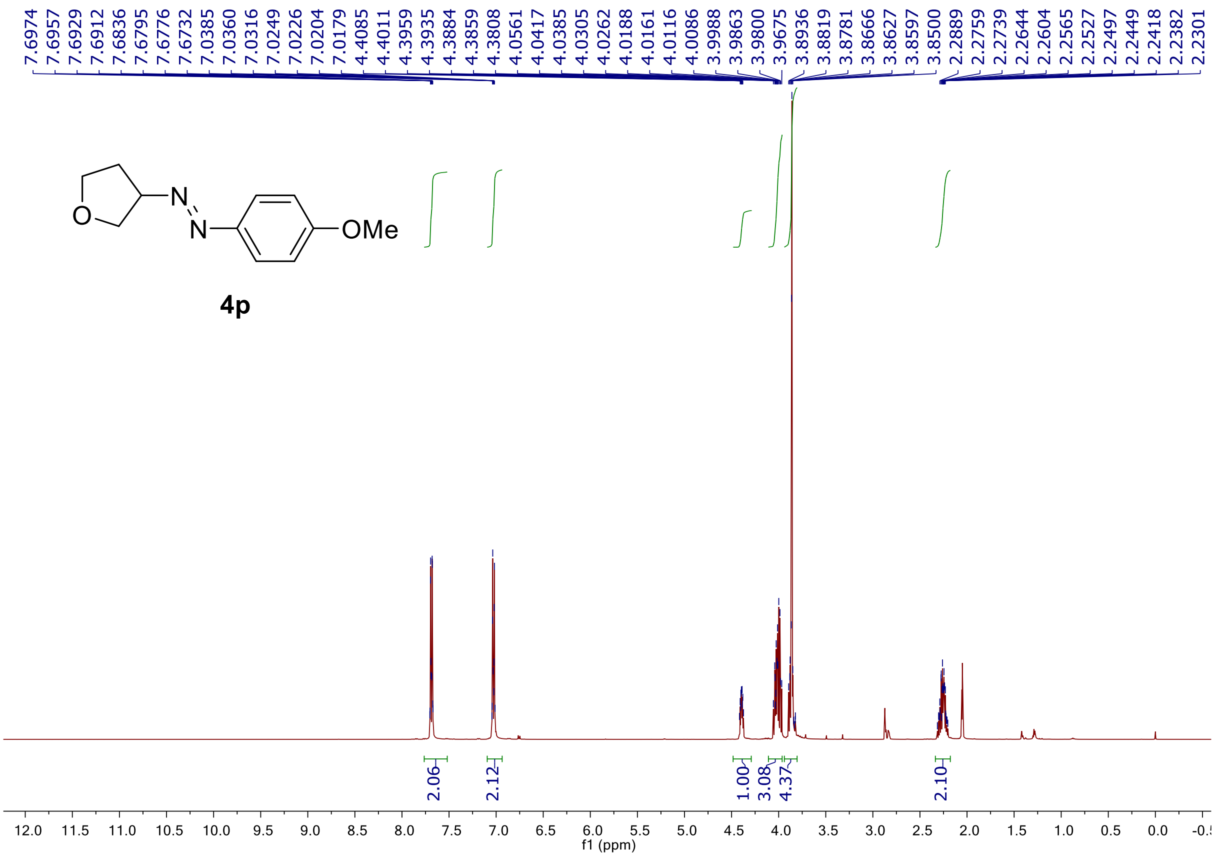

Supplementary Fig. 86. ^{1}H NMR of compound **4p.**

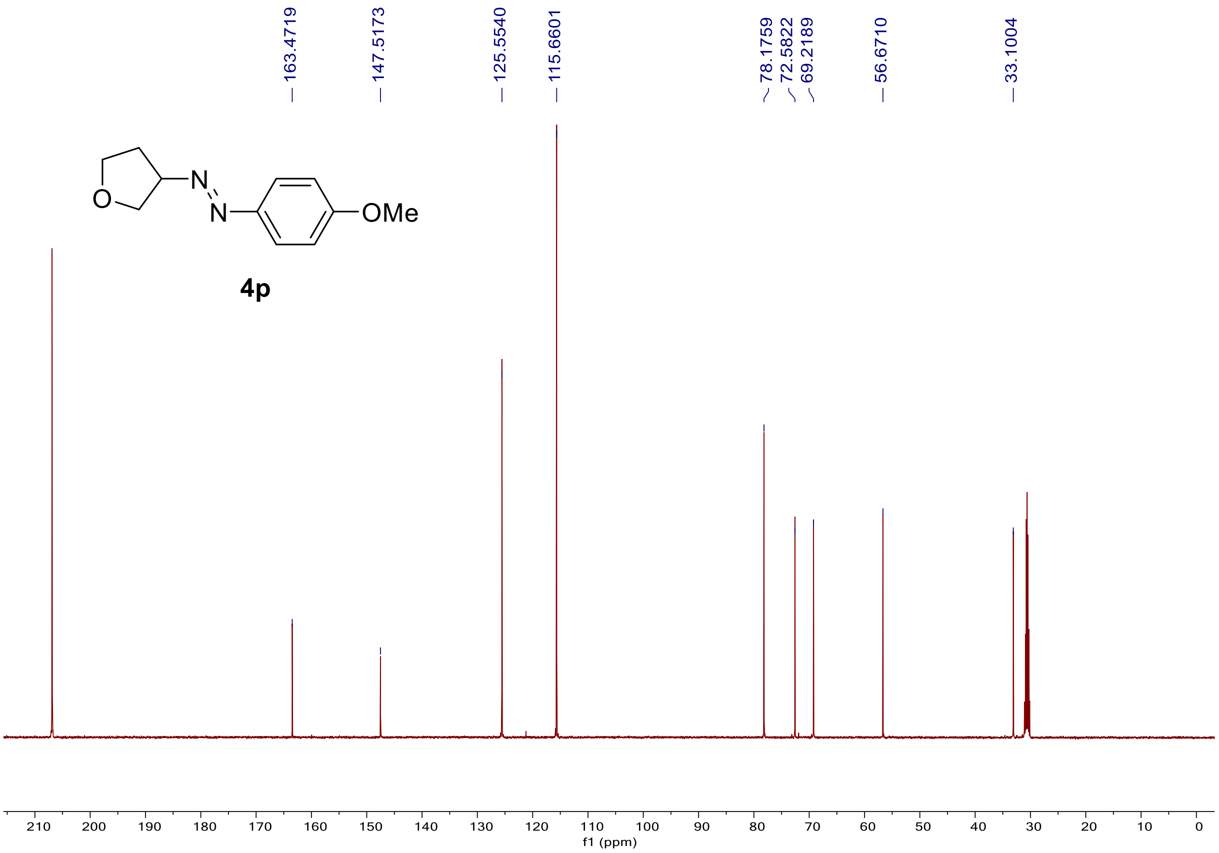

Supplementary Fig. 87. ^{13}C NMR of compound **4p.**

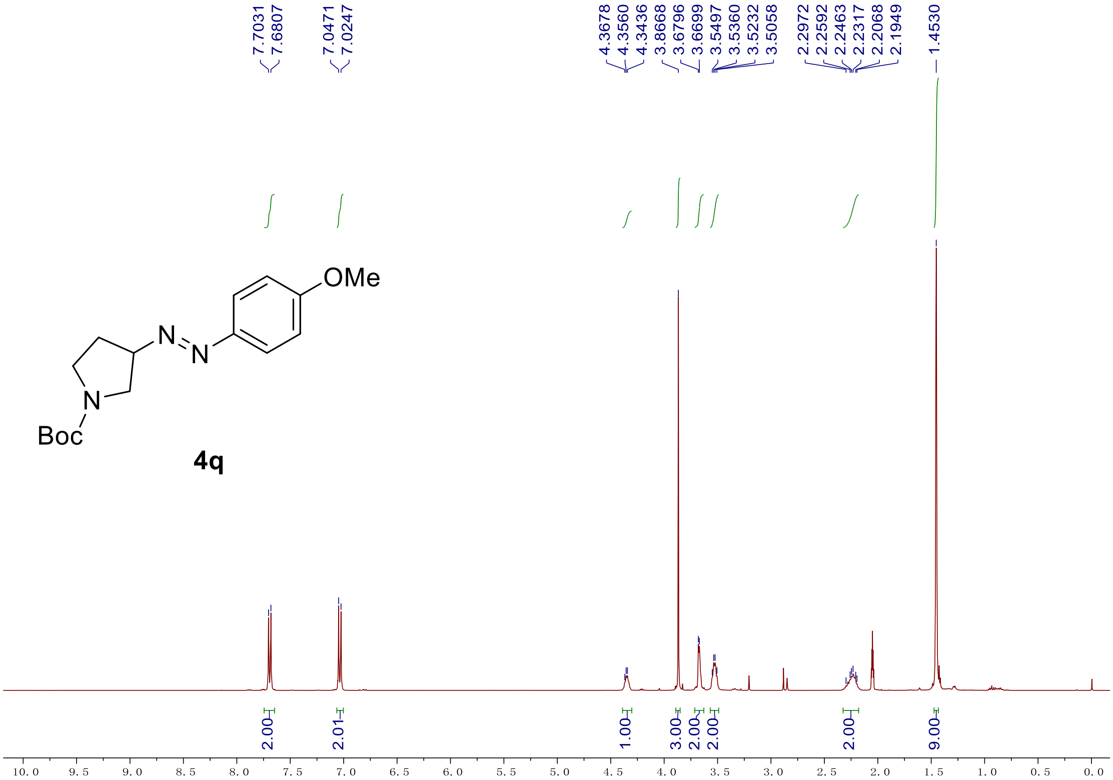

Supplementary Fig. 88. ^{1}H NMR of compound **4q.**

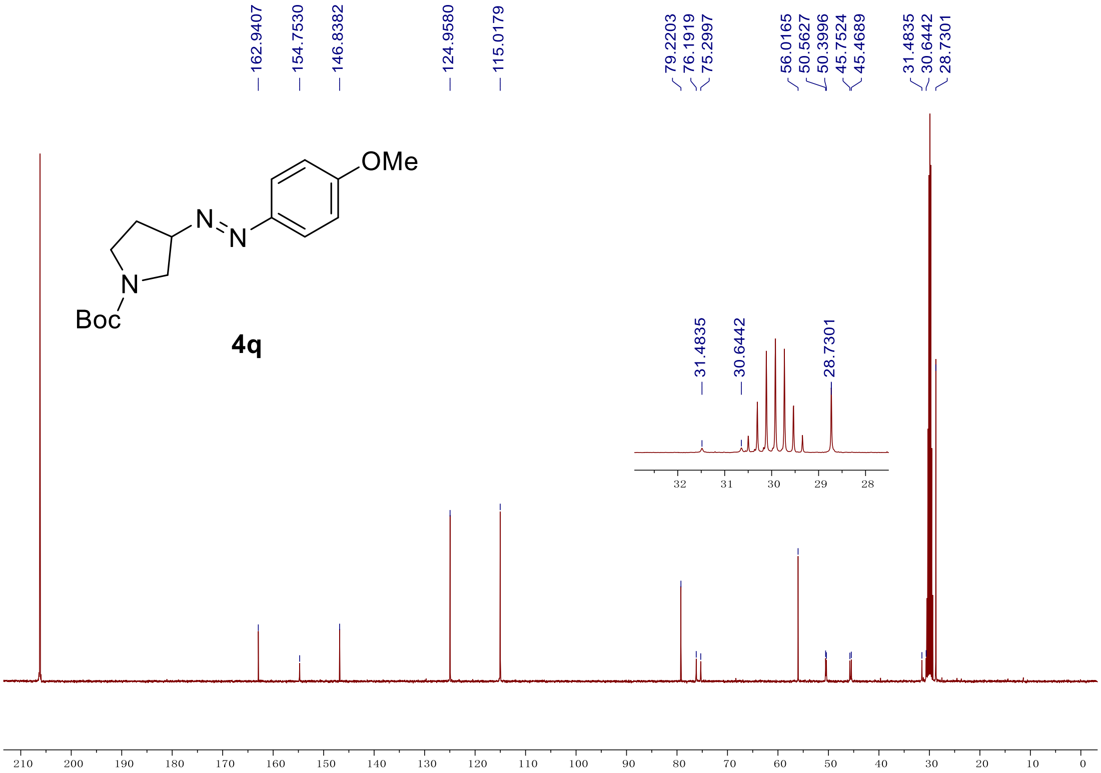

Supplementary Fig. 89. ^{13}C NMR of compound **4q.**

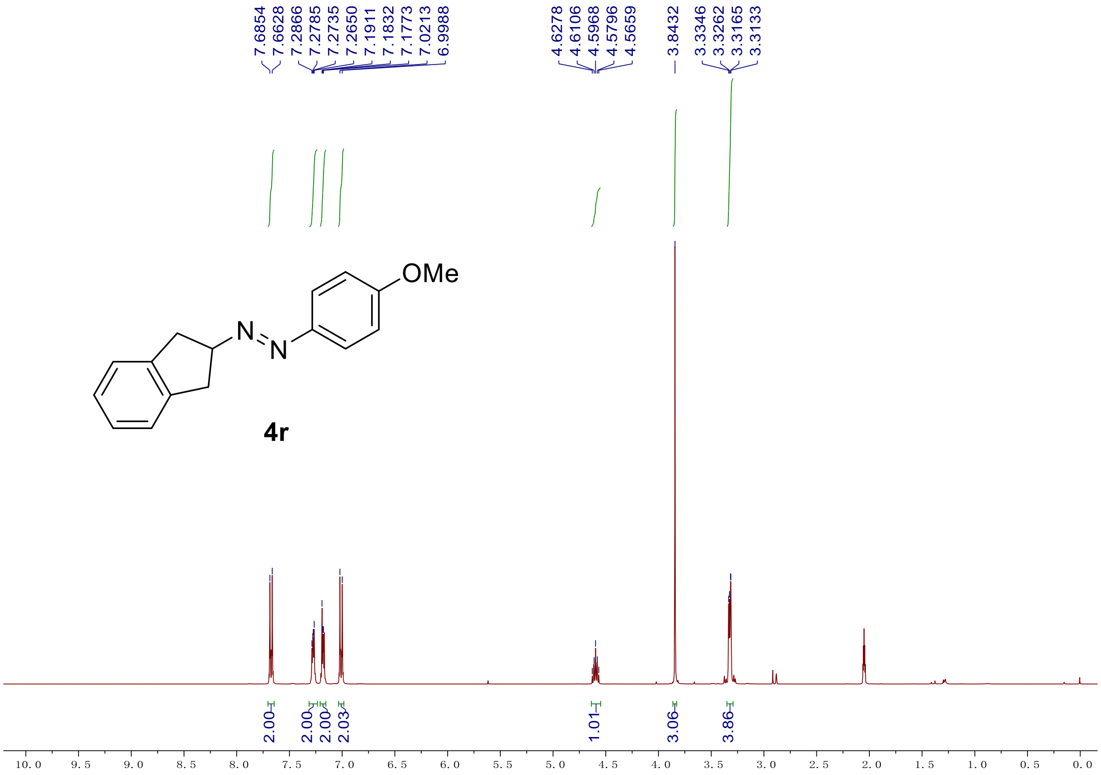

Supplementary Fig. 90. ^{1}H NMR of compound **4r.**

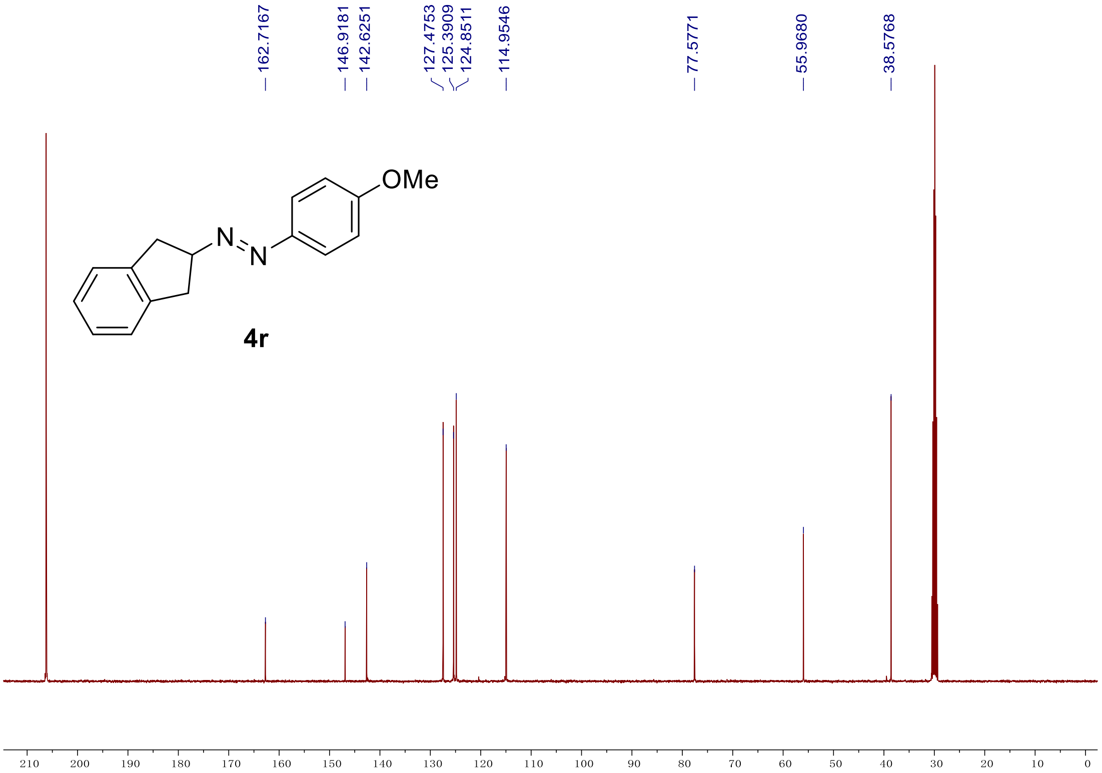

Supplementary Fig. 91. ^{13}C NMR of compound **4r.**

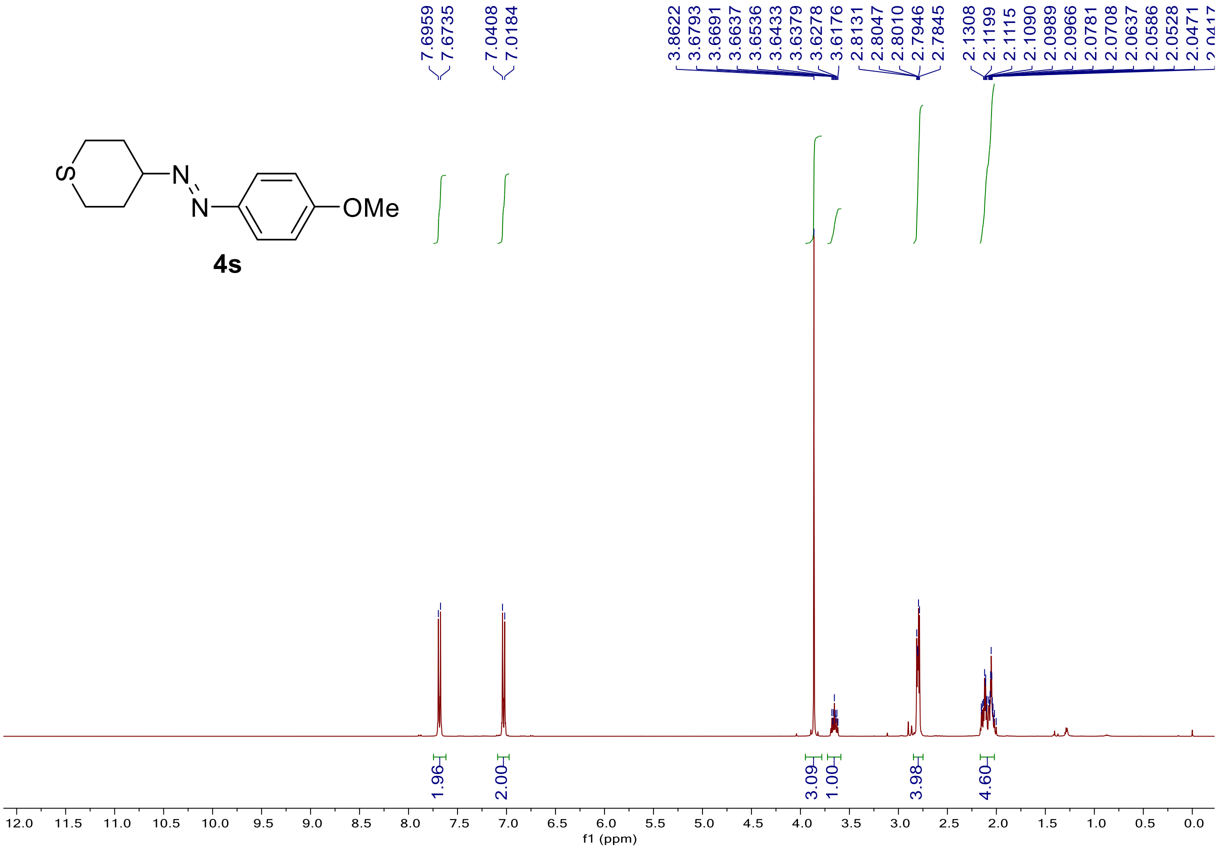

Supplementary Fig. 92. ^{1}H NMR of compound **4s.**

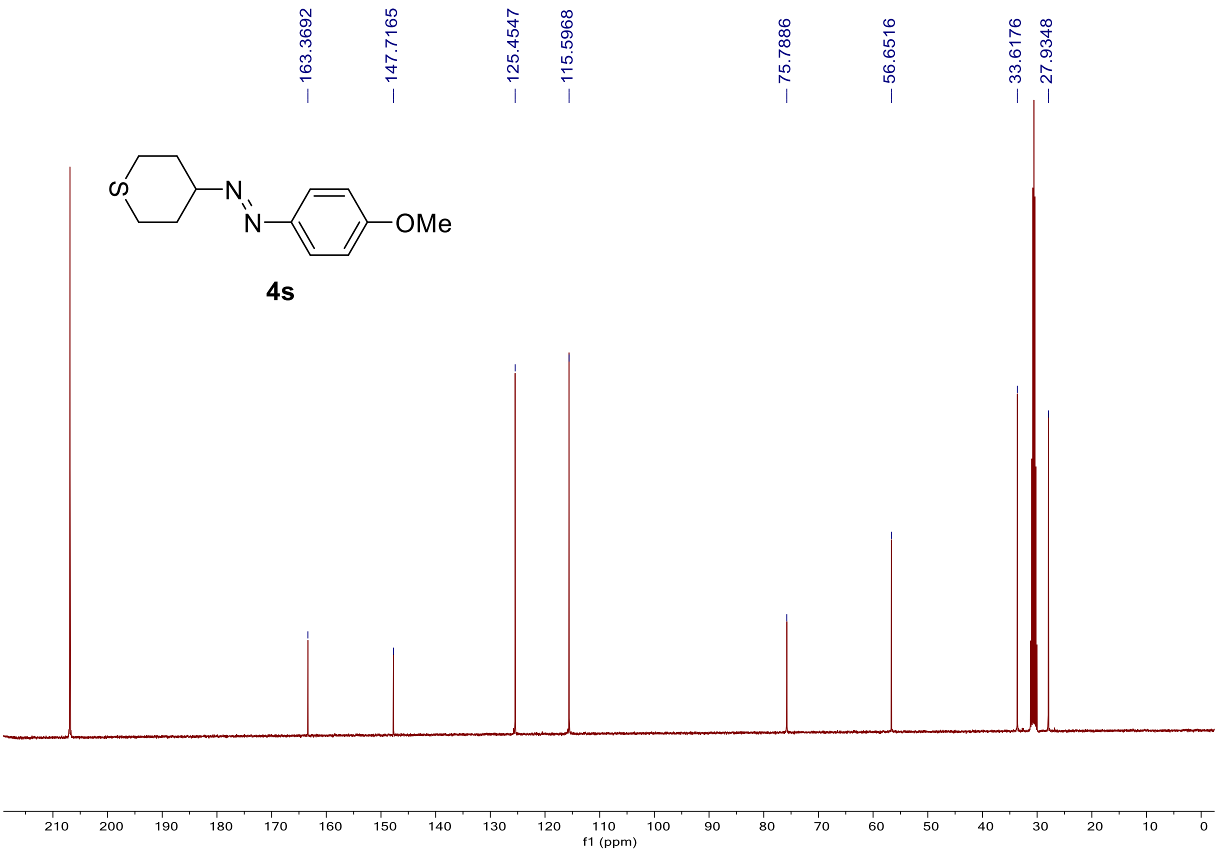

Supplementary Fig. 93. ^{13}C NMR of compound **4s.**

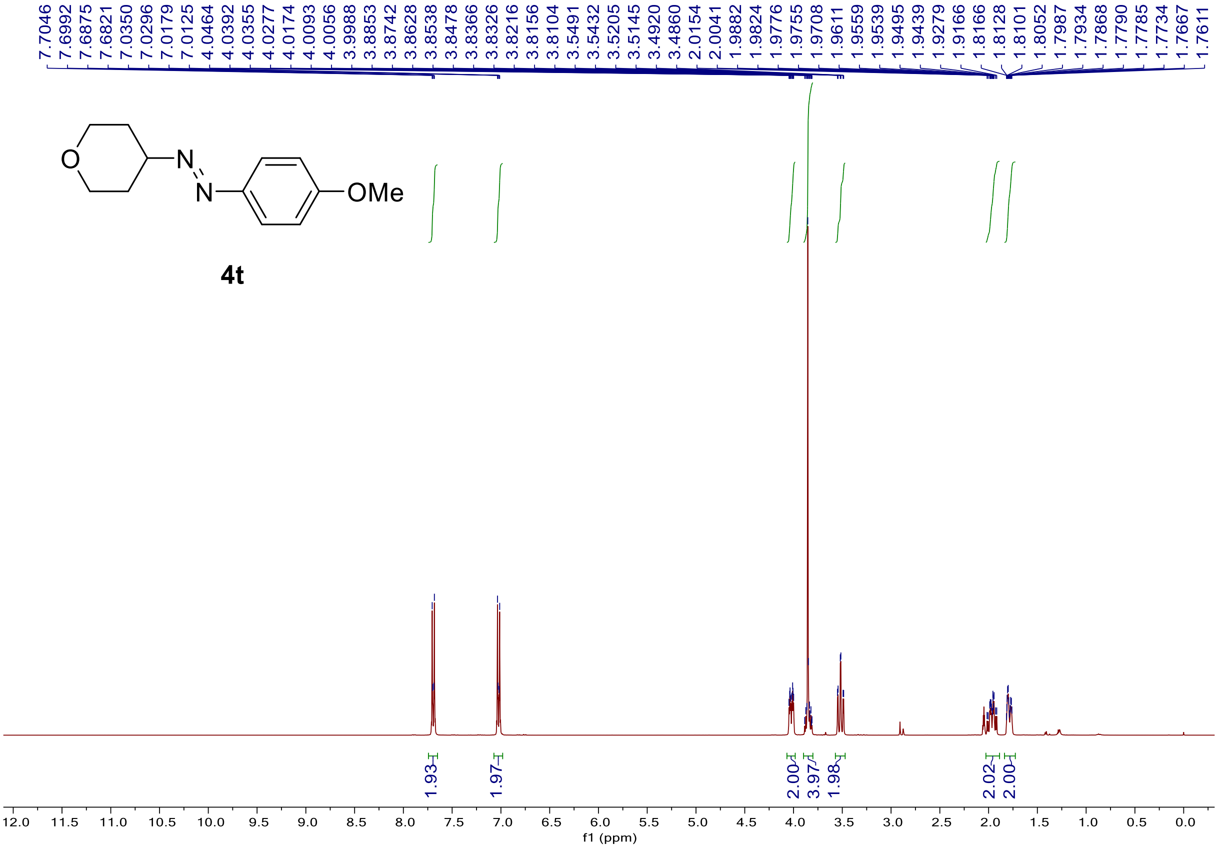

Supplementary Fig. 94. ^{1}H NMR of compound **4t.**

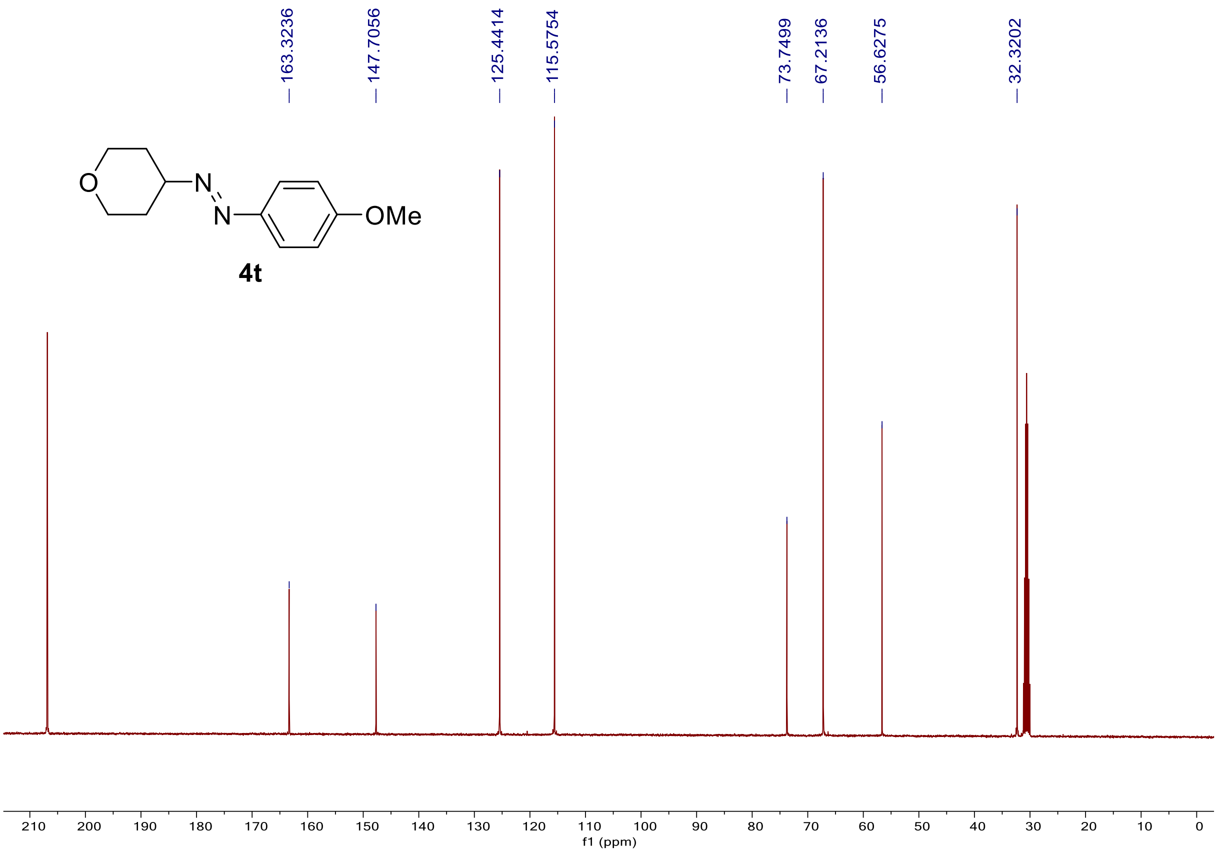

Supplementary Fig. 95. ^{13}C NMR of compound **4t.**

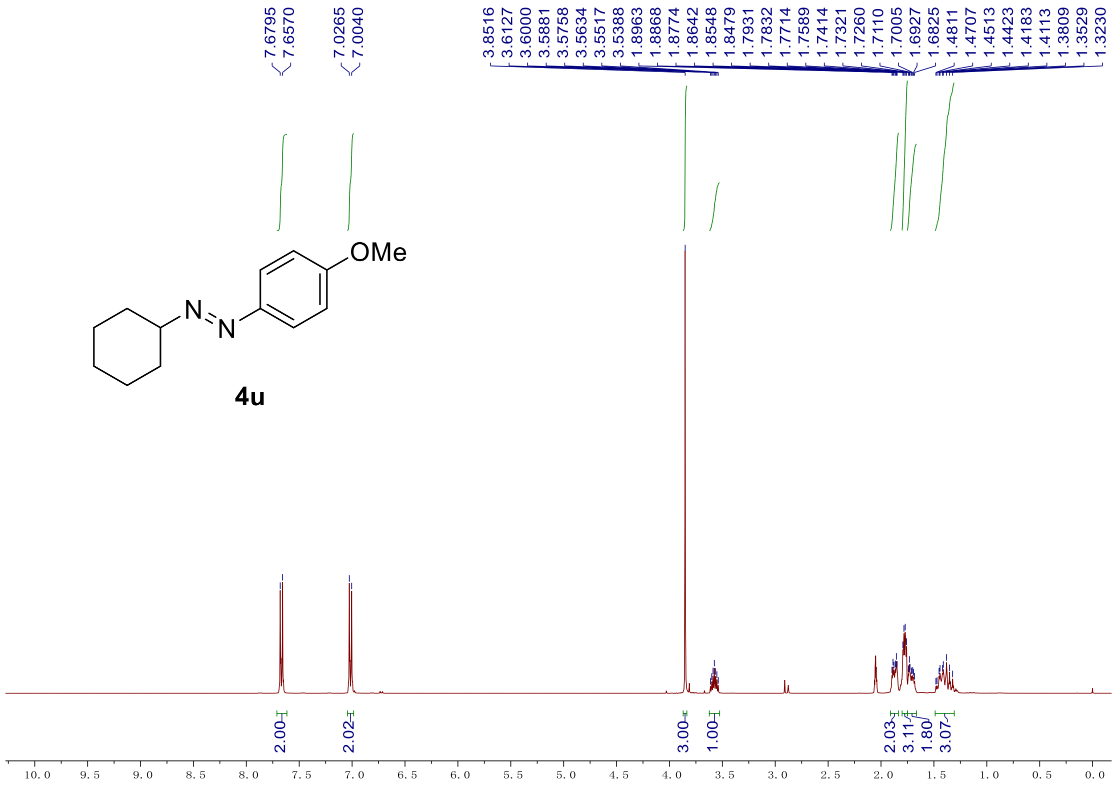

Supplementary Fig. 96. ^{1}H NMR of compound **4u.**

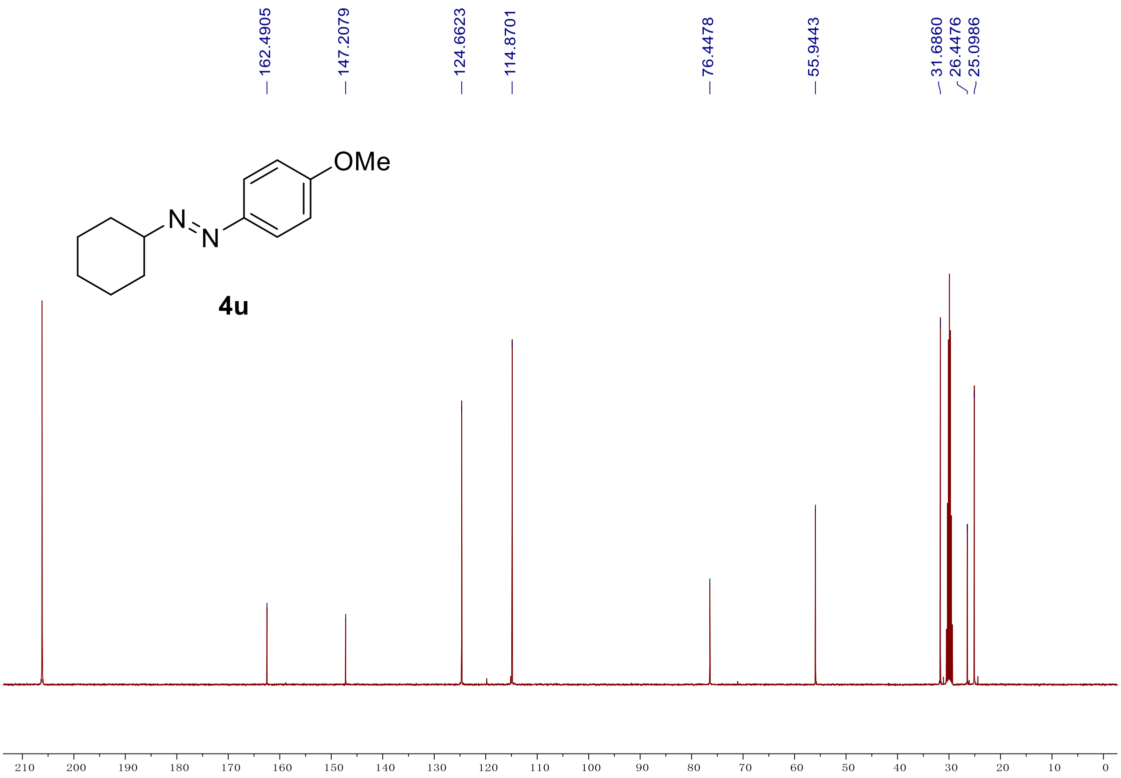

Supplementary Fig. 97. ^{13}C NMR of compound **4u.**

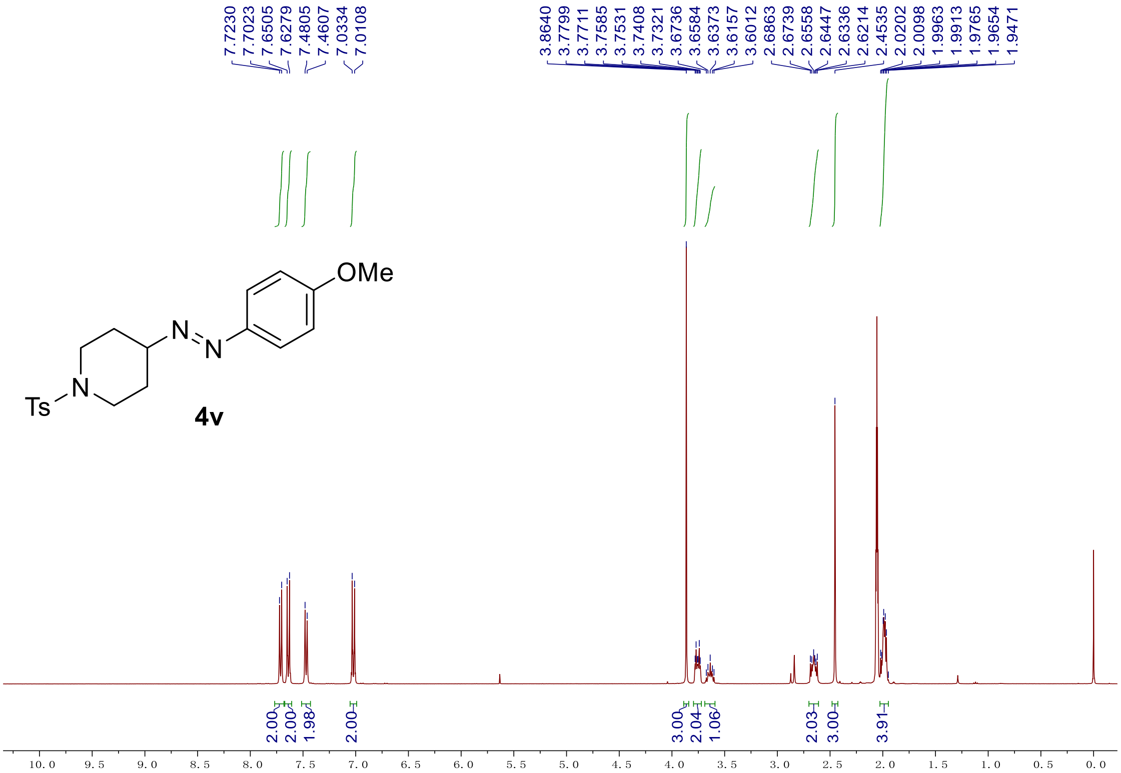

Supplementary Fig. 98. ^{1}H NMR of compound **4v.**

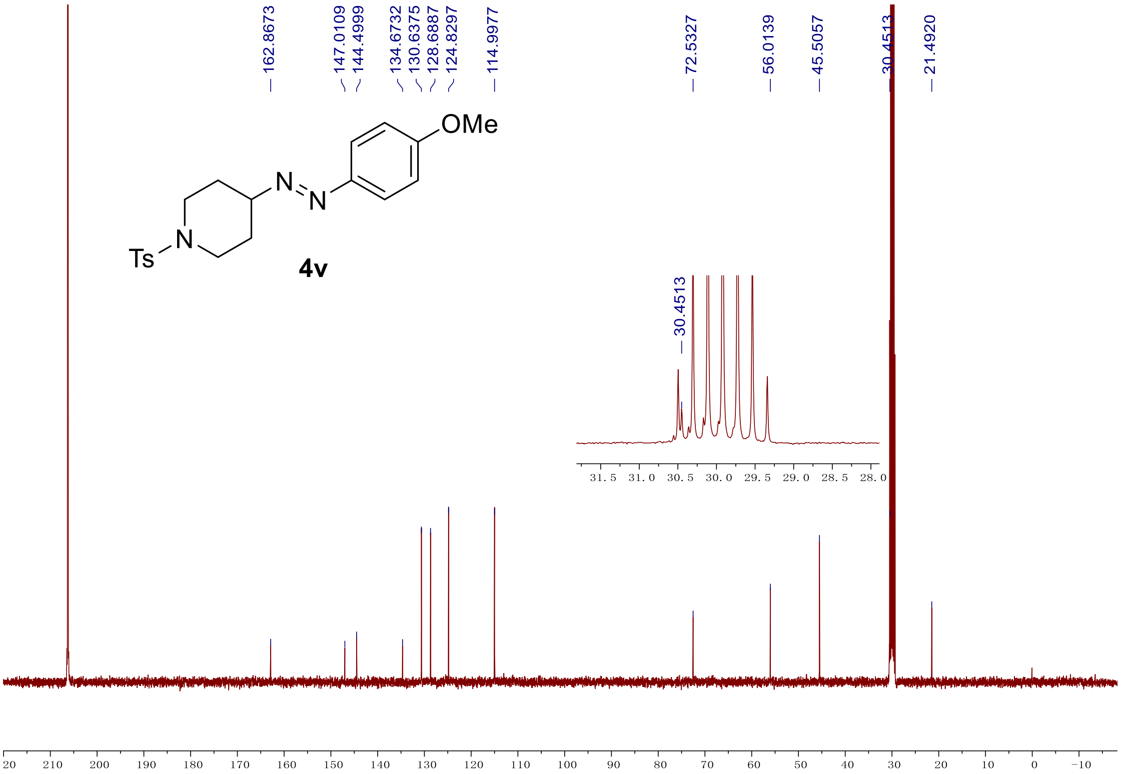

Supplementary Fig. 99. ^{13}C NMR of compound **4v.**

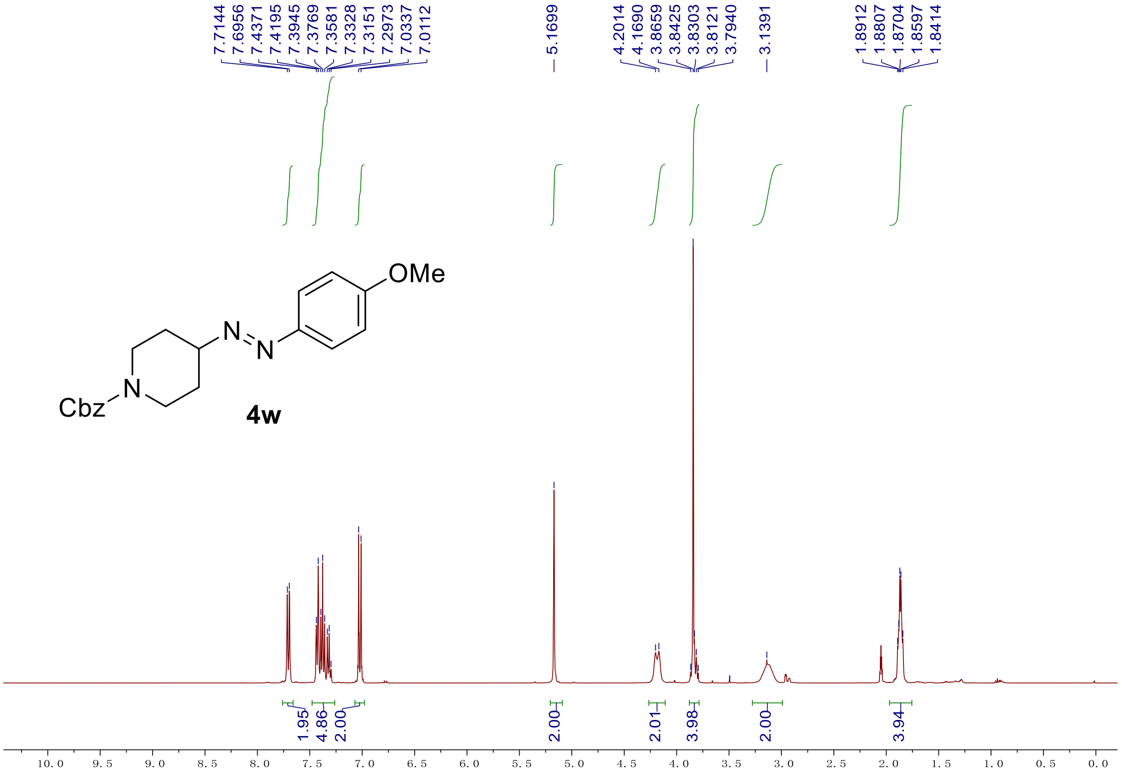

Supplementary Fig. 100. ^{1}H NMR of compound **4w.**

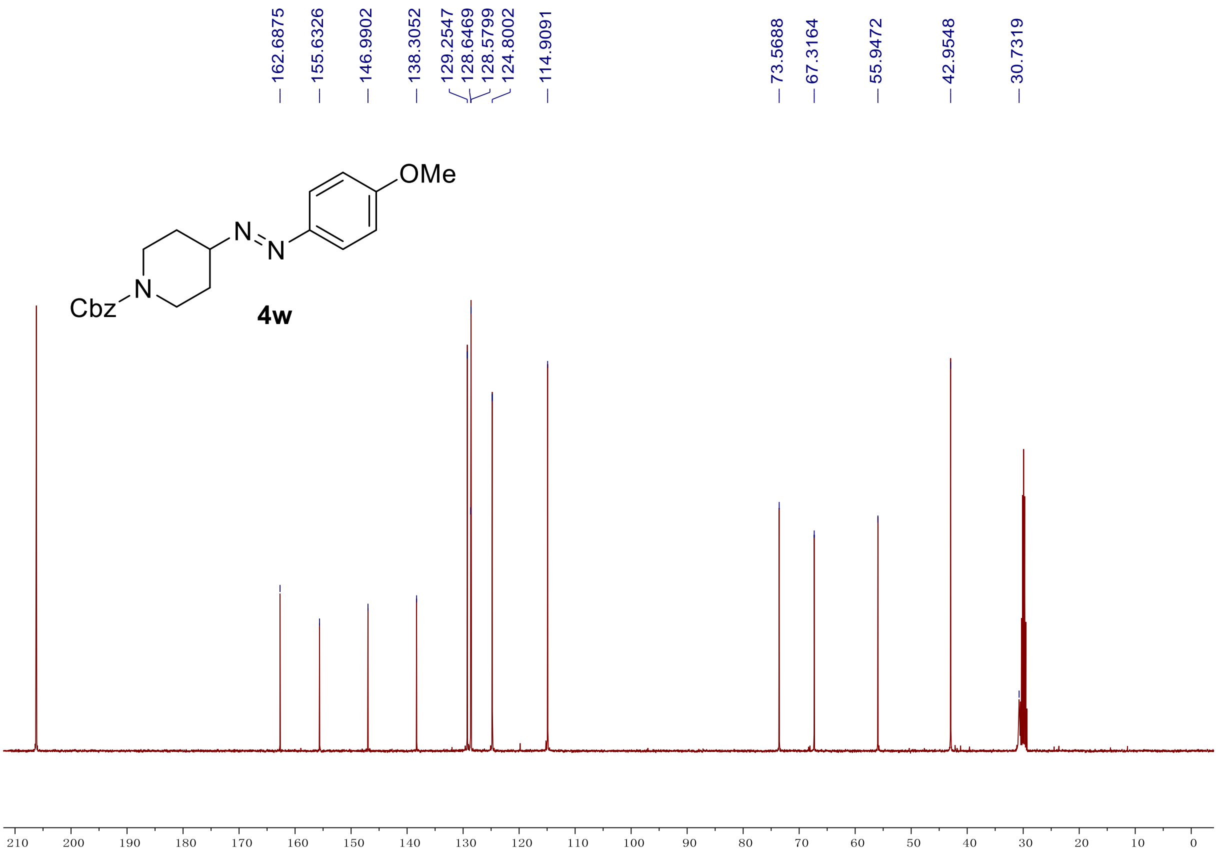

Supplementary Fig. 101. ^{13}C NMR of compound **4w.**

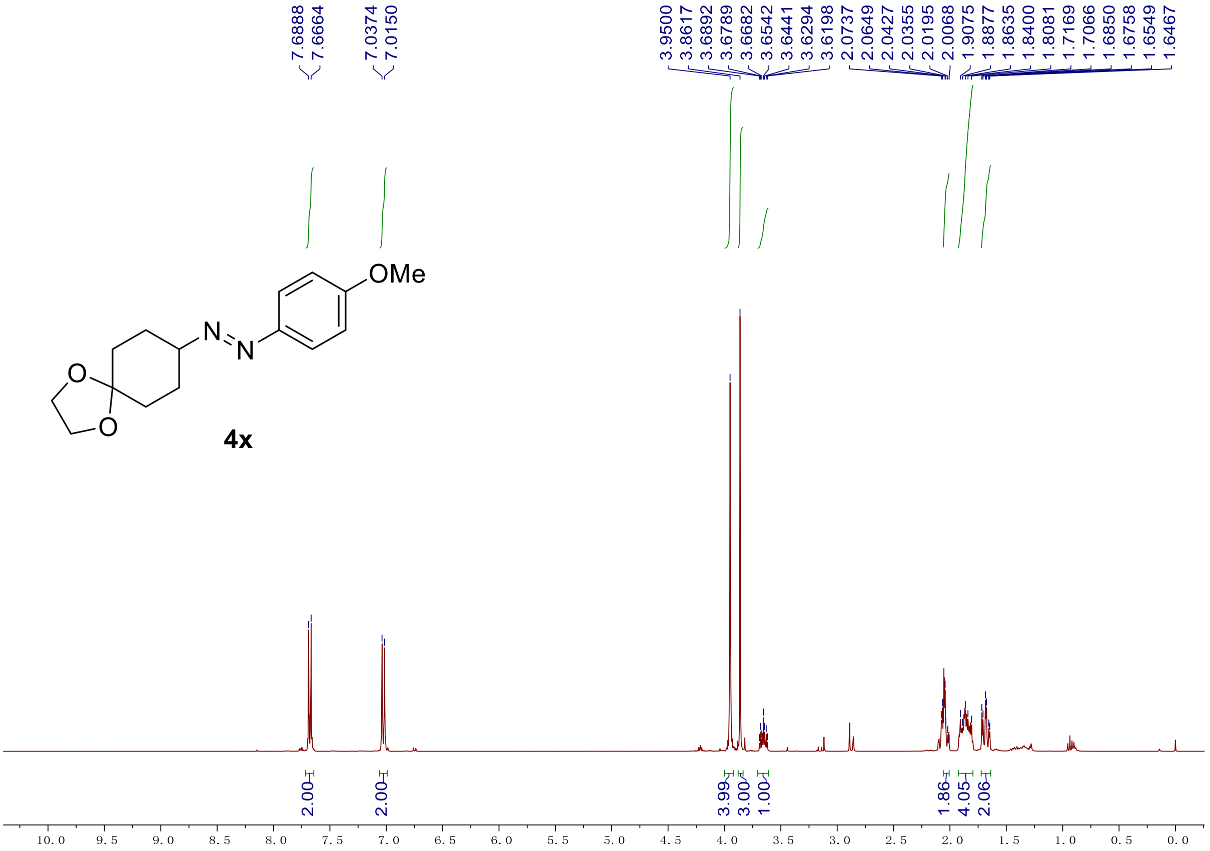

Supplementary Fig. 102. ^{1}H NMR of compound **4x.**

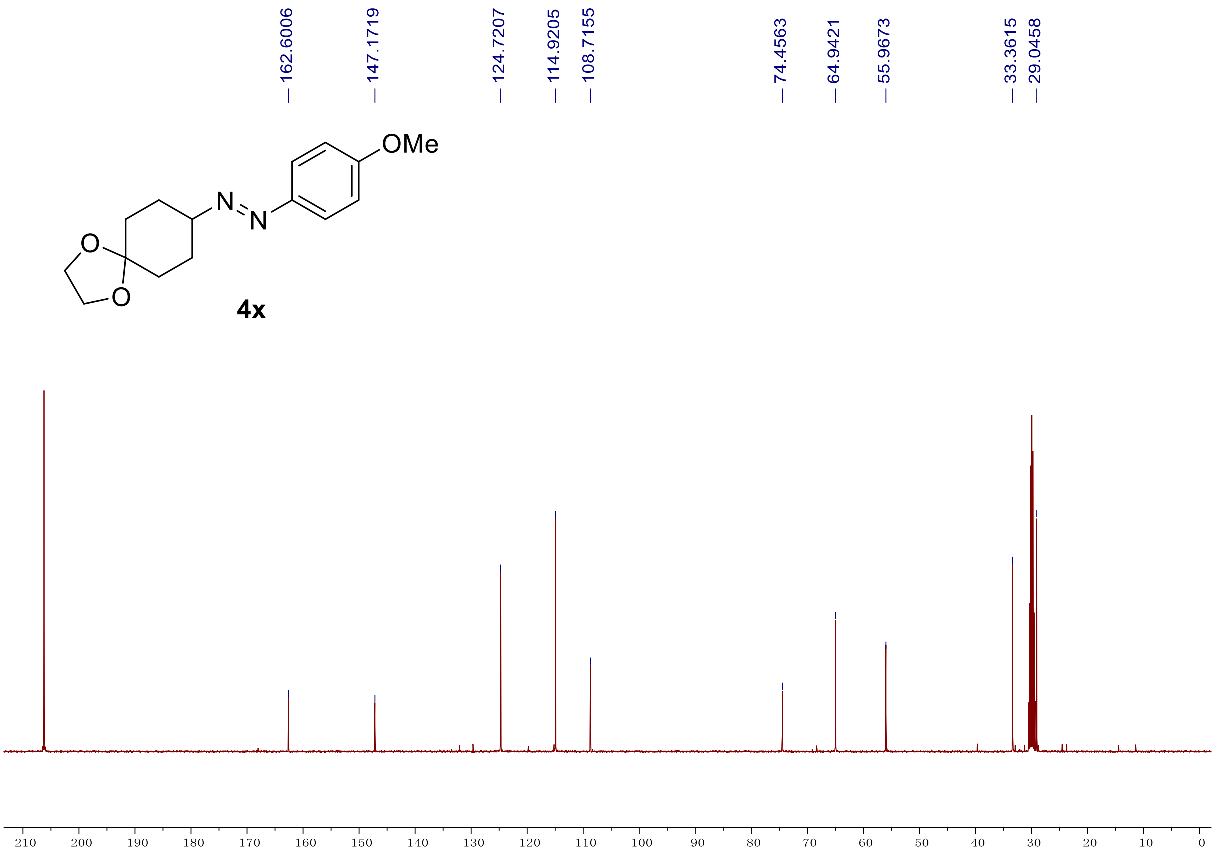

Supplementary Fig. 103. ^{13}C NMR of compound **4x.**

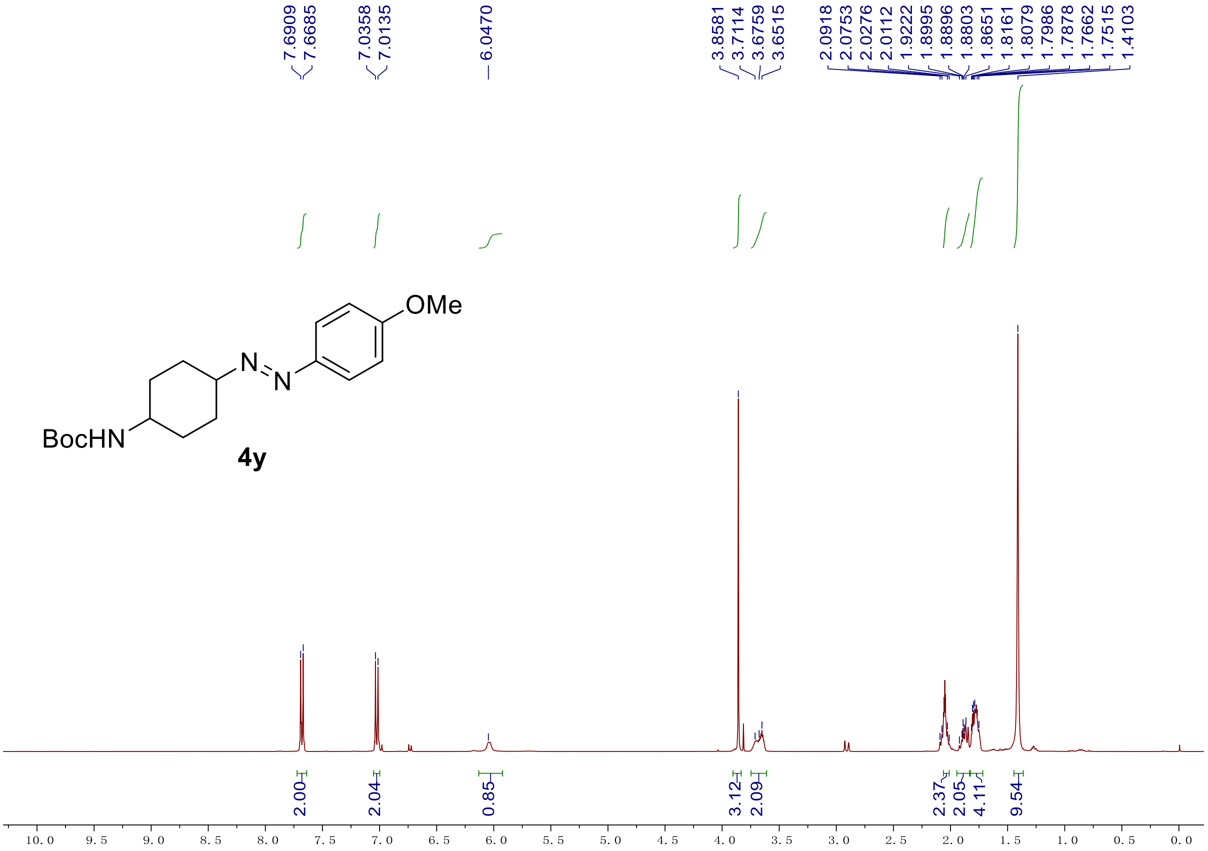

Supplementary Fig. 104. ^{1}H NMR of compound **4y.**

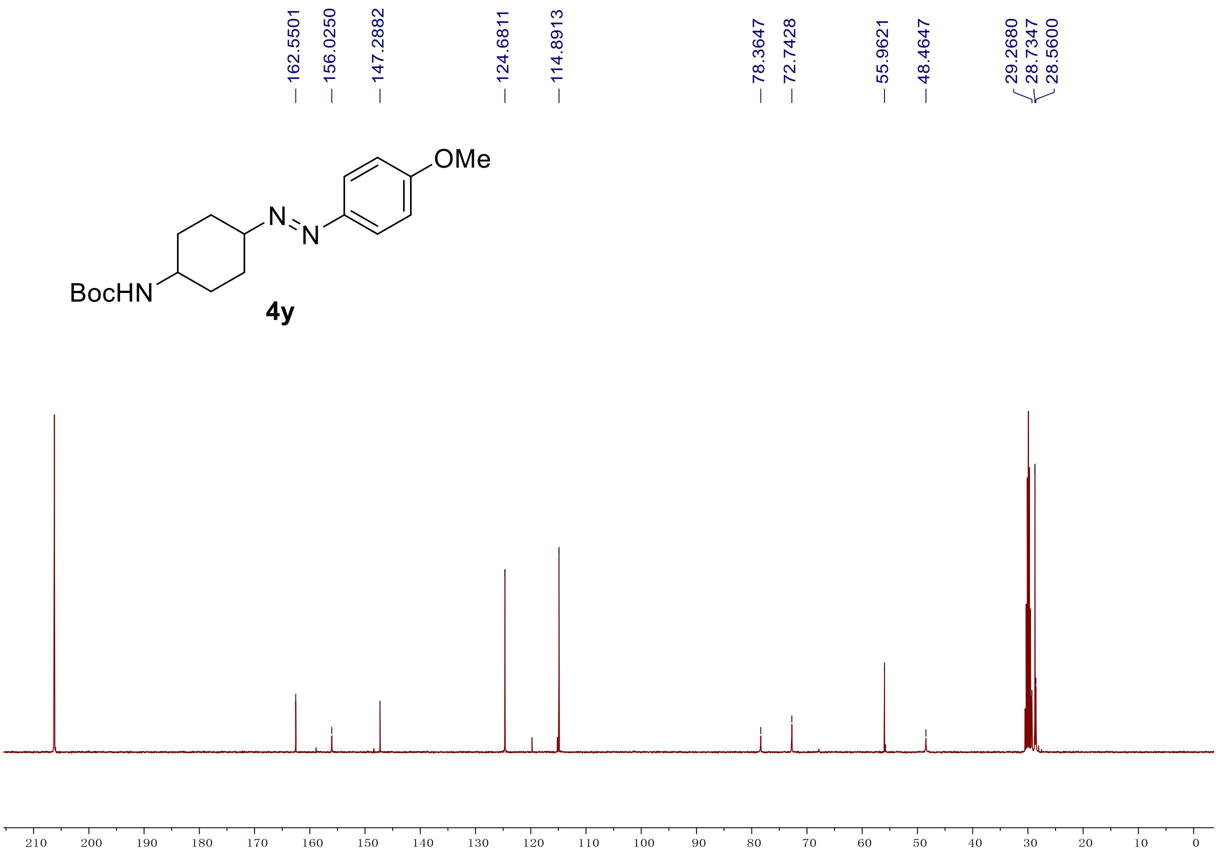

Supplementary Fig. 105. ^{13}C NMR of compound **4y.**

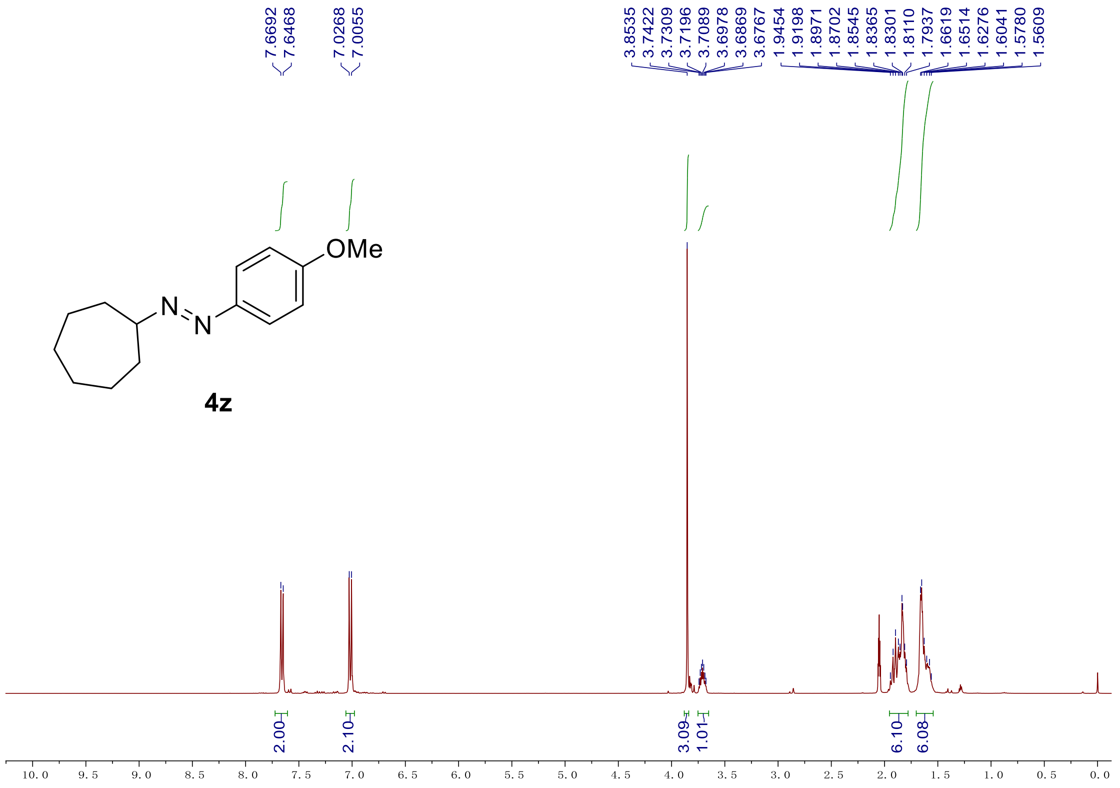

Supplementary Fig. 106. ^{1}H NMR of compound **4z.**

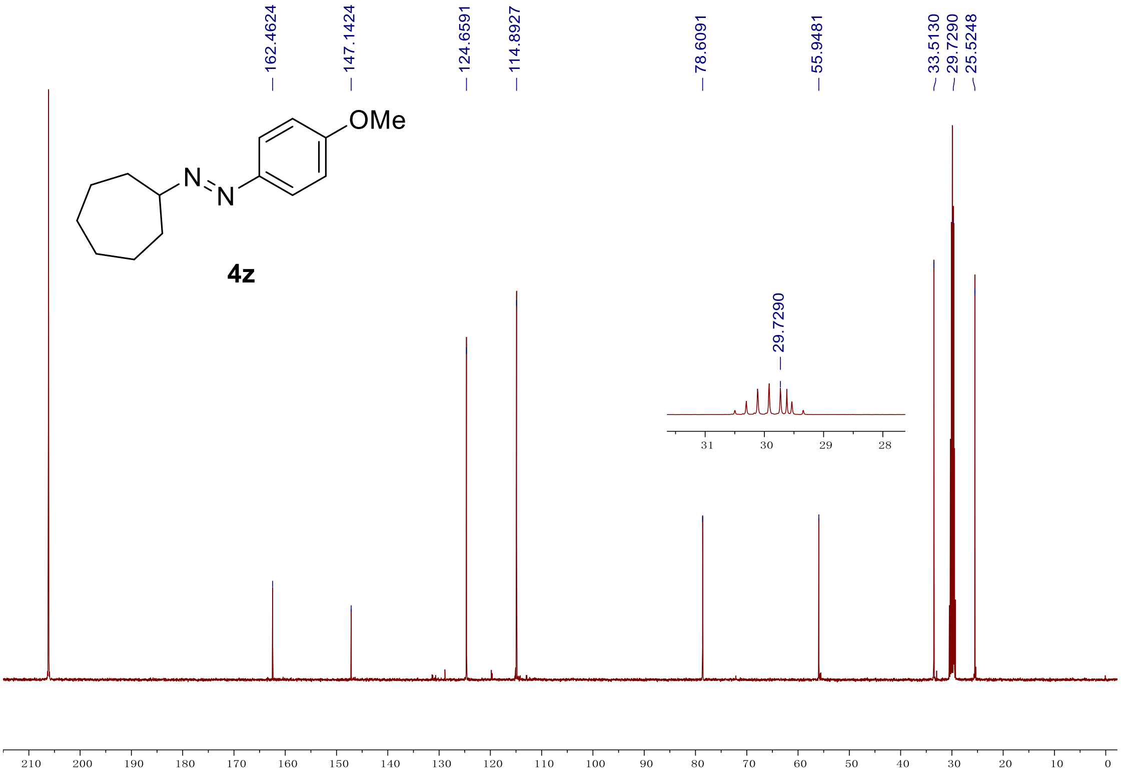

Supplementary Fig. 107. ^{13}C NMR of compound **4z.**

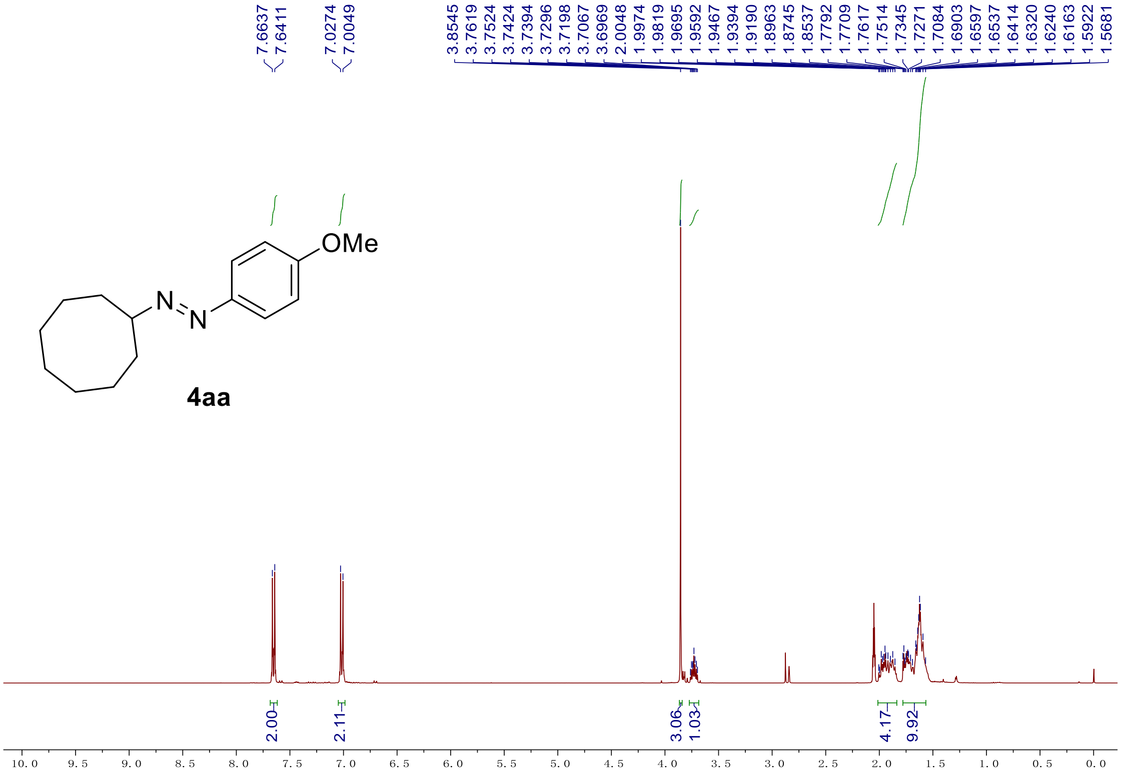

Supplementary Fig. 108. ^{1}H NMR of compound **4aa.**

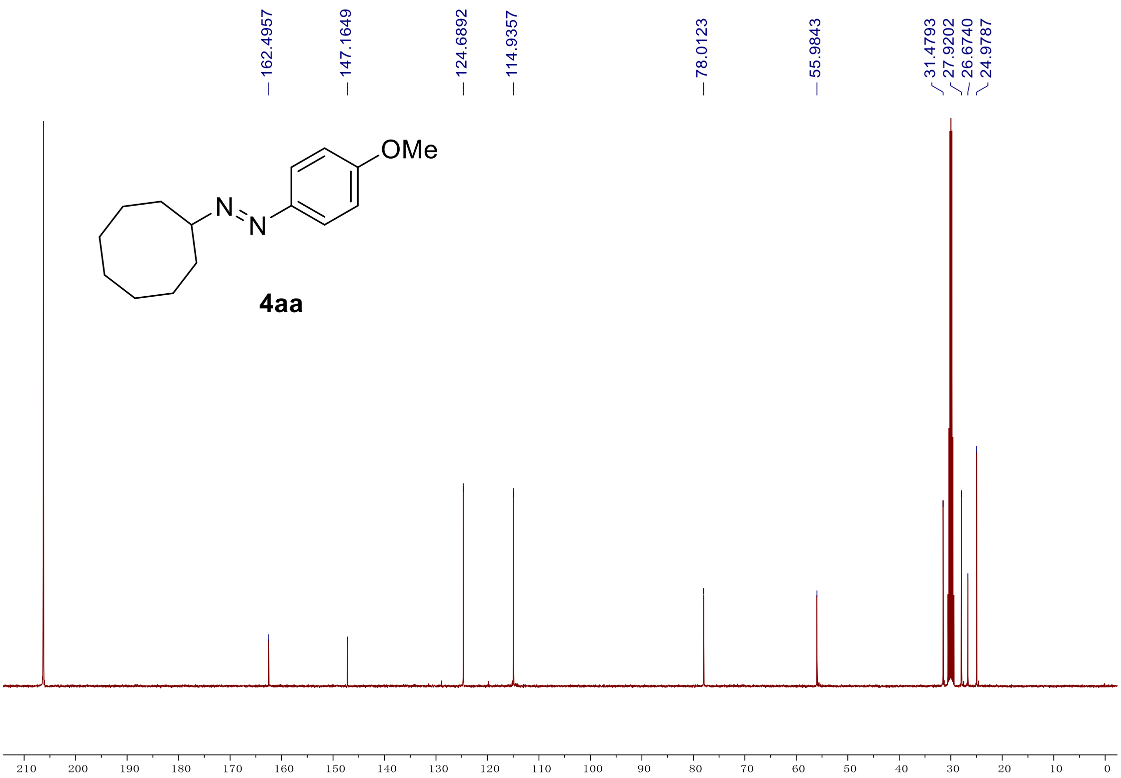

Supplementary Fig. 109. ^{13}C NMR of compound **4aa.**

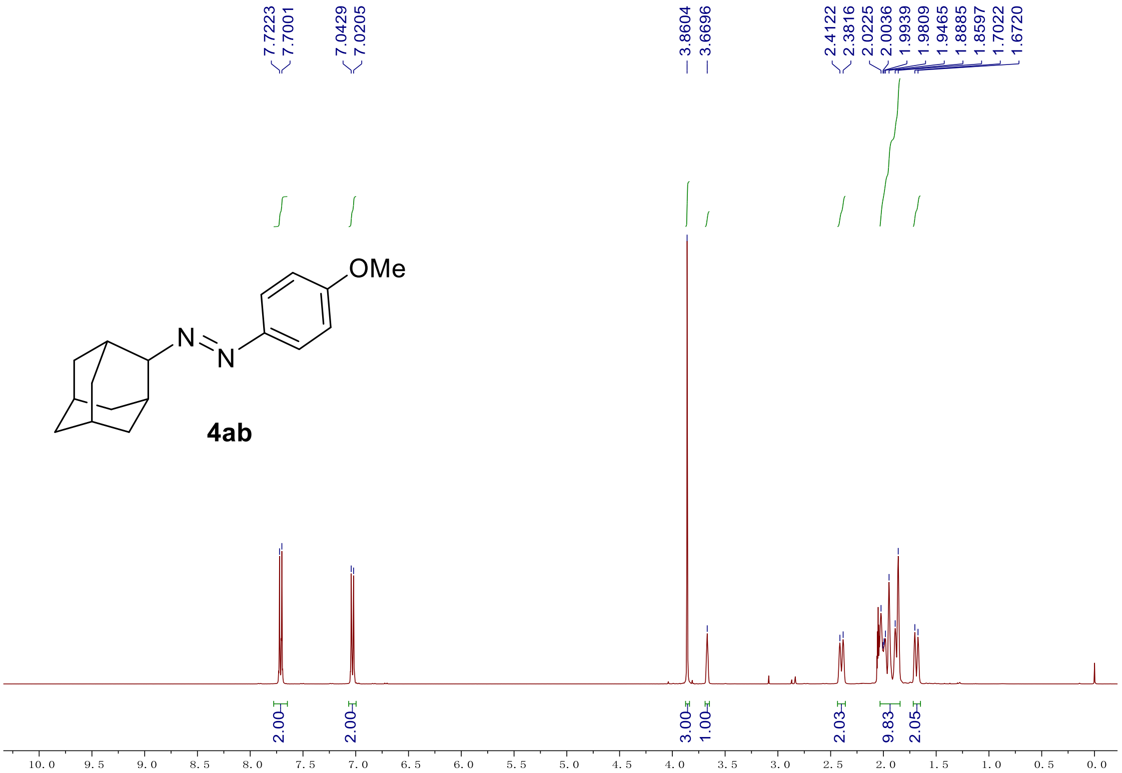

Supplementary Fig. 110. ^{1}H NMR of compound **4ab.**

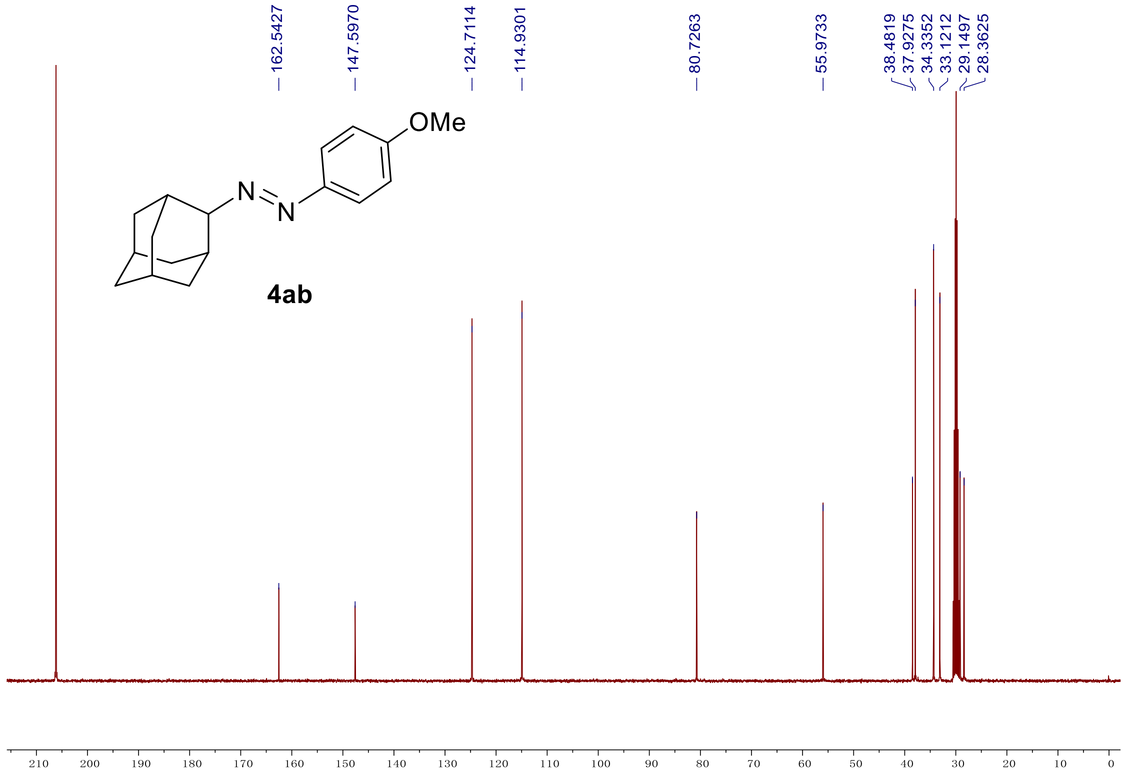

Supplementary Fig. 111. ^{13}C NMR of compound **4ab.**

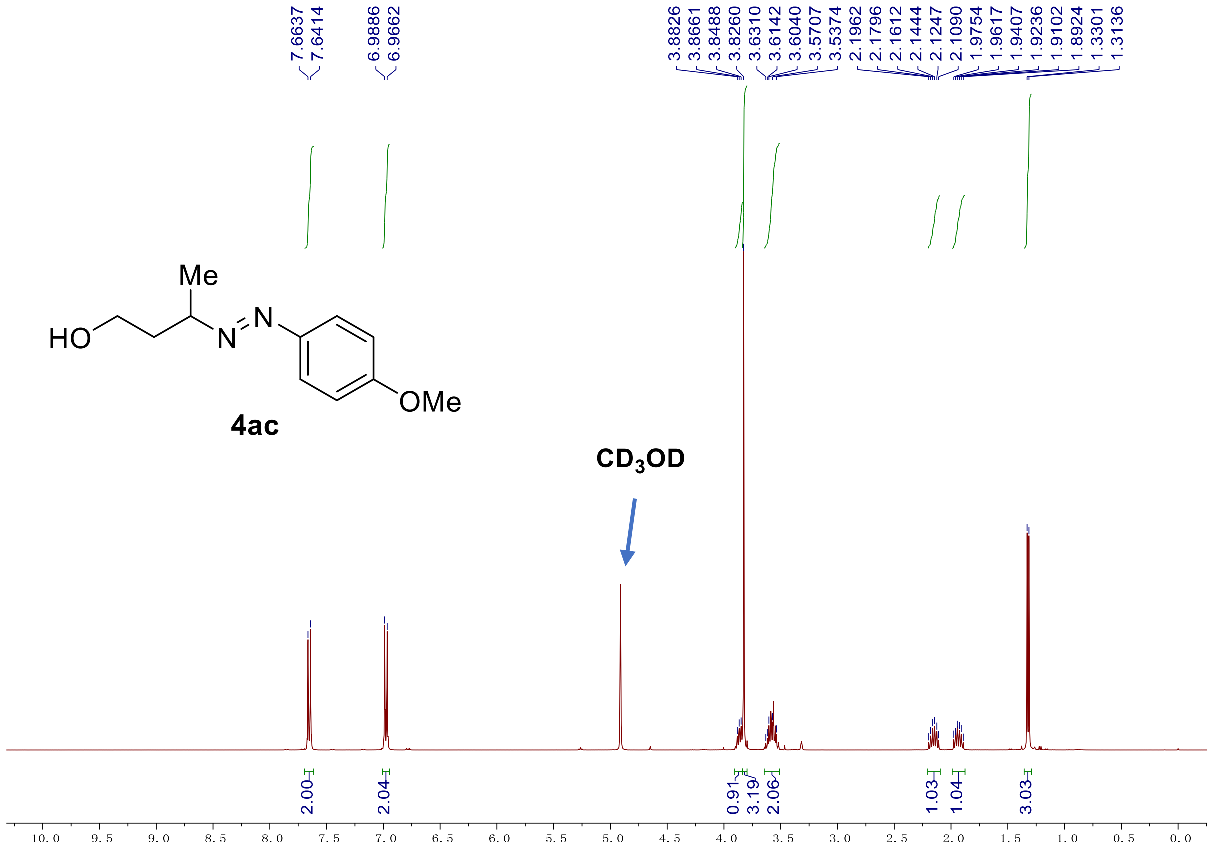

Supplementary Fig. 112. ^{1}H NMR of compound **4ac.**

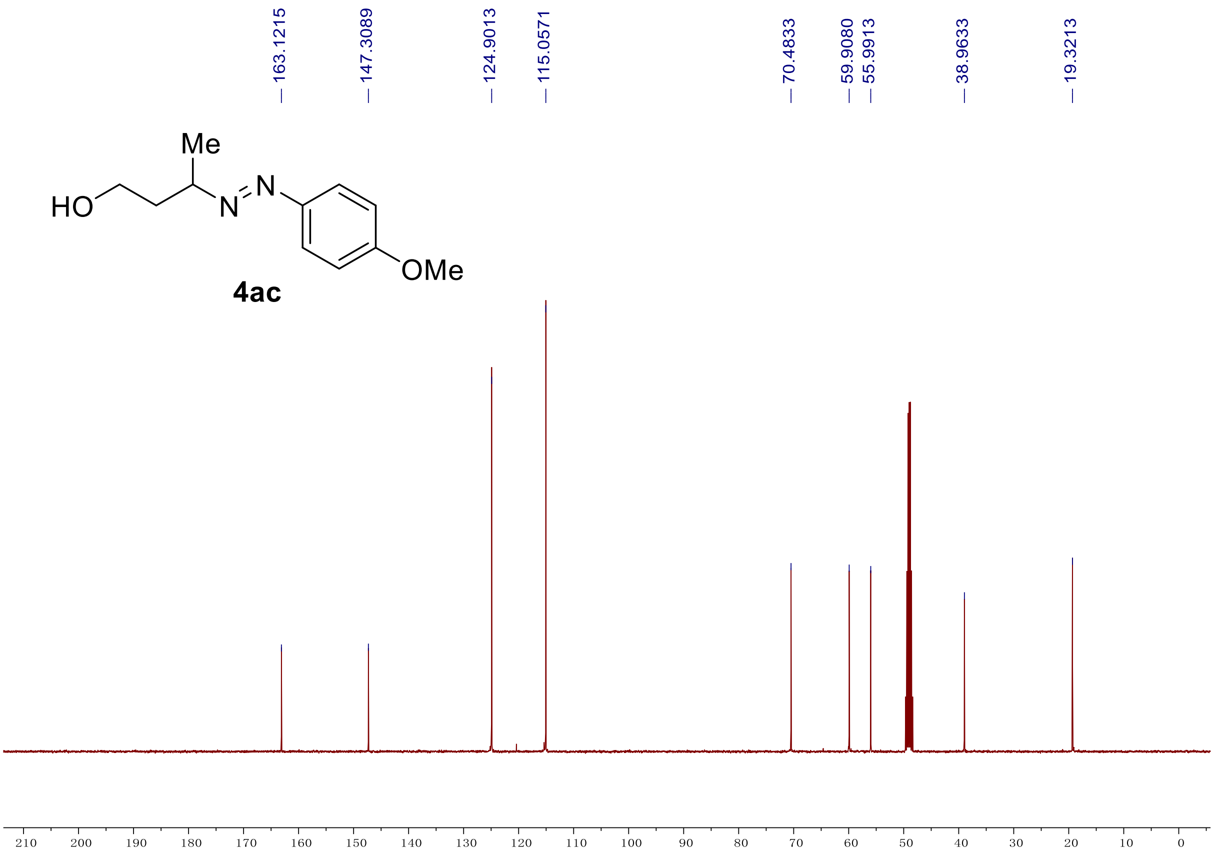

Supplementary Fig. 113. ^{13}C NMR of compound **4ac.**

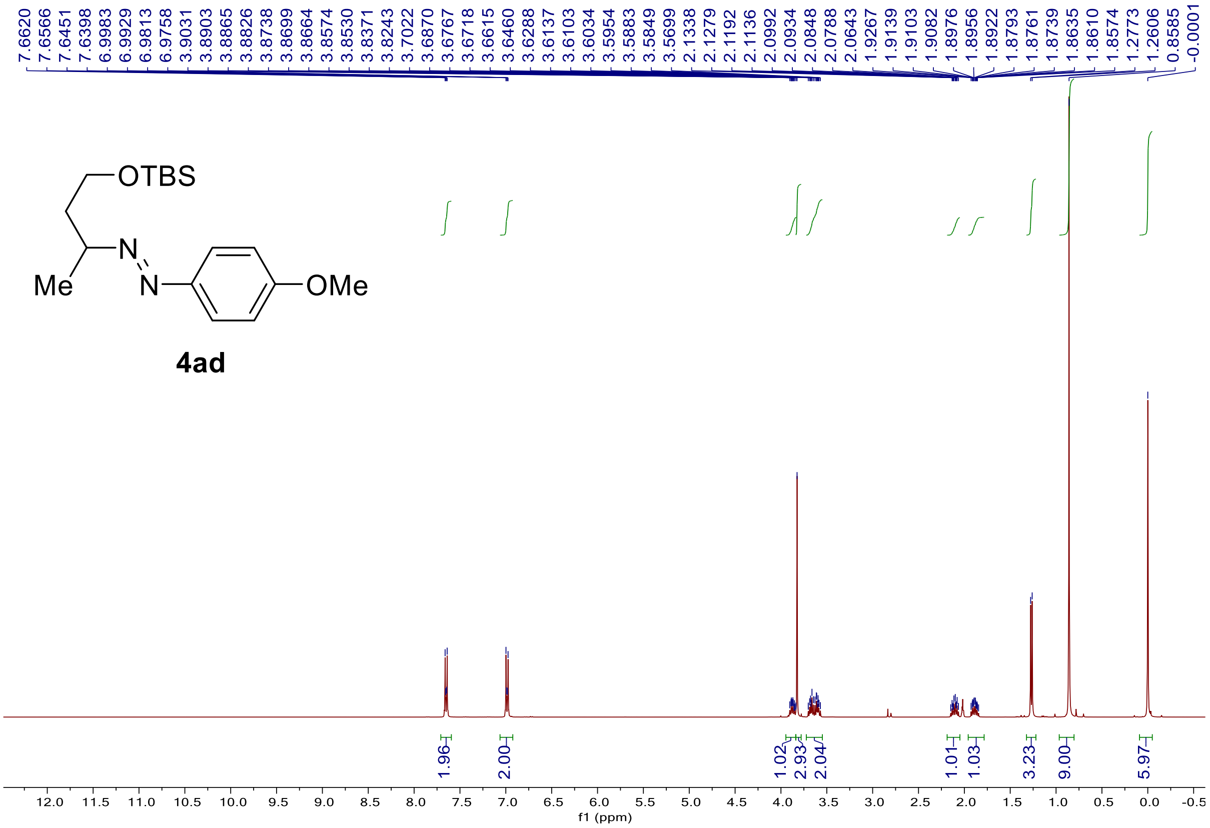

Supplementary Fig. 114. ^{1}H NMR of compound **4ad.**

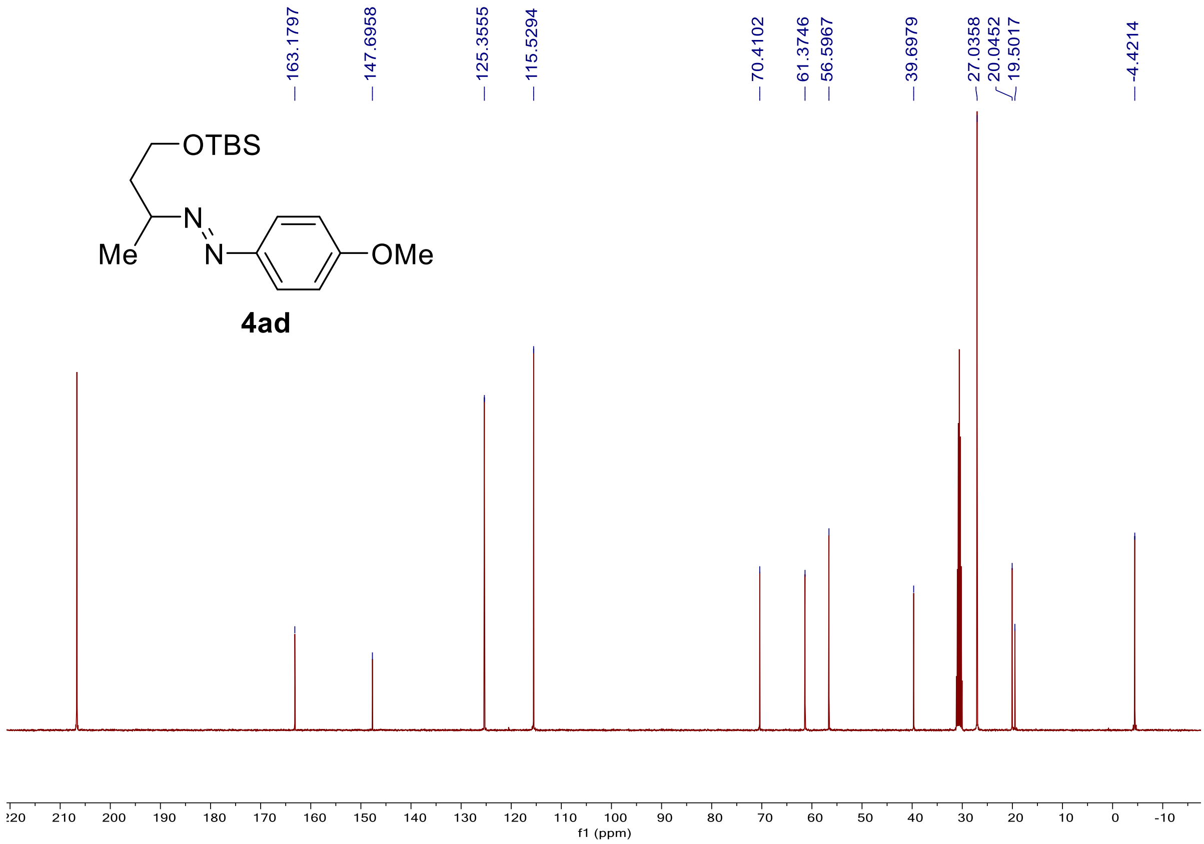

Supplementary Fig. 115. ^{13}C NMR of compound **4ad.**

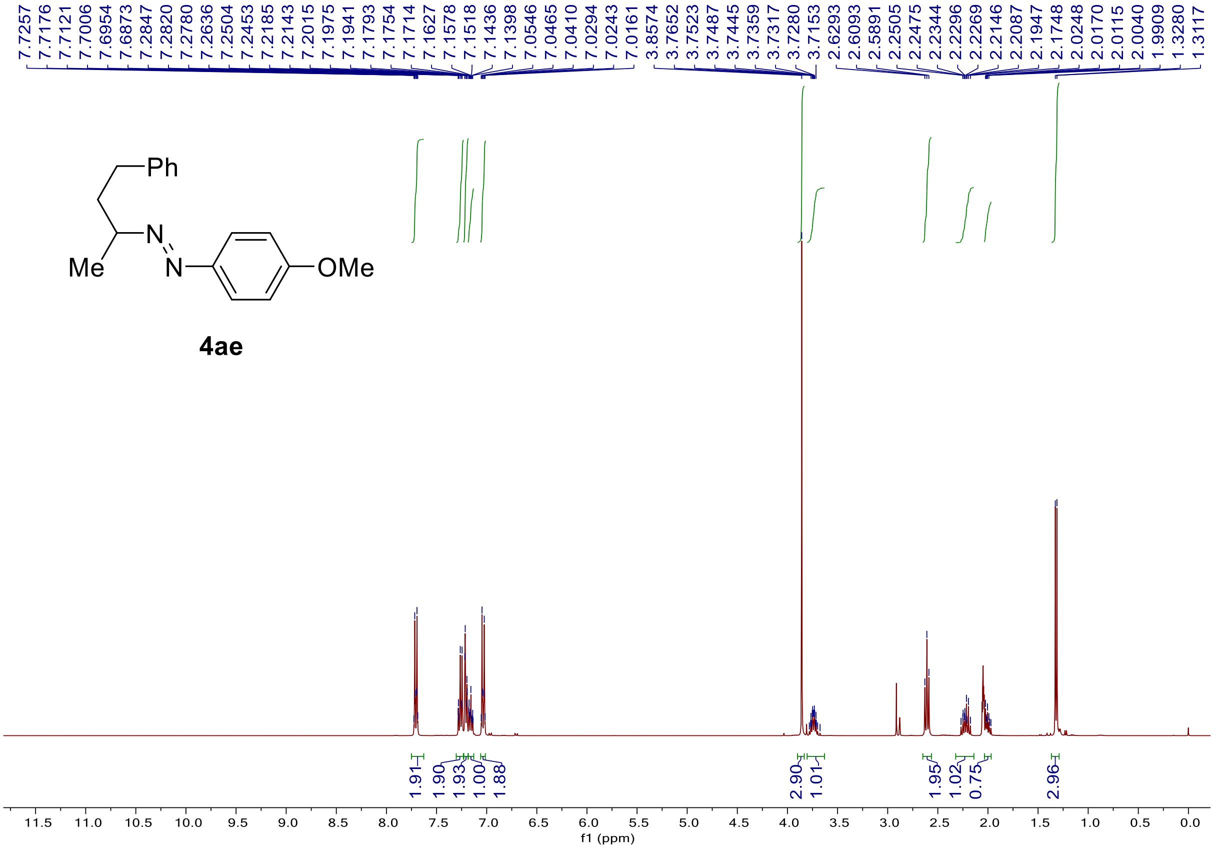

Supplementary Fig. 116. ^{1}H NMR of compound **4ae.**

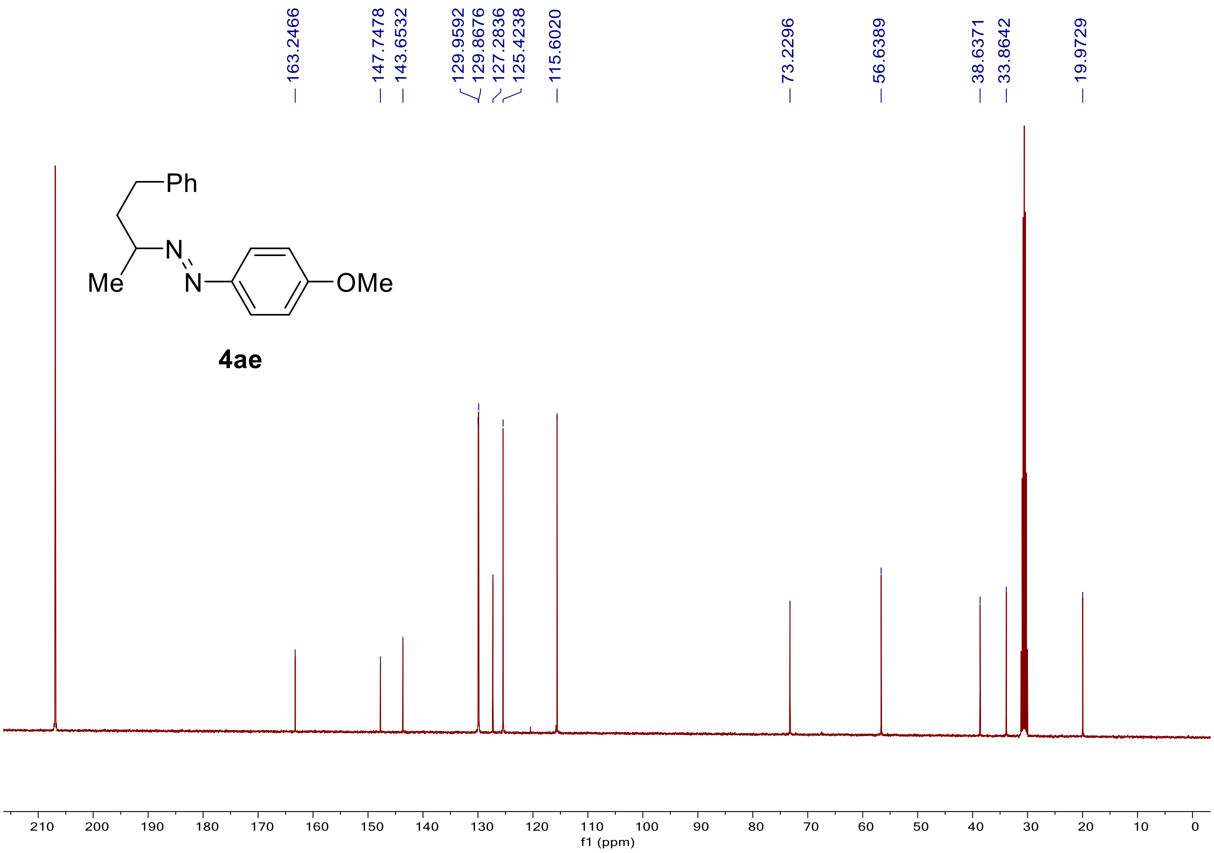

Supplementary Fig. 116. ^{13}C NMR of compound **4ae.**

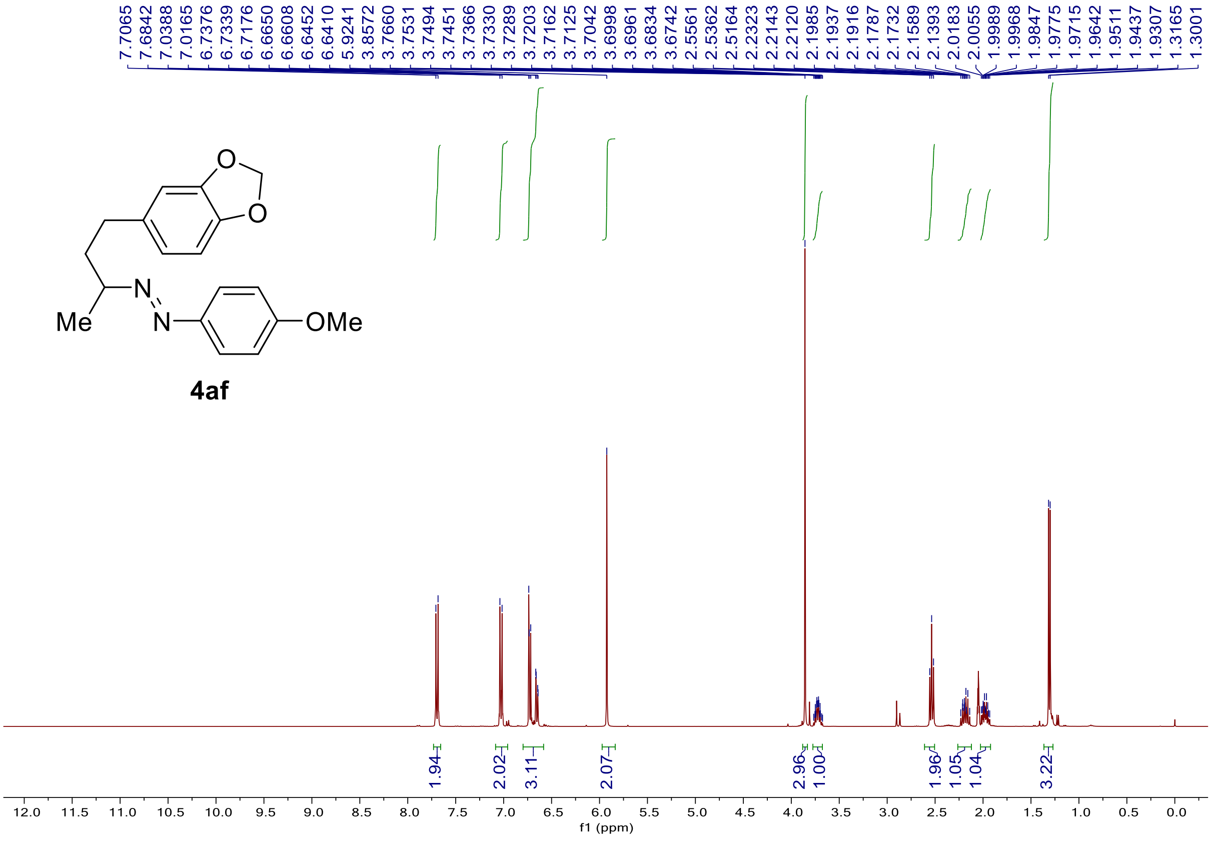

Supplementary Fig. 117. ^{1}H NMR of compound **4af.**

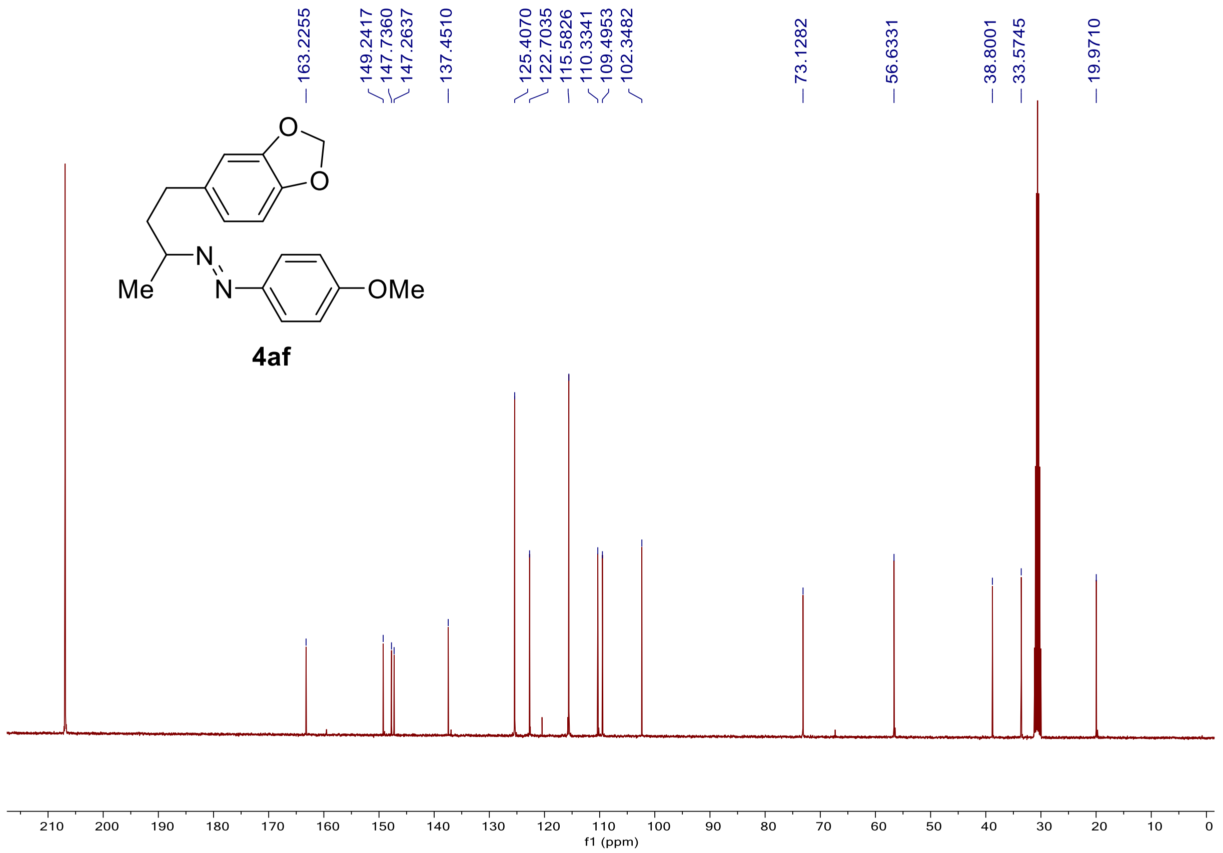

Supplementary Fig. 118. ^{13}C NMR of compound **4af.**

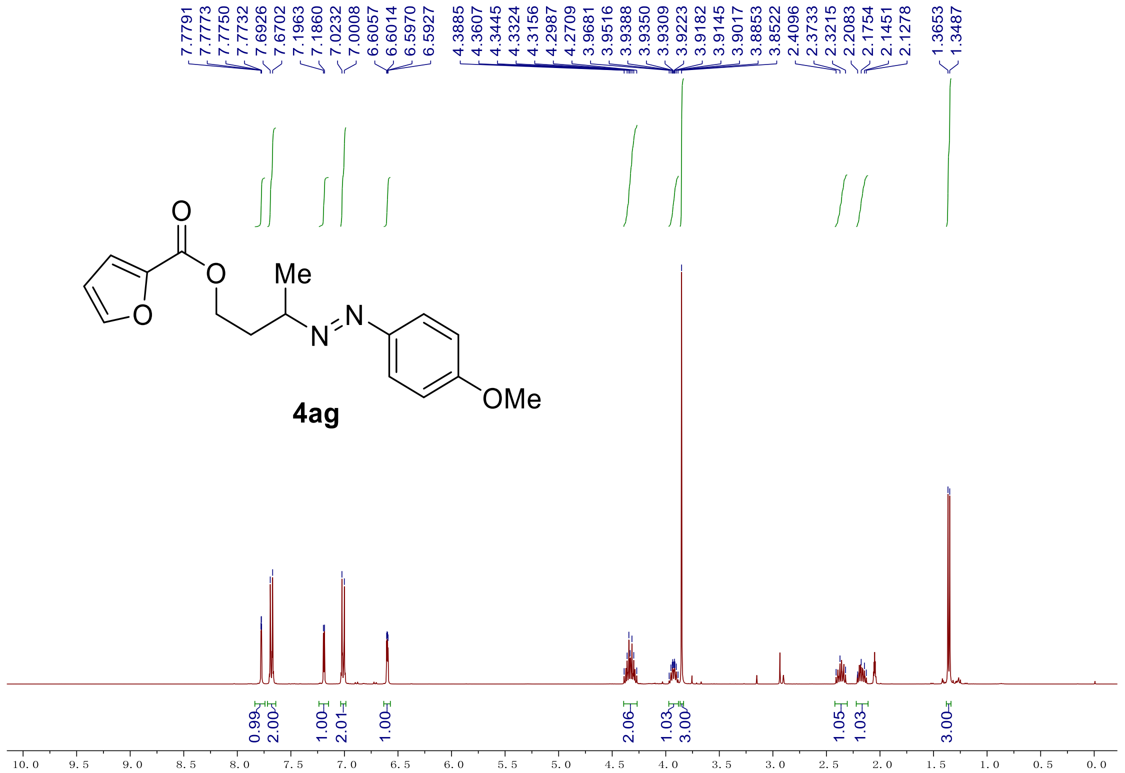

Supplementary Fig. 119. ^{1}H NMR of compound **4ag.**

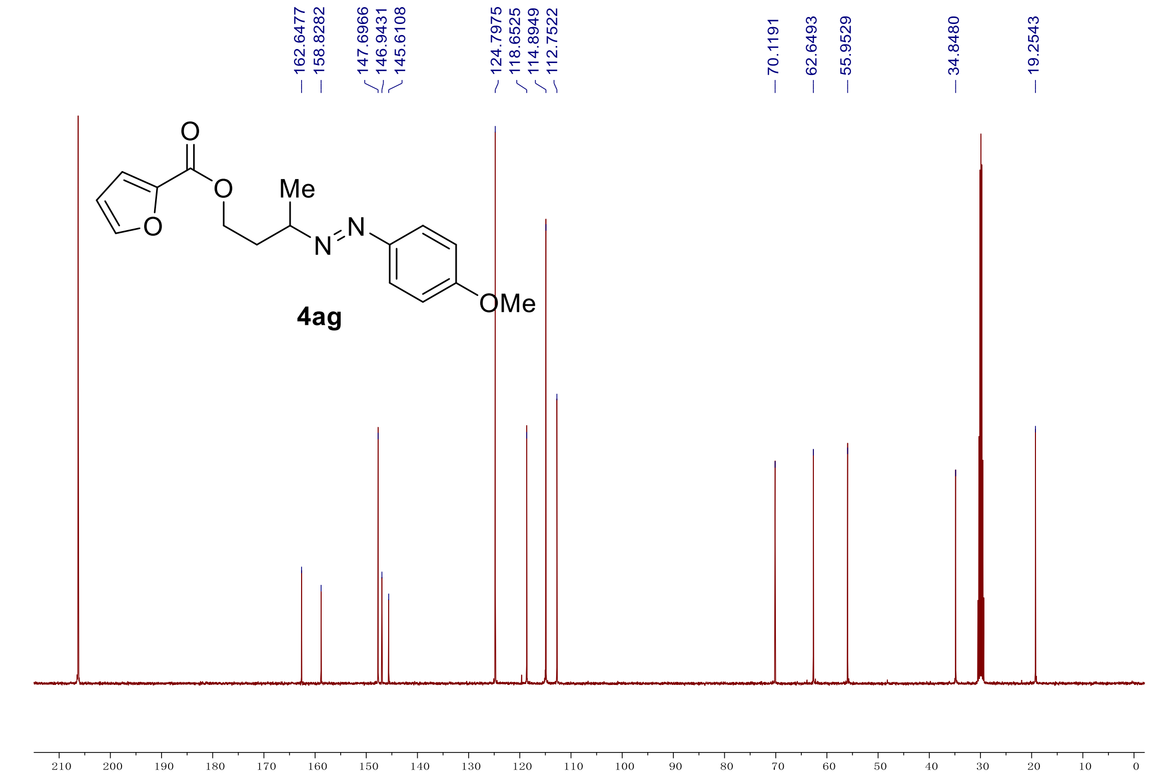

Supplementary Fig. 120. ^{13}C NMR of compound **4ag.**

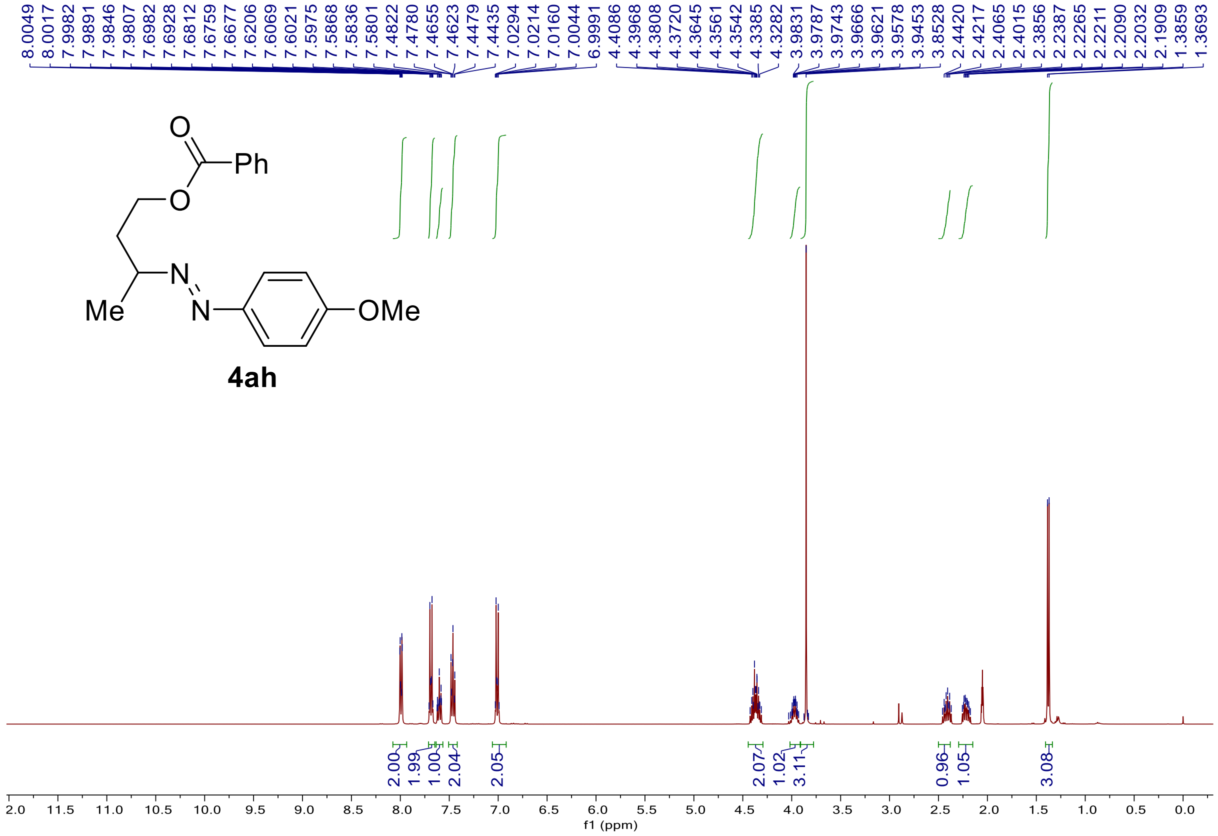

Supplementary Fig. 121. ^{1}H NMR of compound **4ah.**

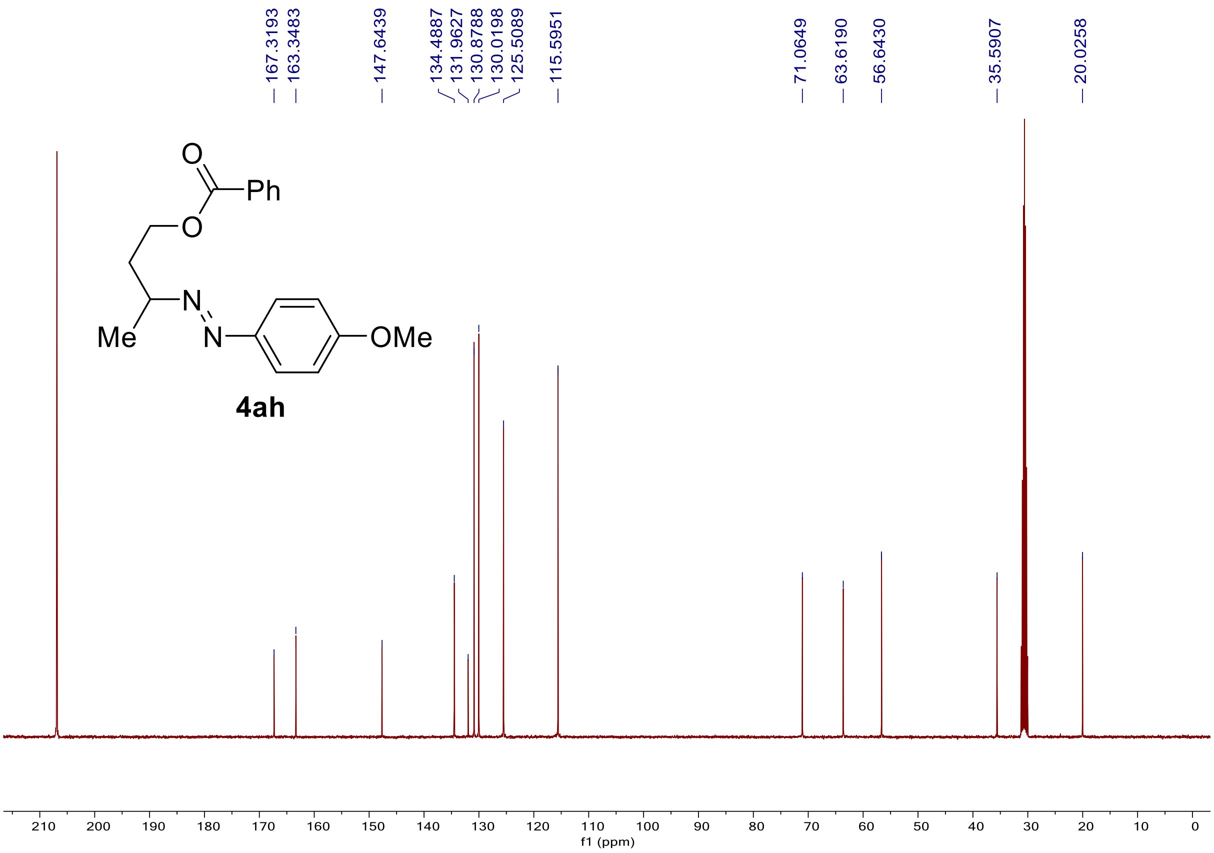

Supplementary Fig. 122. ^{13}C NMR of compound **4ah.**

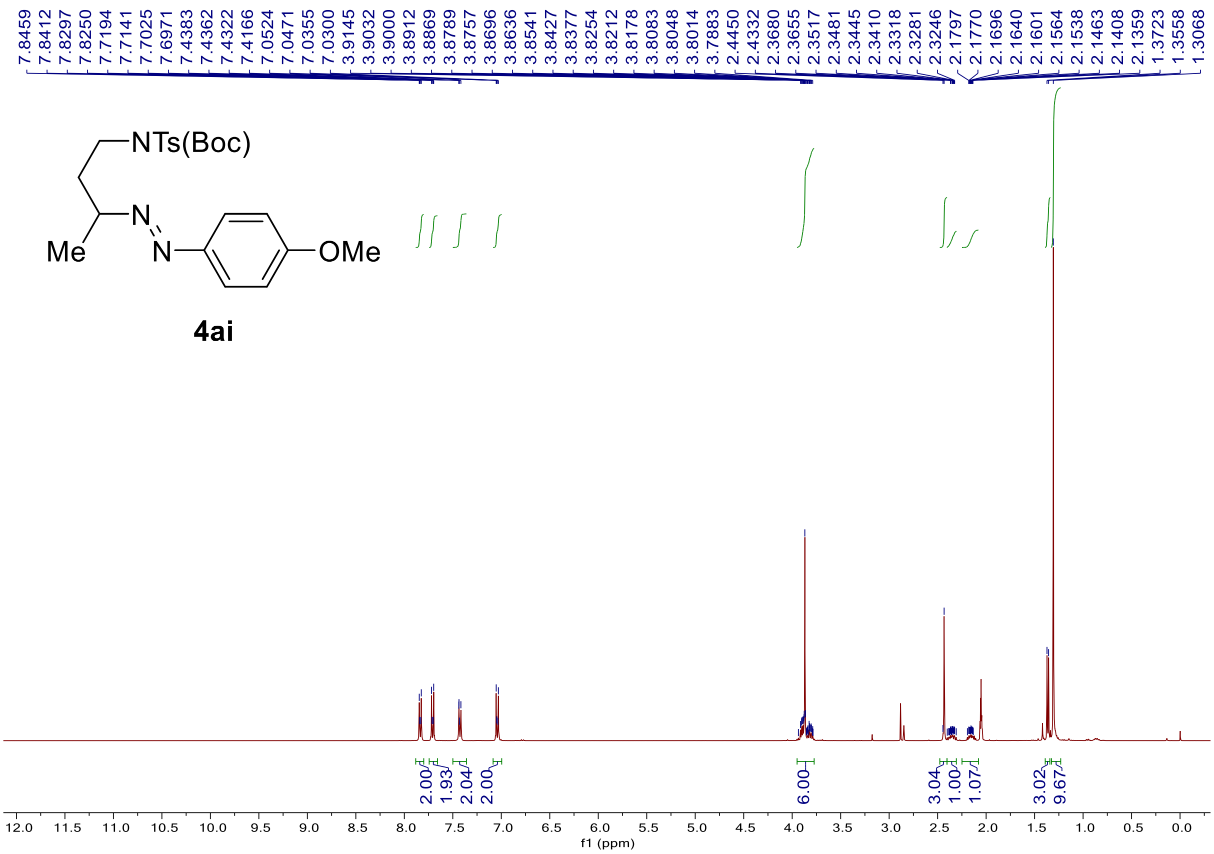

Supplementary Fig. 124. ^{1}H NMR of compound **4ai.**

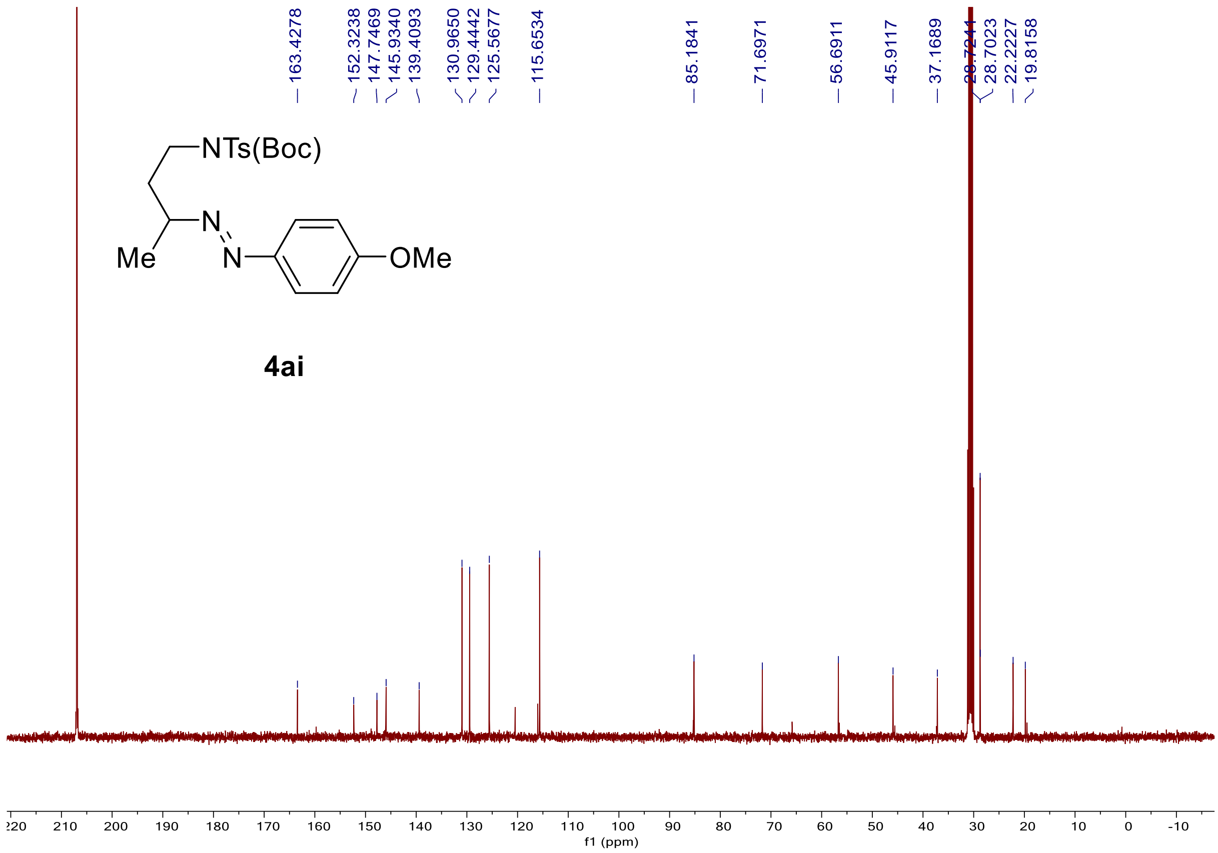

Supplementary Fig. 123. ^{13}C NMR of compound **4ai.**

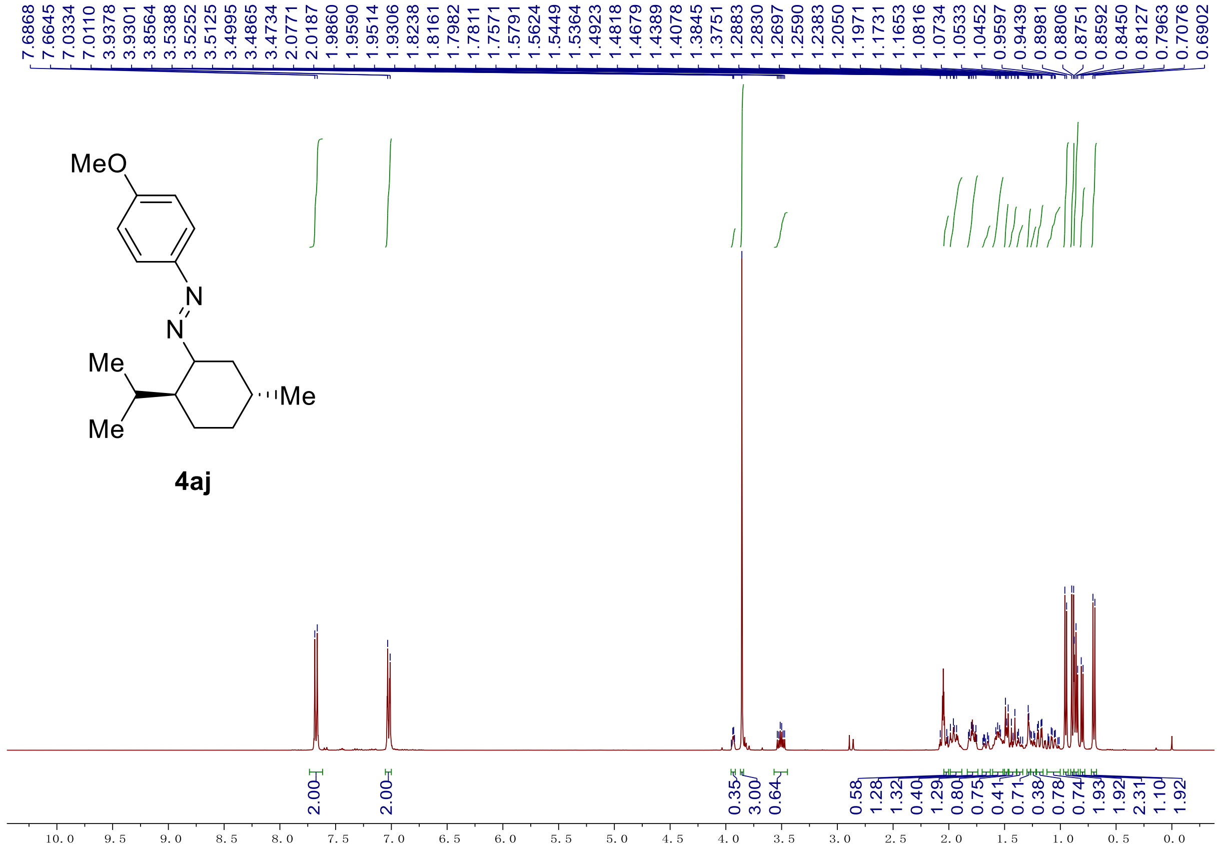

Supplementary Fig. 124. ^{1}H NMR of compound **4aj.**

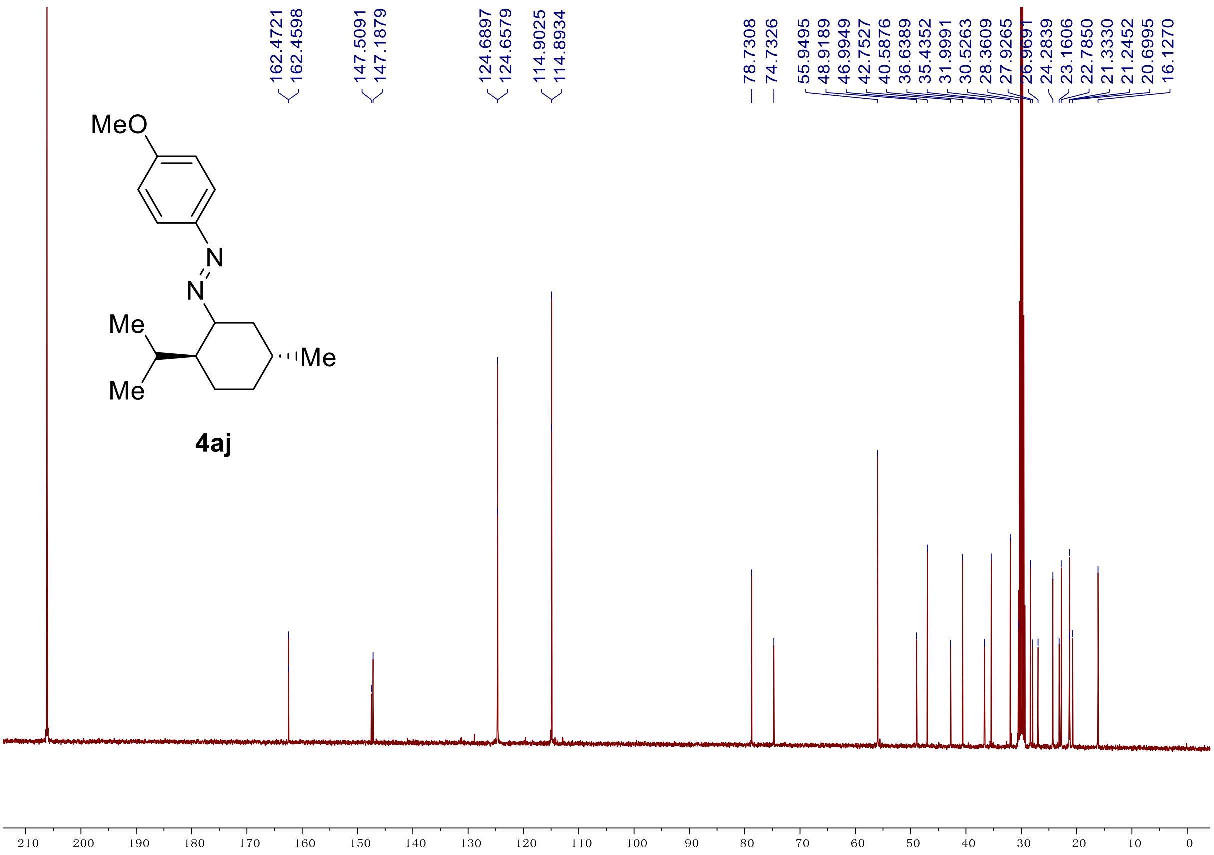

Supplementary Fig. 125. ^{13}C NMR of compound **4aj.**

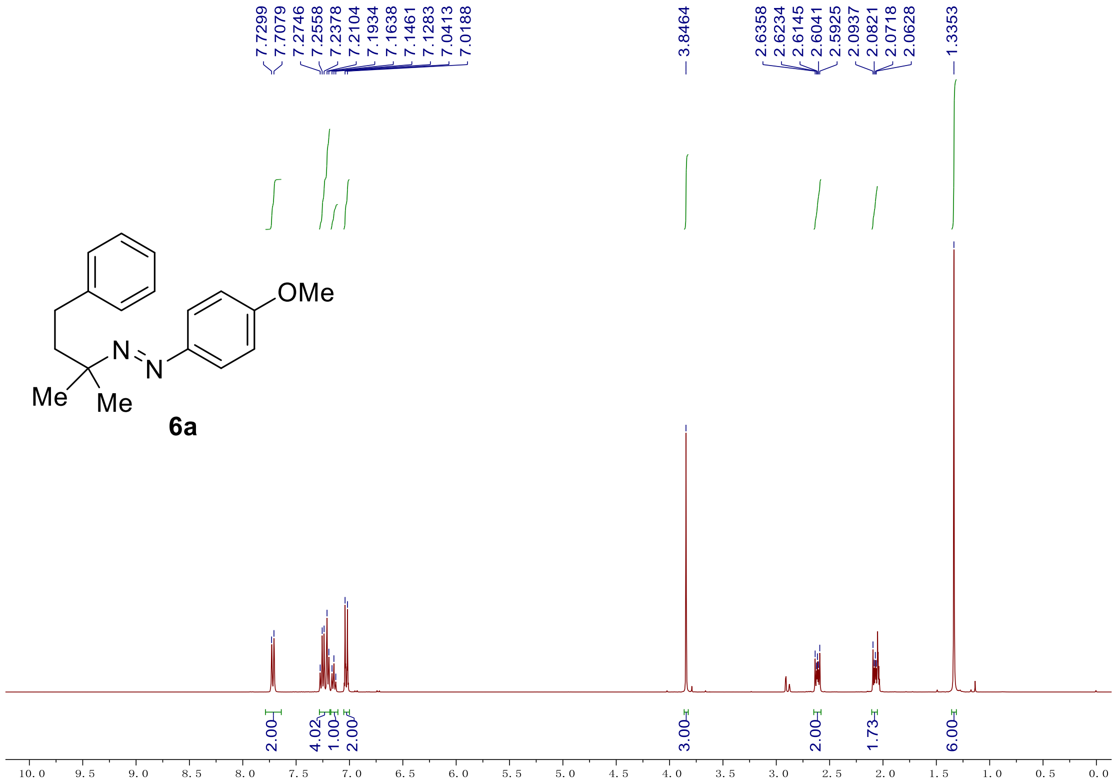

Supplementary Fig. 126. ^{1}H NMR of compound **6a.**

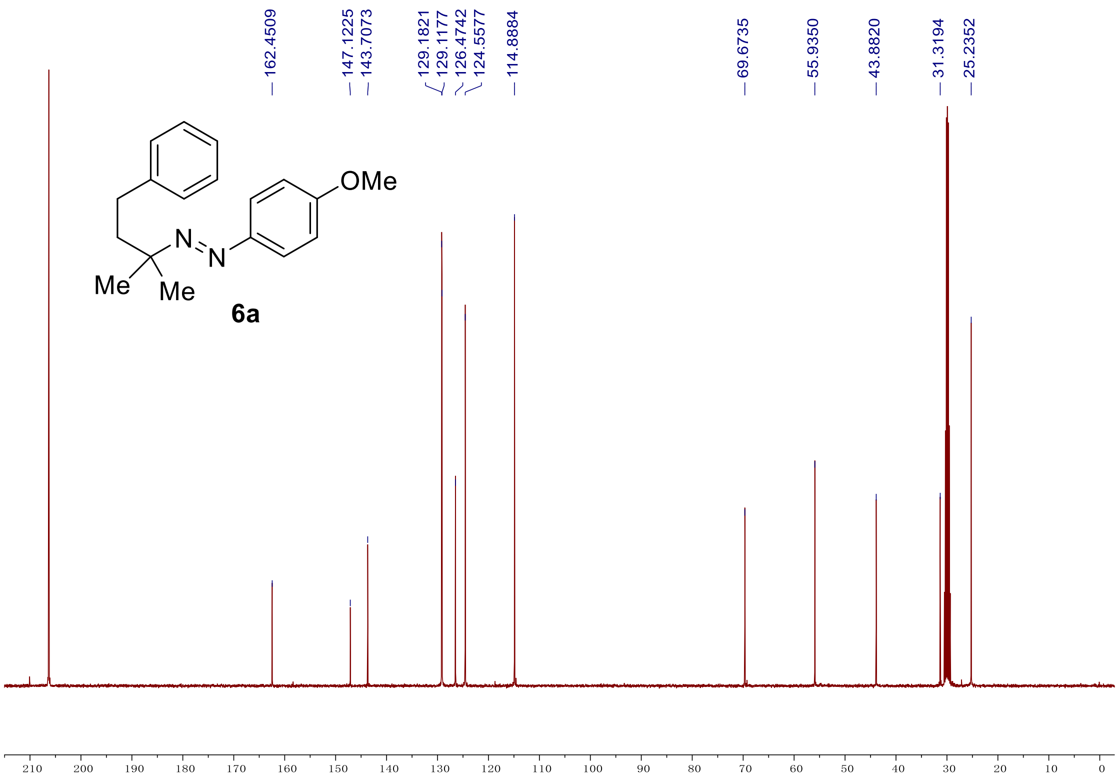

Supplementary Fig. 127. ^{13}C NMR of compound **6a.**

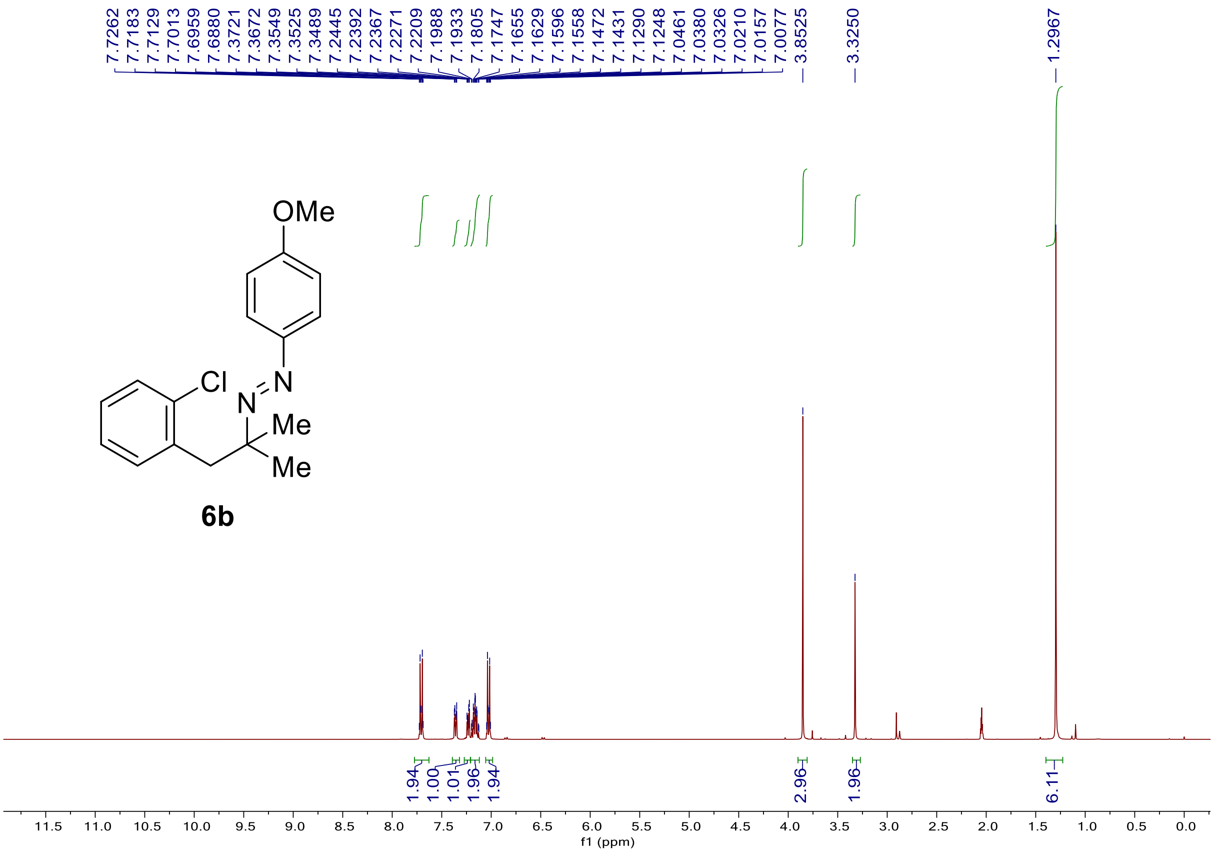

Supplementary Fig. 128. ^{1}H NMR of compound **6b.**

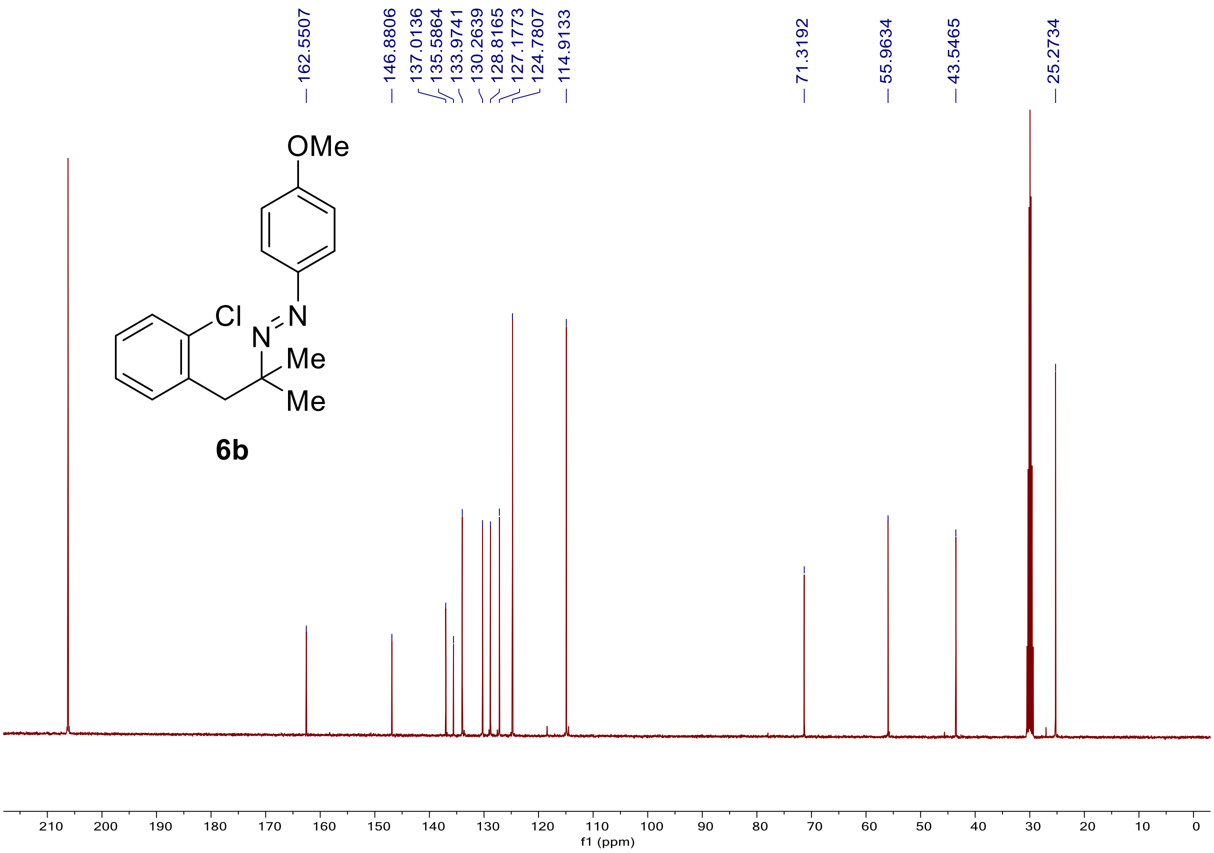

Supplementary Fig. 129. ^{13}C NMR of compound **6b.**

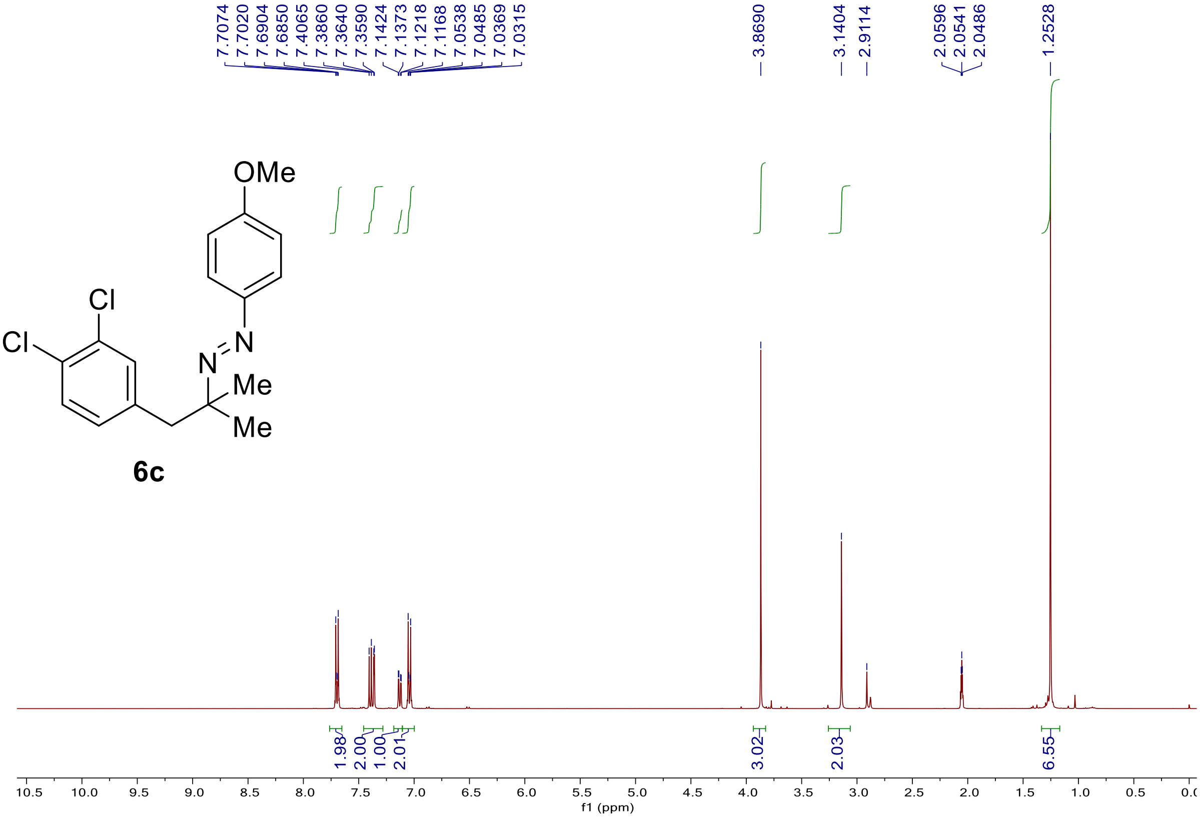

Supplementary Fig. 132. ^{1}H NMR of compound **6c.**

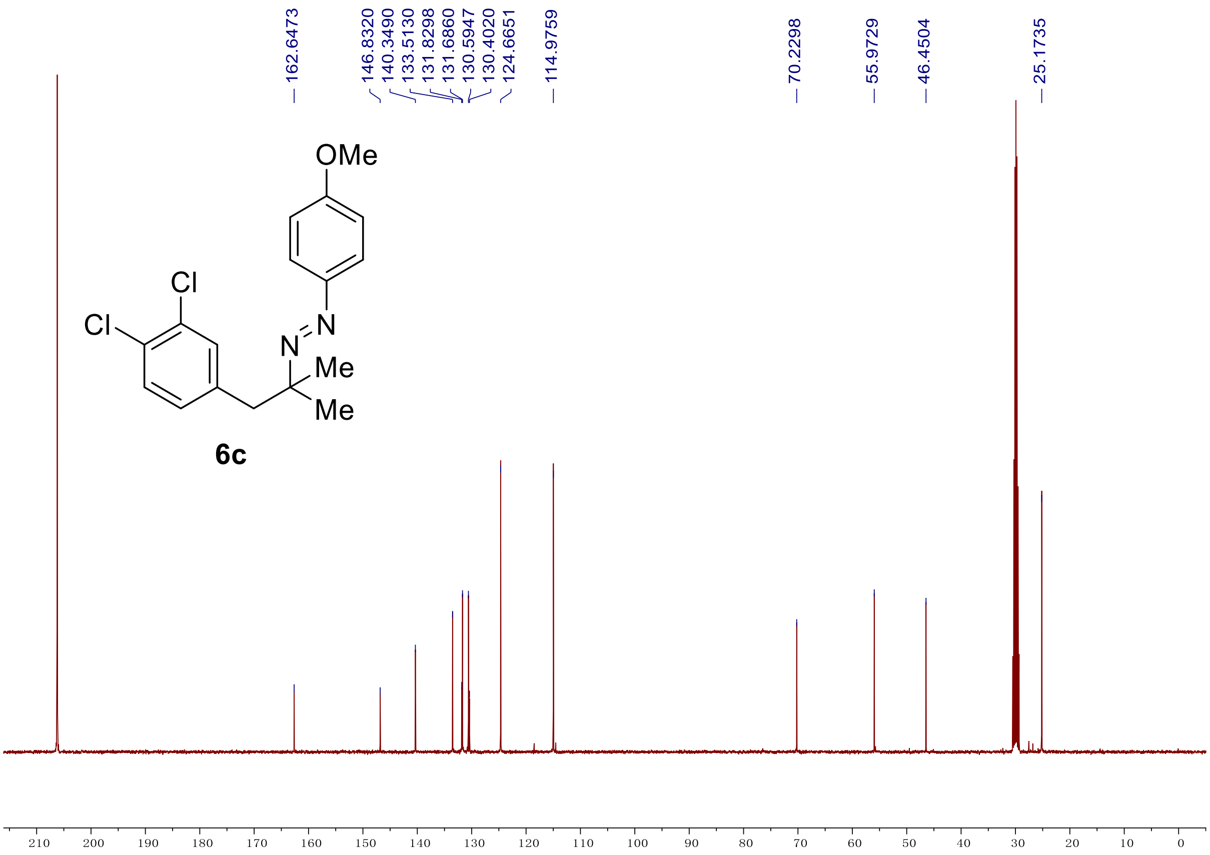

Supplementary Fig. 130. ^{13}C NMR of compound **6c.**

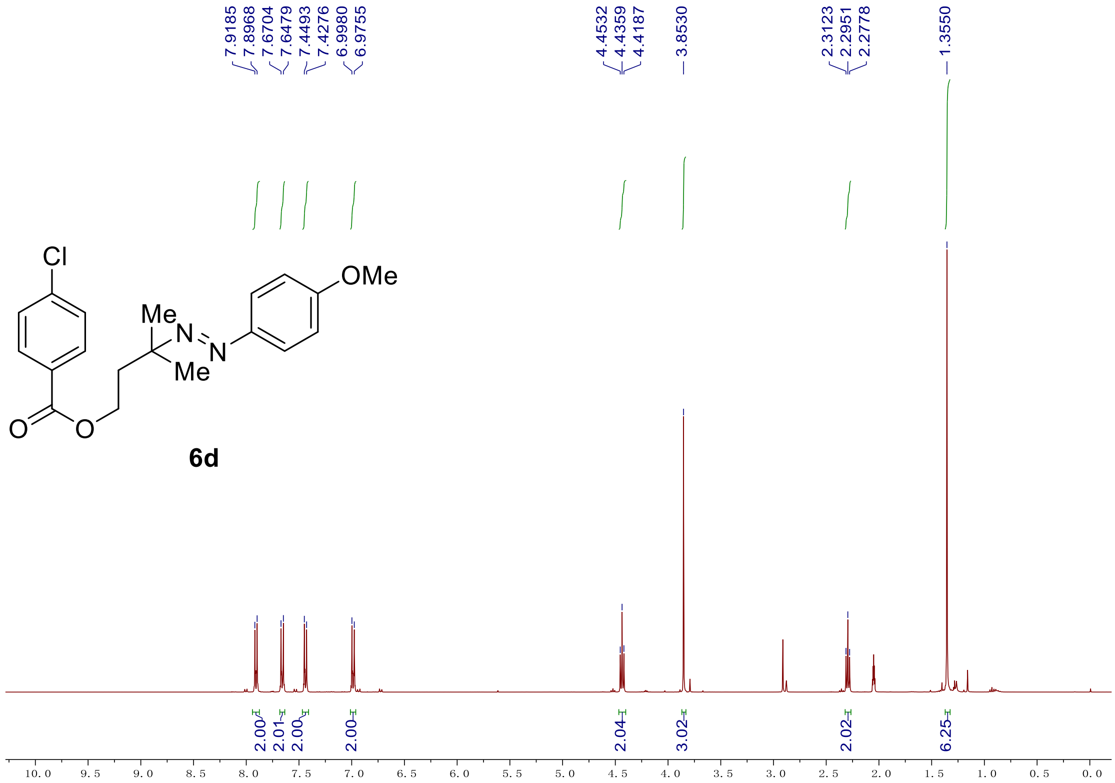

Supplementary Fig. 131. ^{1}H NMR of compound **6d.**

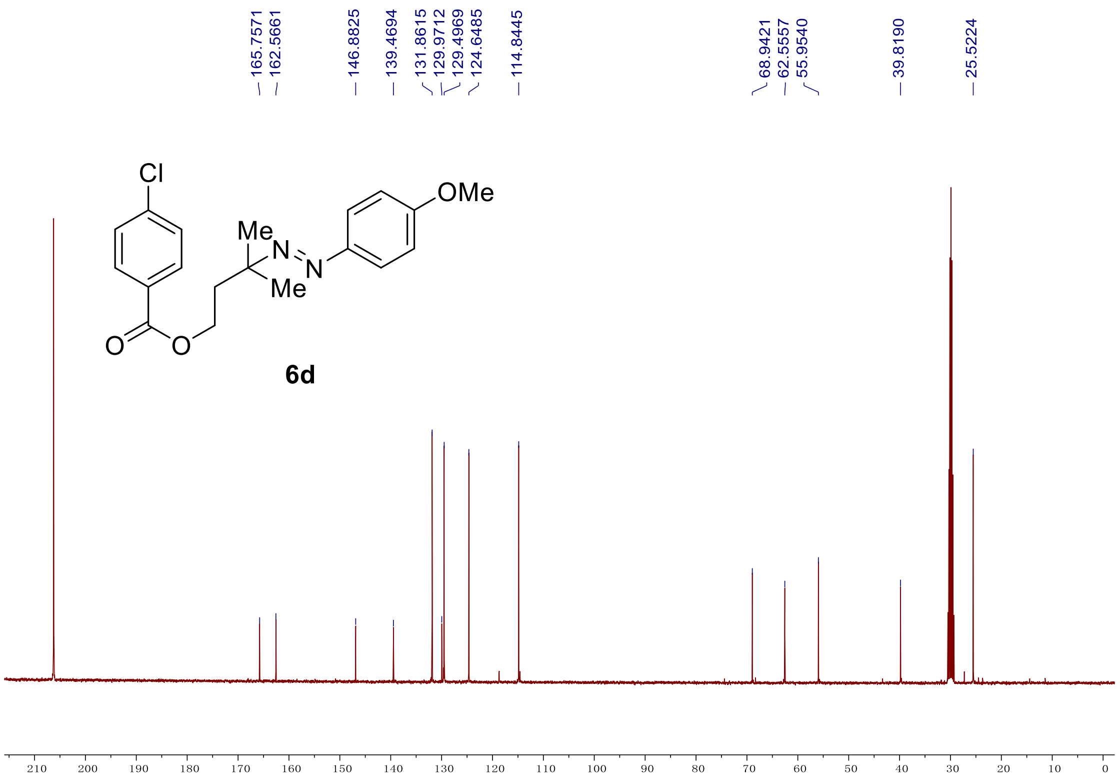

Supplementary Fig. 132. ^{13}C NMR of compound **6d.**

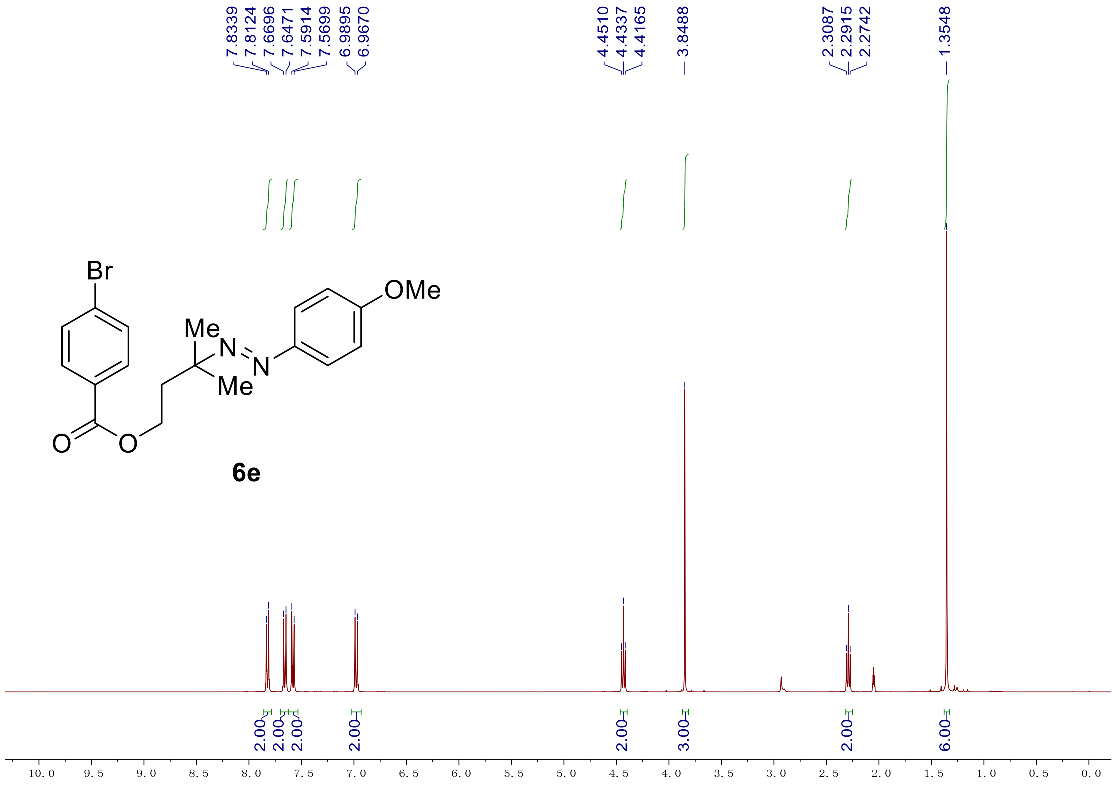

Supplementary Fig. 133. ^{1}H NMR of compound **6e.**

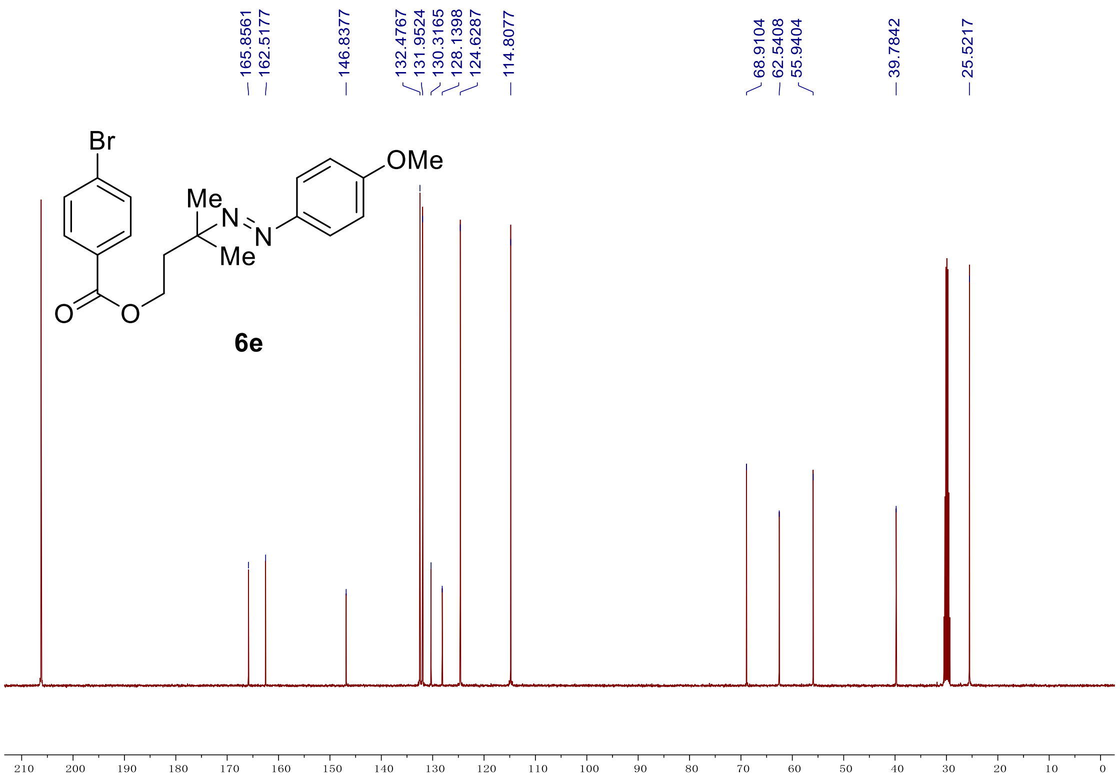

Supplementary Fig. 134. ^{13}C NMR of compound **6e.**

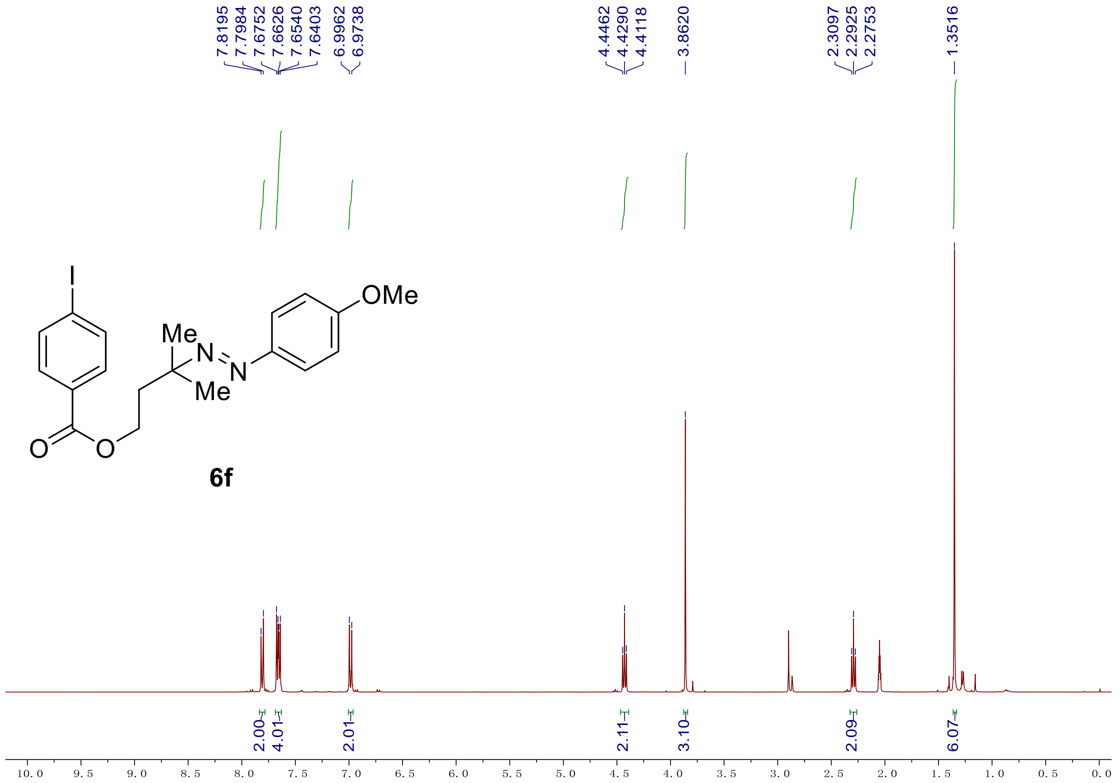

Supplementary Fig. 135. ^{1}H NMR of compound **6f.**

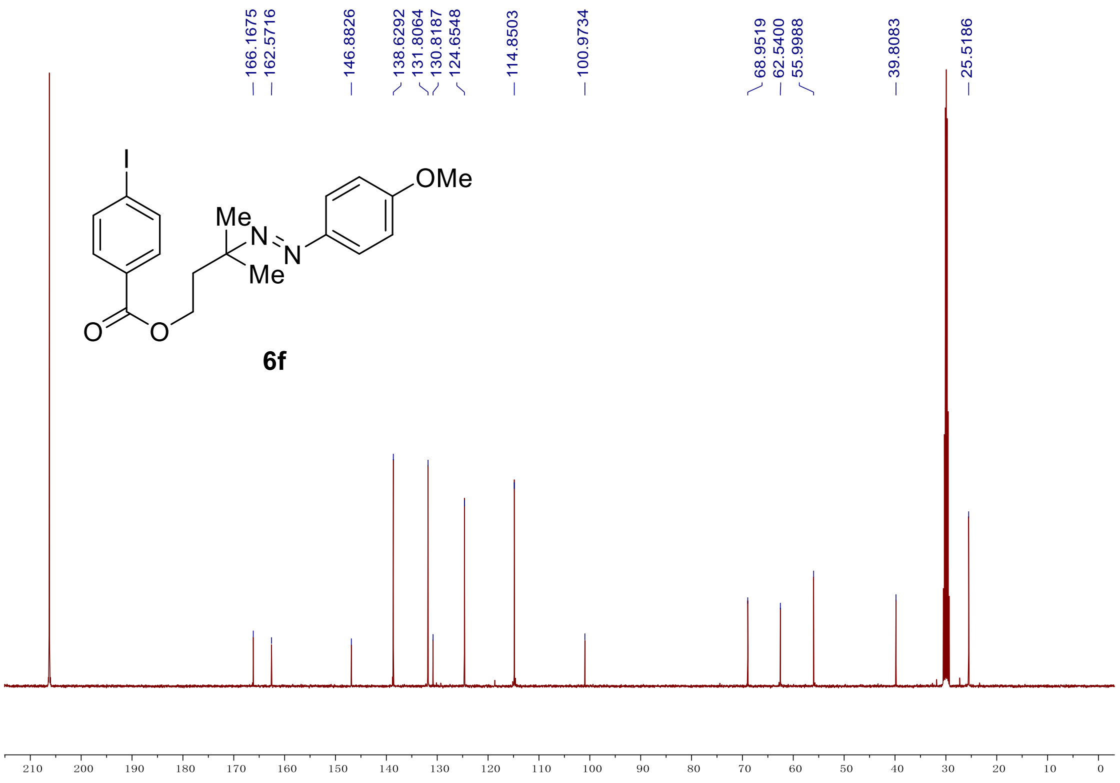

Supplementary Fig. 136. ^{13}C NMR of compound **6f.**

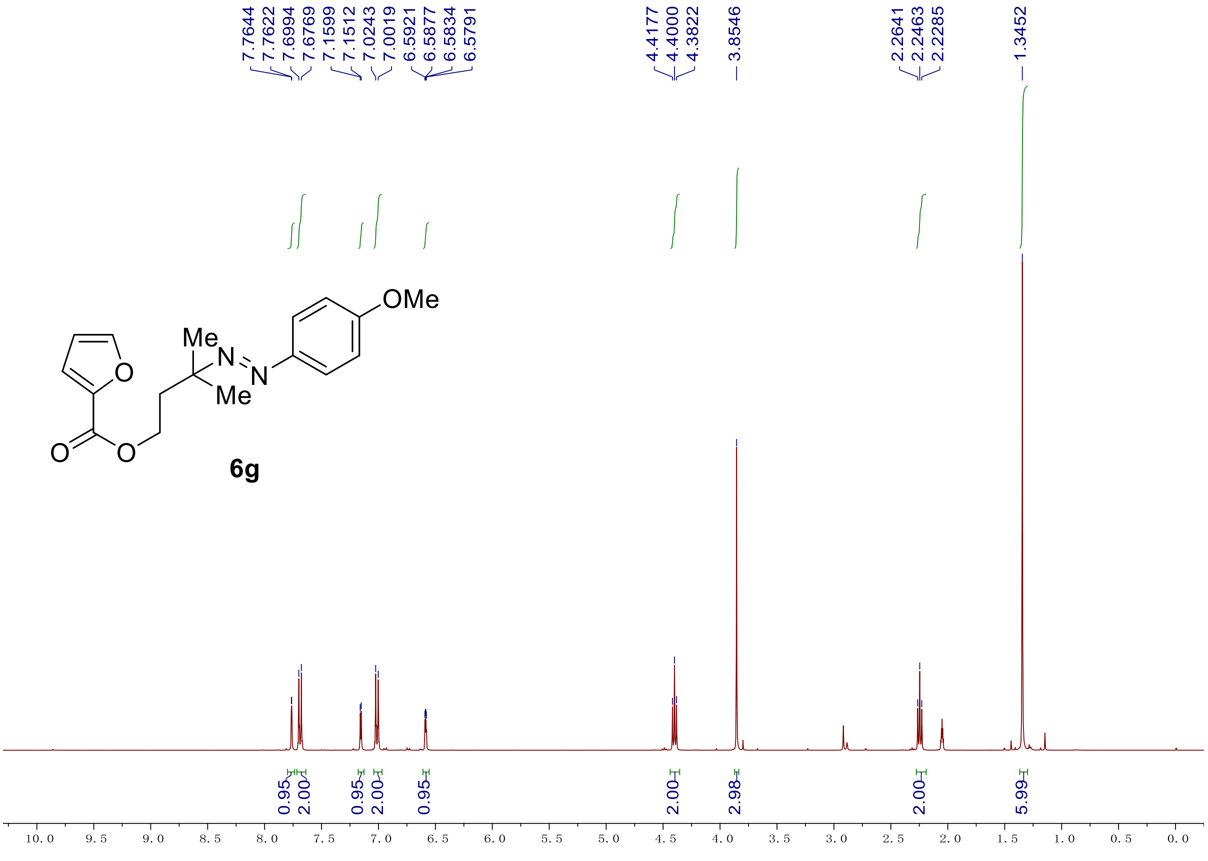

Supplementary Fig. 137. ^{1}H NMR of compound **6g.**

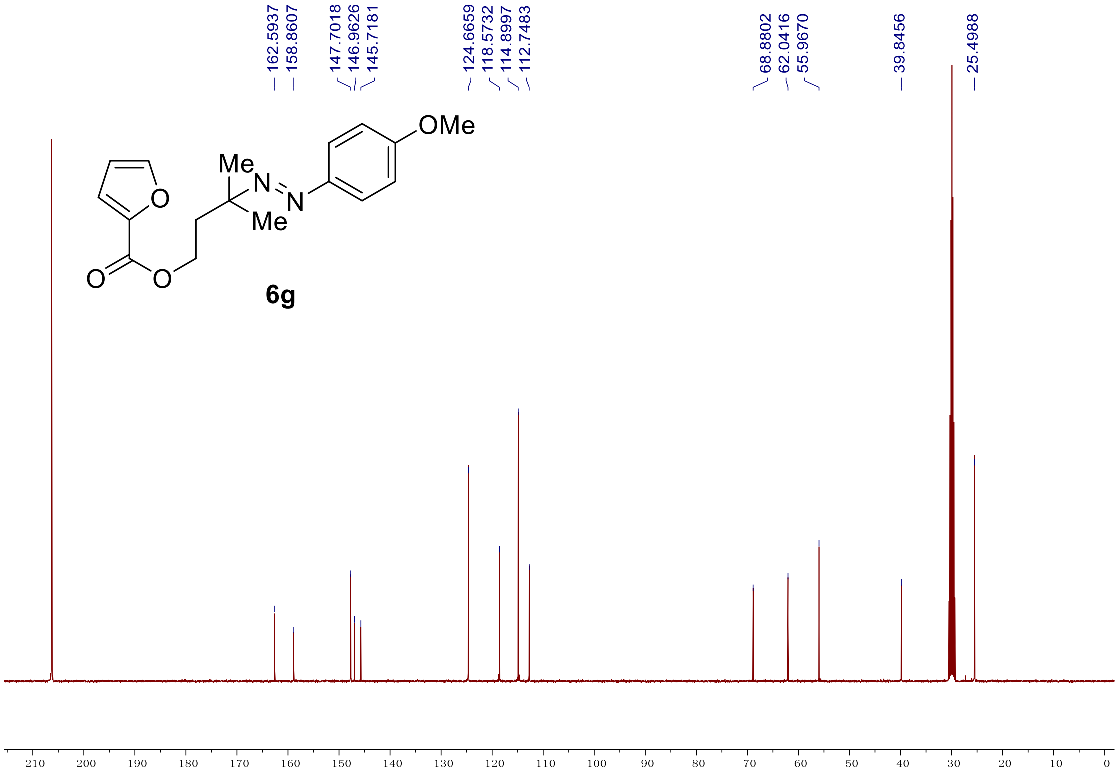

Supplementary Fig. 138. ^{13}C NMR of compound **6g.**

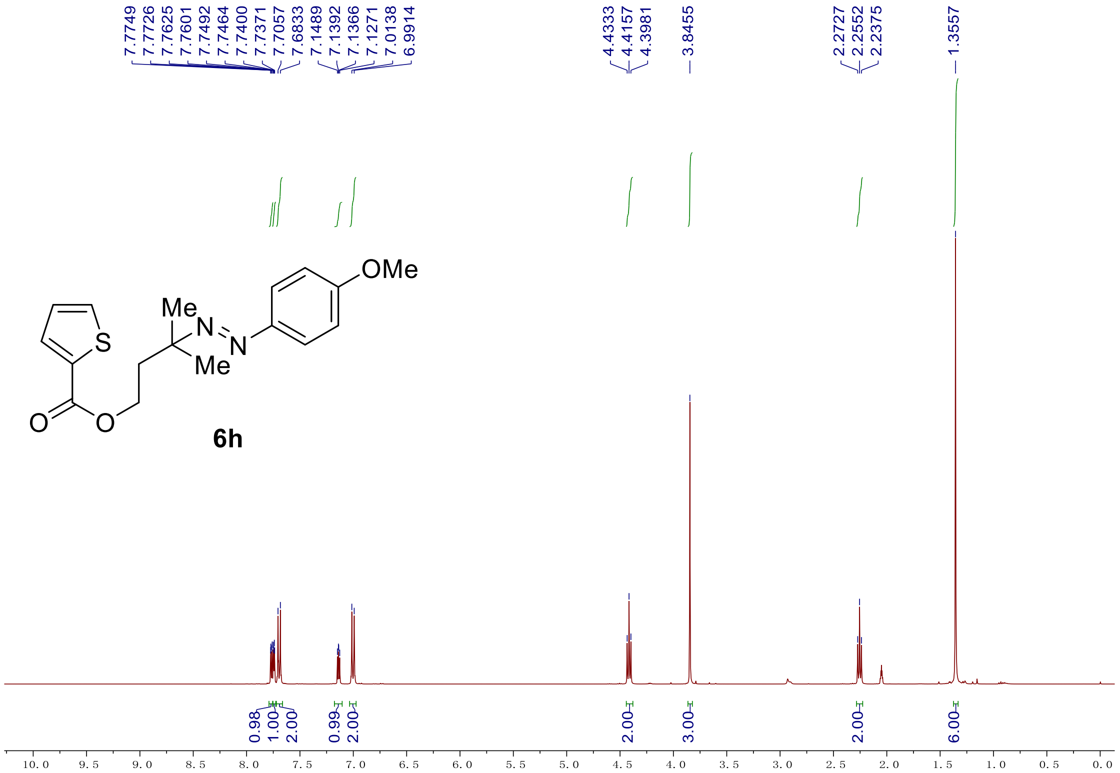

Supplementary Fig. 139. ^{1}H NMR of compound **6h.**

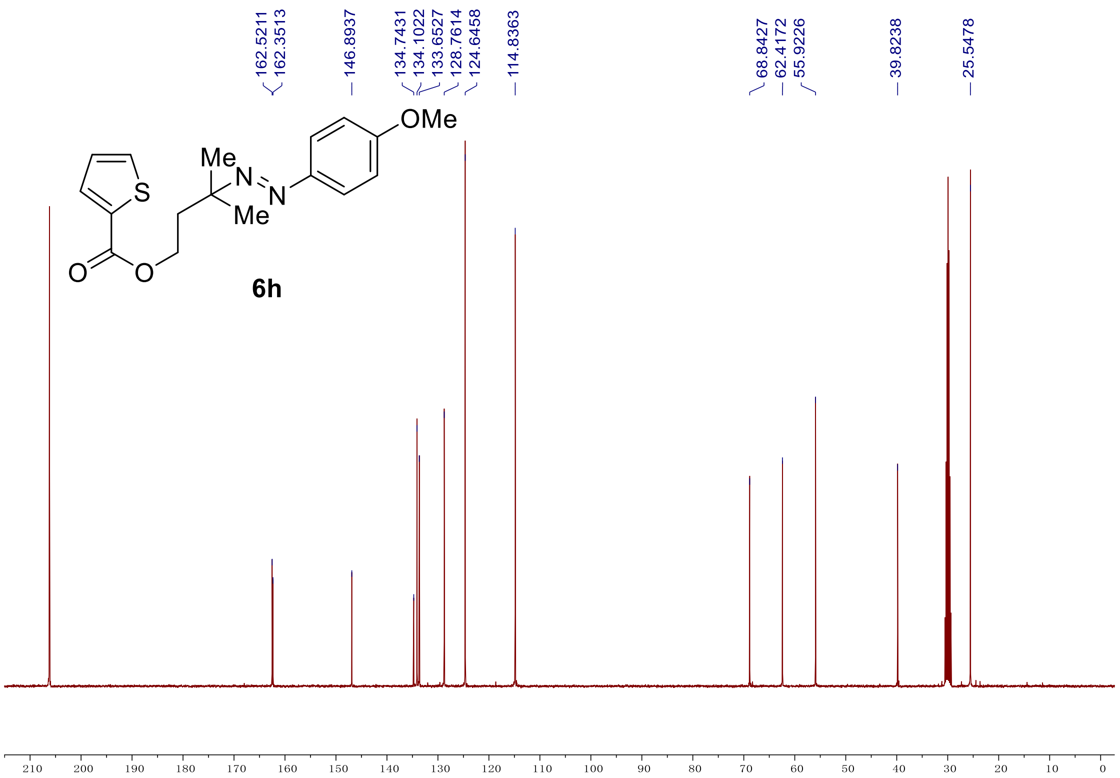

Supplementary Fig. 140. ^{13}C NMR of compound **6h.**

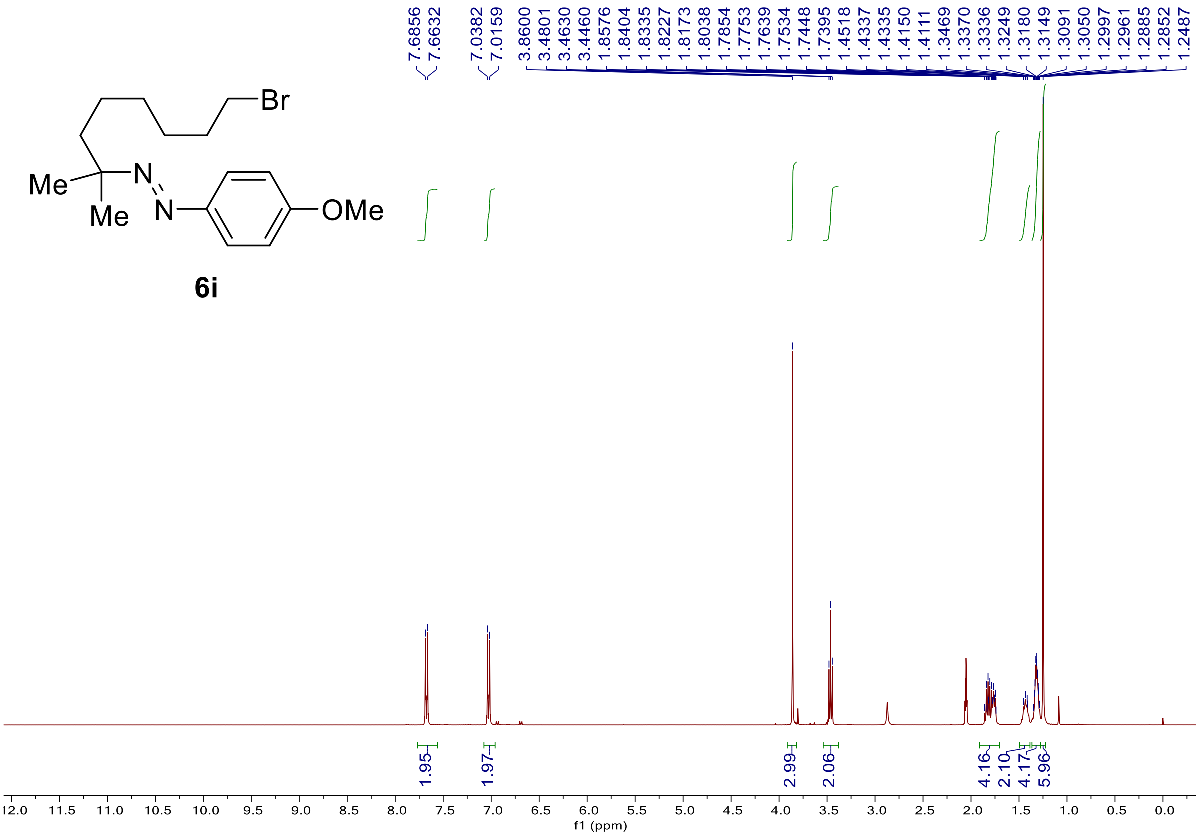

Supplementary Fig. 141. ^{1}H NMR of compound **6i.**

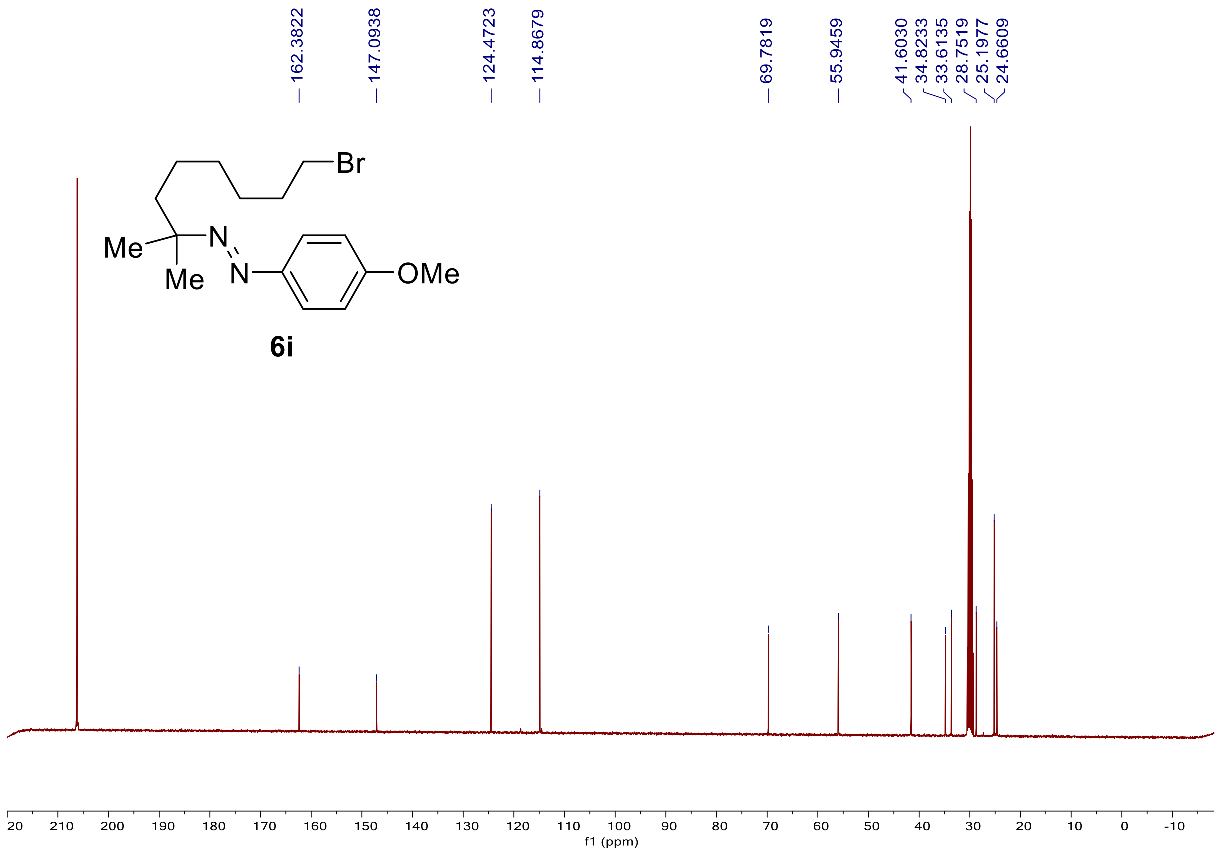

Supplementary Fig. 142. ^{13}C NMR of compound **6i.**

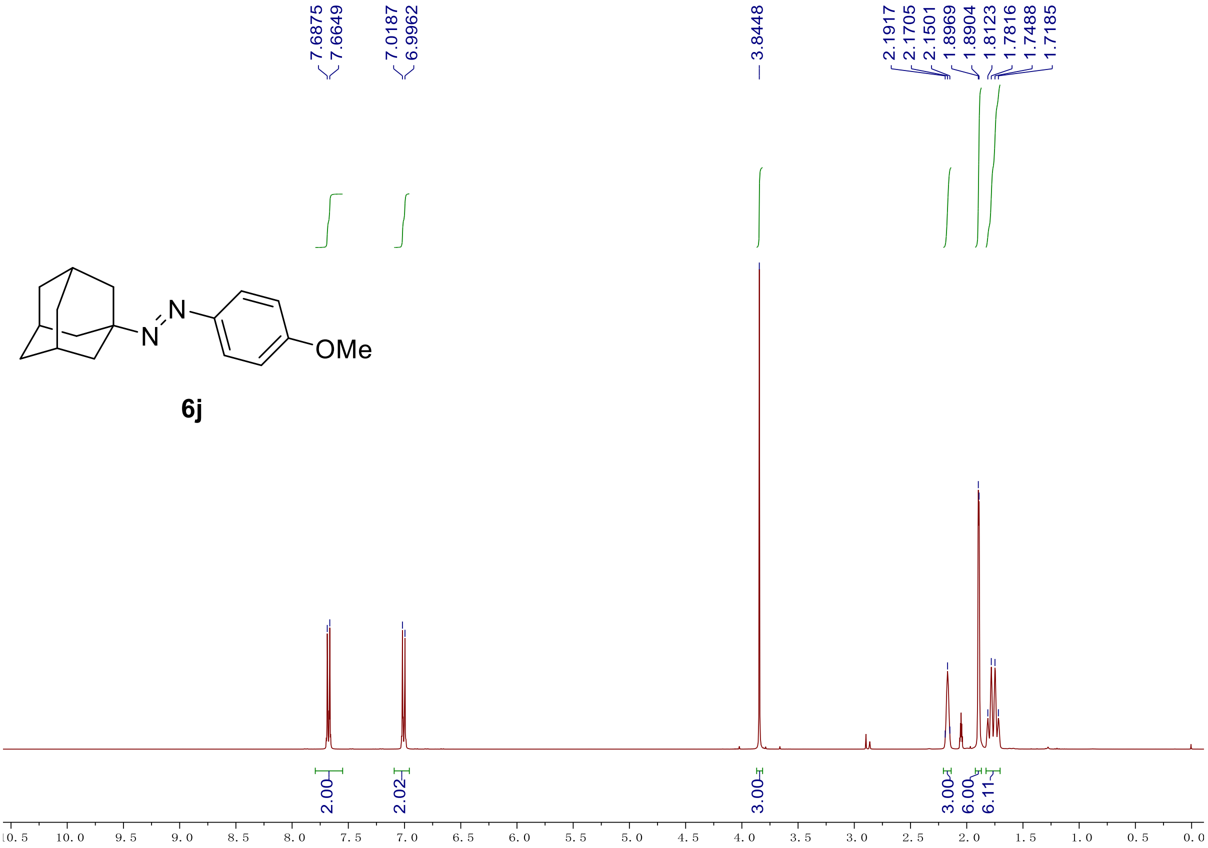

Supplementary Fig. 143. ^{1}H NMR of compound **6j.**

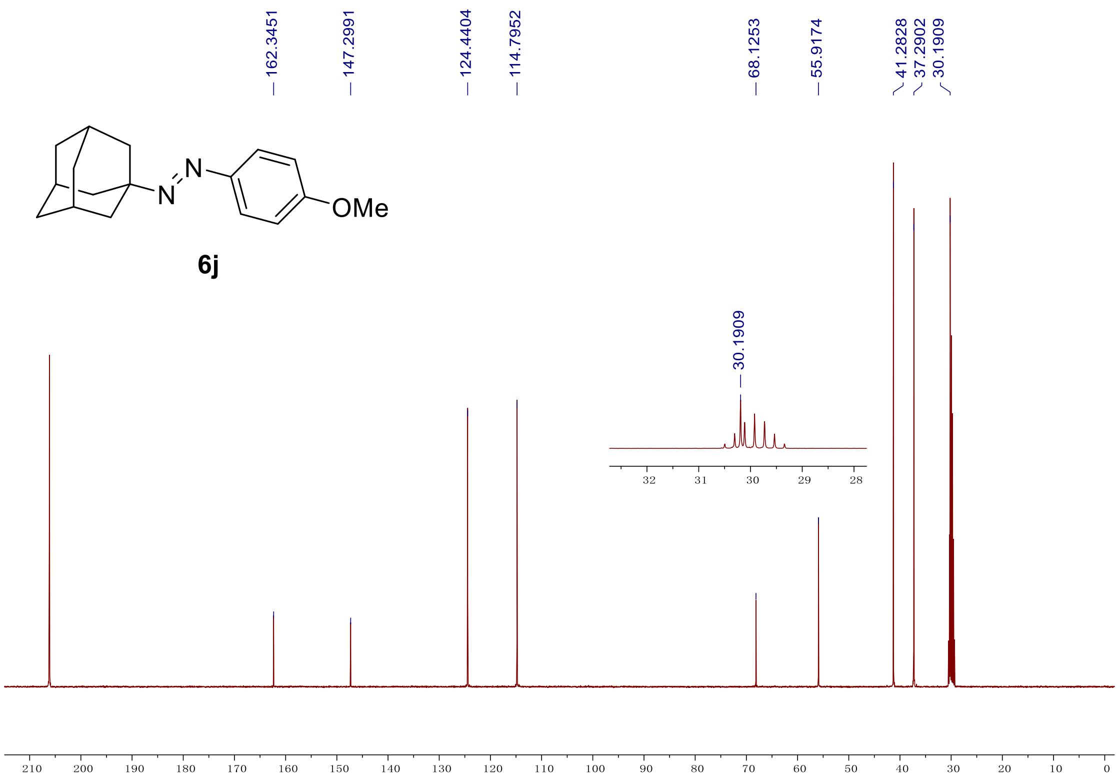

Supplementary Fig. 144. ^{13}C NMR of compound **6j.**

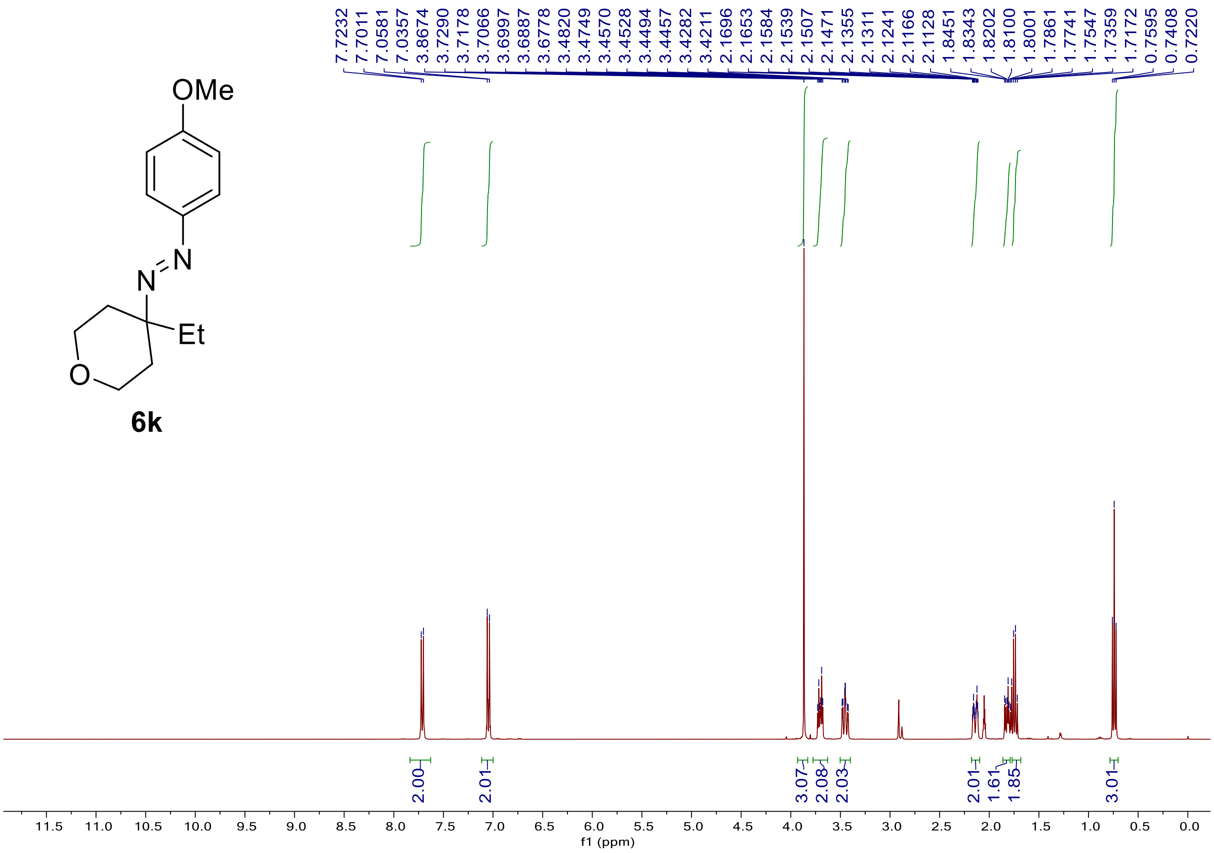

Supplementary Fig. 145. ^{1}H NMR of compound **6k.**

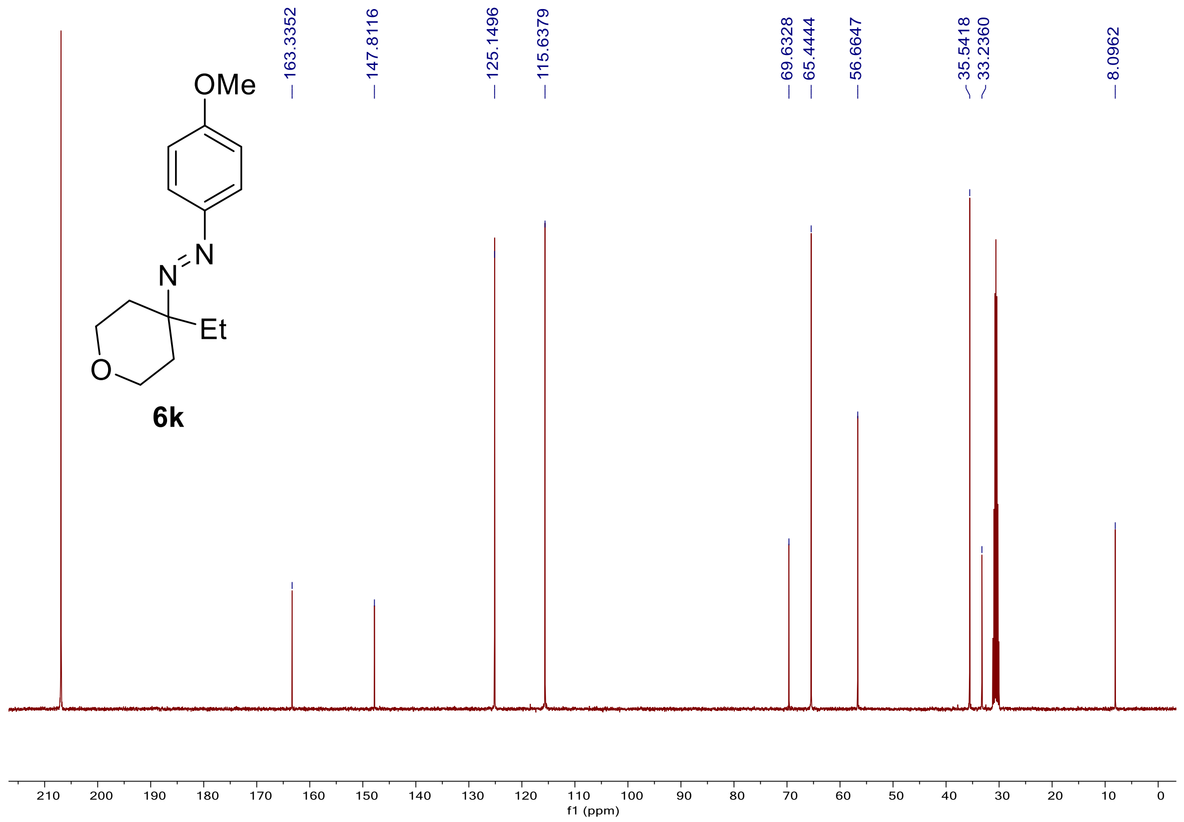

Supplementary Fig. 146. ^{13}C NMR of compound **6k.**

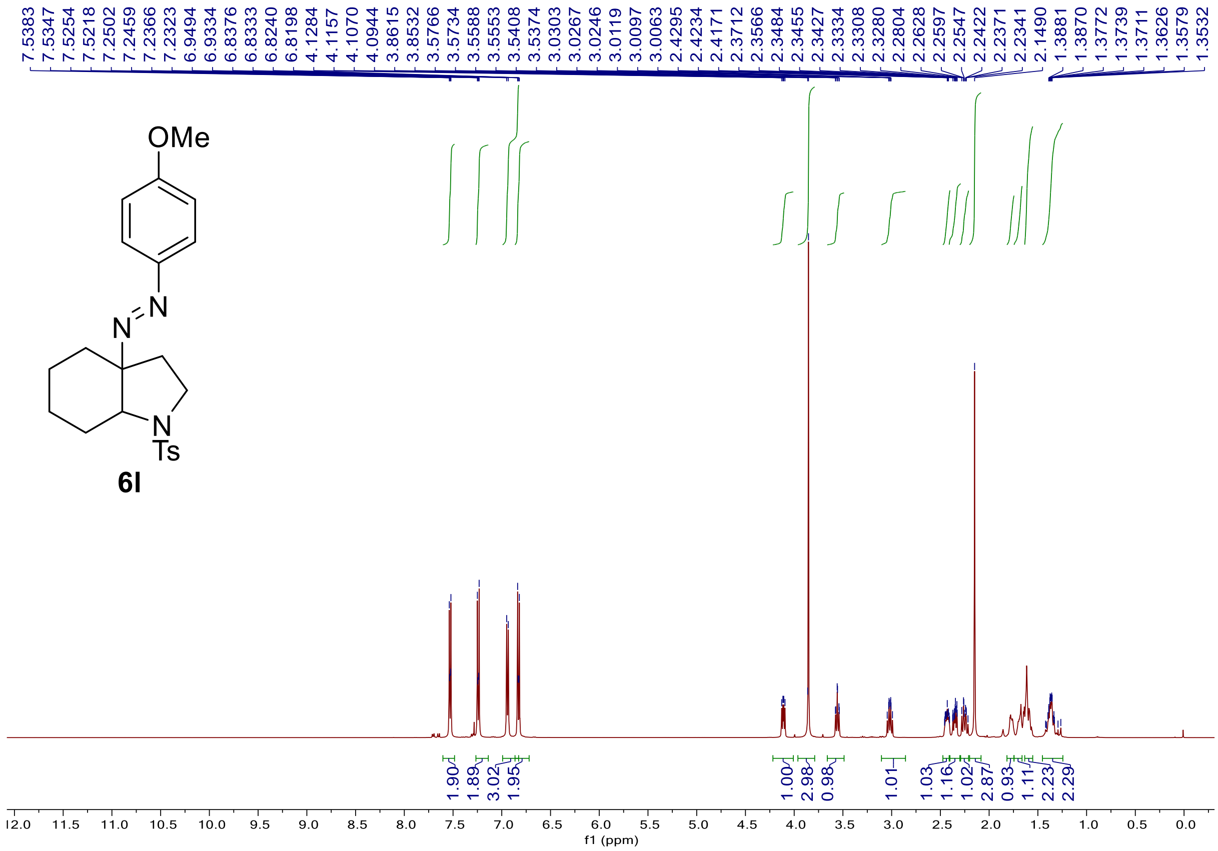

Supplementary Fig. 147. ^{1}H NMR of compound **6l.**

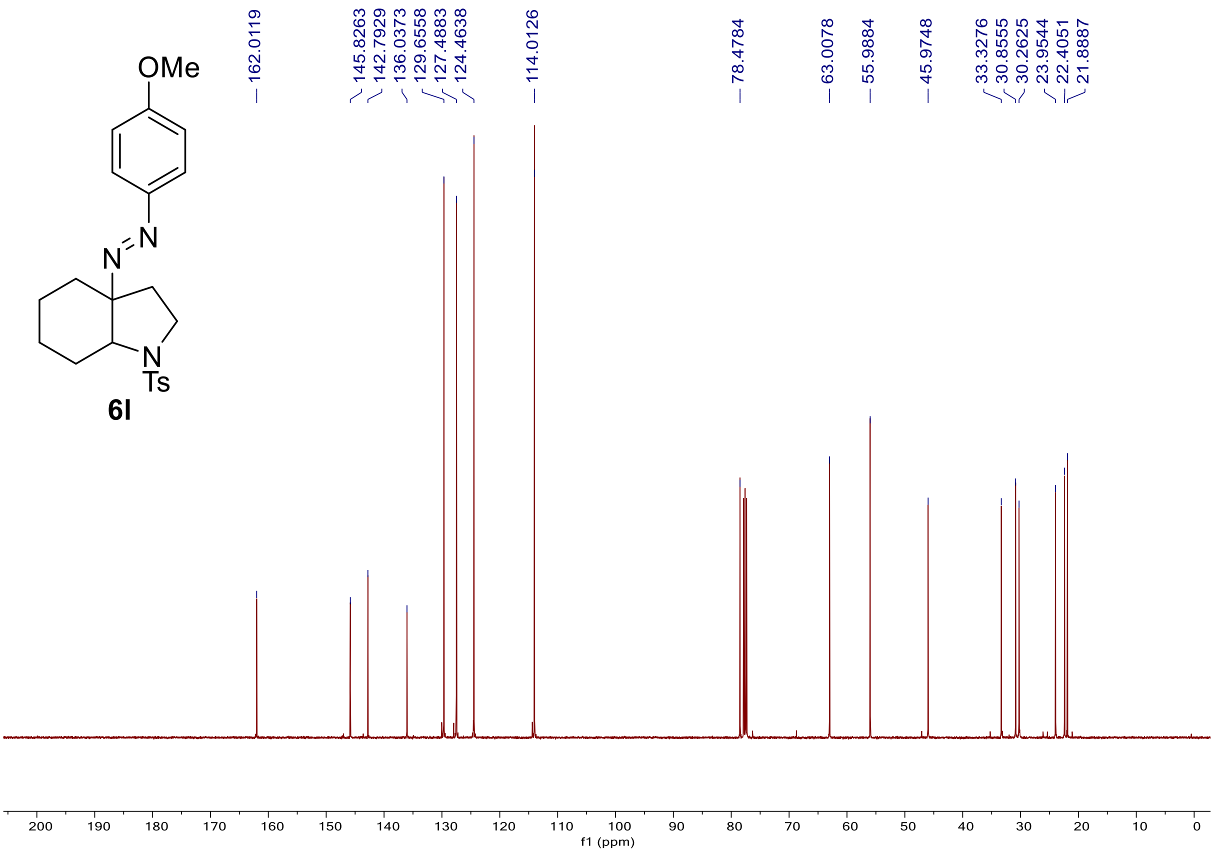

Supplementary Fig. 148. ^{13}C NMR of compound **6l.**

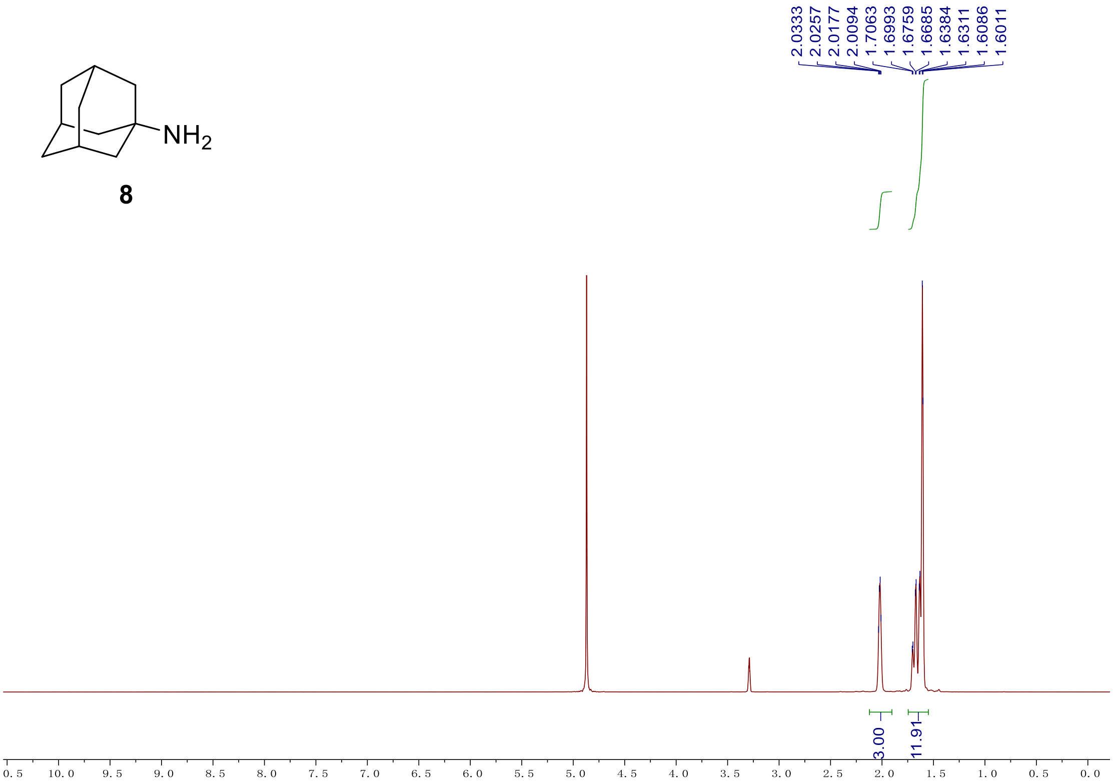

Supplementary Fig. 149. ^{1}H NMR of compound **8.**

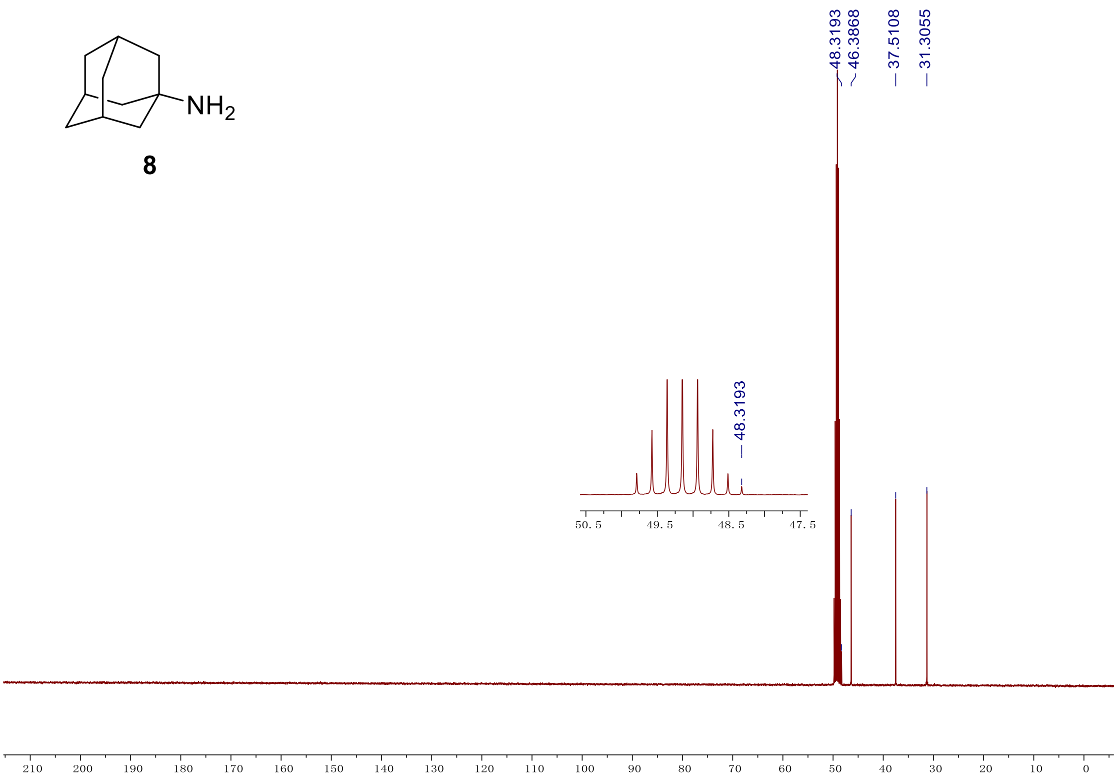

Supplementary Fig. 150. ^{13}C NMR of compound **8.**

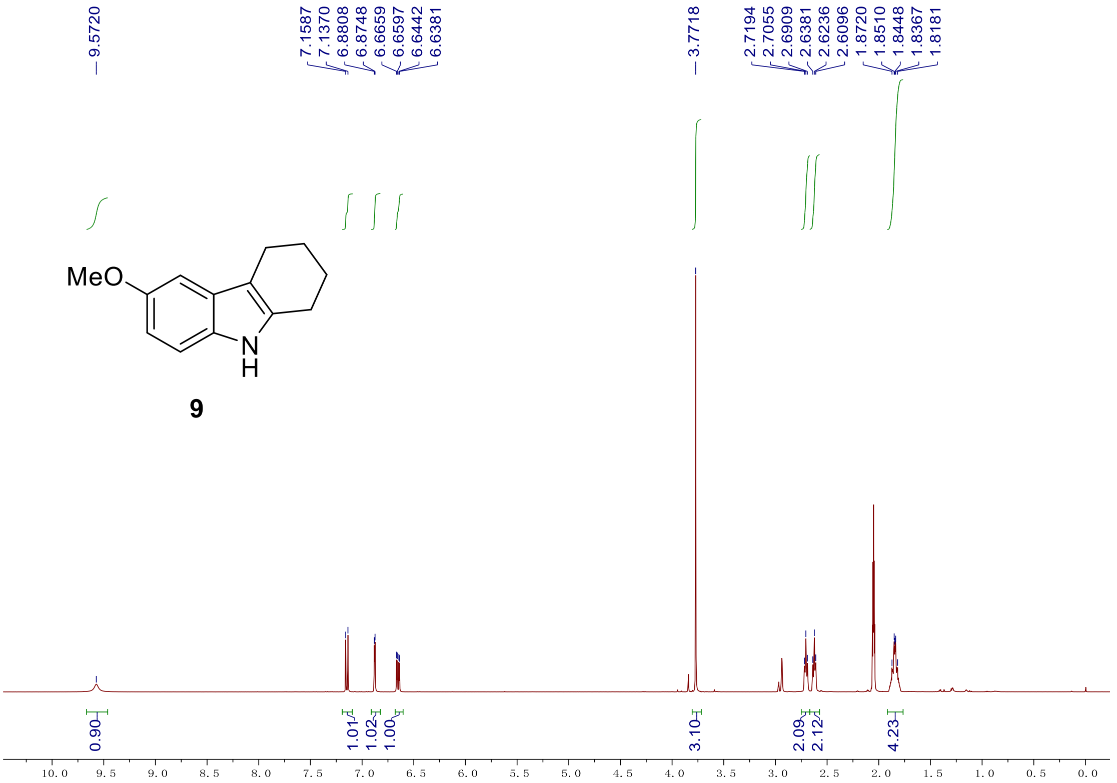

Supplementary Fig. 151. ^{1}H NMR of compound **9.**

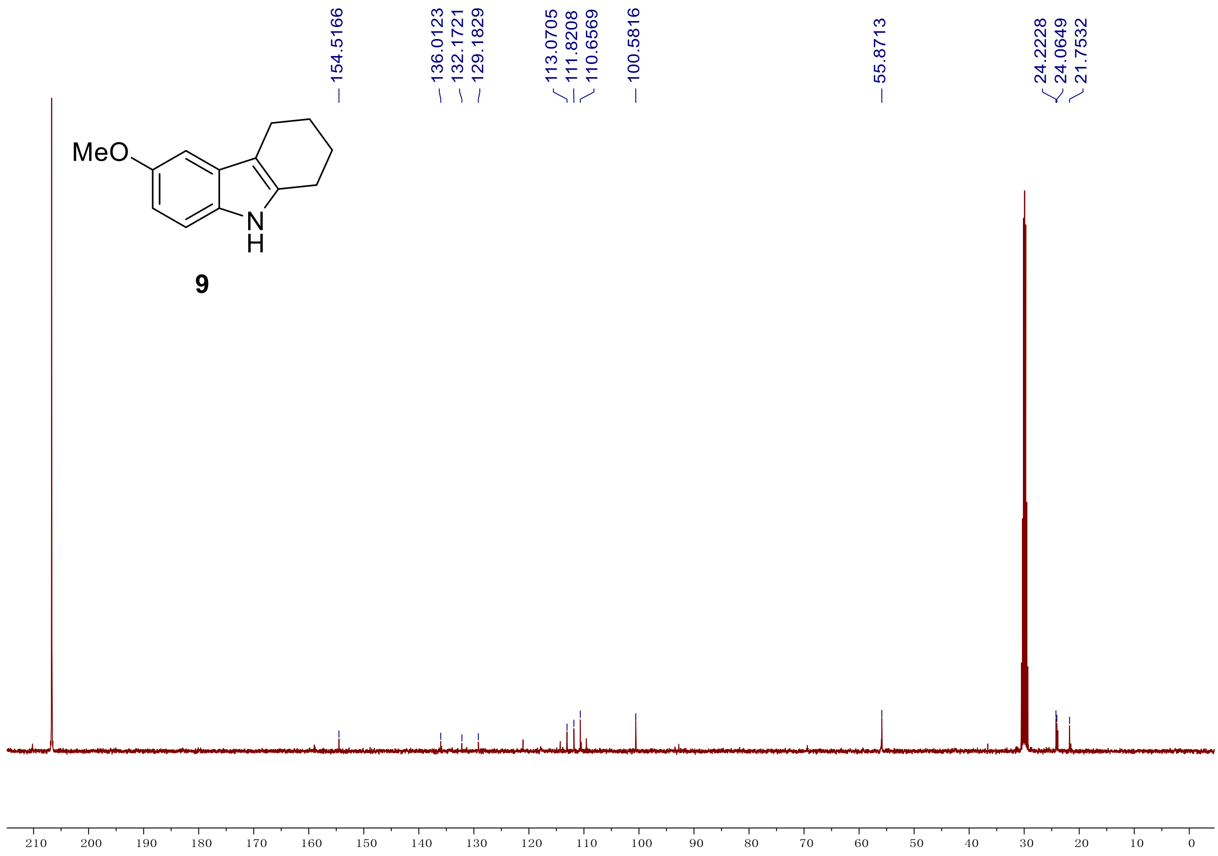

Supplementary Fig. 152. ^{13}C NMR of compound **9.**

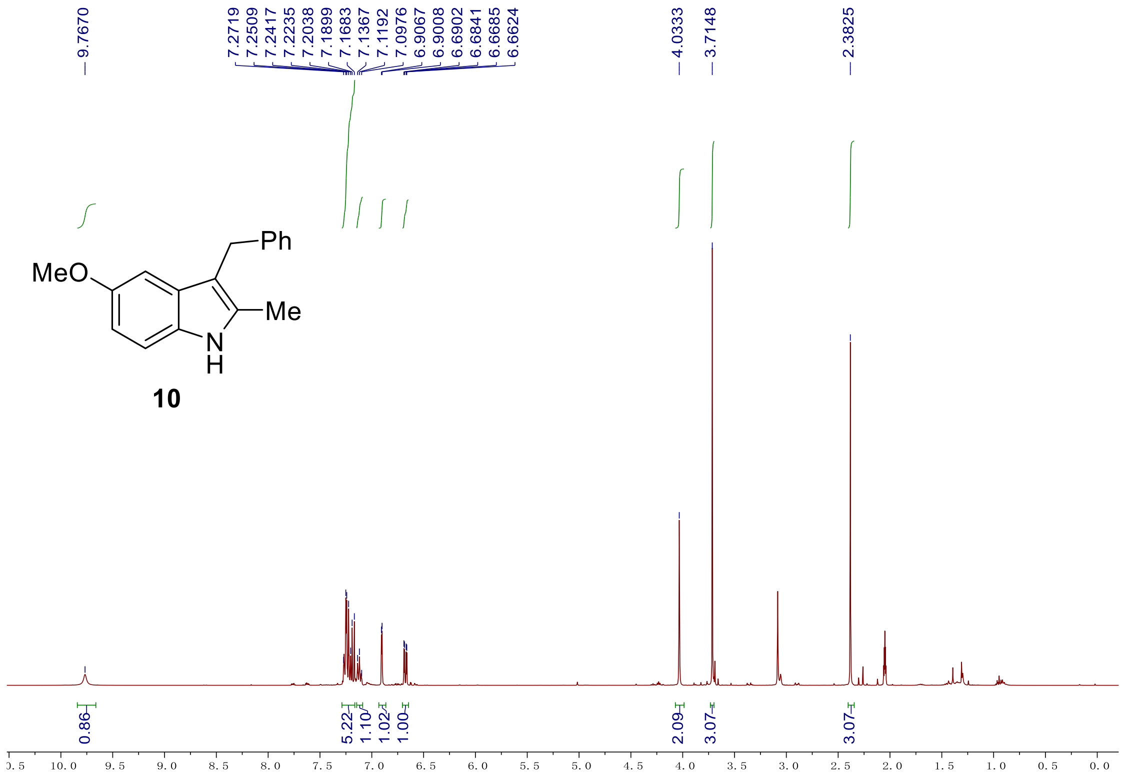

Supplementary Fig. 153. ^{1}H NMR of compound **10.**

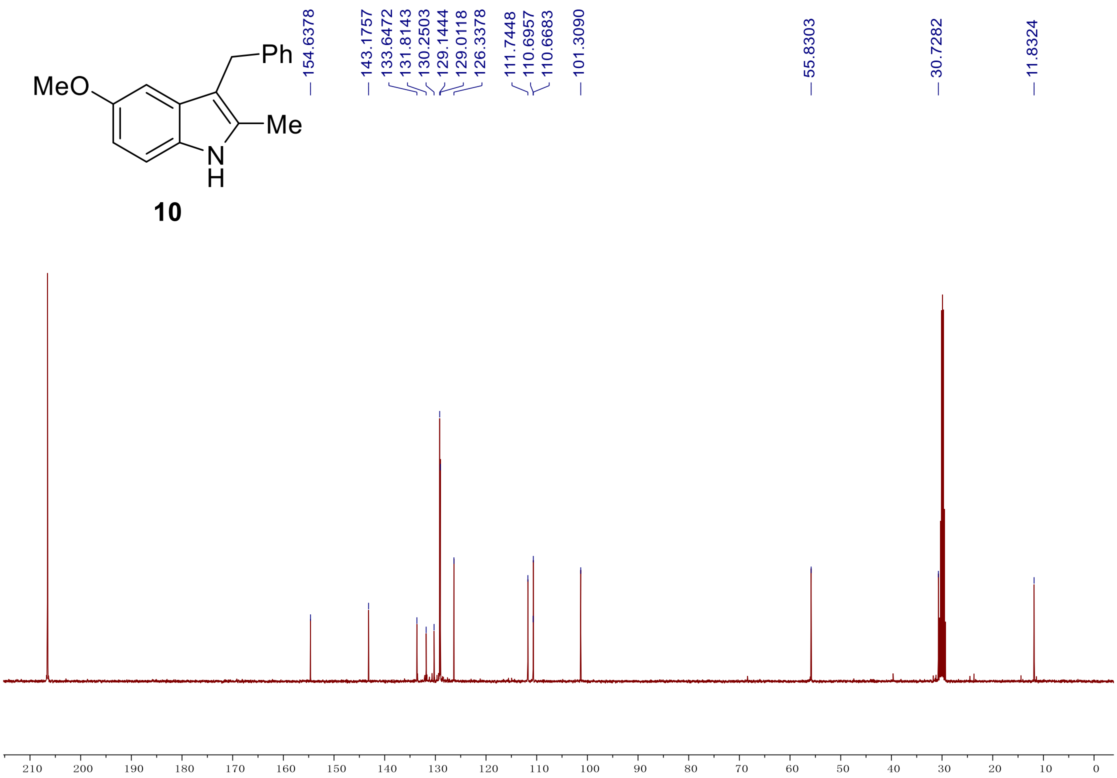

Supplementary Fig. 154. ^{13}C NMR of compound **10.**

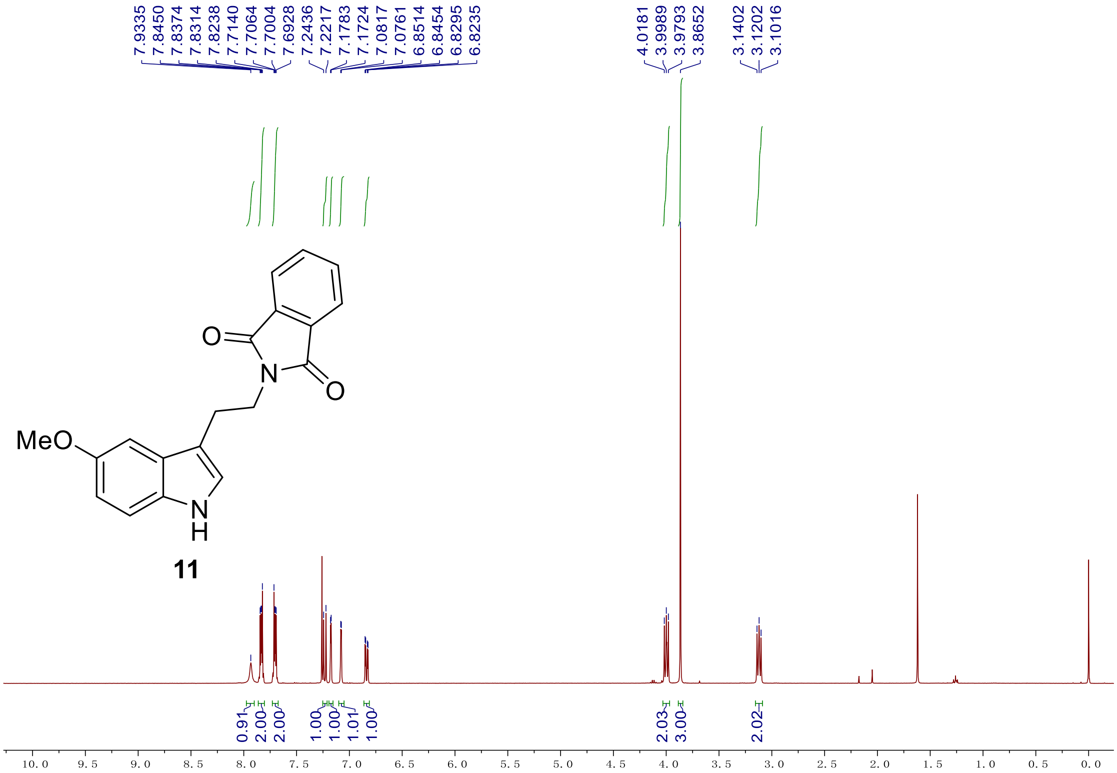

Supplementary Fig. 158. ^{1}H NMR of compound **11.**

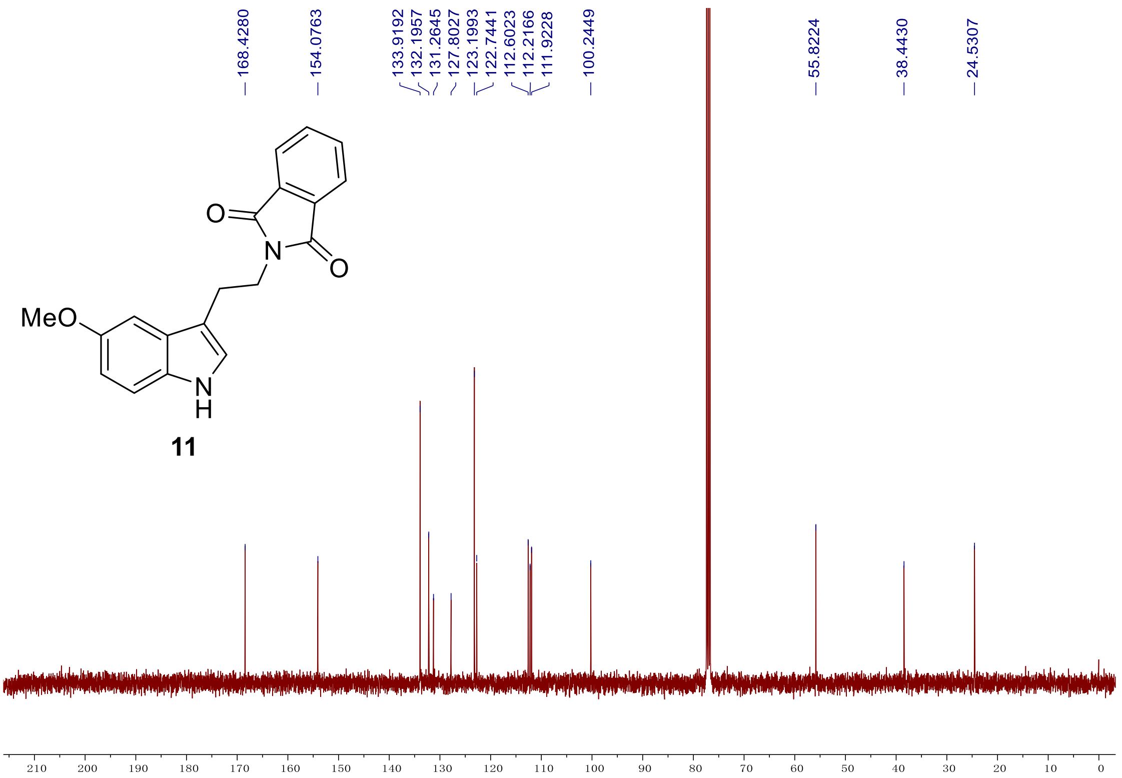

Supplementary Fig. 155. ^{13}C NMR of compound **11.**

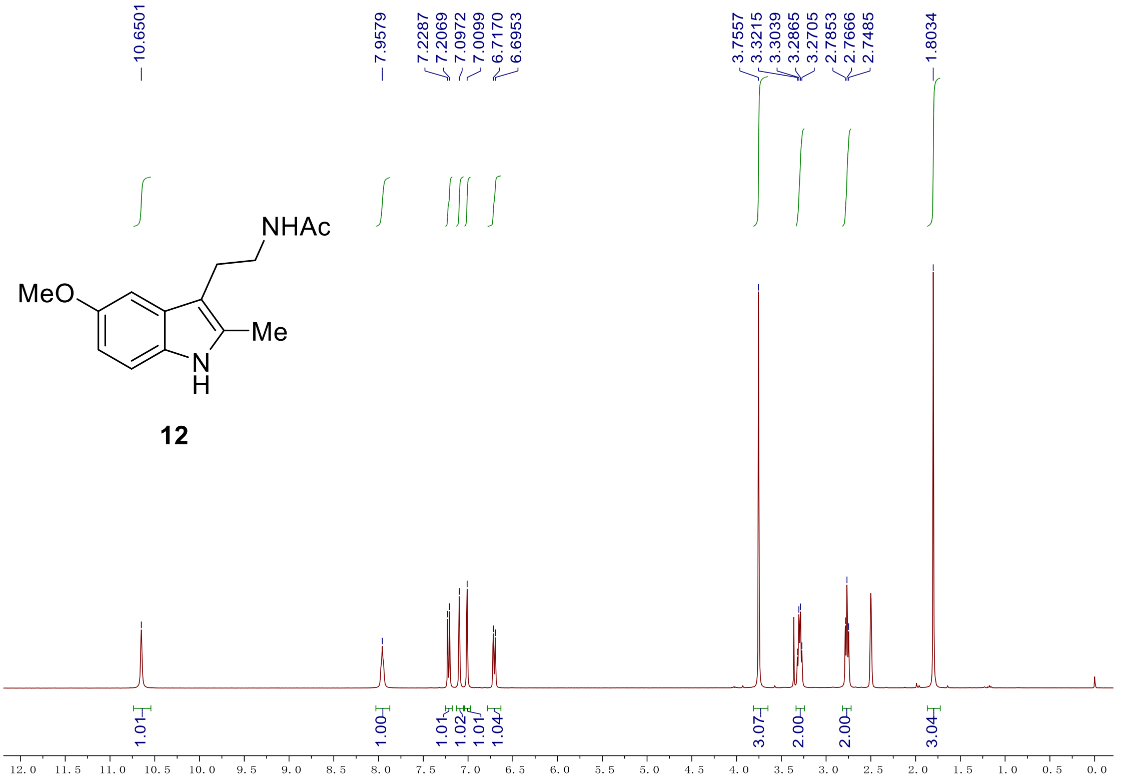

Supplementary Fig. 156. ^{1}H NMR of compound **12.**

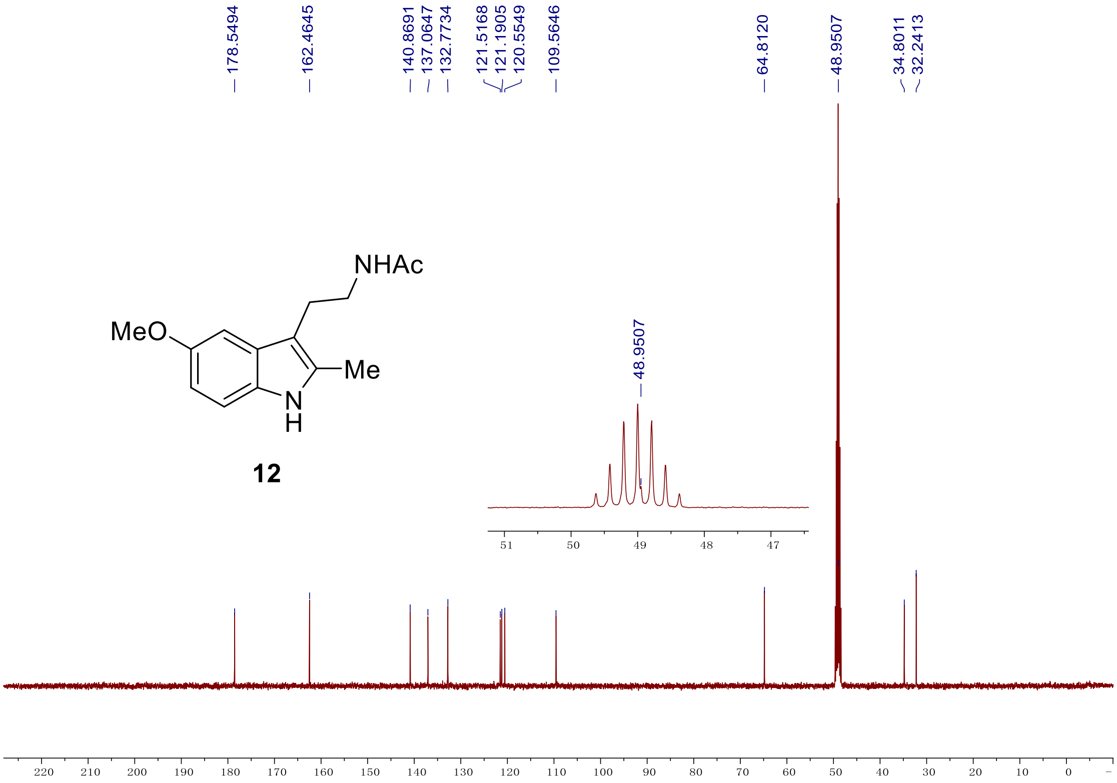

Supplementary Fig. 157. ^{13}C NMR of compound **12.**

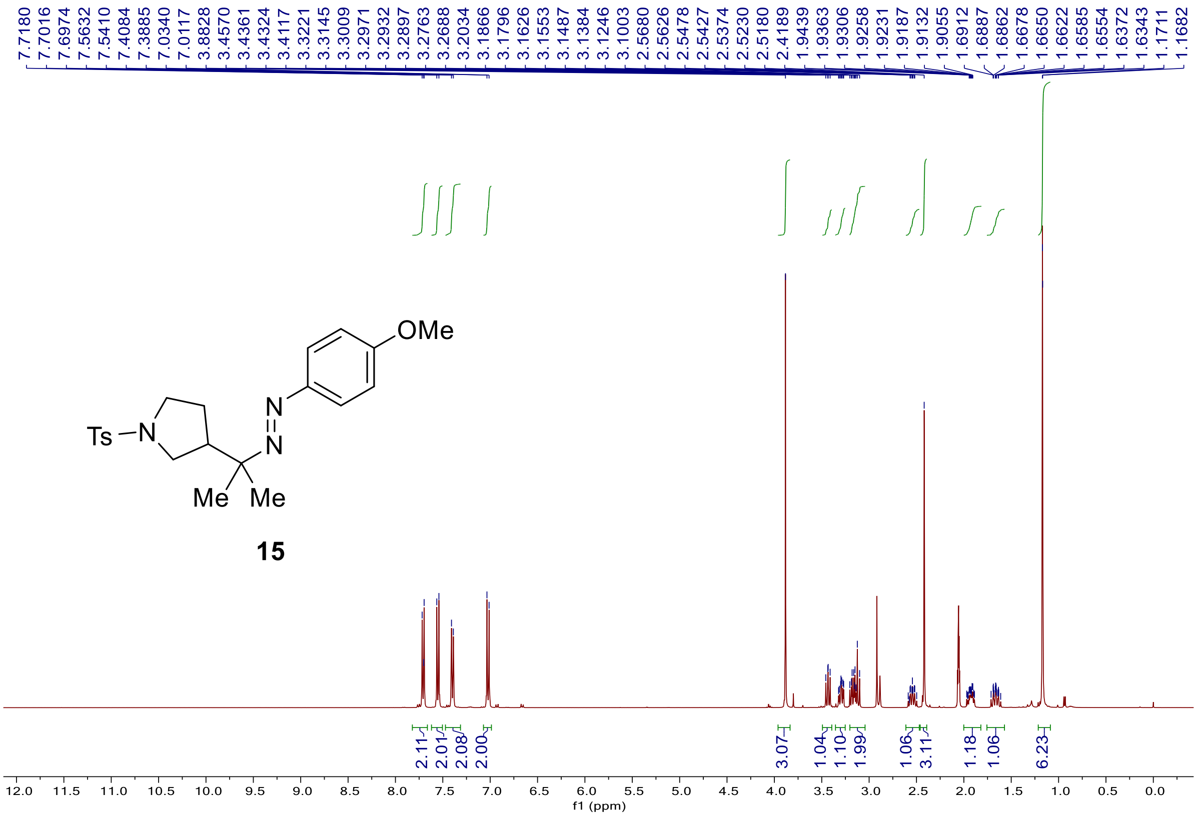

Supplementary Fig. 162. ^{1}H NMR of compound **15.**

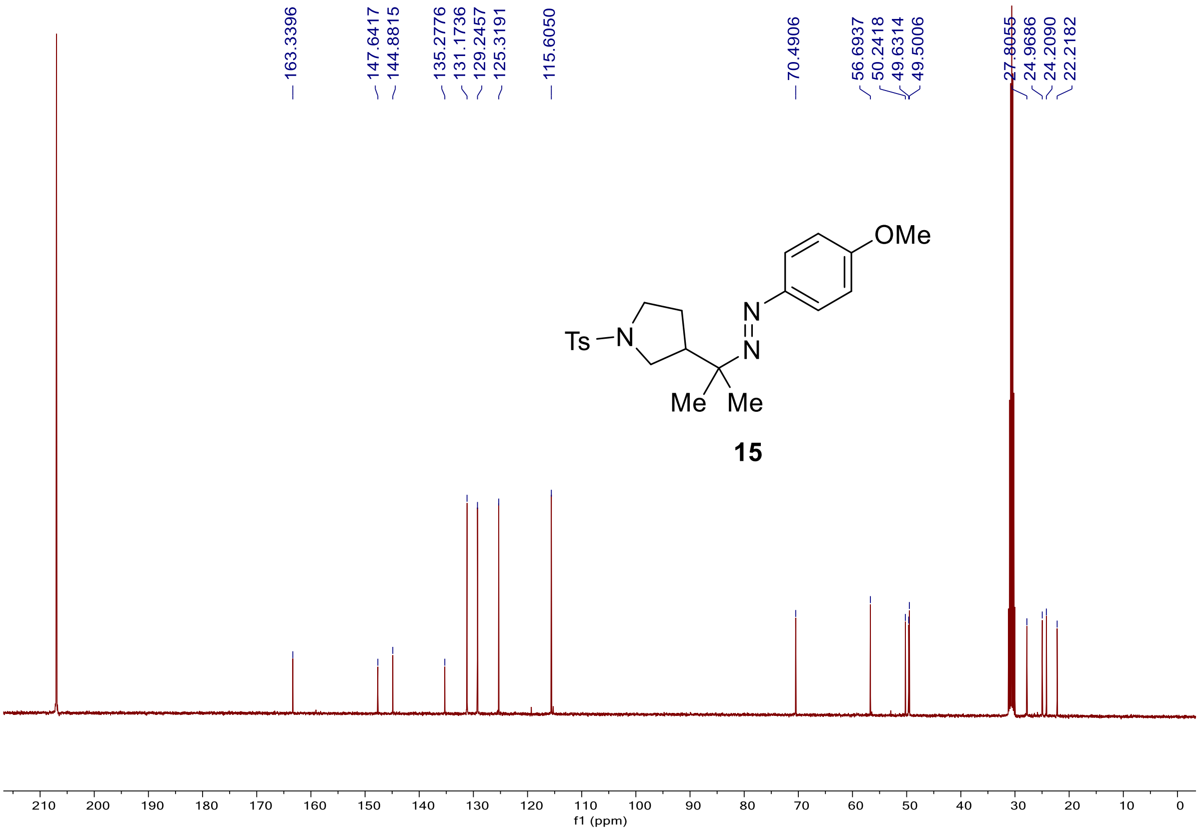

Supplementary Fig. 158. ^{13}C NMR of compound **15.**

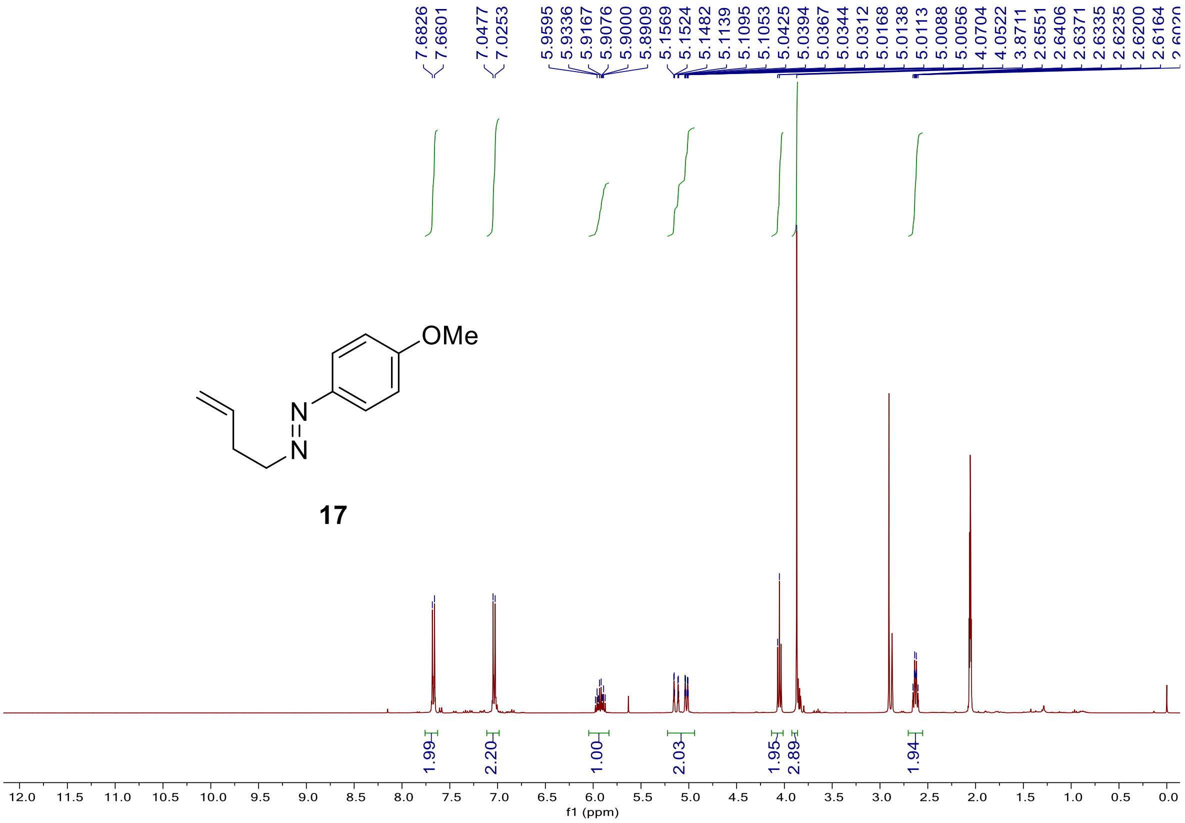

Supplementary Fig. 159. ^{1}H NMR of compound **17.**

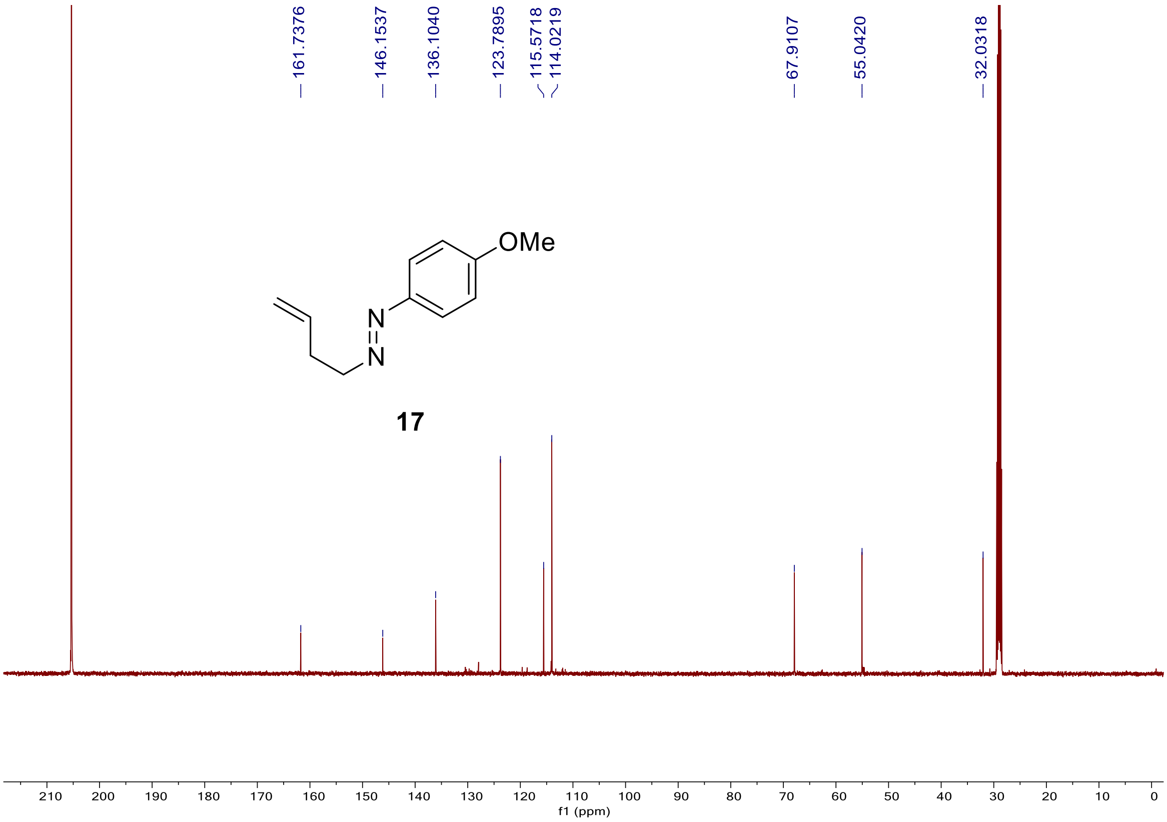

Supplementary Fig. 160. ^{13}C NMR of compound **17.**

Supplementary References

16. References

1. Wu, J.; Gu, Y.; Leng, X.; Shen, Q., Copper-promoted sandmeyer difluoromethylthiolation of aryl and heteroaryl diazonium salts. *Angew. Chem. Int. Ed.* **2015**, *54*, 7648-52.
2. Liu, Y.; Pan, Q.; Hu, X.; Guo, Y.; Chen, Q. Y.; Liu, C., Rapid Access to N-Protected Sulfonimidoyl Fluorides: Divergent Synthesis of Sulfonamides and Sulfonimidamides. *Org. Lett.* **2021**, *23*, 3975-3980.
3. Chen, Y.; Su, L.; Gong, H., Copper-Catalyzed and Indium-Mediated Methoxycarbonylation of Unactivated Alkyl Iodides with Balloon CO. *Org. Lett.* **2019**, *21*, 4689-4693.
4. Chen, Y.; Ma, G.; Gong, H., Copper-Catalyzed Reductive Trifluoromethylation of Alkyl Iodides with Togni's Reagent. *Org. Lett.* **2018**, *20*, 4677-4680.
5. Liu, X.-G.; Dong, C.-S.; Li, F.; Zhang, B., Manganese-Mediated Direct Functionalization of Hantzsch Esters with Alkyl Iodides via an Aromatization–Dearomatization Strategy. *Org. Lett.* **2021**, *23*, 4002-4007.
6. Villalobos, A.; Blake, J. F.; Biggers, C. K.; Butler, T. W.; Chapin, D. S.; Chen, Y. L.; Ives, J. L.; Jones, S. B.; Liston, D. R., Novel Benzisoxazole Derivatives as Potent and Selective Inhibitors of Acetylcholinesterase. *J. Med. Chem.* **1994**, *37*, 2721-2734.
7. Liu, Q.; Hong, J.; Sun, B.; Bai, G.; Li, F.; Liu, G.; Yang, Y.; Mo, F., Transition-Metal-Free Borylation of Alkyl Iodides via a Radical Mechanism. *Org. Lett.* **2019**, *21*, 6597-6602.
8. Caiger, L.; Sinton, C.; Constantin, T.; Douglas, J. J.; Sheikh, N. S.; Juliá, F.; Leonori, D., Radical hydroxymethylation of alkyl iodides using formaldehyde as a C1 synthon. *Chem. Sci.* **2021**, *12*, 10448-10454.
9. Ortega, N.; Feher-Voelger, A.; Brovetto, M.; Padrón, J. I.; Martín, V. S.; Martín, T., Iron(III)-Catalyzed Halogenations by Substitution of Sulfonate Esters. *Adv. Synth. Catal.* **2011**, *353*, 963-972.
10. Louafi, F.; Moreau, J.; Shahane, S.; Golhen, S.; Roisnel, T.; Sinbandhit, S.; Hurvois, J.-P., Electrochemical Synthesis and Chemistry of Chiral 1-Cyanotetrahydroisoquinolines. An Approach to the Asymmetric Syntheses of the Alkaloid (−)-Crispine A and Its Natural (+)-Antipode. *J. Org. Chem.* **2011**, *76*, 9720-9732.
11. Sunagatullina, A. S.; Lutter, F. H.; Knochel, P., Preparation of Primary and Secondary Dialkylmagnesiums by a Radical I/Mg-Exchange Reaction Using sBu_2Mg in Toluene. *Angew. Chem. Int. Ed.* **2022**, *61*, e202116625 (1 of 5) .
12. Moriya, T.; Yoneda, S.; Kawana, K.; Ikeda, R.; Konakahara, T.; Sakai, N., Indium(III)-Catalyzed Reductive Bromination and Iodination of Carboxylic Acids to Alkyl Bromides and Iodides: Scope, Mechanism, and One-Pot Transformation to Alkyl Halides and Amine Derivatives. *J. Org. Chem.* **2013**, *78*, 10642-10650.
13. Wang, L.; Jiang, X.; Tang, P., Silver-mediated fluorination of alkyl iodides with $TMSCF_3$ as the fluorinating agent. *Org. Chem. Front.* **2017**, *4*, 1958-1961.
14. Yang, T.; Jiang, Y.; Luo, Y.; Lim, J. J. H.; Lan, Y.; Koh, M. J., Chemoselective Union of Olefins, Organohalides, and Redox-Active Esters Enables Regioselective Alkene Dialkylation. *J. Am. Chem. Soc.* **2020**, *142*, 21410-21419.
15. Jiang, W.-T.; Xu, M.-Y.; Yang, S.; Xie, X.-Y.; Xiao, B., Alkylation-Terminated Catellani Reactions Using Alkyl Carbagermatranes. *Angew. Chem. Int. Ed.* **2020**, *59*, 20450-20454.
16. Qian, L.; Fu, J.; Yuan, P.; Du, S.; Huang, W.; Li, L.; Yao, S. Q., Intracellular Delivery of Native Proteins Facilitated by Cell-Penetrating Poly(disulfide)s. *Angew. Chem. Int. Ed.* **2018**, *57*, 1532-1536.
17. Barré, B.; Gonnard, L.; Campagne, R.; Reymond, S.; Marin, J.; Ciapetti, P.; Brellier, M.; Guérinot, A.; Cossy, J., Iron- and Cobalt-Catalyzed Arylation of Azetidines, Pyrrolidines, and Piperidines with Grignard Reagents. *Org.*

Lett. **2014**, *16*, 23, 6160-6163.
18. Hofmayer, M. S.; Hammann, J. M.; Haas, D.; Knochel, P., Cobalt-Catalyzed C(*sp2*)–C(*sp3*) Cross-Coupling Reactions of Diarylmanganese Reagents with Secondary Alkyl Iodides. *Org. Lett.* **2016**, *18*, 24, 6456-6459.
19. Artaryan, A.; Mardyukov, A.; Kulbitski, K.; Avigdori, I.; Nisnevich, G. A.; Schreiner, P. R.; Gandelman, M., Aliphatic C–H Bond Iodination by a N-Iodoamide and Isolation of an Elusive N-Amidyl Radical. *J. Org. Chem.* **2017**, *82*, 14, 7093-7100.
20. Barolo, S. M.; Lukach, A. E.; Rossi, R. A., Syntheses of 2-substituted indoles and fused indoles by photostimulated reactions of o-iodoanilines with carbanions by the SRN1 mechanism. *J. Org. Chem.* **2003**, *68*, 7, 2807-2811.
21. Ellwood, A. R.; Porter, M. J., Selective Conversion of Alcohols into Alkyl Iodides Using a Thioiminium Salt. *J. Org. Chem.* **2009**, *74*, 7982-7985.
22. Xue, W.; Qu, Z.-W.; Grimme, S.; Oestreich, M., Copper-Catalyzed Cross-Coupling of Silicon Pronucleophiles with Unactivated Alkyl Electrophiles Coupled with Radical Cyclization. *J. Am. Chem. Soc.* **2016**, *138*, 43, 14222-14225.
23. Mahecha-Mahecha, C.; Lecornué, F.; Akinari, S.; Charote, T.; Gamba-Sánchez, D.; Ohwada, T.; Thibaudeau, S., Sequential Suzuki-Miyaura Coupling/Lewis Acid-Catalyzed Cyclization: An Entry to Functionalized Cycloalkane-Fused Naphthalenes. *Org. Lett.* **2020**, *22*, 16, 6267-6271.
24. Murray, J. I.; Spivey, A. C., Aminesvs. N-Oxides as Organocatalysts for Acylation, Sulfonylation and Silylation of Alcohols: 1-MethylimidazoleN-Oxide as an Efficient Catalyst for Silylation of Tertiary Alcohols. *Adv. Synth. Catal.* **2015**, *357*, 3825-3830.
25. Andersen, C.; Ferey, V.; Daumas, M.; Bernardelli, P.; Guérinot, A.; Cossy, J., Copper-Catalyzed Cross-Coupling between Alkyl (Pseudo)halides and Bicyclopentyl Grignard Reagents. *Org. Lett.* **2020**, *22*, 6021-6025.
26. Chen, H.; Jia, X.; Yu, Y.; Qian, Q.; Gong, H., Nickel-Catalyzed Reductive Allylation of Tertiary Alkyl Halides with Allylic Carbonates. *Angew. Chem. Int. Ed.* **2017**, *56*, 13103-13106.
27. Kiyokawa, K.; Ito, R.; Takemoto, K.; Minakata, S., C–H oxygenation at tertiary carbon centers using iodine oxidant†. *Chem. Commun.* **2018**, *54*, 7609.
28. Sun, K.; Liu, S.; Bec, P. M.; Driver, T. G., Rhodium-Catalyzed Synthesis of 2,3-Disubstituted Indoles from β,β-Disubstituted Stryryl Azides†. *Angew. Chem. Int. Ed.* **2011**, *50*, 1702-1706.
29. Sosič, I.; Anderluh, M.; Sova, M.; Gobec, M.; Mlinarič Raščan, I.; Derouaux, A.; Amoroso, A.; Terrak, M.; Breukink, E.; Gobec, S., Structure–Activity Relationships of Novel Tryptamine-Based Inhibitors of Bacterial Transglycosylase. *J. Med. Chem.* **2015**, *58*, 24, 9712-9721.
30. Bartolucci, S.; Mari, M.; Bedini, A.; Piersanti, G.; Spadoni, G., Iridium-Catalyzed Direct Synthesis of Tryptamine Derivatives from Indoles: Exploiting N-Protected β-Amino Alcohols as Alkylating Agents. *J. Org. Chem.* **2015**, *80*, 6, 3217-3222.
31. Gaussian 16, Revision A.03, M. J. Frisch, G. W. Trucks, H. B. Schlegel, G. E. Scuseria, M. A. Robb, J. R. Cheeseman, G. Scalmani, V. Barone, G. A. Petersson, H. Nakatsuji, X. Li, M. Caricato, A. V. Marenich, J. Bloino, B. G. Janesko, R. Gomperts, B. Mennucci, H. P. Hratchian, J. V. Ortiz, A. F. Izmaylov, J. L. Sonnenberg, D. Williams-Young, F. Ding, F. Lipparini, F. Egidi, J. Goings, B. Peng, A. Petrone, T. Henderson, D. Ranasinghe, V. G. Zakrzewski, J. Gao, N. Rega, G. Zheng, W. Liang, M. Hada, M. Ehara, K. Toyota, R. Fukuda, J. Hasegawa, M. Ishida, T. Nakajima, Y. Honda, O. Kitao, H. Nakai, T. Vreven, K. Throssell, J. A. Montgomery, Jr., J. E. Peralta, F. Ogliaro, M. J. Bearpark, J. J. Heyd, E. N. Brothers, K. N. Kudin, V. N. Staroverov, T. A. Keith, R. Kobayashi, J. Normand, K. Raghavachari, A. P. Rendell, J. C. Burant, S. S. Iyengar, J. Tomasi, M. Cossi, J. M. Millam, M. Klene, C. Adamo, R. Cammi, J. W. Ochterski, R. L. Martin, K. Morokuma, O. Farkas, J. B. Foresman, and D. J. Fox, Gaussian, Inc., Wallingford CT, **2016**.

32. S. Grimme, *J. Comput. Chem.* **2006**, *27*, 1787.

33. A. V. Marenich, C. J. Cramer, D. G. Truhlar, *J. Phys. Chem. B.,* **2009**, *113*, 6378.
