# Supplementary material for: Transition-metal free C–N bond formation from alkyl iodides and diazonium salts via halogen-atom transfer
Source: Nat Commun. 2022 Dec 27;13:7961. doi: 10.1038/s41467-022-35613-7 (PMC9794826; doi:10.1038/s41467-022-35613-7)
Supplement: Supplementary file 2 — Description of Additional Supplementary Data 1 [file 41467_2022_35613_MOESM2_ESM.docx]

**File Name:** Supplementary Data 1

**Description:** Cartesian coordinates of the optimized structures for computational details of DFT (DFT supported mechamism).
