# Supplementary material for: Transition-metal free C–N bond formation from alkyl iodides and diazonium salts via halogen-atom transfer
Source: Nat Commun. 2022 Dec 27;13:7961. doi: 10.1038/s41467-022-35613-7 (PMC9794826; doi:10.1038/s41467-022-35613-7)
Supplement: Supplementary file 3 — Supplementary Data 1 [file 41467_2022_35613_MOESM3_ESM.docx]

**Cartesian coordinates of the optimized structures:**

**1a**

E = -879.750273846 a.u.

Zero-point Energies = -879.600038 a.u.

Thermal correction to Gibbs Free Energy = 0.107647 a.u.

Single point Energy(6-311++G(2d,2p)) = -880.091365898 a.u.

Single point Energy(def2-TZVP) = -880.171667388 a.u.

0 1

C -1.53147600 -2.04128700 -0.06049600

C -0.16575700 -1.90904500 -0.10275100

C 0.39246300 -0.68150700 0.29651900

C -0.37333500 0.40033600 0.73928400

C -1.74590500 0.24292500 0.78202700

C -2.33351500 -0.96621800 0.37847700

H -2.01691600 -2.96129800 -0.36372200

H 0.46655400 -2.72164500 -0.44305600

H 0.13176300 1.31439800 1.03722900

H -2.35067400 1.07373200 1.12243200

N 2.87147500 -0.58904300 0.20525200

N 1.76441200 -0.57277400 0.24799600

B 2.36067200 2.34610500 -0.05870100

F 1.85772400 2.09578000 1.26085500

F 2.42824500 3.69002400 -0.31280100

F 1.46839300 1.69086400 -0.95446100

F 3.61169500 1.70953000 -0.15715400

O -3.64559300 -1.20188800 0.37554500

C -4.53519900 -0.17089100 0.78318200

H -4.43914400 0.70738100 0.13613300

H -5.53471500 -0.59160300 0.68352200

H -4.35720400 0.11026600 1.82671700

**Int1**

E = -570.496943935 a.u.

Zero-point Energies = -570.320025 a.u.

Thermal correction to Gibbs Free Energy = 0.137833 a.u.

Single point Energy(6-311++G(2d,2p)) = -570.682595994 a.u.

Single point Energy(def2-TZVP) = -570.727397861 a.u.

0 1

C -0.89650300 -2.22059300 0.53303500

C 0.16122700 -2.78703900 1.21575500

C 1.35272500 -2.07202400 1.35643800

C 1.49092200 -0.80227200 0.81563500

C 0.42414100 -0.23302600 0.12519300

C -0.77367300 -0.93731000 -0.01862100

H -1.83385600 -2.75195800 0.41097300

H 0.08140500 -3.77937200 1.64511900

H 2.42109500 -0.26611100 0.93253800

H 0.54558400 0.75885500 -0.29326900

N 2.51554100 -3.80278700 2.49887500

N 2.42172300 -2.72748000 2.07424900

O 3.57496300 -1.61337700 2.22084900

C 4.66472000 -2.16060600 2.88610600

H 4.44433000 -3.20691600 3.16503300

H 5.55322400 -2.15767100 2.24117500

H 4.87138300 -1.59828700 3.80559300

O -1.86365500 -0.47351200 -0.66665800

C -1.80341700 0.81473700 -1.24164700

H -2.77706100 0.98214900 -1.70268200

H -1.62488100 1.58466300 -0.48069700

H -1.02292000 0.87399100 -2.01045400

**^oss^TS1-2**

E = -570.458977826 a.u.

Zero-point Energies = -570.288318 a.u.

Thermal correction to Gibbs Free Energy = 0.127889 a.u.

Single point Energy(6-311++G(2d,2p)) = -570.650737820 a.u.

Single point Energy(def2-TZVP) = -570.695460183 a.u.

0 1

C -1.80768300 -1.34956500 0.29876100

C -0.46096100 -1.07399500 0.07739300

C -0.01602900 0.23767300 0.02347700

C -0.91000900 1.30053900 0.18971600

C -2.24633500 1.03459600 0.40918600

C -2.70542700 -0.29176200 0.46554700

H -2.13872600 -2.38029900 0.33847300

H 0.25143700 -1.88250300 -0.05392500

H -0.54370200 2.32115000 0.14485500

H -2.96562100 1.83548300 0.54227500

N 1.92640800 1.49854900 -0.27287500

N 1.39346600 0.44649000 -0.20860500

O 4.87302400 -0.79079500 -0.11008100

C 3.94138100 -1.74432700 -0.36114400

H 4.03653400 -2.20417000 -1.35944500

H 3.88035000 -2.51798000 0.42359400

H 2.96694100 -1.20498000 -0.35112400

O -4.02907700 -0.43802300 0.68509600

C -4.55730100 -1.74584200 0.75521500

H -5.62549100 -1.62736800 0.93761600

H -4.40791000 -2.28853400 -0.18654400

H -4.10910600 -2.31441800 1.57956500

**^3^TS1-2**

E = -570.439395744 a.u.

Zero-point Energies = -570.266597 a.u.

Thermal correction to Gibbs Free Energy = 0.132574 a.u.

Single point Energy(6-311++G(2d,2p)) = -570.627416707 a.u.

Single point Energy(def2-TZVP) = -570.672070048 a.u.

0 3

C -2.56795800 -1.66093600 0.79818300

C -1.23719100 -2.03627100 0.66721400

C -0.28695000 -1.12780900 0.20015500

C -0.67850400 0.17402300 -0.15806300

C -2.00008200 0.54794100 -0.03774800

C -2.95454800 -0.36299100 0.44491400

H -3.28869400 -2.38057900 1.16771100

H -0.91754300 -3.03986200 0.92825900

H 0.06672700 0.86101100 -0.54414700

H -2.33163600 1.54338900 -0.31322100

N 1.97605300 -0.82331100 -0.14522800

N 1.03881600 -1.57604100 0.06923100

O 2.16625500 -0.52224800 -1.86160500

C 3.40060400 0.11364100 -1.99539900

H 3.47264600 1.02956900 -1.39384000

H 4.24348700 -0.54823200 -1.75732500

H 3.46408000 0.38719600 -3.05765700

O -4.21725100 0.10783300 0.52632900

C -5.22803700 -0.75811400 0.99734300

H -6.15113300 -0.17834800 0.97862100

H -5.33746500 -1.63582900 0.34823500

H -5.02682700 -1.08689200 2.02456900

**Int2**

E = -455.447760360 a.u.

Zero-point Energies = -455.315638 a.u.

Thermal correction to Gibbs Free Energy = 0.097607 a.u.

Single point Energy(6-311++G(2d,2p)) = -455.592241784 a.u.

Single point Energy(def2-TZVP) = -455.628833654 a.u.

0 2

C 2.50606100 -1.13758000 -4.17240000

C 3.08187100 -1.46780300 -2.96201800

C 4.40343400 -1.09057100 -2.70167200

C 5.13450000 -0.38996100 -3.64839000

C 4.56041800 -0.05314300 -4.87187700

C 3.24084600 -0.42800200 -5.13620000

H 1.48358800 -1.41529200 -4.40506300

H 2.52435200 -2.01587600 -2.20914900

H 6.15805700 -0.10873100 -3.42238700

H 5.14634200 0.49484500 -5.59999800

N 4.57180600 -1.99314800 -0.56035300

N 5.06819700 -1.40345200 -1.45650500

O 2.58221900 -0.15707400 -6.28437100

C 3.26424600 0.55380100 -7.29532600

H 2.55578000 0.65913500 -8.11719200

H 3.56875100 1.54942300 -6.94902100

H 4.14701400 0.00417800 -7.64545600

**TS2-3**

E = -455.426410107 a.u.

Zero-point Energies = -455.297549 a.u.

Thermal correction to Gibbs Free Energy = 0.093215 a.u.

Single point Energy(6-311++G(2d,2p)) = -455.570739368 a.u.

Single point Energy(def2-TZVP) = -455.607417403 a.u.

0 2

C 3.20157000 -1.15174200 -4.71642900

C 3.53286000 -1.67468900 -3.46207900

C 4.45654600 -0.99726500 -2.70738800

C 5.08322600 0.16644300 -3.09959500

C 4.75204500 0.68348000 -4.34615800

C 3.81295600 0.02722900 -5.15518200

H 2.47399100 -1.66953600 -5.33118100

H 3.07105600 -2.58957400 -3.09952000

H 5.80785200 0.66069900 -2.45974400

H 5.20645300 1.59592300 -4.71859200

N 4.43201900 -2.64430900 -0.63715100

N 4.93442700 -1.69283200 -0.97473500

O 3.56470000 0.61812500 -6.35067500

C 2.63042300 0.00962400 -7.21275000

H 1.63358100 -0.04101100 -6.75565300

H 2.58620400 0.64027700 -8.10136500

H 2.94753800 -1.00075700 -7.50219800

**Int3**

E = -455.441620758 a.u.

Zero-point Energies = -455.313331 a.u.

Thermal correction to Gibbs Free Energy = 0.086203 a.u.

Single point Energy(6-311++G(2d,2p)) = -455.587277185 a.u.

Single point Energy(def2-TZVP) = -455.624419632 a.u.

0 2

C 3.25859600 -1.03863800 -4.76142300

C 3.66228300 -1.47789800 -3.49187200

C 4.59470300 -0.72612500 -2.82364800

C 5.16485700 0.43174200 -3.30994300

C 4.75901000 0.86135400 -4.57067700

C 3.80859200 0.12930300 -5.29562500

H 2.52248200 -1.61579000 -5.30994800

H 3.23938800 -2.38493100 -3.06945900

H 5.90089400 0.99667100 -2.74593600

H 5.16585400 1.76407200 -5.01566500

N 4.14074900 -3.21756000 -0.43184900

N 4.85550900 -2.45628700 -0.08099200

O 3.48742900 0.64125400 -6.51329800

C 2.53862500 -0.04866400 -7.29206100

H 1.56535000 -0.10563500 -6.78667900

H 2.43075800 0.52442500 -8.21385700

H 2.87766700 -1.06420800 -7.53626300

**Int4**

E = -345.957241146 a.u.

Zero-point Energies = -345.835171 a.u.

Thermal correction to Gibbs Free Energy = 0.090498 a.u.

Single point Energy(6-311++G(2d,2p)) = -346.069192942 a.u.

Single point Energy(def2-TZVP) = -346.095948476 a.u.

0 2

C 2.52652800 -1.01587600 -4.14316400

C 3.05725200 -1.27782900 -2.88286500

C 4.34475400 -0.84760900 -2.63659500

C 5.13150500 -0.18393800 -3.54330300

C 4.58980500 0.07618000 -4.81104100

C 3.28947100 -0.34083800 -5.10583900

H 1.51977100 -1.32579300 -4.40560700

H 2.46799700 -1.80076700 -2.13557700

H 6.14179000 0.13765200 -3.30797100

H 5.19256200 0.59947600 -5.54502800

O 2.67105000 -0.13977900 -6.29981200

C 3.38525200 0.53261800 -7.30983500

H 2.71530900 0.58473900 -8.16903800

H 3.65784500 1.55112400 -7.00271700

H 4.29464900 -0.01337600 -7.59391500

**Int5**

E = -553.284585717 a.u.

Zero-point Energies = -553.029070 a.u.

Thermal correction to Gibbs Free Energy = 0.203956 a.u.

Single point Energy(6-311++G(2d,2p)) = -553.462764066 a.u.

Single point Energy(def2-TZVP) = -839.860384081 a.u.

0 2

C 2.93044300 1.23185200 -1.39048300

C 3.58063300 0.02455500 -1.40663200

C 4.91020700 -0.00306100 -0.96103300

C 5.51700900 1.17678200 -0.52299800

C 4.80581100 2.38451200 -0.52667800

C 3.48565400 2.42120200 -0.96726500

H 3.09812400 -0.88611100 -1.74861500

H 5.44825700 -0.94455200 -0.96369700

H 5.30829200 3.28195700 -0.17955200

H 2.92798000 3.35271500 -0.97233100

I -0.13976700 1.26999300 -2.39991700

C -3.18321900 -0.44318900 -1.51161400

C -2.84361400 -0.14830100 -2.98651200

C -2.27788700 1.26833800 -2.98509700

C -3.13044400 1.98671100 -1.94404400

C -3.36662800 0.94302500 -0.83430800

H -4.07778100 -1.06686200 -1.42931700

H -2.36386800 -0.99213700 -1.03840400

H -3.76396100 -0.11784200 -3.58693100

H -2.18273600 -0.88928600 -3.44178200

H -2.26361900 1.74765300 -3.96319100

H -2.69544200 2.92115400 -1.58258400

H -4.08132200 2.23673300 -2.43585600

H -2.63453400 1.07413700 -0.03217700

H -4.35790000 1.05868700 -0.38715500

O 6.79723000 1.25884300 -0.07217200

C 7.56523100 0.07925000 -0.04324500

H 8.54351800 0.36549600 0.34521800

H 7.12119300 -0.67619000 0.61869500

H 7.68691900 -0.34875400 -1.04721700

**TS5-6**

E = -553.280490003 a.u.

Zero-point Energies = -553.025823 a.u.

Thermal correction to Gibbs Free Energy = 0.206636 a.u.

Single point Energy(6-311++G(2d,2p)) = -553.458424753 a.u.

Single point Energy(def2-TZVP) = -839.857451446 a.u.

0 2

C 2.52316100 1.22482000 -1.50388900

C 3.19539400 0.02469900 -1.50094400

C 4.52365200 0.00512900 -1.05775900

C 5.12837200 1.18968600 -0.63238300

C 4.40989000 2.39180500 -0.64880300

C 3.09105000 2.41572200 -1.08806300

H 2.72053700 -0.89369600 -1.83178400

H 5.06333300 -0.93536300 -1.05239300

H 4.90618800 3.29657700 -0.31222300

H 2.53305300 3.34664500 -1.10052000

I 0.10461500 1.25885100 -2.31697300

C -3.02450200 -0.45216300 -1.47872300

C -2.68210200 -0.16071100 -2.95447800

C -2.15600500 1.26155600 -2.95963300

C -2.97729400 1.98189100 -1.90806600

C -3.21019500 0.93385400 -0.80088900

H -3.91859700 -1.07647900 -1.39545300

H -2.20466300 -0.99917700 -1.00420300

H -3.59798800 -0.16431800 -3.56519600

H -1.99856900 -0.88871600 -3.39826900

H -2.07932100 1.74662300 -3.93175500

H -2.52294100 2.90770000 -1.54672300

H -3.93325400 2.25440000 -2.38117500

H -2.47615600 1.06557700 -0.00069800

H -4.20075900 1.04531800 -0.35099100

O 6.40813900 1.28124700 -0.18444700

C 7.18085400 0.10474000 -0.14553900

H 8.15879400 0.39860200 0.23801500

H 6.74095200 -0.64567000 0.52471400

H 7.30194300 -0.33243900 -1.14554800

**Int6**

E = -553.305243680 a.u.

Zero-point Energies = -553.051819 a.u.

Thermal correction to Gibbs Free Energy = 0.203806 a.u.

Single point Energy(6-311++G(2d,2p)) = -553.482946388 a.u.

Single point Energy(def2-TZVP) = -839.882422364 a.u.

0 2

C 2.56688700 1.24968800 -1.55581500

C 3.30321800 0.07490900 -1.57620800

C 4.62564600 0.06811700 -1.13042000

C 5.20668500 1.24737300 -0.66346000

C 4.45580700 2.42777300 -0.64674800

C 3.14282300 2.43127000 -1.09011500

H 2.86203100 -0.84811000 -1.93686500

H 5.18124900 -0.86216900 -1.15605900

H 4.92292000 3.33597500 -0.28066400

H 2.57314700 3.35409300 -1.07091600

I 0.54320700 1.25040000 -2.23606700

C -3.84380100 -0.38386500 -1.64815700

C -2.99356400 -0.29899700 -2.93395300

C -2.72858500 1.16982600 -3.08863200

C -3.21753200 1.94084300 -1.90058200

C -3.42182000 0.84919300 -0.83373400

H -4.90746400 -0.30726000 -1.90598300

H -3.70283600 -1.32282900 -1.10456000

H -3.49819400 -0.73585800 -3.80591300

H -2.05365000 -0.86253100 -2.81328100

H -2.41776500 1.63115000 -4.02082800

H -2.52776300 2.73168000 -1.57954100

H -4.17641600 2.43974400 -2.12639100

H -2.46860700 0.65230500 -0.32792300

H -4.15424100 1.12717800 -0.06981100

O 6.47918800 1.35275200 -0.20916700

C 7.27989700 0.19252400 -0.20516000

H 8.24878700 0.49703300 0.19201300

H 6.85424600 -0.58804300 0.43874400

H 7.41408200 -0.20745400 -1.21859800

**Int7**

E = -195.831518645 a.u.

Zero-point Energies = -195.703798 a.u.

Thermal correction to Gibbs Free Energy = 0.099301 a.u.

Single point Energy(6-311++G(2d,2p)) = -195.895867170 a.u.

Single point Energy(def2-TZVP) = -195.906880114 a.u.

0 2

C -0.30701300 -0.80883600 -1.50034500

C 1.18070000 -0.82891600 -1.88490600

C 1.66461500 0.60303500 -1.57824500

C 0.44055600 1.43730400 -1.79818100

C -0.78657300 0.59033900 -1.93805000

H -0.87787000 -1.62160800 -1.95958100

H -0.40754900 -0.90680800 -0.41227200

H 1.28076500 -1.02673200 -2.95935200

H 1.74917900 -1.59786500 -1.35292900

H 2.01284600 0.67465700 -0.53312900

H 2.51529700 0.90717200 -2.20244400

H -1.63500400 0.95395400 -1.34334000

H -1.13655700 0.56618800 -2.98477100

H 0.44217100 2.52066100 -1.84726600

**Int8**

E = -1075.59378622 a.u.

Zero-point Energies = -1075.314243 a.u.

Thermal correction to Gibbs Free Energy = 0.226256 a.u.

Single point Energy(6-311++G(2d,2p)) = -1075.99248930 a.u.

Single point Energy(def2-TZVP) = -1076.08282458 a.u.

0 2

C -1.45935100 -0.41909600 -1.10243400

C -1.44181500 -0.67399300 0.24353600

C -1.83145400 0.34800300 1.12789200

C -2.25437700 1.62170500 0.68415800

C -2.25195100 1.86274100 -0.66514700

C -1.79731100 0.86664800 -1.56468800

H -1.09015700 -1.18874900 -1.77215900

H -1.04370200 -1.61703900 0.59751700

H -2.54541200 2.38657100 1.39547300

H -2.53658600 2.83038200 -1.06132500

N -1.67871800 0.00480900 3.57143700

N -1.68569900 0.14365700 2.46207000

C 1.24048800 0.08423700 1.89498100

C 1.42949100 1.31460300 1.06737000

C 1.99875500 2.34946900 2.05827900

C 1.45982700 1.89879200 3.42552900

C 1.49943200 0.36095700 3.33921800

H 0.47081100 1.66570900 0.64599000

H 2.06028600 1.11576600 0.19566300

H 3.09358700 2.29000000 2.06088200

H 1.72720000 3.38039800 1.81146500

H 2.02926200 2.29872400 4.26943000

H 0.41992300 2.23599500 3.54547300

H 2.49334100 -0.01799000 3.63108700

H 0.79046000 -0.13290000 4.02277700

O -1.69216500 1.23374100 -2.82763300

C -1.00970600 0.35066800 -3.73712200

H -0.87234600 0.93527000 -4.64510200

H -0.05121400 0.04229000 -3.31347300

H -1.62595400 -0.52826100 -3.94581400

B 1.33656300 -1.88550800 -1.47272700

F 0.86090400 -2.30268800 -0.19241500

F 1.37489300 -0.46621900 -1.48992900

F 0.38210000 -2.29534600 -2.44180100

F 2.57451500 -2.41997900 -1.73016600

H 1.20366000 -0.90514800 1.44799700

**TS8-9**

E = -1075.59101250 a.u.

Zero-point Energies = -1075.311418 a.u.

Thermal correction to Gibbs Free Energy = 0.227212 a.u.

Single point Energy(6-311++G(2d,2p)) = -1075.99081378 a.u.

Single point Energy(def2-TZVP) = -1076.08144662 a.u.

0 2

C 1.46664200 0.12482400 -3.78408900

C 1.47957400 -0.03571100 -2.42304000

C 0.82954500 0.92940300 -1.62681000

C 0.14548000 2.04279700 -2.17264000

C 0.16499200 2.19689200 -3.53058200

C 0.87625900 1.27239000 -4.34354700

H 1.99989400 -0.59887900 -4.39289100

H 2.04414000 -0.85543700 -1.99467600

H -0.34760700 2.76068100 -1.52684000

H -0.31206900 3.04540600 -4.00672000

N 1.27490000 0.75285900 0.78766100

N 1.01169300 0.84968100 -0.29161300

C 4.16858900 0.59594700 -0.37163600

C 4.17241500 1.82185500 -1.23967400

C 5.14297300 2.77526700 -0.51517900

C 4.97407700 2.40899300 0.96758600

C 4.83391800 0.87485300 0.94555700

H 3.17435200 2.29851900 -1.28553600

H 4.44091400 1.58362400 -2.27396700

H 6.16956200 2.55921900 -0.83380900

H 4.94675300 3.83211900 -0.72208500

H 5.80021200 2.75570400 1.59550800

H 4.05165800 2.86064400 1.35813000

H 5.82834700 0.39977000 0.97242200

H 4.28830500 0.47925200 1.81334700

O 0.95495600 1.57473700 -5.62229100

C 1.84383700 0.79994800 -6.45310200

H 1.88902000 1.34393000 -7.39471400

H 2.82831300 0.72711700 -5.98494600

H 1.43526000 -0.20105000 -6.61266000

B 4.40757700 -1.04120800 -4.11678200

F 3.93318000 -1.42993700 -2.82957900

F 4.32644300 0.37465500 -4.21390500

F 3.51098400 -1.57762200 -5.08220800

F 5.68964800 -1.48331700 -4.32172000

H 4.10695700 -0.39742300 -0.80565300

**Int9**

E = -1075.63499911 a.u.

Zero-point Energies = -1075.350605 a.u.

Thermal correction to Gibbs Free Energy = 0.233393 a.u.

Single point Energy(6-311++G(2d,2p)) = -1076.02194719 a.u.

Single point Energy(def2-TZVP) = -1076.11023890 a.u.

0 2

C -1.53049300 -0.93904200 -1.18095400

C -1.51520400 -0.77074300 0.17862600

C -1.99185600 0.43931800 0.73040800

C -2.62507800 1.41206300 -0.07734900

C -2.62436300 1.24358400 -1.43256200

C -2.02501200 0.09353900 -2.00436700

H -1.06661200 -1.81721800 -1.60764800

H -1.07762900 -1.52616300 0.81944900

H -3.03926400 2.30190700 0.38293500

H -3.02938200 1.99527900 -2.09964200

N -1.00180000 0.64597500 2.89244700

N -1.88000300 0.68575300 2.07448700

C 0.41234300 0.62360900 2.46577200

C 0.98478100 2.04852400 2.41327400

C 2.50566000 1.77659800 2.35678100

C 2.70778200 0.32641600 2.87570700

C 1.33632000 -0.11614700 3.41929700

H 0.70385900 2.59459100 3.32154300

H 0.61813100 2.58151400 1.53493400

H 2.85221600 1.85103900 1.32425200

H 3.04937100 2.51027000 2.95824200

H 2.98162600 -0.31725500 2.03565400

H 3.48981000 0.24787200 3.63540200

H 1.19520300 -1.20046100 3.39510900

H 1.17573600 0.23480900 4.44595700

O -2.00066400 0.06552600 -3.32381000

C -1.24804800 -0.95609100 -3.98685000

H -1.26593100 -0.68329400 -5.04086400

H -0.22543100 -0.95678000 -3.60433000

H -1.72923800 -1.93080900 -3.85234200

B 1.45676800 0.34344600 -0.94762600

F 1.33392900 -0.77459700 -0.06978500

F 0.51575500 1.32390500 -0.54471700

F 1.14221300 -0.07731200 -2.25208600

F 2.74101600 0.85741000 -0.86711500

H 0.46521500 0.19189200 1.46209000

**Int10**

E = -651.105994214 a.u.

Zero-point Energies = -650.838030 a.u.

Thermal correction to Gibbs Free Energy = 0.222024 a.u.

Single point Energy(6-311++G(2d,2p)) = -651.368447104 a.u.

Single point Energy(def2-TZVP) = -651.417305784 a.u.

1 2

C 2.50670700 -3.51950000 -4.34819400

C 3.28093800 -3.08496600 -3.29210900

C 3.51601200 -1.71284400 -3.11822600

C 2.97897700 -0.75769100 -4.01290200

C 2.21232600 -1.19193100 -5.06010400

C 1.96481500 -2.57562700 -5.24245200

H 2.32666200 -4.57915400 -4.47704300

H 3.70990800 -3.79504900 -2.59310700

H 3.17406500 0.29911100 -3.86260800

H 1.77833000 -0.49620000 -5.76912900

N 4.60487900 -0.28162600 -1.60667400

N 4.29933200 -1.34186100 -2.04892100

C 5.55754000 0.05454100 -0.52108700

C 6.71960200 0.88905700 -1.12103700

C 6.46131100 2.35229400 -0.68385900

C 5.01837400 2.37291600 -0.15833400

C 4.85126000 0.98758800 0.47026200

H 5.86712200 -0.90661000 -0.10044900

H 6.75551800 0.77898300 -2.20850700

H 7.66561000 0.50174200 -0.73458700

H 7.15258400 2.62501100 0.11934300

H 6.62112900 3.05790200 -1.50185000

H 4.83553000 3.17995400 0.55451000

H 4.30637200 2.49731800 -0.98419500

H 5.40473700 0.92609800 1.41442200

H 3.81766500 0.69396200 0.67170100

O 1.21161900 -2.87715700 -6.28559300

C 0.89516900 -4.24039800 -6.56831500

H 0.27289500 -4.21301900 -7.46057500

H 1.80544100 -4.81362300 -6.76944800

H 0.33531800 -4.68585400 -5.74019100

**3a**

E = -651.371781707 a.u.

Zero-point Energies = -651.103457 a.u.

Thermal correction to Gibbs Free Energy = 0.225623 a.u.

Single point Energy(6-311++G(2d,2p)) = -651.579027653 a.u.

Single point Energy(def2-TZVP) = -651.625154450 a.u.

0 1

C 2.34124800 -3.45877400 -4.28766800

C 3.00764500 -2.99410100 -3.15575200

C 3.45012100 -1.67959600 -3.07302700

C 3.22152200 -0.80703500 -4.14640700

C 2.56230600 -1.25572300 -5.27283500

C 2.11702500 -2.58511100 -5.35273300

H 2.00791000 -4.48926100 -4.32381100

H 3.19356800 -3.65520400 -2.31498400

H 3.56982900 0.21733300 -4.07433600

H 2.37395500 -0.59889500 -6.11575900

N 4.50875800 -0.14500200 -1.81506400

N 4.11842700 -1.32396200 -1.86529000

C 5.19032700 0.17888500 -0.56084100

C 6.64404000 0.62036900 -0.88759900

C 6.78112400 2.06407400 -0.37030100

C 5.35020100 2.60836100 -0.43204400

C 4.51696000 1.42179800 0.06234900

H 5.15351300 -0.69192000 0.10319900

H 6.77757800 0.59356900 -1.97473500

H 7.37925300 -0.06191500 -0.45209500

H 7.12921400 2.06309200 0.67043900

H 7.49489700 2.65317700 -0.95412100

H 5.20355600 3.51425100 0.16441200

H 5.08334800 2.84007300 -1.47041500

H 4.58074200 1.34929200 1.15468200

H 3.45806400 1.48415700 -0.20591400

O 1.48309200 -2.91918900 -6.50173300

C 1.01330100 -4.24116700 -6.64158300

H 0.54451100 -4.29146700 -7.62497200

H 1.83539800 -4.96690900 -6.59464800

H 0.26948400 -4.48684000 -5.87273500
